# Diverse Library of 5a-Substituted Carba-Glucosamines

**DOI:** 10.1021/acs.joc.4c02816

**Published:** 2025-02-15

**Authors:** Bjarne Silkenath, Dennis Kläge, Philip Eppelin, Jörg S. Hartig, Valentin Wittmann

**Affiliations:** Department of Chemistry, University of Konstanz, 78457 Konstanz, Germany

## Abstract

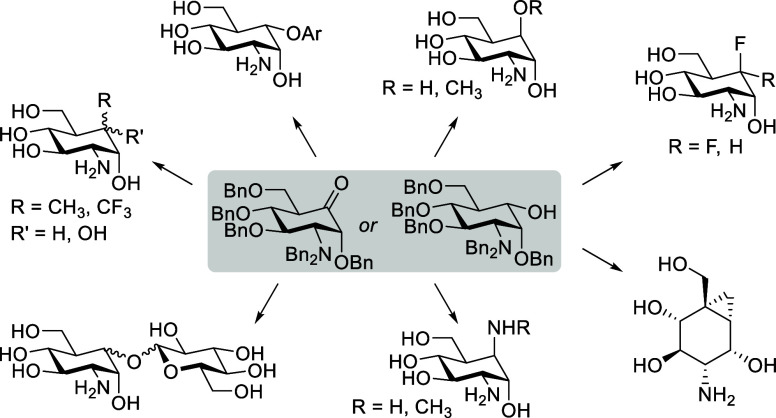

Carba-sugars—carbohydrate mimics in which the ring oxygen is replaced by a methylene group—are carbohydrate analogues of natural or synthetic origin that can have important biological functions. Especially, carba-aminosugars and glycosides containing carba-aminosugars are potent antibiotics. Furthermore, they have been shown to induce the self-cleavage reaction of the *glmS* riboswitch and thereby inhibit the ability of bacteria to synthesize glucosamine-6-phosphate, which is required to build up the bacterial cell wall. We report the synthesis of a library of 20 carba-glucosamine derivatives with various substituents at the carba-position including amines, alkyl, alkoxy, and aryloxy derivatives, fluorine derivatives, glycosylated derivatives, and a cyclopropane derivative. The compounds were obtained in an efficient way starting from late-stage synthetic intermediates of an earlier-developed synthesis of carba-substituted carba-glucosamines. All carba-glucosamine mimics were tested for their antibacterial properties against *Bacillus subtilis*, and some of them displayed promising activities in a filter disk assay.

## Introduction

**Figure 1:**
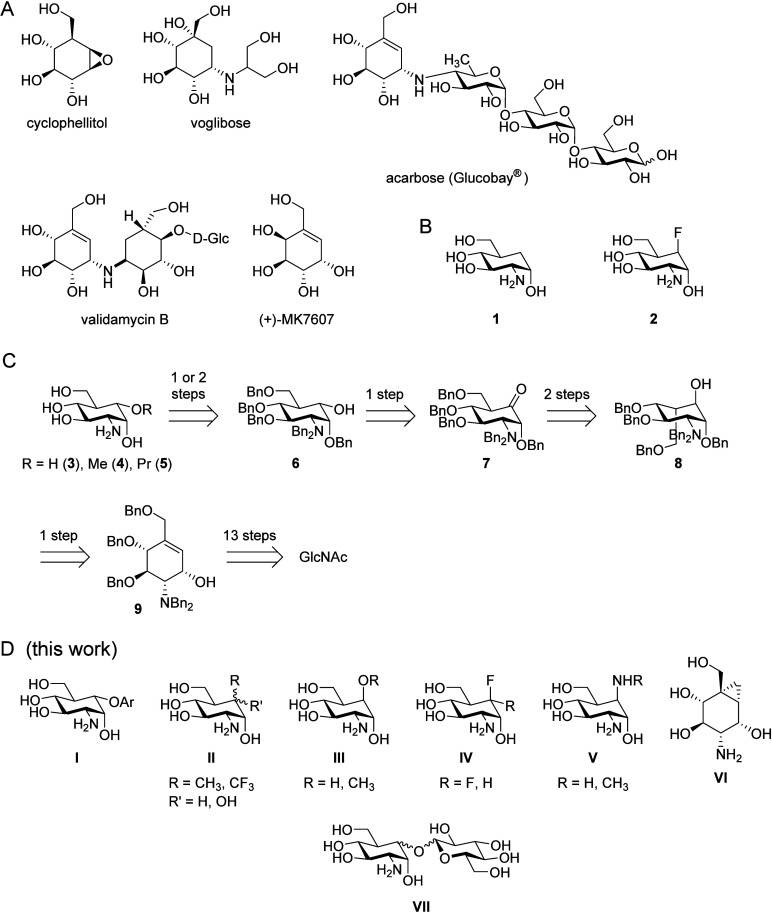
(A) Biologically active carba-sugars: glycosidase inhibitors cyclophellitol, voglibose, and acarbose and antimicrobial compounds validamycin B and (+)-MK7607. (B) Carba-glucosamine (carba-GlcN) analogues **1** and **2** with antibiotic activity that have been shown to activate the *glmS* riboswitch. (C) Retrosynthesis of published 5a-hydroxy- and 5a-alkoxy-modified carba-glucosamine mimics **3**–**5**^42^ with late-stage intermediates that served as starting materials for the carba-glucosamine mimics reported here. (D) Library of new carba-glucosamine mimics reported in this work.

Carba-sugars are carbohydrate mimics in which the ring oxygen, which is part of the hemiacetal function, is replaced by a methylene group.^1^ This position is termed the 5a position. A multitude of these compounds have been either extracted from nature or synthesized.^2−4^ Many carba-sugars possess interesting biological activities ranging from the inhibition of glycosidases used in part to treat diabetes (cyclophellitol, voglibose, and acarbose)^5−13^ to antimicrobial activity (validamycins and MK7607)^14−17^ (Figure 1A). Especially, carba-aminosugars and glycosides containing carba-aminosugars are potent antibiotics.^3^ Previously, we have shown that the carba-analogue **1** of glucosamine and fluorine derivative **2** possess antibiotic properties (Figure 1B).^18,19^ This was explained by their ability to catalyze the self-cleavage reaction of the *glmS* riboswitch and thereby inhibit the ability of bacteria to synthesize glucosamine-6-phosphate, which is required to build up the bacterial cell wall.^19,20^ The antibiotic properties of carba-aminosugars **1** and **2** sparked our interest in the synthesis of modified carba-glucosamines to fully assess the scope of these antimicrobial compounds. Especially, the modification of the 5a (carba) position was of interest to us as it offers room for derivatization not available in the parent carbohydrates. Previous synthetic strategies for carba-sugars provided one target structure at the end of the synthesis. These strategies used the Ferrier rearrangement,^21−24^ Diels–Alder chemistry,^25−31^ various aldol reactions,^32−34^ ring-closing metathesis,^35−39^ as well as microbial oxidations of benzene derivatives.^40,41^ To the best of our knowledge, none of these synthetic strategies allowed for selective derivatization of the 5a position unique to carba-sugars.^3,4^ In our previous research, we established for the first time a synthetic route toward carba-analogues **3**–**5** of glucosamine bearing equatorially oriented alkoxy substituents in the 5a position (Figure 1C).^42^ In light of the multitude of potential biological applications of these compounds, we envisioned the synthesis of a large library of 5a-substituted carba-aminosugars utilizing late-stage intermediates of our established synthesis. Such a library would allow a better understanding of the antibiotic potential of this compound class. The required flexible synthetic strategy would also be of high interest to other fields of research, such as the development of new antidiabetic compounds or glycosidase inhibitors.

Here, we report the synthesis of a diverse library of 20 carba-glucosamine mimics with various substituents at the 5a position (Figure 1D) making use of late-stage synthetic intermediates of our previously developed synthesis of 5a-substituted carba-glucosamines. Starting from 5a-hydroxy (**6**, **8**), 5a-oxo (**7**), or cyclohexene (**9**) derivatives, we employed different reaction types to achieve a variety of compounds with new substitution patterns at C-5a that were previously not accessible. We tested all carba-glucosamine mimics for their antibacterial properties against *Bacillus subtilis* and discovered promising activities for some compounds.

## Results and Discussion

Figure 1C depicts the retrosynthesis of the 5a-hydroxy and -alkoxy derivatives **3**–**5** that we published earlier.^42^ The synthesis starts with *N*-acetylglucosamine (GlcNAc) and delivers the target structures in 18–19 steps. Intermediates **6**–**9** occur at a late stage of the synthesis and therefore serve as ideal starting points for the diversification presented in the current study. We started the investigation of new 5a substituents with the introduction of aryloxy substituents (substitution type **I**). Reactions for the installation of phenoxy groups starting from an alcohol are rare.^43,44^ We opted for the generation of a bromophenoxy substituent via an S_N_Ar reaction, offering the possibility to remove the bromine to give a phenoxy substituent as well as to access more complex aryl modifications by subsequent cross-coupling reactions. Addition of C-nucleophiles to ketone **7** provided access to carba-glucosamine mimics of type **II** with 5a mono- and disubstitution. Reduction of **7** with CBS reagents provided carba-GlcN derivatives with substitution type **III** bearing axial alkoxy or hydroxy substituents. These compounds are stereoisomers of the equatorially substituted compounds **3**–**5** depicted in Figure 1C. It was further possible to introduce fluorine by fluoride addition to ketone **7** and subsequent optional deoxygenation resulting in substitution type **IV.** Reductive amination of the carbonyl of **7** yielded substitution type **V**, a carba-GlcN with an additional amine in the 5a position. A bicyclic carba-GlcN (substitution type **VI**) was prepared by Simmons–Smith cyclopropanation of **9**. It was also possible to glycosylate the hydroxy group in the 5a position of **6** and its epimer **III** (R = H) to generate a new type of carba-disaccharides (substitution type **VII**).

### 5a-Aryloxy-Substituted Carba-Glucosamine Mimics (Substitution Type I)

**Scheme 1:**
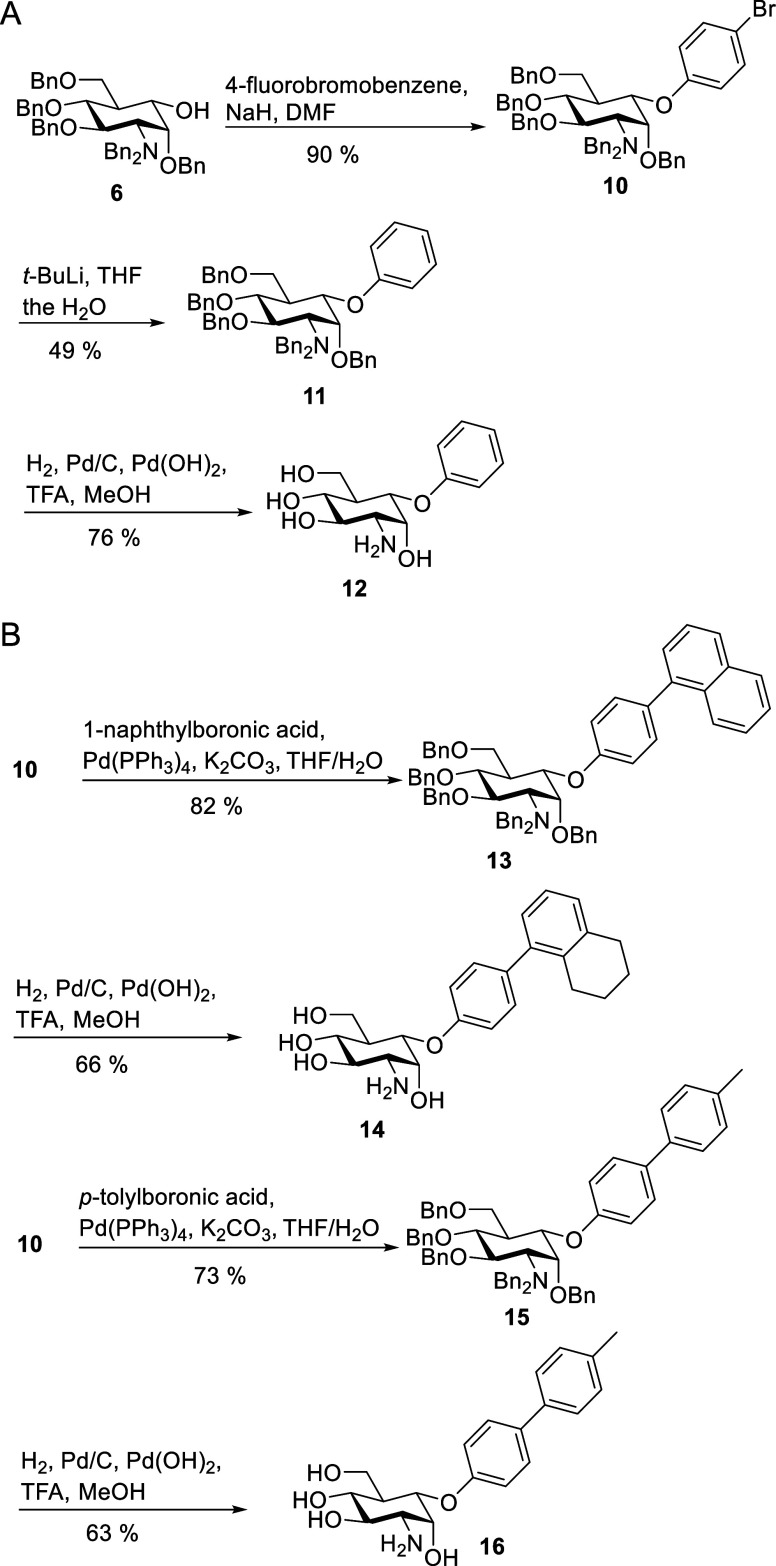
(A) Synthesis of Phenyloxy Derivative **12**; (B) Synthesis of Aryloxy Derivatives **14** and **16** Obtained by Suzuki–Miyaura Cross-Couplings

Carba-sugars bearing a phenoxy substituent in position 5a are promising antibacterial compounds for several reasons. The aryl moiety might allow them to intercalate in RNA structures, such as the *glmS* riboswitch, for which glucosamine mimics have been shown to be potent activators.^18−20^ Furthermore, the aryl moiety decreases the polarity of the glucosamine mimic to a point where passive diffusion into the bacterium can be considered reasonable. Ikegai reported the use of tetraphenyl bismuth fluoride for *O*-phenylation of cyclohexanol under copper catalysis.^43^ However, application of the reported conditions to alcohol **6** gave only trace amounts of the desired phenoxy carba-sugar **11** (Scheme 1A). In an alternative route, alcohol **6** was reacted in an S_N_Ar reaction with 4-fluorobromobenzene^45^ to give compound **10** in a yield of 90%. Compound **10** was successfully dehalogenated by treatment with *t*-BuLi and aqueous workup to give phenoxy-modified glucosamine mimic **11** in a yield of 49%. This compound was deprotected by hydrogenation using a mixture of Pd(0) on carbon and Pd(OH)_2_ on carbon as catalysts^46^ in the presence of trifluoroacetic acid to give compound **12** in a yield of 76%. This was achieved without affecting the aryl moiety at position 5a.

The intermediate aryl bromide **10** offered room for further modification. We decided to introduce larger aromatic residues to further decrease the polarity of the target compound and potentially facilitate intercalation into RNA. To achieve this, Suzuki–Miyaura cross-coupling^47^ was performed (Scheme 1B). With 1-naphthylboronic acid, the naphthyl-modified glucosamine **13** was obtained in a yield of 82%. When *p*-tolylboronic acid was used as the boronic acid, biphenyl compound **15** was isolated in a yield of 73%. When these compounds were subjected to hydrogenation, naphthyl derivative **13** reacted to tetrahydronaphthyl compound **14** in a yield of 66%. Compound **15** was deprotected to obtain **16** in a yield of 63%.

### 5a-Methyl-Substituted Carba-Glucosamine Mimics (Substitution Type II)

**Scheme 2:**
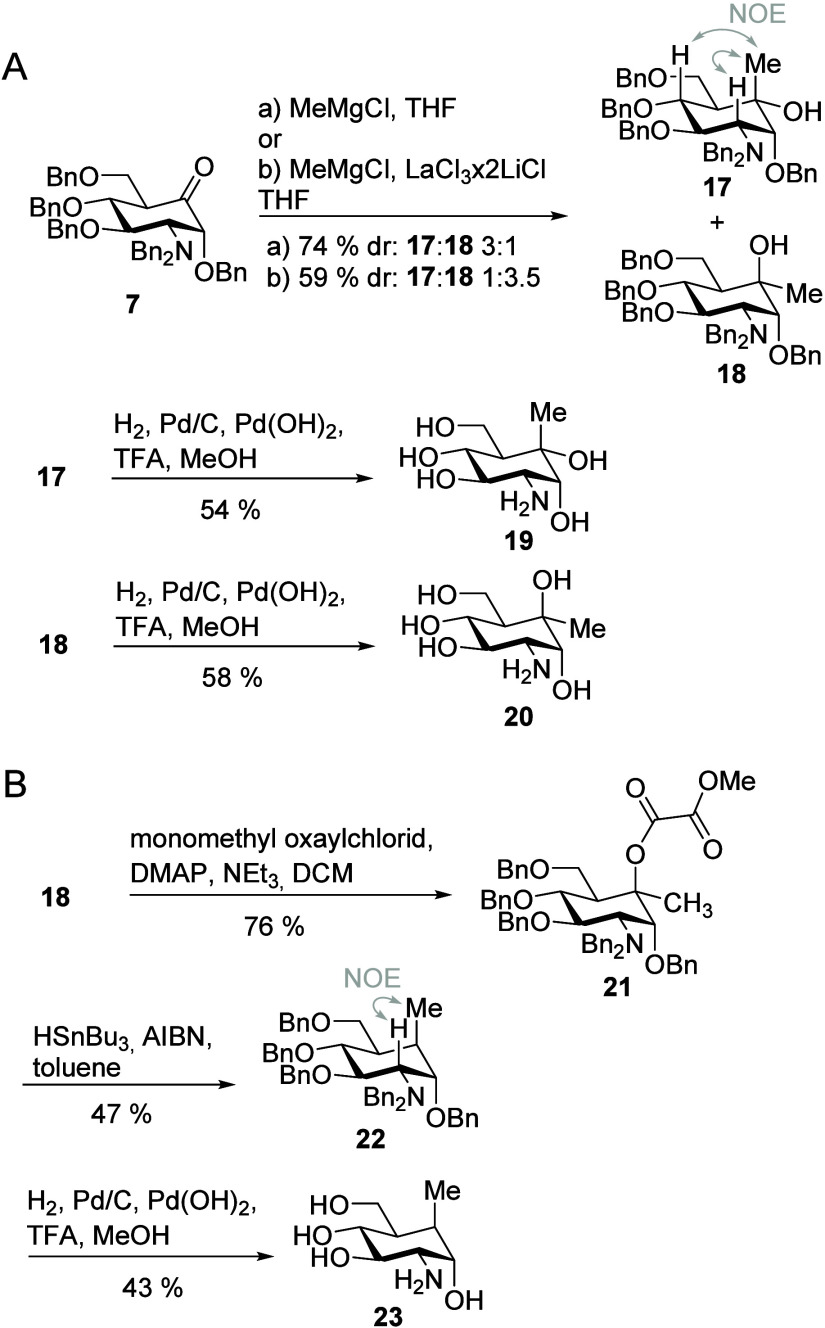
(A) Synthesis of 5a-Methyl-5a-hydroxy-Disubstituted Carba-Sugars **19** and **20**; (B) Deoxygenation of **18** and Deprotection to Give Carba-Glucosamine **23**

Carba-glucosamine mimics bearing an additional carbon–carbon bond in the 5a position have not yet been reported. To access this compound class, methyl magnesium chloride was reacted with ketone **7** resulting in a separable 3:1 mixture of compounds **17** and **18** in a yield of 74% (Scheme 2A). The assignment of the configuration at the newly formed stereocenter (position 5a) was possible by NMR spectroscopy. In isomer **17**, NOEs between the methyl group and H-2 as well as H-4 could be observed, which were missing in the NOESY spectrum of **18** (Scheme 2A and Supporting Information). The observed stereoselectivity of the attack of the Grignard reagent to this cyclohexanone derivative leading to the preferred formation of an axial methyl group is in accordance with previously published reactions of cyclohexanones.^48^ When adding LaCl_3_x2LiCl^49^ to compound **7** before the addition of the Grignard reagent, an inversion of the stereochemical outcome was observed, and a 1:3.5 mixture of **17** and **18** was obtained in a yield of 59%. A plausible explanation for the inverted stereoselectivity is the complexation of the lanthanide(III) salt to the carbonyl group, blocking the attack from the axial direction. Equatorial attack of organometallic reagents to cyclohexanones is also observed in the presence of CeCl_3_.^50^ The unprotected disubstituted carba-sugars **19** and **20** were obtained in a yield of 54 and 58%, respectively, upon hydrogenation.

Compounds **17** and **18** were expected to be promising starting materials for deoxygenation, allowing access to solely 5a-methyl-modified carba-glucosamine mimics. This approach, however, proved to be challenging, and when xanthates were employed for Barton–McCombie deoxygenation,^51^ only trace amounts of the desired products were observed. Alternative deoxygenation methods reported by Jang et al.^52^ and Yasuda et al.^53^ did not provide access to the methyl carba-glucosamines either. Conversion of tertiary alcohol **18** to methyl oxalic acid ester **21** according to Dolan and MacMillan,^54^ however, succeeded in a yield of 76%. Subsequent radical deoxygenation employing HSnBu_3_ and AIBN allowed for the synthesis of carba-glucosamine **22** in a yield of 47%. Hydrogen transfer from HSnBu_3_ to the intermediate C-5a radical exclusively led to the formation of an axial 5a-methyl group, as proven by a NOESY NMR spectrum. Interestingly, the same sequence starting from **17** proceeded with lower yields and stereoselectivity. Compound **22** was deprotected by hydrogenation to give methyl-modified carba-glucosamine **23** in a yield of 43%.

**Scheme 3:**
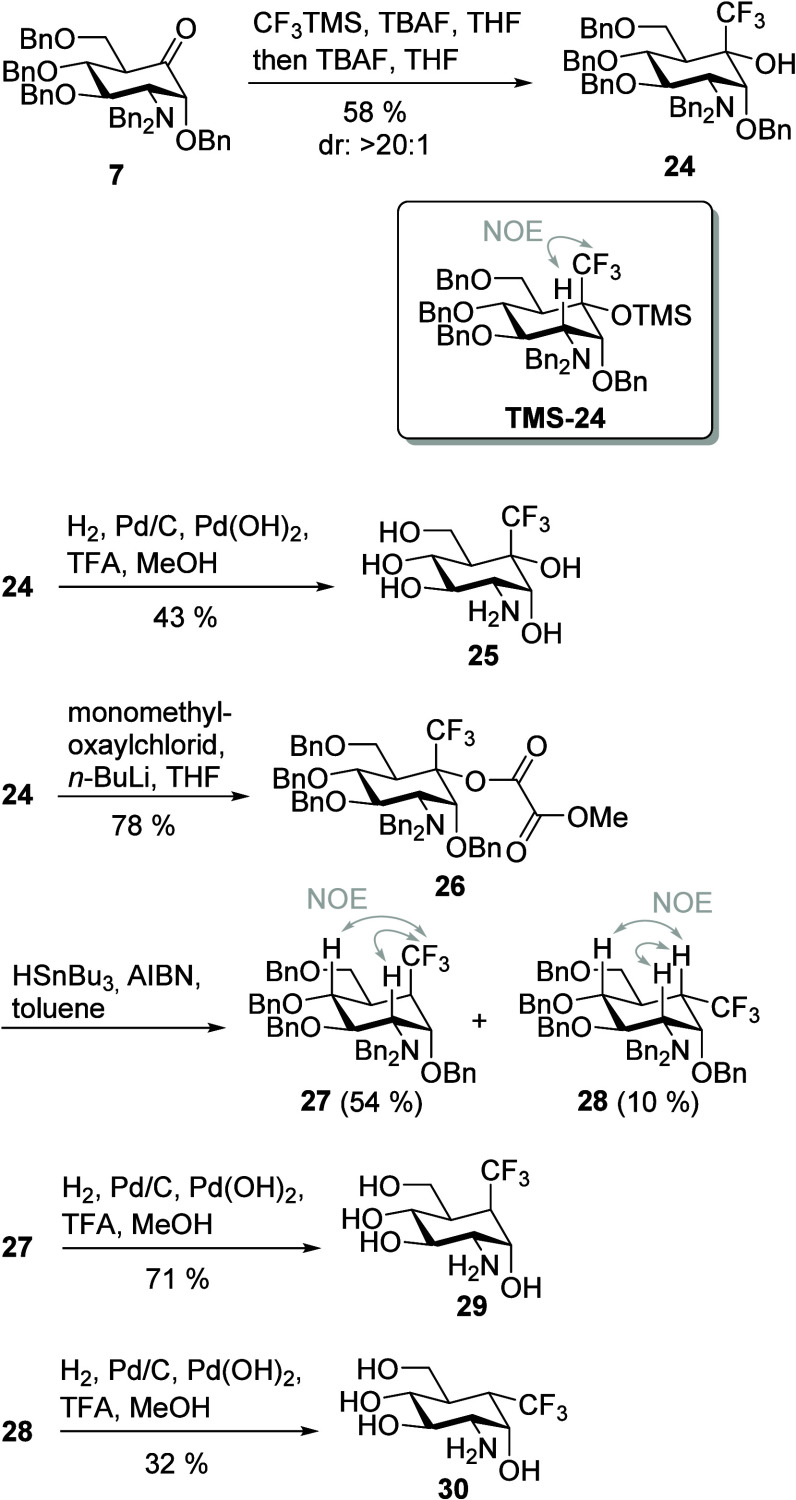
Trifluoromethylation of Ketone **6** Provided Access to 5a-CF_3_-Substituted Carba-Glucosamines **25**, **29**, and **30**

We next aimed for the introduction of an electron-withdrawing trifluoromethyl group in the 5a-position, which was achieved by treatment of ketone **7** with CF_3_TMS (Ruppert–Prakash reagent)^55^ and catalytic amounts of TBAF. Subsequent addition of a stoichiometric amount of TBAF yielded alcohol **24** in 58% with excellent diastereoselectivity. Assignment of the configuration at the carbinol center (C-5a) was carried out by HOESY NMR analysis of intermediate TMS ether **TMS-24**, which was isolated in small amounts (see the Supporting Information). Hydrogenation of the benzyl-protecting groups gave compound **25** in a yield of 43% (Scheme 3). Deoxygenation of compound **24** was performed as described for compound **18**. Formation of the corresponding methyl oxalic acid ester **26** succeeded in a yield of 78%. Subsequent treatment with HSnBu_3_ and AIBN proceeded with incomplete stereoselectivity to give carba-glucosamine **27** with an axial and **28** with an equatorial CF_3_ group in yields of 54% and 10%, respectively. The stereochemistry at position 5a of **27** and **28** was confirmed by NOESY and HOESY NMR spectra (Scheme 3 and the Supporting Information).

### Axial 5a-Hydroxy and -Alkoxy Substituents (Substitution Type III)

**Scheme 4:**
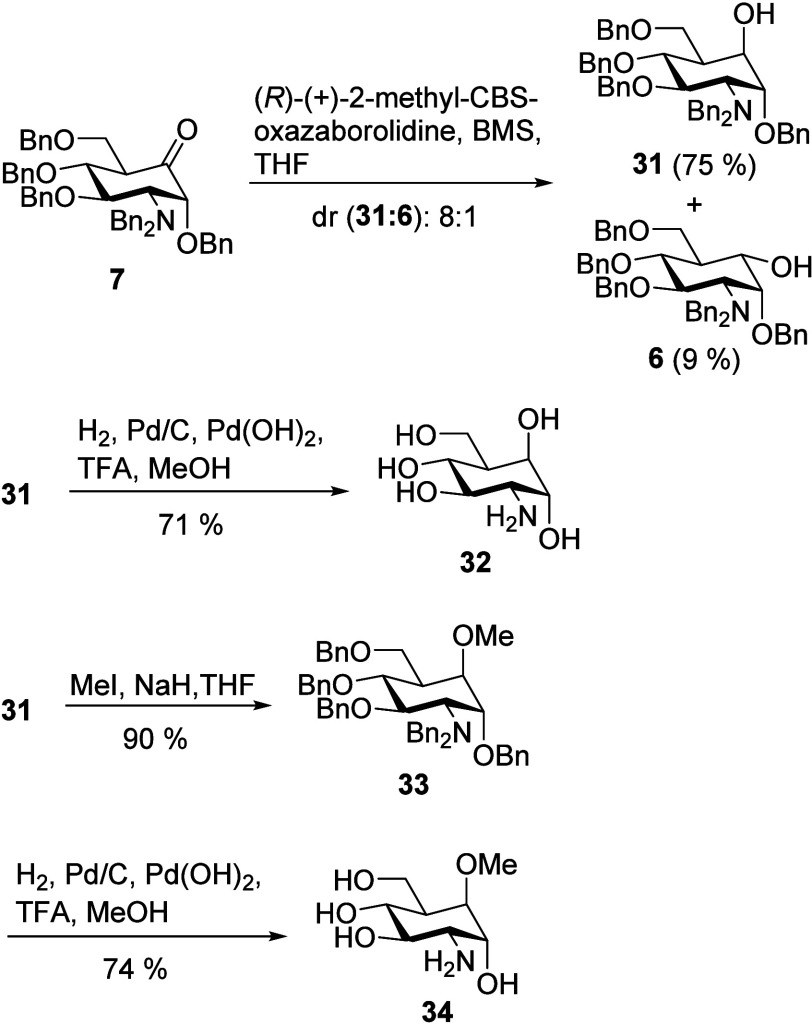
Preparation of Axial Alcohol **31** by CBS Reduction of Ketone **7** and Further Reaction to Carba-Sugars **33** and **34**

We previously reported the synthesis of carba-glucosamines with equatorial hydroxy (**3**) and alkoxy substituents (**4**, **5**).^42^ Compound **3** was obtained from ketone **7** by treatment with (*S*)-(−)-2-methyl-CBS oxazaborolidine as a catalyst and borane dimethyl sulfide as a reducing agent followed by hydrogenation.^42^ Keeping in mind the huge influence of the stereochemistry of a carbohydrate derivative on its biological function, we wanted to provide access to 5a-modified carba-glucosamines with an axial hydroxy group. To access this diastereomer of **6**, we reduced ketone **7** by applying (*R*)-(+)-2-methyl-CBS oxazaborolidine as a catalyst providing compound **31** in a yield of 75% beside small amounts of the diastereoisomer **6** with an equatorial hydroxy group (Scheme 4). Deprotection of **31** gave 5a-axially modified carba-glucosamine mimic **32**. To obtain methoxy derivative **33**, **31** was methylated with methyl iodide in a yield of 90%. Debenzylation of methyl ether **33** finally provided carba-glucosamine mimic **34** with an axial methoxy group.

### 5a-Fluoro-Substituted Carba-Glucosamine Mimics (Substitution Type IV)

**Scheme 5:**
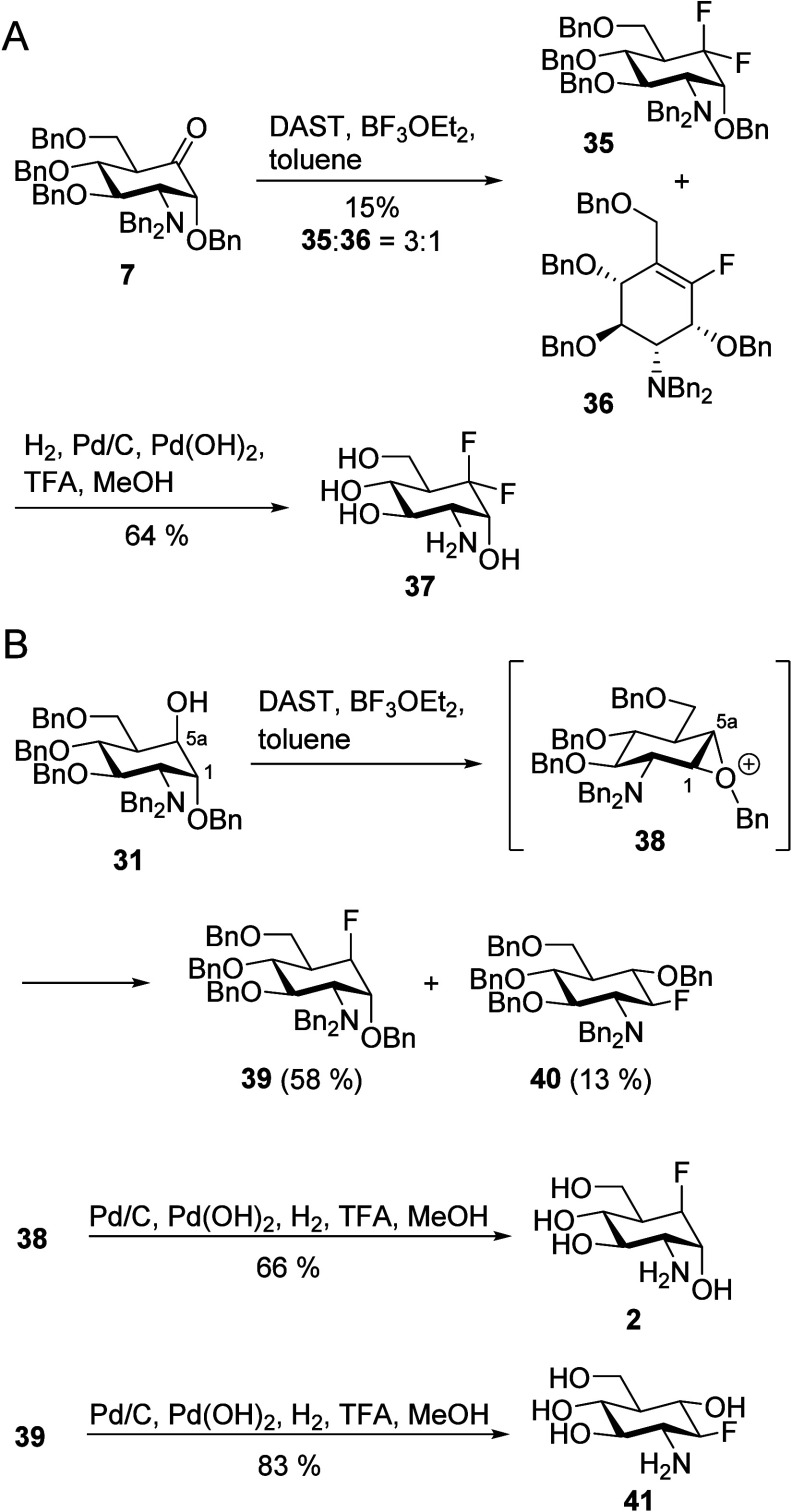
(A) Difluorination of Ketone **7** with DAST Resulted in a Mixture of **35** and **36**; (B) Deoxyfluorination of Axial Alcohol **31** Afforded a Mixture of **39** (Major Product) and Rearrangement Product **40** The mixture was hydrogenated upon which pure **37** could be separated. Hydrogenation of the separated compounds gave **2** and **41**, respectively.

Fluoro-modified carba-glucosamine **2** has been shown to have promising antimicrobial activity.^18^ Furthermore, Deleuze et al. reported the synthesis of *gem*-difluoro-carba-glucose via a triisobutylaluminum-promoted rearrangement^56^ and found that the difluoro substitution restores the anomeric effect.^57^ This implies that *gem*-difluoro-carba-glucosamine **37** might be a very promising mimic of glucosamine, and therefore, we attempted to extend the sugar mimic library toward this compound. Geminal difluorides can be generated from ketones by reaction with DAST.^58,59^ For ketones with a tertiary α position, however, this kind of conversion has been reported to be difficult, suffering from rearrangement and elimination side reactions.^59,60^ Indeed, when we treated ketone **7** with DAST, we first observed only traces of difluoride **35** by LC-MS and ^19^F NMR in a very complex product mixture. After extensive optimization of the conditions, 10 equiv of DAST and 1 equiv of boron trifluoride diethyl etherate in toluene in a PTFE reaction vessel gave 15% of an inseparable 3:1 mixture of difluoride **35** and the elimination product **36** (Scheme 5A). After hydrogenation, deprotected difluoride **37** was isolated in a pure form by HILIC-HPLC.

Previous approaches to the monofluorinated carba-glucosamine analogous to compound **2** but with the fluoride in equatorial position were not successful.^18^ Carba-sugar **2** was synthesized by a completely different approach relying on a Ferrier rearrangement and introduction of the fluoride via electrophilic fluorination in the α position to the resulting ketone.^18^ To provide access to the equatorial fluoride, we aimed for the conversion of the axial alcohol **31** to the fluoride in an S_N_2 fashion by deoxyfluorination. Surprisingly, this reaction yielded two compounds (Scheme 5B). The major product was identified as the axial fluoride **39**, which was obtained in a yield of 58% beside rearrangement product **40** (13%). The formation of these two compounds hints at the formation of oxonium ion intermediate **38**, which is formed by a neighboring group effect. Opening of **38** by fluoride results either in **39** under retention of configuration at the 5a position (^3^*J*_H-5,F_ = 37 Hz) via double inversion (major product according to the Fürst–Plattner rules) or in 1-fluoro compound **40** (^1^H NMR signal of H-1: 4.75 ppm, ddd, ^2^*J*_H,F_ = 51.4 Hz, ^3^*J*_H,H_ = 10.4, 8.7 Hz) under migration of the benzyl ether to the 5a-position. Both compounds were isolated and deprotected by hydrogenation to yield compounds **2** and **41**, respectively. Even though the synthesis of fluoro carba-sugar **2** starting from glucosamine hydrochloride has been published,^18^ the synthetic approach presented here starting from GlcNAc is much more efficient with an overall 25-fold improvement of the total yield.

### 5a-Amine-Substituted Carba-Glucosamine Mimics (Substitution Type V)

**Scheme 6:**
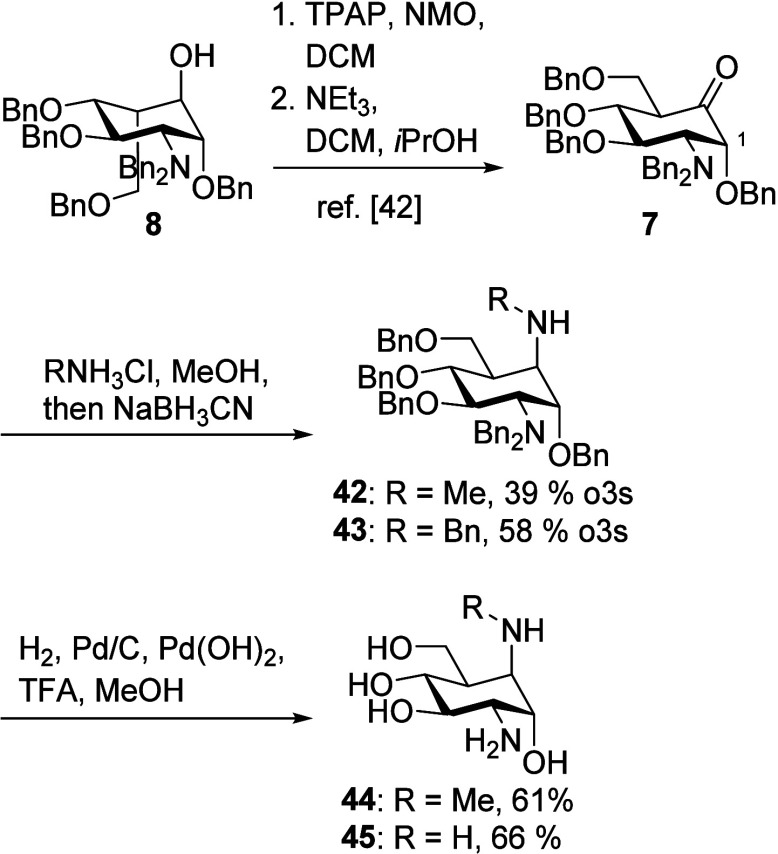
Synthesis of the 5a-Amino-Substituted Carba-Glucosamines **44** and **45**

The carba-glucosamine derivatives **1** and **2** have been shown to activate the self-cleavage reaction of the *glmS* riboswitch. The first step of this process is binding of the carba-glucosamine derivative to the riboswitch. Since RNA is a polyanionic molecule, multiple amino groups can be beneficial for binding to RNA. Indeed, prime examples of naturally occurring RNA ligands are aminoglycosides, which are carbohydrate-like structures with several amino groups.^61^ To potentially increase the RNA affinity of carba-glucosamine derivatives, we aimed at the introduction of an additional amino group in the 5a position. Ley–Griffith oxidation of **8** and subsequent isomerization following our previously published procedure^42^ gave ketone **7**, which was reacted as the crude product in a reductive amination using methyl or benzyl ammonium chloride and sodium cyanoborohydride as the reducing agent yielding diamines **42** and **43**, respectively (Scheme 6). NMR analysis revealed that the introduced amine was exclusively in the axial position, which is in line with our hypothesized coordination of cyanoborohydride to the benzyl ether in the 1-position. This precomplexation was expected to result in the introduction of the hydride from the equatorial position. The configurational assignment was based on the observed multiplicity of H-5a (pseudo-triplet with *J* = 3.2 Hz), indicating the absence of an axial–axial coupling and, therefore, its equatorial orientation. Compounds **42** and **43** were deprotected by catalytic hydrogenation with a mixture of Pd(0) on carbon and Pd(OH)_2_ on carbon, resulting in the amino-modified carba-glucosamine analogues **44** and **45** in yields of 61 and 66%, respectively.

### Bicyclic Carba-Glucosamine Mimics (Substitution Type VI)

**Scheme 7:**
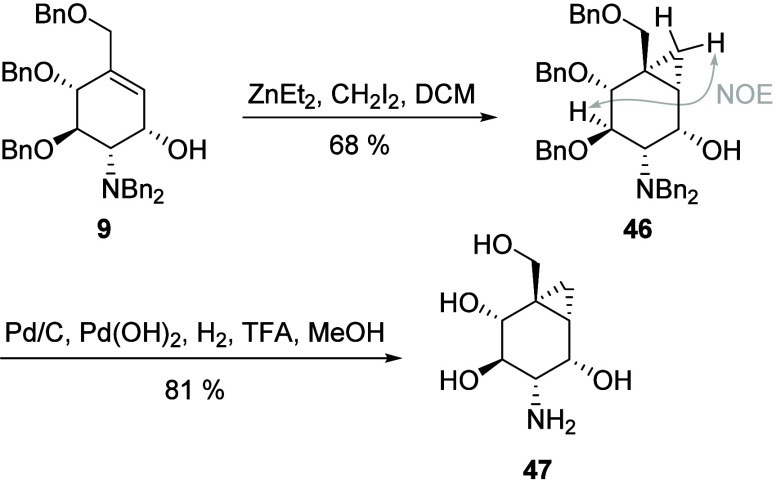
Synthesis of Bicyclic Carba-Glucosamine **47**

Apart from the above-mentioned intermediates of our previously developed carba-sugar synthesis, allyl alcohol **9** provided another point of modification. Allylic alcohol **9**, which was the product of a ring-closing metathesis reaction, proved to be ideal for a diastereoselective Simmons–Smith cyclopropanation^62^ that delivered bicyclic carba-glucosamine mimic **46** in a yield of 68% (Scheme 6). A cross peak between H-3 and one of the diastereotopic cyclopropyl protons in a NOESY NMR spectrum confirmed the stereochemistry depicted in Scheme 7. The cyclopropane ring was stable under the hydrogenation conditions needed for benzyl deprotection, and compound **47** was isolated in a yield of 81%.

### 5a-Carba-Disaccarides (Substitution Type VII)

**Scheme 8:**
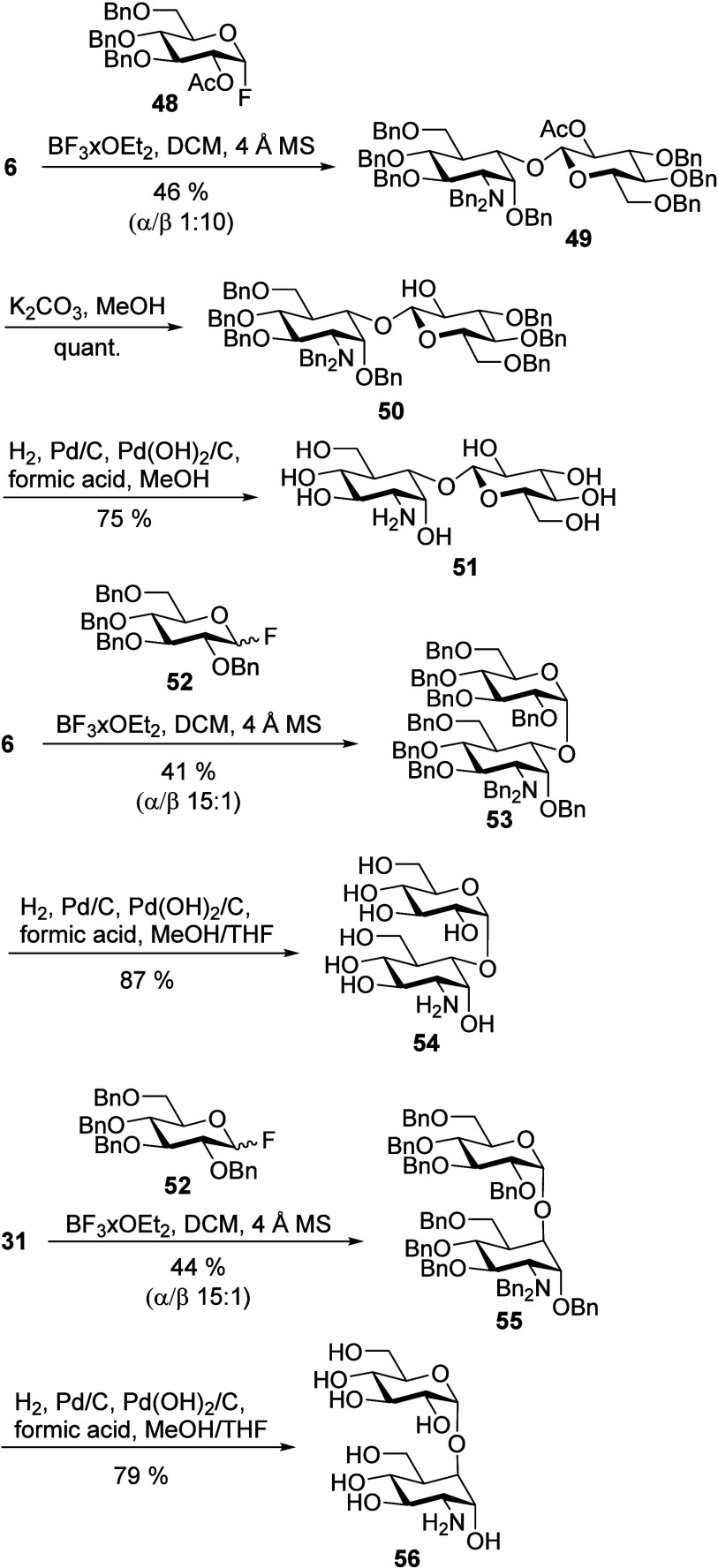
Preparation of Pseudo-Disaccharides **51**, **54**, and **56**

To fully show the scope of possible 5a-modifications, glycosylation of the 5a-hydroxy carba-glucosamine derivatives **6** and **31** to form pseudodisaccharides was attempted (Scheme 8). The obtained 1,5a-linked compounds represent interesting new structural motifs, not previously described in the literature. Glycosylation of the equatorial hydroxy group of **6** turned out to be very challenging. A series of glycosyl donors with varying anomeric leaving groups (acetate, activated by BF_3_ etherate or TMSOTf; bromide, activated by AgNO_3_, AgOTf, or Ag_2_CO_3_; phenyl thiolate, activated by *N*-iodosuccinimide; and trichloroacetimidate, activated by BF_3_ etherate) gave only trace amounts of product. However, reaction of alcohol **6** with 2-*O*-acetyl-protected glucosyl fluoride **48**(63) and BF_3_ etherate as an activator gave β-1,5a-pseudodisaccharide **49** in a yield of 46%. The neighboring group effect of the acetyl-protecting group ensured a high β-selectivity of α/β 1:10 of the glycosylation reaction. The configuration at the anomeric center of the glucose moiety was confirmed by the H1′–H2′ coupling constant (^3^*J* = 7.9 Hz). Deacetylation with potassium carbonate in MeOH gave compound **50** in a quantitative yield. Subsequent hydrogenation removed the benzyl-protecting groups to obtain unprotected β-1,5a-pseudodisaccharide **51** in a yield of 75%. When the equatorial 5a alcohol **6** was reacted with perbenzylated glycosyl fluoride **52**,^64^ α-product **53** (^3^*J*_H1′,H2__′_ = 3.5 Hz) was obtained with high selectivity (α/β 15:1) in a yield of 41%. Compound **53** was debenzylated to give α-1,5a-pseudodisaccharide **54.** Reaction of the axial 5a alcohol **31** with fluoride **52**(64) gave α-product **55** (^3^*J*_H1′,H2__′_ = 3.6 Hz) in a yield of 44%. **55** could be successfully deprotected by hydrogenation to provide α-1,5a-pseudodisaccharide **56**. These experiments demonstrate the high degree of stereochemical control that can be achieved at the obtained 1,5a-glycosidic bond.

### Antibacterial Assays

**Figure 2:**
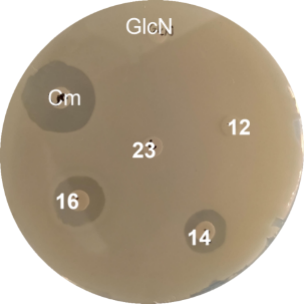
Filter disk assay on Luria–Bertani (LB) agar plates. *B. subtilis* wt 168 was plated out and tested with 10 μL of a 100 mM solution of the respective compound on a filter disk. *Cm*: chloramphenicol at a concentration of 9.3 mM.

All synthesized carba-glucosamine mimics were tested for their antimicrobial properties in filter disk assays. Glucosamine (GlcN) was used as the negative control. This carbohydrate is a substrate of phosphotransferase transporter systems, which couple active transport over the bacterial membrane with phosphorylation of the 6-hydroxy group, thereby producing natural, nontoxic GlcN6P. The known antibiotic chloramphenicol (*Cm*) served as a positive control. For the monofluoro-substituted carba-glucosamine **2**, we observed growth inhibition of *B. subtilis*, which is in line with the previously published results. In addition, we observed clear inhibition zones for the 5a-aryloxy-substituted carba-glucosamines **14** and **16**, whereas phenyloxy-substituted carba-glucosamine **12** did not show any growth inhibition (Figure 2). All other compounds did not show an inhibitory effect, as well.

## Conclusions

In this publication, we report the synthesis of a diverse library of new unnatural carba-glucosamine mimics with various substituents in the 5a-position. Starting from late-stage intermediates of a previously published synthesis of carba-glucosamines, it was possible to access aryloxy-, alkyloxy-, alkyl-, amine-, and fluoro-substituted as well as disubstituted carba-sugars, pseudodisaccharides, and a bicyclic derivative. The reported synthesis is flexible, giving access to a large variety of 5a-modified carba-α-d-glucosamines in reasonable yields and with a controllable stereochemical outcome. Given the fact that many naturally occurring carba-sugars have interesting biological activities, we tested these new members of this still “juvenile” compound class of substituted carba-sugars for their antimicrobial potential. The aryl-modified compounds **14** and **16** showed growth inhibition of *B. subtilis* and, therefore, can be considered as a starting point for the development of new antibiotics. We are convinced that the synthesis of this library is not only of interest for antimicrobial research but also will spark the attention of colleagues working on other applications of carbohydrate mimics, such as antidiabetic compounds or glycosyltransferase inhibitors.

## Data Availability

The data underlying this study are available in the published article and its Supporting Information.

## Supporting Information Available

General experimental methods; syntheses; filter disk assay; and NMR spectra (PDF)

## Notes

The initial version of this manuscript has been uploaded to a preprint repository.^65^
