# Supplementary material for: Diverse Library of 5a-Substituted Carba-Glucosamines
Source: J Org Chem. 2025 Feb 15;90(8):2969–77. doi: 10.1021/acs.joc.4c02816 (PMC11877516; doi:10.1021/acs.joc.4c02816)
Supplement: Supplementary file 1 — jo4c02816_si_001.pdf [file jo4c02816_si_001.pdf]

Supporting Information to

A Diverse Library of 5a-Substituted Carba-Glucosamines

Bjarne Silkenath,[1] Dennis Kläge,[1] Philip Eppelin,[1] Jörg S. Hartig,[1] Valentin Wittmann*,[1]

[1] *Department of Chemistry, University of Konstanz, 78457 Konstanz, Germany*

* Corresponding author:

V. Wittmann, Email: mail@valentin-wittmann.de

Content

1 General Methods S2

2 Syntheses S3

3 Filter Disk Assay S36

4 NMR Spectra S37

5 References S104

1 General Methods

Chemicals were purchased from *Acros Organics*, *Sigma-Aldrich, TCI Chemicals Europe*, *abcr* or *Carbosynth* and used without further purification. Technical solvents were distilled prior to use. If necessary, reactions were carried out under nitrogen atmosphere using the Schlenk technique. Solvents were dried by common methods or bought from *Sigma-Aldrich* or *Acros Organics*. High-resolution mass spectra were recorded on a *micrOTOF II ESI* mass spectrometer from *Bruker* or an *LTQ Orbitrap Velos* mass spectrometer from *Thermo Scientific*. Analysis of data and calculation of the expected mass was performed with *Compass DataAnalysis 4.0* from *Bruker*. Samples were dissolved in water, acetonitrile or mixtures thereof. Preparative high-performance liquid chromatography (HPLC) was performed on a LC-20A device from *Shimadzu* containing the following components. Degasser: DGU-20A3, auto sampler: SIL-20A, pumps: LC-20AT, column oven: CTO-20AC, controller: CMB-20A, photodiode array detector: SPD-M20A, columns for separation and eluents are mentioned in the synthesis procedures. Data analysis was performed with LCsolution v. 1.25 from *Shimadzu*. Preparative flash column chromatography (FC) was carried out on silica gel 60 (Geduran Si 60; 0.040-0.063 mm particle size) from *Merck*. Data regarding solvent mixtures is given as volume ratio (v/v). Technical solvents were distilled prior to use for FC. NMR spectra were recorded on Avance III 400 or Avance III 600 or Avance Neo 800 spectrometers from *Bruker* or Lambda 400 or Lambda 500 spectrometers from JEOL. The measurements were performed at room temperature. Structural assignments were made with additional information from COSY, NOESY, HSQC, ^{1}H-^{13}C-HMBC, and ^{1}H-^{19}F-HOESY experiments. As an analysis program *MestReNova* v12.0 from *Mestrelab Resarch S.L.* was used. Pseudomultiplets are marked with a "p". For the visualization of reaction progresses and to characterize products using R_f values, analytical thin-layer chromatography (TLC) was performed using silica-coated aluminum sheets (TLC Silica gel 60 F) from *Merck*. Detection was carried out either by excitation of the fluorescence at 254 nm or by dipping in one of the following staining solutions and subsequent gentle heating.

- Anisaldehyde reagent: ethanol (135 mL), conc. H_2SO_4 (5 mL), 4-anisaldehyde (3.7 mL), glacial acetic acid (1.5 mL).
- Vanillin reagent: ethanol (250 mL), conc. H_2SO_4(2.5 mL), vanillin (6 g)
- Potassium permanganate reagent: 0.1 % $KMnO_4$ in 1 N NaOH.

2 Syntheses

(1*R*,2*R*,3*R*,4*R*,5*R*,6*R*)-3-Amino-5-fluoro-6-(hydroxymethyl)cyclohexane-1,2,4-triol (2)

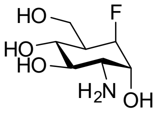

To a stirred solution of **38** (31.5 mg, 42.8 µmol) in dry MeOH (2.5 mL) was added Pd/C (10% waterwet, 30.0 mg), $Pd(OH)_2$-C (20 % waterwet, 30.0 mg) and TFA (25.0 µL). The reaction was purged with hydrogen. The reaction was stirred for 12 h under hydrogen atmosphere. The reaction mixture was centrifuged. The supernatant was purified by HILIC-HPLC to give compound **2** (5.6 mg, 28.3 µmol, 66 %) as a white powder after lyophilization. The analytical data were in accordance with the literature.[1] In literature the acetic acid salt is reported. Here we report the free amine. ^{1}H NMR (D_2O, 500 MHz): δ 5.01 – 4.86 (ddd, 1H, *J* = 45.5, 4.0, 2.0 Hz, H-5a), 4.22 (m, 1H, H-1), 3.99 (dd, *J* = 11.2, 4.4 Hz, 1x H-6, 1H), 3.80 (ddd, 1H, *J* = 11.2, 9.4, 1.4 Hz, 1xH-6), 3.62 – 3.44 (m, 2H, H-3, H-4), 3.02 (m, 1H, H-2), 2.18 – 1.97 (m, 1H, H-5); ^{13}C{^{1}H} NMR (D_2O, 126 MHz,): δ 90.1 (d, *J* = 172.6 Hz, H-5a), 73.7, (C-3), 70.1(C-4), 68.1 (C-1), 58.6 (C-6), 52.4(C-2), 42.7(d, *J* = 18.1 Hz, C-5); ^{19}F NMR (376 MHz, D_2O) δ [ppm] = -201.32 (dd, *J* = 45.5, 38.7 Hz); HRMS (ESI) *m/z*: [*M*+H]$^+$ Calcd for $C_7H_{15}FNO_4$ 196.0980; Found 196.0977; HPLC (Phenomenex® Luna 5µ HILIC 200Å, AXIA Pa, 250 x 21.20 mm, MeCN/12 mM TEAB buffer pH 7.00 = 30/70, flow rate = 8.0 mL/min, ELSD): t_R = 13.2 min.

(1*R*,2*R*,3*R*,4*R*,5*S*,6*R*)-*N*,*N*-Dibenzyl-2,3,6-tris(benzyloxy)-4-((benzyloxy)methyl)-5-((4'-methyl-[1,1'-biphenyl]-4-yl)oxy)cyclohexan-1-amine (10)

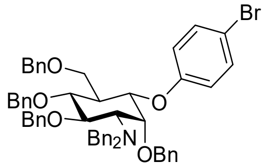

Alcohol **6**[2] (200.0 mg, 275.5 µmol) was dissolved in dry DMF (1 mL). NaH (60 wt%, 14.2 mg, 354.35 µmol) and 4-bromofluorobenzene (33.0 µL, 52.5 mg) were added and the mixture was stirred at 50 °C (oil bath). After 90 min another equivalent of NaH (10.9 mg) and of the 4-bromofluorobenzene (29.99 µL) was added. The reaction was stopped by

addition of saturated aq. NH_4Cl solution (10 mL) and extracted with DCM (3x10 mL). The organic layer was dried over $MgSO_4$, and the solvents were evaporated under reduced pressure. The crude product was purified by FC (petroleum ether/EtOAc = 10:1), and product **10** was isolated as a yellow oil (218.2 mg, 0.25 mmol, 90%). ^{1}H NMR ($CDCl_3$, 500 MHz): δ 7.48 – 7.11 (m, 32H, H-arenes), 7.09 – 7.01 (m, 2H, H-arenes), 6.67 – 6.63 (m, 2H, H-arenes), 5.07 (d, 1H, *J* = 11.6 Hz, O-CHHPh), 4.89 (d, 1H, *J* = 11.6 Hz, O-CHHPh), 4.80 (m, 2H, 2x O-CHHPh), 4.58 (d, 1H, *J* = 10.7 Hz, O-CHHPh), 4.53 (d, 1H, *J* = 10.5 Hz, O-CHHPh), 4.36 (d, 1H, *J* = 12.1 Hz, O-CHHPh), 4.31 – 4.20 (m, 3H, O-CHHPh, H-1, H-3), 4.17 (dd, 1H, *J* = 11.1, 1.9 Hz, H-5a), 4.02 (m, 4H, 2x N-CHHPh), 3.79 (dd, 1H, *J* = 9.2, 2.0 Hz, 1x H-6), 3.70 (dd, 1H, *J* = 11.1, 8.7 Hz, H-4), 3.63 (dd, 1H, *J* = 9.2, 2.3 Hz, 1x H-6), 2.76 (d, 1H, *J* =11.1, 2.0 Hz, H-2), 2.52 (ptpt, 1H, *J* = 11.1, 2.2 Hz, H-5); ^{13}C{^{1}H} NMR ($CDCl_3$, 126 MHz): δ 157.4, 141.1, 139.8, 138.9, 138.3, 132.9, 128.8, 128.7, 128.9, 128.6, 128.2, 128.2, 128.0, 127.9, 127.6, 127.3, 127.1, 117.3, 113.4 (C_{arenes}), 81.1 (C-4), 80.9 (C-3), 79.1 (C-1), 75.7 (O-CH_2Ph), 75.6 (C-5a), 75.1 (O-CH_2Ph), 73.5 (O-CH_2Ph), 64.7 (C-6), 59.0 (C-2), 56.4 (2x N-CH_2Ph), 43.3 (C-5); HRMS (ESI) *m*/*z*: [*M*+H]$^+$ Calcd for $C_{55}H_{55}NBrO_5$ 888.3258; Found 888.3251.

(1*R*,2*R*,3*R*,4*R*,5*S*,6*R*)-*N*,*N*-Dibenzyl-2,3,6-tris(benzyloxy)-4-((benzyloxy)methyl)-5-((4'-methyl-[1,1'-biphenyl]-4-yl)oxy)cyclohexan-1-amine (11)

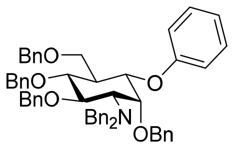

Compound **10** (77.0 mg, 86.6 μmol) was dissolved in dry THF (2 mL) and cooled to -78 °C. *t*-BuLi (1.7 M in hexanes, 203.8 μL, 346.5 μmol) was added, and the mixture was stirred for 5 minutes. Water (2 mL) was added, and the aqueous phase was extracted with DCM (3x5 mL). The combined organic phases were evaporated under reduced pressure. The crude product was purified by HPLC, and **11** was isolated as an oil (32.9 mg, 40.62 μmol, 49%). ^{1}H NMR ($CDCl_3$, 400 MHz): δ 7.53 – 6.97 (m, 33H, H_{arenes}), 6.82 (d, 2H, *J* = 8.2 Hz, H-arenes), 5.08 (d, 1H, *J* = 11.6 Hz, O-CHHPh), 4.94 – 4.85 (m, 2H 2x O-CHHPh), 4.82 (d, 1H, *J* = 10.7 Hz, O-CHHPh), 4.61 (d, 1H, *J* = 10.7 Hz, O-CHHPh), 4.54 (d, 1H, *J* = 10.5 Hz, O-CHHPh), 4.40 – 4.23 (m, 5H, 2x O-CHHPh, H-5a, H-3, H-1), 4.11 -3.94 (m, 4H, 2x N-CHHPh), 3.82 (dd, 1H, *J* = 9.2, 2.0 Hz, 1x H-6), 3.78 – 3.64 (m, 2H, H-4, 1x H-6), 2.80 (dd, 1H, *J* = 11.0, 1.9 Hz, H-2), 2.56 (m, H-5); ^{13}C{^{1}H} NMR ($CDCl_3$, 126 MHz): δ 158.0, 140.9, 139.7, 138.8,

138.7, 138.2 ($C_{quart. arenes}$), 129.9, 128.5, 128.4, 128.4, 128.4, 128.3, 128.0, 127.9, 127.7, 127.6, 127.5, 127.3, 127.1, 126.8, 121.0, 115.3 (C_{arenes}), 80.8 (C-4), 80.7 (C-3), 78.7 (C-1), 75.4 (O-<u>C</u>H_2Ph), 74.7 (C-5a), 73.2 (O-<u>C</u>H_2Ph), 73.1 (O-<u>C</u>H_2Ph), 64.6 (C-6), 58.8 (C-2), 56.1 (2x N-<u>C</u>H_2Ph), 43.1 (C-5), 27.1 (CH_3); HRMS (ESI) *m/z*: $[M+H]^+$ Calcd for $C_{55}H_{56}NO_5$ 810.4153; Found 810.4144; HPLC (Kinetex® 5 µm C8 100Å, 250 x 21.2 mm, MeCN, flow rate= 30 mL/min, λ = 254 nm): t_R = 3.3 min.

(1*R*,2*R*,3*R*,4*R*,5*S*,6*R*)-3-Amino-6-(hydroxymethyl)-5-phenoxycyclohexane-1,2,4-triol (12)

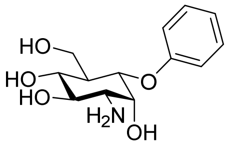

Compound **10** (33.0 mg, 40.7 µmol) was dissolved in methanol p.a. (5 mL). $Pd(OH)_2$/C and Pd/C (each 35 wt%) and TFA (31.2 µL, 407.4 µmol) were added to the mixture. Hydrogen was added to the reaction continuously via a balloon. The reaction was monitored by LC-MS. The catalyst was filtrated off by using a syringe filter, which was washed with 20 mL of methanol. The solvent was evaporated under reduced pressure. The crude product was purified using HPLC and was isolated as a colorless solid (9.4 mg, 31.4 µmol, 76 %) as the formic acid salt of compound **12**. ^{1}H NMR (D_2O, 500 MHz): δ 7.47 – 7.35 (m, 2H, H-ortho), 7.21 – 7.08 (m, 3H, H-meta, H-para), 4.53 (dd, 1H, *J* = 11.3, 2.7 Hz, H-5a), 4.46 (pt, 1H, *J* = 2.7 Hz, H-1), 4.02 (dd, 1H, *J* = 11.6, 2.3 Hz, 1x H-6), 3.89 (dd, 1H, *J* = 11.6, 2.6 Hz, 1x H-6), 3.85 (dd, 1H, *J* = 10.8, 9.2 Hz, H-3), 3.59 (dd, 1H, *J* = 11.0, 9.2 Hz, H-4), 3.30 (dd, 1H, *J* = 10.8, 2.7 Hz, H-2), 2.15 (m, 1H, H-5); $^{13}C\{^1H\}$ NMR (D_2O, 126 MHz): δ 159.4 (O-C phenyl), 132.7 (C-ortho), 125.1 (C-para), 119.2 (C-meta), 76.2 (C-5a), 73.5 (C-3), 72.0 (C-4), 68.2 (C-1), 58.7 (C-6), 56.1 (C-2), 45.6 (C-5); HRMS (ESI) *m/z*: $[M+H]^+$ Calcd for $C_{13}H_{20}NO_5$ 270.1336; Found 270.1334; HPLC (Kinetex® 5 µm C18 100Å, 250 x 21.2 mm, water 0.1 % FA/MeCN 0.1 % FA, 90/10 to 0/100 in 8 min then 0/100, flow rate=8 mL/min, λ = 254 nm): t_R = 8.7 min.

(*1R*,2*R*,3*R*,4*R*,5*S*,6*R*)-*N*,*N*-Dibenzyl-2,3,6-tris(benzyloxy)-4-((benzyloxy)methyl)-5-(4-(naphthalen-1-yl)phenoxy)cyclohexan-1-amine (13)

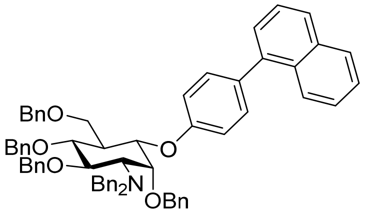

Compound **10** (15.0 mg, 16.9 µmol) was dissolved in degassed THF/water (2:1, 3 mL) in a pressure tube. 1-Naphthylboronic acid (8.7 mg, 50.6 µmol), $Pd(PPh_3)_4$ (1.95 mg, 1.7 µmol), and K_2CO_3 (35.0 mg, 253.1 µmol) were added, the tube was sealed, and the mixture was stirred for two hours at 80 °C (oil bath). Water (5 mL) was added and the mixture extracted with DCM (3x5 mL). The organic layer was dried over $MgSO_4$ and the solvent was evaporated under reduced pressure. The crude product was purified by FC on silica gel (petroleum ether/EtOAc = 100:1), and the product **13** was isolated as a yellow oil (13.0 mg, 13.9 µmol, 82 %). ^{1}H NMR ($CDCl_3$, 400 MHz): δ 8.01 (d, 1H *J* = 8.2 Hz, H-arenes), 7.94 (dd, 1H, *J* = 8.0, 1.5 Hz), 7.88 (d, 1H, *J* = 8.2 Hz, H-arenes), 7.61 – 7.09 (m, 36 H, H-arenes), 6.94 – 6.88 (m, 2H, H-arenes) , 5.09 (d, 1H, *J* = 11.6 Hz, O-CHHPh), 4.96 (d, 1H, *J* = 10.5 Hz, O-CHHPh), 4.91 (d, 1H, *J* = 11.6 Hz, O-CHHPh), 4.83 (d, 1H, *J* = 10.5 Hz, O-CHHPh), 4.63 (m, 2H, 2x O-CHHPh), 4.47 – 4.39 (m, 2H, H-1, O-CHHPh), 4.37 – 4.27 (m, 3H, H-3, H-5a, O-CHHPh), 4.05 (s, 4H, 2x N-CHHPh), 3.85 (dd, 1H, *J* = 9.2, 2.1 Hz, 1x H-6), 3.81 – 3.70 (m, 2H, 1x H-6)), 2.83 (dd, 1H, *J* = 11.0, 2.0 Hz, H-2), 2.60 (m, 1H, H-5); ^{13}C{^{1}H} NMR ($CDCl_3$, 101 MHz): δ 157.5, 140.9, 140.0, 139.6, 138.8, 138.7, 138.3, 134.1, 133.5, 132.0, 131.5, 128.6, 128.5, 128.5, 128.4, 128.3, 128.1, 128.0, 128.0, 127.8, 127.7, 127.6, 127.3, 127.1, 126.8, 126.2, 125.9, 125.6, 115.1 (C_{arenes}), 80.9 (C-3), 80.8 (C-4), 78.9 (C-1), 75.4 (C-5a), 75.2 (O-CH_2Ph), 74.9 (O-CH_2Ph), 73.2(O-CH_2Ph), 73.2 (O-CH_2Ph), 64.7 (C-6), 58.9 (C-2), 56.2 (2x N-CH_2Ph), 43.2 (C-5); HRMS (ESI) *m*/*z*: [*M*+H]$^+$ Calcd for $C_{65}H_{62}NO_5$ 936.4623; Found 936.4611.

(1*R*,2*R*,3*R*,4*R*,5*S*,6*R*)-3-Amino-6-(hydroxymethyl)-5-(4-(5,6,7,8-tetrahydronaphthalen-1-yl)phenoxy)cyclohexane-1,2,4-triol (14)

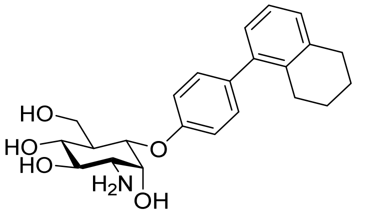

Compound **13** (23 mg, 24.6 μmol) was dissolved in methanol (5 mL). $Pd(OH)_2$/C, Pd/C (each 35 wt%), and TFA (18.9 μL, 245.8 μmol) were added to the mixture. Hydrogen was added to the reaction continuously via a balloon. The reaction was monitored by LC-MS. The catalyst was filtrated off by using a syringe filter, which was washed with 20 mL of methanol. The solvent was evaporated under reduced pressure and the crude product was purified using HPLC, and was isolated as a colorless solid (7.1 mg, 16.5 μmol, 66 %) as the formic acid salt of compound **14**. ^{1}H NMR (D_2O, 400 MHz): δ 8.46 (s, 1H, FA), 7.37 – 7.00 (m, 7H, arenes), 4.57 (d, 1H *J* = 11.2 Hz, H-5a), 4.51 (m, 1H, H-1), 4.03 (m, 1H, 1x H-6), 3.94 – 3.87 (m, 1H, 1x H-6), 3.88 – 3.78 (m, 1H, H-3), 3.68 – 3.52 (m, 1H, H-4), 3.36 – 3.22 (m, 1H, H-2), 2.86 (t, 2H, *J* = 6.2 Hz, 1x CH_2), 2.59 (t, 2H, *J* = 6.2 Hz, 1x CH_2), 2.17 (m,1H, H-5), 1.81 – 1.63 (m, 4H, 2x CH_2); $^{13}C\{^1H\}$ NMR (D_2O, 151 MHz): δ 170.8 (FA), 155.5, 154.7, 141.1, 138.0, 135.2, 134.9, 130.3, 129.9, 128.9, 128.5, 126.8, 125.5, 115.7 (C_{arenes}), 73.1 (C-5a), 70.7 (C-3), 68.9 (C-4), 65.3 (C-2), 55.5 (C-6), 53.1 (C-2), 42.7 (C-5), 29.0 (CH_2), 27.5 (CH_2), 22.6 (CH_2), 22.1 (CH_2); HRMS (ESI) *m*/*z*: [*M*+H]$^+$ Calcd for $C_{23}H_{30}NO_5$ 400.2118; Found 400.2114. HPLC (Kinetex® 5 μm C18 100Å, 250 x 21.2 mm, water 0.1 % FA/MeCN 0.1 % FA, 90/10 to 0/100 in 8 min then 0/100, flow rate=8 mL/min, λ = 254 nm): t_R = 9.7 min.

(1*R*,2*R*,3*R*,4*R*,5*S*,6*R*)-*N*,*N*-Dibenzyl-2,3,6-tris(benzyloxy)-4-((benzyloxy)methyl)-5-((4'-methyl-[1,1'-biphenyl]-4-yl)oxy)cyclohexan-1-amine (15)

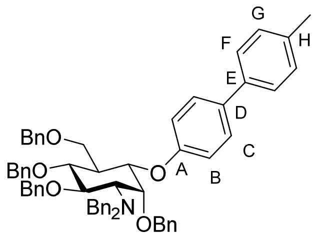

Compound **10** (50.0 mg, 56.3 µmol) was dissolved in degassed THF/water (2:1, 3 mL) in a pressure tube. *p*-Tolylboronic acid (30.6 mg, 225.0 µmol), $Pd(PPh_3)_4$ (6.5 mg, 5.6 µmol), and K_2CO_3 (116.6 mg, 843.7 µmol) were added, the tube was sealed, and the mixture was stirred for two hours at 80 °C (oil bath). Water (5 mL) was added, and the mixture extracted with DCM (3x5 mL). The combined organic layers were dried over $MgSO_4$ and the solvent was evaporated under reduced pressure. The crude product was purified by FC on silica gel (petroleum ether/EtOAc = 100:1), and product **15** was isolated as a yellow oil (36.9 mg, 41.0 µmol, 73 %). ^{1}H NMR ($CDCl_3$, 500 MHz): δ 7.58 – 7.49 (m, 4H, H-arenes), 7.49 – 7.38 (m, 8H, H-arenes), 7.36 – 7.06 (m, 24H, H-arenes), 6.86 (d, *J* = 8.6 Hz, 2H, H-arenes), 5.08 (d, 1H, *J* = 11.6 Hz, O-C<u>H</u>HPh), 4.91 (m, 2H, 2x O-C<u>H</u>HPh), 4.83 (d, 1H, *J* = 10.6 Hz, OC<u>H</u>HPh), 4.64 – 4.55 (m, 2H, 2x O-C<u>H</u>HPh), 4.41 – 4.35 (m, 2H, H-1, 1x O-C<u>H</u>HPh), 4.30 (m, 3H, H-5a, H-3, 1x O-C<u>HH</u>Ph), 4.11 – 3.98 (m, 4H, 2x N-C<u>HH</u>Ph), 3.83 (dd, 1H *J* = 9.3, 2.0 Hz, 1x H-6), 3.78 – 3.69 (m, 2H, 1x H-6, H-4), 2.82 (dd, 1H, *J* = 11.0, 2.0 Hz, H-2), 2.57 (pt, 1H, *J* = 11.0 Hz, H-5), 2.43 (s, 3H, CH_3); $^{13}C\{^1H\}$ NMR ($CDCl_3$, 126 MHz): δ 157.4, 140.9, 139.6, 138.8, 138.7, 138.2, 138.0, 136.7, 134.1 ($C_{quart.\ arenes}$), 129.7, 128.6, 128.4, 128.4, 128.4, 128.3, 128.0, 128.0, 128.0, 127.7, 127.7, 127.5, 127.3, 127.1, 126.8, 126.75, 115.5 (C_{arenes}), 80.8 (C-3), 80.7 (C-4), 78.7 (C-1), 75.4 (O-<u>C</u>H_2Ph), 75.0 (O-<u>C</u>H_2Ph), 74.8 (C-5a), 73.2 (O-<u>C</u>H_2Ph), 73.2 (O-<u>C</u>H_2Ph), 64.6 (C-6), 58.8 (C-2), 56.1 (2x N-<u>C</u>H_2Ph), 43.1 (C-5), 21.2 (CH_3);); HRMS (ESI) *m*/*z*: $[M+H]^+$ Calcd for $C_{62}H_{62}NO_5$ 900.4623; found 900.4611.

(1*R*,2*R*,3*R*,4*R*,5*S*,6*R*)-3-Amino-6-(hydroxymethyl)-5-((4'-methyl-[1,1'-biphenyl]-4-yl)oxy)cyclohexane-1,2,4-triol (16)

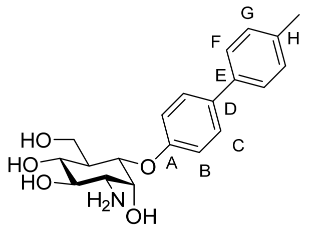

Compound **15** (24.0 mg, 26.7 µmol) was dissolved in methanol (5 mL). $Pd(OH)_2$ and Pd/C (each 35 wt%) and TFA (20.4 µL, 266.6 µmol) were added to the mixture. Hydrogen was added to the reaction continuously via a balloon. The reaction was monitored by LC-MS. The catalyst was filtrated off by using a syringe filter, which was washed with 20 mL of methanol. The solvent was evaporated under reduced pressure. The crude product was purified by HPLC and compound **16** was isolated as a colorless solid (6.0 mg, 17.7 µmol, 63%) as the formic acid salt. ^{1}H NMR (D_2O, 800 MHz): δ 8.32 (s, 1H, FA), 7.54 (d, *J* = 8.2 Hz, 2H, H-C), 7.45 (d, *J* = 7.7 Hz, 2H, H-F), 7.21 (dd, *J* = 7.7 Hz, 2H, H-G), 7.06 (d, *J* = 8.2 Hz, 2H, H-B), 4.43 – 4.37 (m, 1H, H-5a), 4.32 (pt, 1H, *J* = 2.8 Hz, H-1), 3.89 (d, 1H, *J* = 11.5 Hz, 1x H-6), 3.78 – 3.71 (m, 1H, 1x H-6), 3.67 (pt, 1H *J* = 10.0 Hz, H-3), 3.45 (pt, 1H *J* = 10.1 Hz, H-4), 3.13 – 3.07 (m, 1H, H-2), 2.25 (s, 3H, CH_3), 2.03 (m, 1H, H-5); $^{13}C\{^{1}H\}$ NMR (D_2O, 201 MHz): δ 171.0 (C-FA), 156.3 (C-A), 137.7 (C-H), 136.9 (C-E), 134.5 (C-D), 129.7 (C-G), 128.1 (C-F), 126.5 (C-C), 117.0 (C-B), 73.7 (C-5a), 71.4 (C-3), 69.4 (C-4), 65.9 (C-1), 56.2 (C-6), 53.4 (C-2), 43.1 (C-5), 20.0 (CH_3); HRMS (ESI) *m/z*: [*M*+H]$^+$ Calcd for $C_{20}H_{26}NO_5$ 360.1805; Found 360.1804; HPLC (Kinetex® 5 µm C18 100Å, 250 x 21.2 mm, water 0.1 % FA/MeCN 0.1 % FA, 90/10 to 0/100 in 8 min then 0/100, flow rate=8 mL/min, λ = 254 nm): t_R = 9.6 min.

(1*S*,2*R*,3*R*,4*R*,5*R*,6*R*)-2,4,5-Tris(benzyloxy)-6-((benzyloxy)methyl)-3-(dibenzylamino)-1-methylcyclohexan-1-ol (17)

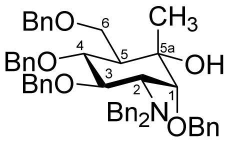

To a stirred solution of **7**[2] (280.0 mg, 382.6 µmol) in dry THF (5 mL) at 0 °C was added methyl magnesium chloride (30.04 mg, 401.7 µmol, 133.9 µL, 3 M in THF). The solution was stirred for 30 min. The reaction was stopped by adding sat. NH_4Cl_{aq} (3 mL) and water

(2 mL). The aqueous layer was extracted with CH_2Cl_2 (3 x 3 mL). The combined organic layers were dried over $MgSO_4$ and concentrated under reduced pressure. The residue was purified by FC (petroleum ether / EtOAc = 12:1 – 8:1) to give compound **17** (160.0 mg, 213.9 µmol, 56 %) as a colourless oil. Compound **18** (50 mg, 68.7 µmol**,** 18 %) was isolated as a minor product. R_f: 0.73 (petroleum ether : EtOAc = 5:1); ^1H NMR (400 MHz, $CDCl_3$): δ 7.55 - 7.20 (m, 30H, arenes), 5.09 (d, 2H, *J* = 11.3 Hz, O-C$\underline{H_2}$Ph), 4.97 (d, 1H, *J* = 11.6 Hz, O-C$\underline{H}$HPh), 4.89 – 4.79 (m, 2H, O-C$\underline{H_2}$Ph), 4.60 – 4.54 (m, 2H, O-C$\underline{H_2}$Ph), 4.48 (d, 1H, *J* = 11.6 Hz, O-C$\underline{H}$HPh), 4.34 (dd, 1H, *J* = 11.2 Hz, 8.6 Hz, H-3), 4.14 - 4.03 (m, 4H, 2 x N- C$\underline{H_2}$Ph), 3.95 (dd, 1H, *J* = 9.3 Hz, 3.0 Hz, H-6a), 3.75 - 3.69 (m, 2H, H-6b, H-1), 3.47 - 3.39 (m, 2H, H-4, O-$\underline{H}$), 2.98 (dd,1H, *J* = 11.2 Hz, 2.0 Hz, H-2), 2.51 (ddd, 1H, *J* = 11.1 Hz, 8.0 Hz, 3.0 Hz, H-5), 0.95 (s, 3H, C-C$\underline{H_3}$); $^{13}C\{^1H\}$ NMR (101 MHz, $CDCl_3$): δ 140.7, 139.4, 138.9, 138.5, 137.8 (arenes, $C_{quart.}$), 128.5, 128.5, 128.4, 128.4, 128.4, 128.4, 128.3, 128.3, 128.3, 127.9, 127.8, 127.8, 127.7, 127.6, 127.6, 127.5, 127.5, 127.2, 127.0, 126.7 (arenes), 88.2 (C-1), 82.0 (C-4), 81.4 (C-3), 75.6 (O-$\underline{C}H_2$Ph), 74.9 (C-5a), 74.7, 73.5, 73.1 (3 x O-$\underline{C}H_2$Ph), 68.0 (C-6), 57.6 (C-2), 56.1 (2 x N-$\underline{C}H_2$Ph), 46.2 (C-5), 22.3 (C-$\underline{C}H_3$); HRMS (ESI) *m/z*: $[M+H]^+$ Calcd for $C_{50}H_{54}NO_5$ 748.3997; Found 784.4012.

(1*R*,2*R*,3*R*,4*R*,5*R*,6*R*)-2,4,5-Tris(benzyloxy)-6-((benzyloxy)methyl)-3-(dibenzylamino)-1-methylcyclohexan-1-ol (18)

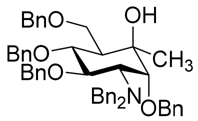

To a stirred solution of **7**[2] (84.0 mg, 114.8 µmol) in dry THF (3 mL) at 0 °C was added $LaCl_3$ x 2LiCl (37.9 mg, 114.8 µmol, 191.3 µL, 0.6 M in THF) and the reaction was stirred for 10 min. To the reaction mixture was added methyl magnesium chloride (9.0 mg, 120.5 µmol, 40.2 µmol, 3 M in THF), and the reaction was stirred for 30 min. The reaction was stopped by adding sat. NH_4Cl_{aq} (2 mL) and water (1 mL). The aqueous layer was extracted with CH_2Cl_2 (3 x 3 mL). The combined organic layers were dried over $MgSO_4$ and concentrated under reduced pressure. The residue was purified by FC (petroleum ether / EtOAc = 12:1 – 8:1) to give compound **18** (39.6 mg, 52.8 µmol, 46 %,) as a colourless oil. Compound **17** (10.9 mg, 14.8 µmol, 13 %) was isolated as a minor product R_f: 0.79 (petroleum ether / EtOAc = 5:1); ^1H NMR (400 MHz, $CDCl_3$): δ 7.49 - 7.04 (m, 30H, arenes), 5.11 (d, 1H, *J* = 11.7 Hz, O-C$\underline{H}$HPh), 4.94 (d, 1H, *J* = 11.7 Hz, O-C$\underline{H}$HPh), 4.85 (d, 2H, *J* = 10.8 Hz, O-C$\underline{H_2}$Ph), 4.69 (d, 1H, *J* = 10.8 Hz, O-C$\underline{H}$HPh), 4.53 (d, 1H, *J* =

10.8 Hz, O-C$\underline{H}$HPh), 4.44 (q, 2H, *J* = 11.6 Hz, O-C$\underline{H_2}$Ph), 4.15 (dd, 1H, *J* = 11.0 Hz, 8.6 Hz, H-3), 4.09 (d, 3H, *J* = 4.4 Hz, N-C$\underline{H_2}$Ph, N-C$\underline{H}$HPh), 4.05 - 3.95 (m, 3H, H-6a, H-4, N-C$\underline{H}$HPh), 3.75 (dd, 1H, *J* = 9.5 Hz, 2.7 Hz, H-6b), 3.68 (s, 1H, O-$\underline{H}$), 3.62 (d, 1H, *J* = 2.6 Hz, H-1), 3.53 (dd, 1H, *J* = 11.0 Hz, 2.6 Hz, H-2), 1.98 – 1.93 (m, 1H, H-5), 1.31 (s, 3H, C-C$\underline{H_3}$); $^{13}C\{^1H\}$ NMR (101 MHz, $CDCl_3$): *δ* 141.0, 139.7, 138.6, 138.4, 137.1 (arenes, $C_{quart.}$), 128.6, 128.5, 128.4, 128.3, 128.3, 128.2, 128.1, 127.7, 127.5, 127.3, 127.1, 127.0, 126.9, 126.4 (arenes), 87.8 (C-1), 81.8 (C-3), 80.5 (C-4), 75.1 (C-5a), 75.0, 73.9, 72.6 (O-$\underline{C}H_2$Ph), 67.2 (C-6), 57.4 (C-2), 56.3 (2 x N-$\underline{C}H_2$Ph), 45.0 (C-5), 25.7 (C-$\underline{C}H_3$); HRMS (ESI) *m*/*z*: $[M+H]^+$ Calcd for $C_{50}H_{54}NO_5$ 748.3997; Found 748.4012.

(1*S*,2*R*,3*R*,4*R*,5*R*,6*R*)-3-Amino-6-(hydroxymethyl)-1-methylcyclohexane-1,2,4,5-tetraol (19)

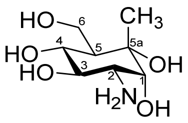

To a stirred solution of **17** (20.0 mg, 26.7 µmol) in dry MeOH (3 mL) were added Pd/C (10% waterwet, 20.0 mg), $Pd(OH)_2$/C (10 % waterwet, 20.0 mg), and TFA (10.0 µL). The reaction was purged with hydrogen and stirred for 24 h under hydrogen atmosphere. The reaction mixture was centrifuged. The supernatant was concentrated and purified by HILIC-HPLC to give compound **19** (3.0 mg, 14.5 µmol, 54 %) as a white powder after lyophilization. ^1H NMR (600 MHz, D_2O): δ 3.84 (m, 2H, H-6a, H-6b), 3.52 (d, 1H, *J* = 2.8 Hz, H-1), 3.44 (dd, 1H, *J* = 10.7 Hz, 8.9 Hz, H-4), 3.36 (dd, 1H, *J* = 11.5 Hz, 8.9 Hz, H-3), 2.83 – 2.79 (m, 1H, H-2), 1.86 (dt, 1H, *J* = 10.7 Hz, 5.0 Hz, H-5), 1.08 (s, 3H, C$\underline{H_3}$); $^{13}C\{^1H\}$ NMR (151 MHz, D_2O): δ 76.3 (C-1), 74.1 (C-4), 73.1 (C-5a), 72.1 (C-3), 62.4, 59.5 (C-6), 52.8 (C-2), 46.0 (C-5), 20.1 ($\underline{C}H_3$); HRMS (ESI) *m*/*z*: $[M+H]^+$ Calcd for $C_8H_{18}NO_5$ 208.1179; Found 208.1183; HPLC (Phenomenex® Luna 5µ HILIC 200Å, AXIA Pa, 250 x 21.20 mm, MeCN/12 mM TEAB buffer pH = 7.00 = 30/70, flow rate = 8.0 mL/min, ELSD): t_R = 11.2 min.

(1*R*,2*R*,3*R*,4*R*,5*R*,6*R*)-3-Amino-6-(hydroxymethyl)-1-methylcyclohexane-1,2,4,5-tetraol (20)

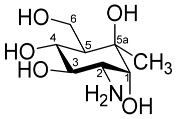

To a stirred solution of **18** (20.0 mg, 26.7 µmol) in dry MeOH (3 mL) was added Pd/C (10% waterwet, 20.0 mg), $Pd(OH)_2$/C (10% waterwet, 20.0 mg) and TFA (10.0 µL). The reaction was purged with hydrogen and stirred for 24h under hydrogen atmosphere. The reaction mixture was filtered over celite. The crude product was purified by HILIC-HPLC to give compound **20** (3.2 mg, 15.4 µmol, 58 %) as a white powder. ^{1}H NMR (500 MHz, D_2O): δ 3.99 – 3.84 (m, 2H, H-6a, H-6b), 3.58 (dd, 1H, *J* = 11.8 Hz, 9.1 Hz, H-4), 3.46 (dd, 1H, *J* = 3.1 Hz, 1.1 Hz, H-1), 3.37 (m, 1H, H-3), 3.09 – 3.03 (m, 1H, H-2), 1.56 – 1.50 (m, 1H, H-5), 1.30 (s, 3H, C-C$\underline{H_3}$); ^{13}C{^{1}H} NMR (126 MHz, D_2O): δ 75.9 (C-1), 75.0 (C-5a), 74.2 (C-3), 70.7 (C-4), 58.9 (C-6), 52.5 (C-2), 45.6 (C-5), 24.4 (C-$\underline{C}H_3$); HRMS (ESI) *m*/*z*: [*M*+H]$^+$ Calcd for $C_8H_{18}NO_5$ 208.1179; Found 208.1181; HPLC (Phenomenex® Luna 5µ HILIC 200Å, AXIA Pa, 250 x 21.20 mm, MeCN/12 mM TEAB buffer pH = 7.00 = 30/70, flow rate = 8.0 mL/min, ELSD): t_R = 10.5 min.

Methyl ((1*R*,2*R*,3*R*,4*R*,5*R*,6*R*)-2,4,5-tris(benzyloxy)-6-((benzyloxy)methyl)-3-(dibenzylamino)-1-methylcyclohexyl) oxalate (21)

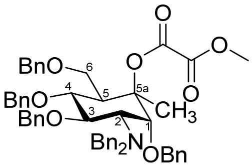

To a stirred solution of **18** (22.0 mg, 29.4 µmol) in dry CH_2Cl_2 (3 mL) was added 4-dimethyl amino pyridine (2.16 mg, 17.65 µmol) and methyl oxalyl chloride (10.8 mg, 88.2 µmol, 8.1 µL). The reaction mixture was stirred for 24 h. The reaction was stopped by adding NH_4Cl_{aq} (2 mL) and water (2 mL). The aqueous layer was extracted with CH_2Cl_2 (3 x 2 mL). The combined organic layers were dried over $MgSO_4$ and concentrated under reduced pressure. The residue was purified by FC (petroleum ether / EtOAc = 20:1 – 16:1) to give compound **21** (18.6 mg, 22.4 µmol, 76 %) as a colourless oil. R_f: 0.58 (petroleum ether / EtOAc = 8:1); ^{1}H NMR (400 MHz, $CDCl_3$): δ 7.44 – 7.02 (m, 30H), 5.06 (d, 1H, *J* = 11.6 Hz), 4.92 (d, 1H, *J* = 11.6 Hz), 4.84 – 4.76 (m, 3H), 4.60 – 4.52 (m, 2H), 4.40 (s, 2H), 4.25 (dd, 1H, *J* = 11.1 Hz, 8.7 Hz), 4.12 (dq, 2H, *J* = 14.3 Hz, 4.8 Hz),

4.01 (d, 2H, 14.3 Hz), 3.76 (dd, 1H, *J* = 10.2 Hz, 6.0 Hz), 3.71 – 3.62 (m, 5H), 3.00 (dd, 1H, *J* = 11.0 Hz, 2.2 Hz), 2.31 (ddd, 1H, *J* = 11.0 Hz, 6.0 Hz, 2.2 Hz), 1.80 (s, 3H), 1.25 (s, 1H); $^{13}C\{^{1}H\}$ NMR (151 MHz, D_2O): δ 157.5, 155.3 (C=O), 140,3, 139.3, 138.5, 138.5, 137.8 ($C_{quart.}$), 128.6, 128.5, 128.5, 128.4, 128.4, 128.4, 128.3, 128.2, 128.2, 127.8, 127.7, 127.7, 127.6, 127.5, 127.5, 127.4, 127.2, 126.7 (arenes), 83.3 (C-1), 81.2 (C-4), 81.1 (C-3), 75.6, 75.2, 74.2, 73.4 (O-CH_2Ph), 66.4 (C-6), 57.0 (C-2), 56.5 (2 x N-CH_2Ph), 53.3 (O-CH_3), 47.3 (C-5), 22.7 (C-CH_3); HRMS (ESI) *m/z*: [*M*+H]$^+$ Calcd for $C_{53}H_{56}NO_8$ 834.4000; Found 834.4016.

(1*S*,2*R*,3*R*,4*R*,5*R*,6*S*)-N,N-dibenzyl-2,3,6-tris(benzyloxy)-4-((benzyloxy)methyl)-5-methylcyclohexan-1-amine (22)

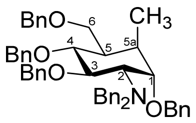

To a stirred solution of **21** (60.0 mg, 71.9 µmol) in dry toluene (2 mL) was added AIBN (71.9 µL, 14.4 µmol, 0.2 M in toluene) and tributyltin hydride (38.8 µL, 143.8 µmol). The reaction mixture was heated (oil bath) to 100 °C and stirred for 2 h. The volatiles were evaporated. The crude product was purified by FC (petroleum ether / EtOAc = 18:1 – 14:1) to give compound **22** (25 mg, 34.2 µmol, 47%) as a colourless oil. R_f: 0.43 (petroleum ether / EtOAc = 10:1); ^{1}H NMR (400 MHz, $CDCl_3$): δ 7.50 – 7.15 (m, 30H, arenes), 5.03 (d, 1H, *J* = 11.5 Hz, O-CHHPh), 4.93 (d, 1H, *J* = 11.5 Hz, O-CHHPh), 4.76 (d, 1H, *J* = 10.7 Hz, O-CHHPh), 4.62 (d, 1H, *J* = 10.8 Hz, O-CHHPh), 4.52 – 4.41 (m, 4H, 2 x O-CH_2Ph), 4.25 (pt, 1H, *J* = 9.6 Hz, H-3), 4.06 (q, 4H, *J* = 13.9 Hz, 2 x N-CH_2Ph), 3.75 – 3.66 (m, 2H, H-1, H-6a), 3.56 – 3.47 (m, 2H, H-4, H-6b), 2.99 (d, 1H, *J* = 11.0 Hz, H-2), 2.53 – 2.36 (m, 2H, H-5, H-5a), 0.61 (d, 3H, *J* = 7.1 Hz, C-CH_3); $^{13}C\{^{1}H\}$ NMR (101 MHz, $CDCl_3$): δ 141.3, 139.8, 138.7, 138.7 ($C_{quart.}$), 128.7, 128.5, 128.4, 128.4, 128.3, 128.2, 127.9, 127.9, 127.7, 127.7, 127.6, 127.5, 127.2, 127.2, 126.6 (arenes), 85.1 (C-1), 81.9, (C-3), 81.9 (C-4), 74.8, 73.3, 73.0, 71.3 (O-CH_2Ph), 69.2 (C-6), 57.1 (C-2), 56.5 (2 x N-CH_2Ph), 39.7 (C-5), 31.3 (C-5a), 12.2 (C-CH_3); HRMS (ESI) *m/z*: [*M*+H]$^+$ Calcd for $C_{50}H_{54}NO_4$ 732.4047; Found: 732.4043.

(1*R*,2*R*,3*S*,4*S*,5*R*,6*R*)-3-Amino-6-(hydroxymethyl)-5-methylcyclohexane-1,2,4-triol (23)

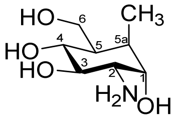

To a stirred solution of **22** (18.00 mg, 24.59 µmol) in dry MeOH (3 mL) were added Pd/C (10 % waterwet, 20.00 mg), $Pd(OH)_2/C$ (10 % waterwet, 20.00 mg), and TFA (14.9 µg, 0.13 µmol, 10.0 µL). The reaction was purged with hydrogen and stirred for 24 h. The reaction mixture was centrifugated. The supernatant was purified by HILIC-HPLC to give compound **23** (2.00 mg, 10.46 µmol, 43 %) as a white powder after lyophilization. ^{1}H NMR (500 MHz, D_2O): δ 3.83 (dd, 1H, *J* =11.2 Hz, 5.1 Hz, , H-6a), 3.93 - 3.90 (m, 1H, H-1), 3.52 (dd, 1H, *J* = 11.0 Hz, 9.5 Hz, H-6b), 3.42 – 3.33 (m, 2H, H-3, H-4), 2.97 (dd, *J* = 1.4 Hz, 10.1 Hz, H-2), 2.14 – 2.07 (m, 1H, H-5a), 2.03 – 1.96 (m, 1H, H-5), 0.92 (d, 3H, *J* = 7.5 Hz, C-$\underline{H}_3$); $^{13}C\{^{1}H\}$ NMR (126 MHz, D_2O): δ 75.0 (C-3), 73.4 (C-1), 71.4 (C-4), 60.9 (C-6), 52.7 (C-2), 40.7 (C-5), 35.1 (C-5a), 10.8 (C-$\underline{C}H_3$); HRMS (ESI) *m/z*: $[M+H]^+$ Calcd for $C_8H_{18}NO$ 192.1230; Found 192.1229; HPLC (Phenomenex® Luna 5µ HILIC 200Å, AXIA Pa, 250 x 21.20 mm, MeCN/12 mM TEAB buffer pH = 7.00 = 30/70, flow rate = 8.0 mL/min, ELSD): t_R = 12.8 min.

(1*S*,2*R*,3*R*,4*R*,5*R*,6*R*)-2,4,5-Tris(benzyloxy)-6-((benzyloxy)methyl)-3-(dibenzylamino)-1-(trifluoromethyl) cyclohexan-1-ol (24)

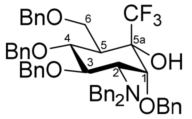

To a stirred solution of **7**[2] (150.0 mg, 204.9 µmol) in dry THF (3 mL) at 0 °C was added trifluoromethyl trimethyl silane (50.3 mg, 415.3 µmol, 60.5 µL) and TBAF (5.4 mg, 20.8 µmol, 20.4 µL, 1 M in THF). The reaction mixture was allowed to reach rt and was stirred for 15 min. The reaction was stopped by adding water (2 mL). The aqueous layer was extracted with CH_2Cl_2 (3 x 4 mL). The combined organic layers were dried over $MgSO_4$ and concentrated under reduced pressure. The residue was dissolved in THF and TBAF (58.9.4 mg, 225.3 µmol, 225.3 µL, 1 M in THF) was added the reaction was stirred for 1 h. The reaction was stopped by adding water (2 mL) The aqueous layer was extracted with CH_2Cl_2 (3 x 4 mL). The combined organic layers were dried over $MgSO_4$ and

concentrated under reduced pressure. The residue was purified by FC (petroleum ether / EtOAc = 10:1 – 5:1) to give compound **24** (95.0 mg, 118.5 µmol, 58 %) as a colourless oil. *R*$_f$: 0.46 (petroleum ether / EtOAc = 6:1); ^{1}H NMR (500 MHz, $CDCl_3$): δ 7.44 – 7.05 (m, 30H, arenes), 5.15 (d, 1H, *J* = 10.3 Hz, O-CHHPh), 4.96 – 4.86 (m, 2H, O-CH_2Ph), 4.72 (d, 1H, *J* = 10.8 Hz, O-CHHPh), 4.64 (d, 1H, *J* = 10.3 Hz, O-CHHPh), 4.53 (d, 1H, *J* = 11.6 Hz, O-CHHPh), 4.39 (d, 1H, *J* = 11.6 Hz, O-CHHPh), 4.33 (d, 1H, 10.8 Hz, O-CHHPh), 4.31 – 4.26 (m, 1H, H-3), 4.07 (d, 1H, *J* = 1.9 Hz, H-1), 4.04 – 3.97 (m, 5H, H-6a, 2 x N-CH_2Ph), 3.62 – 3.54 (m, 1H, H-6b), 3.45 (dd, 1H, *J* = 11.8 Hz, 8.3 Hz, H-4), 3.01 (dd, 1H, *J* = 11.2 Hz, 1.9 Hz, H-2), 2.93 – 2.84 (m, 1H, H-5), 1.44 (s, 1H, O-H); $^{13}C\{^1H\}$ NMR (126 MHz, $CDCl_3$): δ 140.3, 139.2, 138.6, 137.8, 136.8 (arenes, $C_{quart.}$), 128.7, 128.7, 128.6, 128.5, 128.5, 128.4, 128.4, 128.3, 128.2, 128.1, 128.1, 128.0, 127.8, 127.6, 127.3, 127.0, 126.9, 126.8 (arenes), 125.6, 126.6 (CF_3), 80.5 (C-1), 80.3 (C-3, C-4), 80.0, 79.8 (C-5a), 75.9, 75.1, 74.0, 73.0 (O-CH_2Ph), 69.2 (C-6), 56.5 (C-2), 56.1 (2 x N-CH_2Ph), 43.6 (C-5); ^{19}F NMR (377 MHz, $CDCl_3$): δ -70.95 (CF_3); HRMS (ESI) *m/z*: $[M+H]^+$ Calcd for $C_{50}H_{51}F_3NO_5$ 802.3714; Found 802.3724.

(1*R*,2*R*,3*S*,4*R*,5*R*,6*R*)-N,N-dibenzyl-2,5,6-tris(benzyloxy)-4-((benzyloxy)methyl)-3-(trifluoromethyl)-3-((trimethylsilyl)oxy)cyclohexan-1-amine (TMS-24)

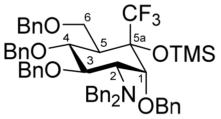

To a stirred solution of **7**[2] (152.0 mg, 207.7 µmol) in THF abs. (3 mL) at 0 °C was added trifluoromethyl trimethyl silane (59.1 mg, 415.3 µmol, 61.5 µL) and TBAF (5.4 mg, 20.8 µmol, 20.8 µL, 1 M in THF). The reaction mixture was allowed to reach rt and was stirred for 15 min. The reaction was stopped by adding water. The aqueous layer was extracted with CH_2Cl_2 (3 x 4 mL). The combined organic layers were dried over $MgSO_4$ and concentrated under reduced pressure. The residue was purified by HPLC to give **TMS-24** (41.2mg, 47.1 µmol, 22 %) as a colourless oil. *R*$_f$: 0.46 (petroleum ether / EtOAc = 8:1); ^{1}H NMR (500 MHz, $CDCl_3$): δ 7.46 – 7.08 (m, 30H, arenes), 5.07 (d, 1H, *J* = 11.7 Hz, O-CHHPh), 4.99 (d, 1H, *J* = 10.6 Hz, O-CHHPh), 4.90 (d, 1H, *J* = 11.7 Hz, O-CHHPh), 4.72 (d, 3H, *J* = 10.6 Hz, O-CHHPh, O-CH_2Ph), 4.41 – 4.26 (m, 2H, O-CH_2Ph), 4.31 (dd, 1H, *J* = 11.1 Hz, 8.5 Hz, H-3), 4.10 – 3.91 (m, 7H, H-4, H-6a, H-1, 2 x N- CH_2Ph), 3.72 (dd, 1H, *J* = 9.8 Hz, 1.4 Hz, H-6b), 3.09 (dd, 1H, *J* = 11.1 Hz, 1.9 Hz, H-2), 2.53 (dd, 1H, *J* = 11.1 Hz, 4.1 Hz,

H-5), 0.16 (s, 9H, Si(C$\underline{H_3}$)$_3$); $^{13}C\{^1H\}$ NMR (126 MHz, $CDCl_3$): δ 140.5, 139.5, 138.9, 138.2, 138.2 (arenes, $C_{quart.}$), 128.6, 128.5, 128.4, 128.4, 128.4, 128.3, 128.3, 128.1, 127.9, 127.8, 127.6, 127.6, 127.5, 127.3, 127.2, 127.0, 126.8 (arenes), 124.1 ($\underline{C}F_3$), 83.7 (C-1), 81.1 (C-3), 80.3 (C-5a), 80.1 (C-4), 75.6, 74.9, 73.3, 73.0 (O-$\underline{C}H_2$Ph), 64.5 (C-6), 56.5, (C-2), 56.3 (2 x N-$\underline{C}H_2$Ph), 46.3 (C-5), 2.6 (Si($\underline{C}H_3$)$_3$); ^{19}F NMR (471 MHz, $CDCl_3$): δ -68.81 (C$\underline{F_3}$); HRMS (ESI) m/z: $[M+H]^+$ Calcd for $C_{53}H_{58}F_3NO_5Si$ 874.4109; Found = 874.4122. HPLC (Kinetex® 5 µm C8 100Å, 250 x 21.2 mm, water /MeCN, 10/90 to 0/100 in 1 min then 0/100, flow rate=8 mL/min, λ = 254 nm): t_R = 4.7 min.

(1*S*,2*R*,3*R*,4*R*,5*R*,6*R*)-3-Amino-6-(hydroxymethyl)-1-(trifluoromethyl) cyclohexane-1,2,4,5-tetraol (25)

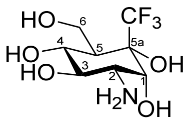

To a stirred solution of **24** (20.0 mg, 26.7 µmol) in dry MeOH (3 mL) was added Pd/C (10 % waterwet, 20.0 mg), $Pd(OH)_2$/C (10 % waterwet, 20.0 mg), and TFA (10.0 µL). The reaction was purged with hydrogen. The reaction was stirred for 24h under hydrogen atmosphere. The reaction mixture was centrifuged. The supernatant was purified by HILIC-HPLC to give compound **25** (2.8 mg, 10.7 µmol, 43 %) as a white powder after lyophilization. ^1H NMR (600 MHz, D_2O): δ 3.95 – 3.82 (m, 3H, H-1, H-6a, H-6b), 3.57 – 3.50 (m, 1H, H-4), 3.46 – 3.32 (m, 1H, H-3), 2.69 – 2.58 (m, 1H, H-2), 2.19 – 2.08 (m, 1H, H-5); $^{13}C\{^1H\}$ NMR (151 MHz, D_2O): δ 126.3 (CF_3), 76.6 (C-5a), 75.1 (C-3), 71.2 (C-1), 70.9 (C-4), 58.6 (C-6), 51.9 (C-2), 45.1 (C-5); ^{19}F NMR (376 MHz, D_2O): δ -71.0 (s, 1H, C$\underline{F_3}$); HRMS (ESI) m/z: $[M+H]^+$ Calcd for $C_8H_{15}F_3NO_5$ 262.0897, Found 262.0901; HPLC (Phenomenex® Luna 5µ HILIC 200Å, AXIA Pa, 250 x 21.20 mm, MeCN/12 mM TEAB buffer pH = 7.00 = 30/70, flow rate = 8.0 mL/min, ELSD): t_R = 9.7 min.

Methyl ((1*S*,2*R*,3*R*,4*R*,5*R*,6*R*)-2,4,5-tris(benzyloxy)-6-((benzyloxy)methyl)-3-(dibenzylamino)-1-(trifluoromethyl)cyclohexyl) oxalate (26)

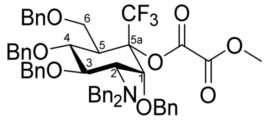

To a stirred solution of **24** (930 mg, 115.9 µmol) in dry THF (3 mL) at -78 °C was added *n*-butyllithium (139.2 µL, 2.5 M in hexane, 347.0 µmol) and methyl oxalyl chloride (21.3 mg, 173.9 µmol, 16.0 µL) The reaction mixture was stirred for 2 h at -78 °C. The reaction was stopped by adding NH_4Cl_{aq} (2 mL) and water (2 mL). The aqueous layer was extracted with CH_2Cl_2 (3 x 2 mL). The combined organic layers were dried over $MgSO_4$ and concentrated under reduced pressure. The residue was purified by FC (petroleum ether / EtOAc = 15:1 – 10:1) to give compound **26** (80.0 mg, 90.0 µmol, 78 %) as a colourless oil. ^{1}H NMR (500 MHz, $CDCl_3$): δ 7.47 – 6.95 (m, 30H, arenes), 5.10 (d, 1H, *J* = 11.6 Hz, O-CHHPh),), 4.93 (m, 2H, O-CHHPh, H-1), 4.78 (d, 1H, *J* = 11.4 Hz, O-CHHPh), 4.73 (m, 3H, O-CHHPh),), 4.53 (d, 1H *J* = 11.4 Hz, O-CHHPh), 4.48 (d, 1H, *J* = 11.5 Hz, O-CHHPh), 4.39 – 4.34 (m, 1H, O-CHHPh), 4.31 (m, 1H, H-3), 4.12 – 4.02 (m, 3H, H-4, N-CH_2Ph), 4.02 – 3.93 (m, 3 H, N- CH_2Ph, H-6), 3.80 (dd, 1H, *J* = 10.4, 1.6 Hz, H-6), 3.75 (s, 3H, OMe), 3.03 (m, 1H,H-2), 2.83 (m, 1H, H-5); ^{13}C{^{1}H} NMR (126 MHz, $CDCl_3$): δ 157.2 (CO), 155.1(CO), 139.9, 139.2, 138.6, 138.1, 137.8, 128.6 (arenes, $C_{quart.}$), 128.5, 128.4, 128.4, 127.7, 127.6, 127.5, 127.2, 127.1, 127.0, 126.9 (arenes), 124.6 (CF_3), 86.5(C-5a), 81.0(C-1), 80.9 (C-3), 80.0 (C-4), 75.7 (O-CH_2Ph), 74.9 (O-CH_2Ph), 73.6 (O-CH_2Ph), 73.4 (O-CH_2Ph), 64.1 (C-6), 56.8 (C-2), 56.4 (2 x N-CH_2Ph), 53.7(OMe), 45.3(C-5); ^{19}F NMR (471 MHz, $CDCl_3$): δ -64.45(CF_3); HRMS (ESI) *m/z:* [*M*+H]$^+$ Calcd for $C_{53}H_{53}F_3NO_8$ 888.3718; Found 888.3708.

(1*S*,2*R*,3*R*,4*R*,5*R*,6*S*)-N,N-dibenzyl-2,3,6-tris(benzyloxy)-4-((benzyloxy)methyl)-5-(trifluoromethyl)cyclohexan-1-amine (27) and (1*S*,2*R*,3*R*,4*R*,5*S*,6*S*)-N,N-dibenzyl-2,3,6-tris(benzyloxy)-4-((benzyloxy)methyl)-5-(trifluoromethyl)cyclohexan-1-amine (28)

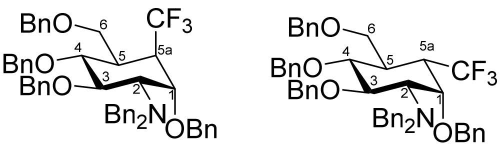

To a stirred solution of **26** (80.0 mg, 90.1 µmol) in degassed dry toluene (3 mL) was added AIBN (18.02 3 mg, 18.0 µmol,) and tributyltin hydride (48.6 µL, 180.2 µmol). The reaction mixture was heated (oil bath) to 100 °C and stirred for 15 h. The reaction mixture was concentrated. The crude product was purified using HPLC to give compounds **27** (38 mg, 48.4 µmol, 54 %) and **28** (7 mg, 8.9 µmol, 10 %) as a colourless oils. **27:** ^{1}H NMR (500 MHz, $CDCl_3$): δ 7.47 – 7.07 (m, 30H, arenes), 5.01 (d, 1H, *J* = 11.5 Hz, O-C<u>H</u>HPh), 4.91 (d, 1H, *J* = 11.6 Hz, O-C<u>H</u>HPh), 4.73 (d, 1H, *J* = 10.7 Hz, O-C<u>H</u>HPh), 4.52 (m, 2H, O-C<u>H</u>HPh), 4.49 – 4.44 (m, 2H, O-C<u>H</u>HPh), 4.44 – 4.37 (m, 1H, O-C<u>H</u>HPh), 4.20 (dd, 1H, *J* = 11.1, 8.3 Hz, H-3), 4.15 (pt, 1H, *J* = 2.5 Hz, H-1), 4.05 (d, 2H, *J* = 13.9 Hz, N- C<u>H$_2$</u>Ph), 3.97 (d, 2H, *J* = 13.9 Hz, N- C<u>H$_2$</u>Ph), 3.77 (dd, 1H, *J* = 9.2, 3.9 Hz, H-6), 3.68 (dd, 1H, *J* = 11.9, 8.3 Hz, H-4), 3.52 (pt, 1H, *J* = 9.7 Hz, H-6), 3.02 (dd, 1H, *J* = 11.1, 2.4 Hz, H-2), 2.99 – 2.88 (m, 1H, H-5a), 2.63 (m,1H, H-5); ^{13}C{^{1}H} NMR (126 MHz, $CDCl_3$): δ 140.6, 139.4, 138.4, 138.2, 137.4 (arenes, $C_{quart.}$), 128.6, 128.5, 128.5, 128.4, 128.4, 128.2, 128.2, 127.9, 127.9, 127.8, 127.7, 127.6, 127.3 (arenes), 127.1(CF_3), 126.8, 126.6 (arenes), 81.5 (C-3), 81.1 (C-4), 78.5 (C-1), 75.1 (O-<u>C</u>H$_2$Ph), 73.3 (O-<u>C</u>H$_2$Ph), 73.1 (O-<u>C</u>H$_2$Ph), 72.3 (O-<u>C</u>H$_2$Ph), 67.4 (C-6), 58.1 (C-2), 56.5 (2 x N-<u>C</u>H$_2$Ph), 41.8 (C-5a), 38.4 (C-5); ^{19}F NMR (377 MHz, $CDCl_3$): δ -60.29 (d, *J* = 10.9 Hz). HRMS (ESI) *m/z*: [*M*+H]$^+$ Calcd for $C_{50}H_{51}F_3NO_4$ 786.3765; Found 786.3758; HPLC (Kinetex® 5 µm C8 100Å, 250 x 21.2 mm, MeCN, 10 mL/min, λ = 254 nm): t_R = 13.6 min. **28:** ^{1}H NMR (500 MHz, $CDCl_3$): δ 7.58 – 6.87 (m, 30H, arenes), 5.07 (d, 1H, *J* = 11.6 Hz, O-C<u>H</u>HPh), 4.87 (d, 1H, *J* = 11.7 Hz, O-C<u>H</u>HPh), 4.76 (d, 1H, *J* = 10.7 Hz, O-C<u>H</u>HPh), 4.68 (s, 2H, O-C<u>H</u>HPh), 4.52 (d, 1H, *J* = 10.8 Hz, O-C<u>H</u>HPh), 4.44 (d, 1H, *J* = 11.6 Hz,), 4.38 – 4.31 (m, 1H, O-C<u>H</u>HPh), 4.29 (m, 1H, H-1), 4.22 (pt, 1H, *J* = 9.7 Hz, H-3), 4.12 (d, 2H, *J* = 14.7 Hz, N- C<u>H$_2$</u>Ph), 4.01 (d, 2H, *J* = 14.6 Hz, N- C<u>H$_2$</u>Ph), 3.95 (d, 1H, *J* = 9.7 Hz, H-6), 3.63 (pt, 1H, *J* = 9.6 Hz, H-4), 3.48 (d, 1H, *J* = 9.7 Hz, H-6), 2.68 (d, 1H, *J* = 10.8 Hz, H-2), 2.33 (m, 1H, H-5), 2.29 – 2.16 (m, 1H, H-5a); ^{13}C{^{1}H} NMR (126 MHz, $CDCl_3$): δ 140.5, 139.4, 138.4, 138.0 (arenes, $C_{quart.}$), 128.5, 128.5, 128.5, 128.4, 128.3, 128.2, 128.0, 127.9, 127.8, 127.7, 127.3,

127.0, 126.8 (arenes), 82.2 (C-4), 81.1 (C-3), 79.0 (C-1), 75.3 (O-$\underline{C}H_2$Ph), 73.9 (O-$\underline{C}H_2$Ph), 73.4 (O-$\underline{C}H_2$Ph), 73.3 (O-$\underline{C}H_2$Ph), 65.6 (C-6), 61.4 (C-2), 56.3 (2 x N-$\underline{C}H_2$Ph), 43.8 (C-5a), 38.1 (C-5); ^{19}F NMR (377 MHz, $CDCl_3$): δ -62.57 (d, J = 8.5 Hz); HRMS (ESI) m/z $[M+H]^+$ Calcd for $C_{50}H_{51}F_3NO_4$ 786.37647; Found 786.3764; HRMS (ESI) m/z: [M+H]+ Calcd for $C_{50}H_{51}F_3NO_4$ 786.3765; Found 786.3758; HPLC (Kinetex® 5 µm C8 100Å, 250 x 21.2 mm, MeCN, 10 mL/min, λ = 254 nm): t_R = 17.1 min.

(1*S*,2*R*,3*R*,4*R*,5*R*,6*S*)-*N*,*N*-Dibenzyl-2,3,6-tris(benzyloxy)-4-((benzyloxy)methyl)-5-(trifluoromethyl)cyclohexan-1-amine (29)

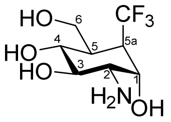

To a stirred solution of **27** (20.0 mg, 25.5 µmol) in dry MeOH (2 mL) was added Pd/C (10 % waterwet, 20.0 mg), $Pd(OH)_2$/C (20 % waterwet, 20.0 mg), and TFA (20.0 µL). The reaction was purged with hydrogen and stirred for 12 h under hydrogen atmosphere. The reaction mixture was centrifuged. The supernatant was purified by HILIC-HPLC to give compound **29** (4.4 mg, 17.9 µmol, 71 %) as a white powder after lyophilization with minor impurities. ^{1}H NMR (500 MHz, D_2O): δ 4.32 (pt, 1H, J = 2.9 Hz, H-1), 4.05 (dd, 1H, J = 11.7, 5.3 Hz, H-6), 3.77 (m, 1H, H-6), 3.70 – 3.58 (m, 1H, H-4), 3.45 (m, 1H, H-3), 2.99 (m, 1H, H-2), 2.77 (m, 1H, H-5a), 2.35 (m, 1H, H-5); ^{13}C{^{1}H} NMR (126 MHz, D_2O): δ 75.6 (C-3), 71.5 (C-4), 67.4 (C-1), 59.9 (C-6), 53.3 (C-2), 45.4 (C-5a), 39.4 (C-5); ^{19}F NMR (377 MHz, $CDCl_3$): δ -60.27 (d, J = 12.4 Hz); HRMS (ESI) m/z: $[M+H]^+$ Calcd for $C_8H_{15}F_3NO_4$ 246.0948; Found 246.0945; HPLC (Phenomenex® Luna 5µ HILIC 200Å, AXIA Pa, 250 x 21.20 mm, MeCN/12 mM TEAB buffer pH = 7.00 = 30/70, flow rate = 8.0 mL/min, ELSD): t_R = 14.7 min.

(1*S*,2*R*,3*R*,4*R*,5*S*,6*S*)-*N*,*N*-Dibenzyl-2,3,6-tris(benzyloxy)-4-((benzyloxy)methyl)-5-(trifluoromethyl)cyclohexan-1-amine (30)

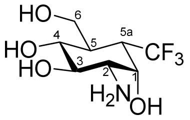

To a stirred solution of **28** (5.0 mg, 6.4 µmol) in dry MeOH (0.8 mL) was added Pd/C (10 % waterwet, 5.0 mg), $Pd(OH)_2/C$ (20 % waterwet, 5.0 mg), and TFA (5.0 µL). The reaction was purged with hydrogen and stirred for 12 h under hydrogen atmosphere. The reaction mixture was centrifuged. The supernatant was purified by HILIC-HPLC to give compound **30** (0.5 mg, 2.0 µmol, 32 %) as a white powder after lyophilization with minor impurities. Because of the limited amount of product, only ^{1}H and ^{19}F NMR data are reported. ^{1}H NMR (500 MHz, D_2O): δ 4.26 (pt, 1H, *J* = 2.6 Hz, H-1), 3.98 (d, 1H, *J* = 12.0 Hz, H-6), 3.62 (d, 1H, *J* = 12.0 Hz, H-6), 3.44 – 3.34 (m, 2H, H-3, H-4), 2.63 (m, 1H, H-2), 2.58 – 2.47 (m, 1H, H-5a), 1.94 – 1.83 (m, 1H, H-5); ^{19}F NMR (377 MHz, D_2O): δ -63.64 (d, *J* = 11.0 Hz); HRMS (ESI) *m*/*z*: [*M*+H]$^+$ calcd for $C_8H_{15}F_3NO_4$ 246.0948; Found 246.0944; HPLC (Phenomenex® Luna 5µ HILIC 200Å, AXIA Pa, 250 x 21.20 mm, MeCN/12 mM TEAB buffer pH = 7.00 = 30/70, flow rate = 8.0 mL/min, ELSD): t_R = 15.2 min.

(1*R*,2*R*,3*S*,4*R*,5*R*,6*S*)-2,4,5-Tris(benzyloxy)-6-((benzyloxy)methyl)-3-(dibenzylamino)cyclohexane-1-ol (31)

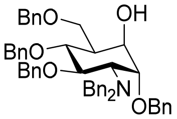

The ketone **7**[2] (130 mg, 177.8 µmol) was dissolved in anhydrous THF (5 mL) cooled to -20 °C and (*R*)-(+)-2-methyl-CBS-oxazaborolidine (1 M in THF, 53 µL, 53 µM) was added. After stirring for 5 min, BH_3•SMe_2 (5 M in THF, 35 µL, 177.6 µmol) was added slowly. After slowly warming to room temperature, the reaction was stopped by the addition of water (2 mL) and extracted with DCM (3 x 3 mL). The combined organic phases were dried over $MgSO_4$ and concentrated under reduced pressure. The crude product was purified by HPLC to yield **31** (98 mg, 133.5 µmol, 75 %). ^{1}H NMR ($CDCl_3$, 400 MHz): δ 7.55 – 7.10 (m, 30H, H-arenes), 5.03 (d, 1H, *J* = 11.7 Hz, O-CHHPh), 4.92 (d, 1H, *J* = 11.7 Hz, O-CHHPh), 4.81 (d, 1H, *J* = 10.8 Hz, O-CHHPh), 4.62 (d, *J* = 10.8 Hz, 1H, O-CHHPh), 4.58 – 4.46 (m, 2H, 2x O-CHHPh), 4.44 (m, 2H, 2x O-CHHPh), 4.21 – 4.13 (m, 2H,

H-3, H-5a), 4.05 (m, 5H, 4x N-C<u>H</u>HPh, 1x H-6), 4.00 – 3.87 (m, 2H, H-4, H-1), 3.65 (m, OH, 1x H-6), 3.37 (dd, 1H, *J* = 11.0, 2.6 Hz, H-2), 2.09 (m, 1H, H-5a); $^{13}C\{^1H\}$ NMR ($CDCl_3$, 201 MHz): δ 141.2, 139.8, 138.8, 138.4, 137.3 ($C_{quart.}$ arenes), 128.8, 128.6, 128.5, 128.4, 128.4, 128.2, 128.2, 128.2, 127.9, 127.7, 127.7, 127.2, 127.1, 126.6 (C_{arenes}), 81.9 (C-1), 81.5 (C-3), 79.4 (C-4), 75.3 (O-<u>C</u>H_2Ph), 73.9 (O-<u>C</u>H_2Ph), 73.0 (O-<u>C</u>H_2Ph), 72.8 (O-<u>C</u>H_2Ph), 71.1 (C-5a), 70.6 (C-6), 57.4 (C-2), 56.5 (2x N-<u>C</u>H_2Ph), 41.8 (C-5); HRMS (ESI) *m/z*: $[M+H]^+$ calcd for $C_{49}H_{52}NO_5$ 734.3840; Found 734.3830; HPLC (Kinetex® 5 µm C18 100Å, 250 x 21.2 mm, MeCN, flow rate= 30mL/min, λ = 254 nm): t_R = 3.8 min.

(1*R*,2*R*,3*R*,4*R*,5*R*,6*R*)-3-Amino-6-(hydroxymethyl)cyclohexane-1,2,4,5-tetraol (32)

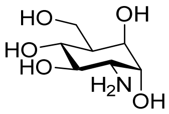

To a stirred solution of **31** (8.0 mg, 10.9 µmol) in dry MeOH (3 mL) was added Pd/C (10 % waterwet, 8.0 mg), $Pd(OH)_2$/C (20 % waterwet, 8.0 mg), and TFA (8.0 µL). The reaction was purged with hydrogen and stirred for 24 h under hydrogen atmosphere. The reaction mixture was filtered over celite. The crude product was purified by HILIC-HPLC to give compound **32** (1.50 mg, 10.9 µmol, 71 %) as a white powder. ^1H NMR (D_2O, 500 MHz): δ 4.13 (pt, 1H, *J* = 3.2 Hz, H-5a), 4.07 (pt, 1H, *J* = 3.5 Hz, H-1), 3.96 (dd, 1H *J* = 11.1, 4.4 Hz, 1x H-6), 3.81 (dd, 1H, *J* = 11.1, 8.7 Hz, 1x H-6), 3.65 (dd, 1H, *J* = 10.6, 9.1 Hz, H-3), 3.55 (dd, 1H, *J* = 11.3, 9.1 Hz, H-4), 3.30 (dd, 1H, *J* = 10.6, 3.1 Hz, H-2), 2.03 (ptdd, 1H, *J* = 11.3, 5.6, 3.5 Hz, H-5); $^{13}C\{^1H\}$ NMR (101 MHz, D_2O): δ 73.1 (C-3), 70.3 (C-4), 70.2 (C-1), 69.2 (C-5a), 59.4 (C-6), 52.6 (C-2), 42.5 (C-5); HRMS (ESI) *m/z*: $[M+H]^+$ Calcd for $C_7H_{16}NO_5$ 194.1023; Found 194.1027; HPLC (Phenomenex® Luna 5µ HILIC 200Å, AXIA Pa, 250 x 21.20 mm, MeCN/12 mM TEAB buffer pH = 7.00 = 30:70, flow rate = 8.0 mL/min, ELSD): t_R = 17.4 min.

(1*S*,2*R*,3*R*,4*S*,5*R*,6*R*)-*N*,*N*-Dibenzyl-2,3,6-tris(benzyloxy)-4-((benzyloxy)methyl)-5-methoxycyclohexan-1-amine (33)

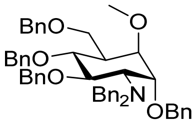

Alcohol **31** (80.0 mg, 109 µmol) was dissolved in THF (4 mL) and NaH (60 wt%, 13.0 mg, 327.0 µmol) was added. After stirring for 10 min, MeI (27.1 mL, 436.0 µmol) was added. After 3h, MeOH (3 mL) was added. Water (5 mL) was added, and the aqueous phase was extracted with DCM (3x 10 mL). The combined organic layers were dried over $MgSO_4$ and the solvent was evaporated under reduced pressure. The crude product was purified by FC on silica gel (EtOAc/petroleum ether 1:10) and the product **33** was isolated as a yellow oil (57.0 mg, 76.2 µmol, 90 %). ^{1}H NMR ($CDCl_3$, 500 MHz): δ 7.45 – 7.10 (m, 30H, H-arenes), 5.02 (d, 1H, *J* = 11.7 Hz, O-C<u>H</u>HPh), 4.90 (d, 1H *J* = 11.7 Hz, O-C<u>H</u>HPh), 4.70 (d, 1H, *J* = 10.7 Hz, O-C<u>H</u>HPh), 4.60 – 4.54 (m, 2H, 2x O-C<u>H</u>HPh), 4.49 (d,1H, *J* = 12.2 Hz, O-C<u>H</u>HPh), 4.43 (d, 1H, *J* = 12.2 Hz, O-C<u>H</u>HPh), 4.38 (d, 1H, *J* = 10.7 Hz, O-C<u>H</u>HPh), 4.17 (dd, 1H, *J* = 11.0, 8.5 Hz, H-3), 4.08 (d, 2H, *J* = 13.8 Hz, 2x N-C<u>H</u>HPh), 3.98 (d, 2H, *J* = 13.8 Hz, N-C<u>H</u>HPh), 3.96 (pt, 1H, *J* = 3.1 Hz, H-1), 3.69 (dd, 1H, *J* = 9.0, 4.0 Hz, 1x H-6), 3.62 (pt, 1H, *J* = 3.3 Hz, H-5a), 3.57 (dd, 1H, *J* = 10.4, 9.0 Hz, 1x H-6), 3.45 (dd, 1H, *J* = 11.5, 8.5 Hz, H-4), 3.13 (dd, 1H, *J* = 11.0, 2.5 Hz, H-2), 2.97 (s, 3H, CH_3), 2.42 (ptpt, 1H, *J* = 10.9, 3.4 Hz, H-5); ^{13}C{^{1}H} NMR ($CDCl_3$, 126 MHz): δ 141.3, 139.8, 138.9, 138.5, 138.2 ($C_{quart.\ arenes}$), 128.8, 128.5, 128.4, 128.2, 128.1, 127.9, 127.7, 127.9, 127.5, 127.2, 127.0, 126.6 (C_{arenes}), 81.9 (C-4), 81.4 (C-3), 79.3 (C-1), 74.9 (C-5a), 74.7 (O-<u>C</u>H_2Ph), 73.0 (O-<u>C</u>H_2Ph), 72.9 (O-<u>C</u>H_2Ph), 72.8 (O-<u>C</u>H_2Ph), 67.1 (C-6), 57.4 (CH_3), 56.7 (C-2), 56.6 (2x N-<u>C</u>H_2Ph), 41.9 (C-5); HRMS (ESI) *m/z*: [*M*+H]$^+$ Calcd for $C_{50}H_{54}NO_5$ 748.3997; Found 748.3990.

(1*R*,2*R*,3*R*,4*R*,5*R*,6*R*)-3-Amino-6-(hydroxymethyl)-5-methoxycyclohexane-1,2,4-triol (34)

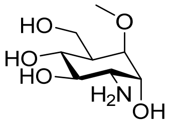

To a stirred solution of **33** (18.0 mg, 24.1 µmol) in dry MeOH (3 mL) was added Pd/C (10 % waterwet, 18.0 mg), $Pd(OH)_2$/C (20 % waterwet, 18.0 mg), and TFA (18.0 µL). The reaction was purged with hydrogen and stirred for 24 h under hydrogen atmosphere. The

reaction mixture was filtered over celite. The crude product was purified by HILIC-HPLC to give compound **34** (3.70 mg, 17.9 µmol, 74 %) as a white powder after lyophilization. ^{1}H NMR ($CDCl_3$, 600 MHz): δ 4.29 (pt, 1H *J* = 3.2 Hz, H-1), 3.90 (dd, 1H, *J* = 11.0, 4.2 Hz, 1x H-6), 3.74 – 3.66 (m, 2 H, 1x H-6, H-5a), 3.59 (dd, 1H, *J* = 10.7, 9.1 Hz, H-3), 3.42 (s, 3H, CH_3), 3.41 – 3.37 (m, 1H, H-4), 3.18 (dd, 1H, *J* = 10.7, 3.2 Hz, H-2), 2.03 (dddd, 1H, *J* = 11.9, 9.2, 4.2, 2.9 Hz, H-5); ^{13}C{^{1}H} NMR (151 MHz, D_2O): δ 78.7 (C-5a), 72.0 (C-3), 70.4 (C-4), 65.4 (C-1), 58.9 (C-6), 58.1 (CH_3, 52.9 (C-2), 42.5 (C-5); HRMS (ESI) *m*/*z*: [*M*+H]$^{+}$ Calcd for $C_8H_{18}NO_5$ 208.1179; Found 208.1177; HPLC (Phenomenex® Luna 5µ HILIC 200Å, AXIA Pa, 250 x 21.20 mm, MeCN/12 mM TEAB buffer pH = 7.00 = 30/70, flow rate = 8.0 mL/min, ELSD): t_R = 16.7 min.

(1*R*,2*R*,4*R*,5*R*,6*R*)-*N*,*N*-Dibenzyl-2,5,6-tris(benzyloxy)-4-((benzyloxy)methyl)-3,3-difluorocyclohexan-1-amine (35)

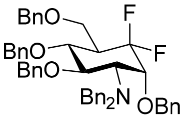

HPLC-purified ketone **7**[2] (300 mg, 409.9 µmol) was dissolved in dry toluene (2 mL) in a Teflon reaction flask. DAST (541 µL, 4.1 mmol) was added followed by the addition of boron trifluoride diethyl etherate (67 µL, 409.9 µmol). The reaction flask was sealed and stirred overnight. The reaction mixture was slowly added into 1M aqueous NaOH solution (5 mL) and extracted with DCM (3x 2 mL). The combined organic layers were dried over $MgSO_4$ and concentrated under reduced pressure. HPLC purification resulted in a 3:1 mixture of the desired compound **35** and the elimination product **36** (47 mg, approximately 62 µmol, 15 %). To facilitate separation from byproduct **36** and obtain a sample of **35** for analytical purposes, the double bond of **36** was selectively hydrogenated generating monofluorinated carbasugar. To this end, a sample of 10 mg of the mixture of **35** and **36** was dissolved in in dry MeOH (1 mL) and Pd/C (10 % waterwet, 5.0 mg) was added. The reaction was purged with hydrogen and stirred for 8 h under hydrogen atmosphere. The reaction mixture was filtered, and the obtained product was purified by HPLC. Compound **35** was obtained with a small amount of byproduct **36** (approximately 10 % based on ^{19}F NMR) still remaining. ^{1}H NMR ($CDCl_3$, 500 MHz): δ 7.46 – 7.05 (m, 30H), 5.01 (d, 1H, *J* = 11.6 Hz, O-CHHPh), 4.86 (d, 1H, *J* = 11.6 Hz, O-CHHPh), 4.76 (d, 1H, *J* = 10.6 Hz, O-CHHPh), 4.62 (m, 2H, 2x O-CHHPh), 4.53 (d, 1H, *J* = 12.3 Hz, O-CHHPh), 4.49 (d, 1H,

J = 12.3 Hz, O-CHHPh), 4.23 (dd, 1H, *J* = 11.2, 8.6 Hz, H-3), 4.07 – 3.94 (m, 5H, H-1, 4x N-CHHPh), 3.84 (dd, 1H, *J* = 10.2, 4.3 Hz, 1x H-6), 3.80 (dd, 1H, *J* = 10.1, 2.1 Hz, 1x H-6), 3.74 (dd, 1H, *J* = 11.2, 8.6 Hz, H-4), 3.05 (d, 1H, *J* = 11.2 Hz, H-2), 2.61 – 2.47 (m, 1H, H-5); $^{13}C\{^{1}H\}$ NMR ($CDCl_3$, 126 MHz): δ 140.3, 139.3, 138.4, 138.2, 137.5 ($C_{quart.\ arenes}$), 128.5, 128.5, 128.2, 128.0, 127.9, 127.8, 127.7, 127.4, 127.0, 126.9 (C_{arenes}), 80.7 (C-1), 80.2 (C-3), 79.4 (C-4), 75.3 (O-CH_2Ph), 75.0 (O-CH_2Ph), 73.4 (O-CH_2Ph), 73.1(O-CH_2Ph), 63.7 (C-6), 57.1 (C-2), 56.2 (2x N-CH_2Ph), 44.1 (C-5); ^{19}F NMR (471 MHz, $CDCl_3$): δ -108.9 (d, *J* = 256.3 Hz), -109.8 (dd, *J* = 256.3, 28.3 Hz); HPLC (Kinetex® 5 µm C18 100Å, 250 x 21.2 mm, MeCN, flow rate= 30 mL/min, λ = 254 nm) t_R = 3.6 min.

(1*R*,2*R*,3*R*,4*R*,6*R*)-3-Amino-5,5-difluoro-6-(hydroxymethyl)cyclohexane-1,2,4-triol (37)

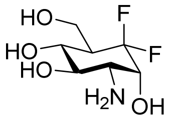

To a stirred solution of the 3:1 mixture of **35** and **36** (30.0 mg, 6.8 µmol) in dry MeOH (4 mL) was added Pd/C (10 % waterwet, 30.0 mg), $Pd(OH)_2$/C (20 % waterwet, 30.0 mg), and TFA (20.0 µL). The reaction was purged with hydrogen and stirred for 24 h under hydrogen atmosphere. The catalyst was filtrated off by using a syringe filter, which was washed with 20 mL of methanol. The solvent was evaporated under reduced pressure. The crude product was purified by HILIC-HPLC to give compound **37** (5.4 mg, 25.3 µmol, 64 %) as a white powder after lyophilization. ^{1}H NMR (D_2O, 500 MHz): δ 4.27 (m,1H, H-1), 4.08 – 4.01 (m, 2H, H-6), 3.86 (dd, 1H, *J* = 10.9, 9.2 Hz, H-3), 3.61 (dd, 1H, *J* = 11.3, 9.2 Hz, H-4), 3.41 (m, 1H, H-2), 2.43 – 2.32 (m, 1H, H-5); $^{13}C\{^{1}H\}$ NMR (D_2O, 151 MHz, ^{19}F-coupled): δ 121.7 (dd, *J* = 252.8, 244.6 Hz, C-5a), 70.3 (C-3), 68.8 (d, *J* = 10.8 Hz, C-4), 67.7 (dd, *J* = 34.3, 26.8 Hz, C-1), 55.4 (d, *J* = 2.8 Hz, C-6), 52.2 (d, *J* = 9.7 Hz, C-2), 44.0 (t, *J* = 19.6 Hz, C-5); $^{13}C\{^{1}H\}$ NMR (D_2O, 126 MHz, ^{19}F-decoupled): δ 121.9 (C-5a), 70.4 (C-3), 69.0 (C-4), 68.0 (C-1), 55.6 (C-6), 52.3 (C-2), 44.2 (C-5); ^{19}F NMR (471 MHz, D_2O): δ -109.90 (d, *J* = 261.4 Hz), -114.53 (dd, *J* = 261.4, 30.4 Hz); HRMS (ESI) *m/z*: $[M+H]^+$ Calcd for $C_7H_{14}F_2NO_4$ 214.0885; Found 214.0888; HPLC (Phenomenex® Luna 5µ HILIC 200Å, AXIA Pa, 250 x 21.20 mm, MeCN/12 mM TEAB buffer pH = 7.00 = 30/70, flow rate = 8.0 mL/min, ELSD): t_R = 9.2 min.

(1*R*,2*R*,3*R*,4*R*,5*R*,6*R*)-*N*,*N*-Dibenzyl-2,3,6-tris(benzyloxy)-4-((benzyloxy)methyl)-5-fluorocyclohexan-1-amine (39) and (1*R*,2*R*,3*R*,4*R*,5*S*,6*S*)-*N*,*N*-dibenzyl-2,3,5-tris(benzyloxy)-4-((benzyloxy)methyl)-6-fluorocyclohexan-1-amine (40)

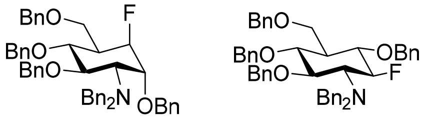

A boron trifluoride diethyl etherate solution (1 % in toluene, 4.5 mL, 258.9 µmol) was cooled to 0 °C and DAST (342 µL, 2.6 mmol) was added. The mixture was stirred for 1 h and then cooled to -78 °C. Alcohol **31** (190 mg, 258.9 µmol) was dissolved in dry toluene (1 mL) and slowly added to the DAST solution. The reaction was allowed to warm up to room temperature overnight. The reaction mixture was slowly added into 1 M aqueous NaOH solution (5 mL) and extracted with DCM (3x 2 mL). The combined organic layers were dried over $MgSO_4$ and concentrated under reduced pressure. HPLC purification resulted in axial fluoride **39** (110 mg, 149.5 µmol, 58 %) as well as the rearrangement product **40** (25 mg, 34.0 µmol, 13 %). **39**: ^{1}H NMR ($CDCl_3$, 400 MHz): δ 7.52 – 7.07 (m, 30H), 5.03 – 4.84 (m, 3H, 2x O-CHHPh, H-5a), 4.74 (d, 1H, *J* = 10.8 Hz, O-CHHPh), 4.61 (d, 1H, *J* = 10.8 Hz, O-CHHPh), 4.55 (d, 1H, J = 10.8 Hz, O-CHHPh), 4.47 (m, 2H, 2x O-CHHPh), 4.40 (d, 1H, *J* = 10.8 Hz, O-CHHPh), 4.20 (dd, 1H, *J* = 11.1, 8.6 Hz, H-3), 4.10 – 3.98 (m, 5H, 4x N-CHHPh, H-1), 3.71 (dd, 1H, *J* = 9.1, 4.0 Hz, 1x H-6), 3.49 (m, 2H, 1x H-6, H-4), 3.12 (dt, 1H, *J* = 11.1, 3.2 Hz, H-2), 2.50 – 2.30 (m, 1H, H-5); ^{13}C{^{1}H} NMR ($CDCl_3$, 101 MHz): δ 140.9, 139.5, 138.6, 138.3, 137.8 ($C_{quart.}$), 128.6, 128.5, 128.5, 128.5, 128.3, 128.1, 128.0, 127.9, 127.8, 127.6, 127.3, 127.1, 126.8 (C_{arenes}), 86.9 (d, *J* = 177.5 Hz, C-5a), 80.8 (C-3, C-4), 79.6 (d, J = 25.5 Hz, C-1) , 75.2 (O-CH_2Ph), 73.4 (O-CH_2Ph), 73.2 (O-CH_2Ph), 73.1 (O-CH_2Ph), 66.6 (C-6), 57.3 (C-2), 56.6 (C-2), 42.1 (d, *J* = 25.5 Hz, C-5); ^{19}F NMR (377 MHz, $CDCl_3$): δ -203.63 (dd, *J* = 46.9, 37.0 Hz); HPLC (Kinetex® 5 µm C18 100Å, 250 x 21.2 mm, MeCN, flow rate = 30mL/min, λ = 254 nm): t_R = 4.1 min. **40**: ^{1}H NMR ($CDCl_3$, 500 MHz): δ 7.82 – 6.79 (m, 30H, H-arenes), 4.99 (m, 2H, 2x O-CHHPh), 4.89 (d, 1H, *J* = 10.7 Hz, O-CHHPh), 4.75 (ddd, 1H, *J* = 51.4, 10.4, 8.7 Hz, H -1), 4.51 (m, 2H, 2x O-CHHPh), 4.43 – 4.34 (m, 2H, 2x O-CHHPh), 3.98 (d, 2H, *J* = 14.0 Hz, 2x N-CHHPh), 3.92 (d, 2H, *J* = 14.0 Hz, 2x N-CHHPh), 3.79 – 3.73 (m, 2H, 2x H-6), 3.69 (ddd, 1H, *J* = 12.6, 11.4, 8.7 Hz, H-5a), 3.65 – 3.55 (m, 2H, H-3, H-4), 3.07 (m, 1H, H-2), 1.57 (m, 1H, H-5); ^{13}C{^{1}H} NMR ($CDCl_3$, 126 MHz): δ 139.9, 139.2, 138.6, 138.5, 138.1 ($C_{quart.}$), 128.9, 128.6, 128.5, 128.5, 128.3, 128.3, 128.2, 128.0, 127.9, 127.9, 127.8, 127.4, 127.2, 126.9 (C_{arenes}), 96.4 (d, *J* = 182.9 Hz, C-1), 81.8 (d, *J* = 9.7 Hz, C-3), 79.1 (C-4), 76.7 (d, *J* = 17.2 Hz, C-5a), 75.5 (O-CH_2Ph), 75.3 (O-CH_2Ph),74.4 (O-

CH2Ph), 73.4 (O-CH2Ph), 64.3 (C-6), 60.9 (d, *J* = 15.7 Hz, C-2), 55.0 (2x N-CH2Ph), 44.4 (d, *J* = 9.7 Hz, C-5); ^{19}F NMR (377 MHz, $CDCl_3$): δ -186.3 (dpt, *J* = 51.4, 11.4 Hz); HRMS (ESI) *m/z*: [*M*+H]$^+$ Calcd for $C_{49}H_{51}FNO_4$ 736.3797; Found 736.3787; HPLC (Kinetex® 5 µm C18 100Å, 250 x 21.2 mm, MeCN, flow rate = 30mL/min, λ = 254 nm): t_R = 5.1 min.

(1*R*,2*R*,3*R*,4*S*,5*S*,6*R*)-6-Amino-5-fluoro-3-(hydroxymethyl)cyclohexane-1,2,4-triol (41)

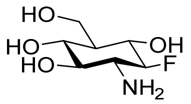

To a stirred solution of **39** (5.0 mg, 6.8 µmol) in dry MeOH (1.5 mL) was added Pd/C (10 % waterwet, 5.0 mg), $Pd(OH)_2$-C (20 % waterwet, 5.0 mg), and TFA (7.0 µL). The reaction was purged with hydrogen and stirred for 12 h under hydrogen atmosphere. The reaction mixture was centrifuged. The supernatant was purified by HILIC-HPLC to give compound **41** (1.1 mg, 5.6 µmol, 83 %) as a white powder after lyophilization. ^{1}H NMR (D_2O, 500 MHz): δ 4.65 – 4.50 (ddd, 1H, *J* = 51.8, 10.3, 8.9 Hz, H-1), 3.93-3.91 (m, 2H, H-6), 3.88 (ddd, 1H, *J* = 13.4, 11.2, 8.9 Hz, H-5a), 3.62 – 3.53 (m, 2H, H-3, H-4), 3.37 (pq, 1H, *J* = 10.1 Hz, H-2), 1.58 – 1.52 (m, 1H, H-5). ^{13}C{^{1}H} NMR (D_2O, 151 MHz, ^{19}F-coupled): δ 93.1 (d, *J* = 180.8 Hz, C-1), 71.8 (d, *J* = 8.9 Hz, C-3), 69.1 (C-4), 67.4 (d, *J* = 17.8 Hz, C-5a), 55.9 (C-6), 54.0 (d, *J* = 17.1 Hz, C- 2), 45.3 (d, *J* = 7.2 Hz, C-5); ^{13}C{^{1}H} NMR (D_2O, 126 MHz, ^{19}F-decoupled): δ 93.2 (C-1), 71.8 (C-3), 69.1 (C-4), 67.4 (C-5a), 55.9 (C-6), 54.0 (C-2), 45.3 (C-5); ^{19}F NMR (471 MHz, D_2O): δ -192.7 (dpt, *J* = 51.8, 11.7 Hz); HRMS (ESI) *m/z*: [*M*+H]$^+$ Calcd for $C_7H_{15}FNO_4$ 196.0980; Found 196.0979; HPLC (Phenomenex® Luna 5µ HILIC 200Å, AXIA Pa, 250 x 21.20 mm, MeCN/12 mM TEAB buffer pH = 7.00 = 30/70, flow rate = 8.0 mL/min, ELSD): t_R = 12.5 min.

(1*S*,2*S*,3*R*,4*R*,5*R*,6*R*)-*N*^{1},*N*^{1}-Dibenzyl-2,5,6-tris(benzyloxy)-4-((benzyloxy)methyl)-*N*^{3}-methylcyclohexane-1,3-diamine (42)

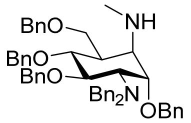

Compound **8**[2] (171 mg, 23.3µmol) was dissolved in anhydrous DCM (4 mL). TPAP (8 mg, 23 µmol) and NMO (54 mg, 466.0 µmol) were added, and the solution was stirred for 1 h

at room temperature. The reaction mixture was filtered over a short plug of silica which was washed with petroleum ether/EtOAc 1:1 (10 mL), and the solvent was removed under reduced pressure. The crude ketone was dissolved in a 1:1 mixture of anhydrous DCM and anhydrous isopropanol (4 mL) to which NEt$_3$ (193.1 µL, 1.39 mmol) was added. The reaction was stirred for 6 h. Methylammonium chloride (1.1 g, 16.26 µmol) was suspended in dry methanol (8 mL) and the reaction mixture containing ketone **7** was added. The mixture was stirred for 10 min and NaBH$_3$CN (24.8 mg, 394.8 µmol) was added. The reaction was stirred overnight. Saturated NaHCO$_3$ solution (10 mL) was added. The aqueous phase was extracted with DCM (3x 15 mL), and the crude product was purified by FC (petroleum ether/EtOAc = 10:1 – 2:1) to yield **42** (67 mg, 89.7 µmol, 39 % o3s starting from alcohol **8**). ^{1}H NMR (CDCl$_3$, 600 MHz): δ 7.51 – 7.06 (m, 30H, arenes), 5.08 (d, 1H, *J* = 11.7 Hz, O-C$\underline{H}$HPh), 4.94 (d, 1H *J* = 11.7 Hz, O-C$\underline{H}$HPh), 4.75 (d, 1H *J* = 10.7 Hz, O-C$\underline{H}$HPh), 4.63 – 4.56 (m, 2H, O-C$\underline{HH}$Ph), 4.54 (d, 1H, *J* = 10.7 Hz, O-C$\underline{H}$HPh), 4.46 (d, 1H, *J* = 12.0 Hz, O-C$\underline{H}$HPh), 4.43 (d, 1H, *J* = 12.0 Hz, O-C$\underline{H}$HPh), 4.18 (dd, 1H, *J* = 11.0, 8.5 Hz, H-3), 4.10 (d, 2H, *J* = 13.9 Hz, 2x N-C$\underline{H}$HPh), 4.00 (d, 2H, *J* = 13.9 Hz, N-C$\underline{HH}$Ph), 3.96 – 3.89 (m, 2H, H-4, H-1), 3.85 (dd, 1H *J* = 9.5, 6.0 Hz, 1x H-6), 3.59 (dd, 1H *J* = 9.5, 2.6 Hz, 1x H-6), 3.43 (dd, 1H, *J* = 11.0, 2.5 Hz, H-2), 2.92 (pt, 1H, *J* = 3.2 Hz, H-5a), 2.30 (ddpt, 1H, *J* = 11.6, 5.8, 2.8 Hz, H-5), 2.00 (s, 3H, CH$_3$); ^{13}C{^{1}H} NMR (CDCl$_3$, 151 MHz): δ 141.6, 140.0, 138.9, 138.6, 138.2 (C$_{quart.}$), 128.7, 128.6, 128.5, 128.4, 128.3, 128.2, 127.9, 127.8, 127.8, 127.6, 127.6, 127.1, 127.0, 126.5, (C$_{arenes}$) 82.3 (C-4), 81.0 (C-3), 80.9 (C-1), 74.8 (O-$\underline{C}$H$_2$Ph), 73.4 (O-$\underline{C}$H$_2$Ph), 72.9 (O-$\underline{C}$H$_2$Ph), 72.5 (O-$\underline{C}$H$_2$Ph), 69.9 (C-6), 60.3 (C-5a), 56.8 (2x N-$\underline{C}$H$_2$Ph), 40.9 (C-5), 34.5 (CH$_3$); HRMS (ESI) *m*/*z*: [*M*+H]$^+$ Calcd for C$_{50}$H$_{55}$N$_2$O$_4$ 747.4156; Found 747.4138.

(1*S*,2*S*,3*R*,4*R*,5*R*,6*R*)-N^1,N^1,N^3-Tribenzyl-2,5,6-tris(benzyloxy)-4-((benzyloxy)methyl)cyclohexane-1,3-diamine (43)

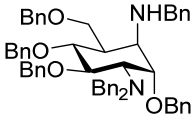

Compound **8**[2] (171 mg, 23.3µmol) was dissolved in dry DCM (4 mL). TPAP (8 mg, 23 µmol) and NMO (54 mg, 466.0 µmol) were added, and the solution was stirred for 1 h at room temperature. The reaction mixture was filtered over a short plug of silica which was washed with petroleum ether/EtOAc 1:1 (10 mL), and the solvent was removed under

reduced pressure. The crude ketone was dissolved in a 1:1 mixture of anhydrous DCM and dry isopropanol (4 mL) to which NEt_3 (193.1 µL, 1.39 mmol) was added. The reaction was stirred for 6 h. Benzylamine hydrochloride (2.3 g, 16.26 µmol) was suspended in dry methanol (8 mL) and the reaction mixture containing ketone **7** was added. The mixture was stirred for 10 min and $NaBH_3CN$ (24.8 mg, 394.8 µmol) was added. The reaction was stirred overnight. Sat. $NaHCO_3$ solution (10 mL) was added. The aqueous phase was extracted with DCM (3x 15 mL), and the crude product was purified by FC (petroleum ether/EtOAc = 10:1 – 3:1) to yield **43** (110 mg, 133.6 µmol, 58 % o3s starting from alcohol **8**). ^{1}H NMR ($CDCl_3$, 400 MHz): δ 7.56 – 7.10 (m, 33H, arenes), 6.99 – 6.89 (m, 2H, arenes), 5.11 (d, 1H, *J* = 11.7 Hz, O-C<u>H</u>HPh), 4.98 (d, 1H, *J* = 11.7 Hz, 1H, O-C<u>H</u>HPh), 4.78 (d, 1H, *J* = 10.8 Hz, O-C<u>H</u>HPh), 4.61 – 4.53 (m, 2H, 2x O-C<u>H</u>HPh), 4.50 (d, 1H, *J* = 11.1 Hz, O-C<u>H</u>HPh), 4.45 – 4.34 (m, 2H, 2x O-C<u>H</u>HPh), 4.21 (dd, 1H, *J* = 10.9, 8.5 Hz, H-3), 4.14 (d, 2H, *J* = 14.0 Hz, 2x N-C<u>H</u>HPh), 4.08 – 3.97 (m, 3H, 2x N-C<u>H</u>HPh, H-4), 3.97 – 3.93 (m, 1H, H-1), 3.94 – 3.88 (m, 1H, 1x H-6), 3.59 (dd, 1H, *J* = 10.9, 2.6 Hz, H-2), 3.55 (dd, 1H, *J* = 9.3, 2.3 Hz, 1x H-6), 3.45 (d, 1H, *J* = 12.6 Hz, 1x NH-C<u>H</u>HPh), 3.30 (d, 1H, *J* = 12.6 Hz, 1x NH-C<u>H</u>HPh), 3.10 (pt, 1H, *J* = 3.2 Hz, H-5a), 2.31 (ddpt, 1H, *J* = 11.3, 5.5, 2.6 Hz, H-5), 2.19 (s, 1H, N<u>H</u>); ^{13}C{^{1}H} NMR ($CDCl_3$, 101 MHz): δ 141.6, 140.6, 140.00, 139.1, 138.7, 138.0 ($C_{quart.}$), 128.7, 128.5, 128.5, 128.4, 128.4, 128.3, 128.2, 127.8, 127.7, 127.6, 127.2, 127.1, 126.9, 126.6 (C_{arenes}), 82.5 (C-3), 81.6 (C-1), 80.9 (C-4), 74.9 (O-<u>C</u>H_2Ph), 73.6 (O-<u>C</u>H_2Ph), 73.3 (O-<u>C</u>H_2Ph), 72.5 (O-<u>C</u>H_2Ph), 70.3 (C-6), 58.0 (C-5a), 57.1 (C-2), 56.6 (2x N-<u>C</u>H_2Ph), 52.4 (NH-CH_2Ph), 40.9 (C-5); HRMS (ESI) *m/z*: [*M*+H]$^+$ Calcd. for $C_{56}H_{59}N_2O_4$ 823.4469; Found 823.4465.

(1*R*,2*R*,3*S*,4*R*,5*R*,6*R*)-3-Amino-6-(hydroxymethyl)-5-(methylamino)cyclohexane-1,2,4-triol (44)

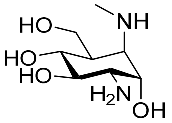

To a stirred solution of **42** (25.0 mg, 33.5 µmol) in dry MeOH (3 mL) were added Pd/C (10 % waterwet, 25.0 mg), $Pd(OH)_2$/C (20 % waterwet, 25.0 mg), and TFA (20.0 µL). The reaction was purged with hydrogen and stirred for 24 h under hydrogen atmosphere. The reaction mixture was centrifuged and the supernatant purified by HILIC-HPLC to give compound **44** (4.2 mg, 20.4 µmol, 61 %) as a white powder after lyophilization. ^{1}H NMR (D_2O, 600 MHz): δ 4.12 (pt, 1H, *J* = 3.2 Hz, H-1), 3.90 (dd, 1H, *J* = 11.5, 4.1 Hz, 1x H-6), 3.81

(dd, 1H, *J* = 11.5, 7.6 Hz, 1x H-6), 3.51 – 3.44 (m, 2H, H-3 & H-4), 2.99 (dd, 1H, *J* = 10.1, 3.2 Hz, H-2), 2.97 (pt, 1H, *J* = 3.5 Hz, H-5a), 2.36 (s, 3H, CH_3), 2.06 (ddpt, 1H, *J* = 11.2, 7.6, 3.8 Hz, H-5); $^{13}C\{^1H\}$ NMR (D_2O, 151 MHz): δ 74.2 (C-3), 70.2 (C-4), 67.6 (C-1), 61.6 (C-5a), 59.8 (C-6), 52.5 (C-2), 41.2 (C-5), 34.3 (CH_3); HRMS (ESI) *m*/*z*: $[M+H]^+$ Calcd for $C_8H_{19}N_2O_4$ 207.1339; Found 207.1342; HPLC (Phenomenex® Luna 5µ HILIC 200Å, AXIA Pa, 250 x 21.20 mm, MeCN/12 mM TEAB buffer pH = 7.00 = 40/60, flow rate = 8.0 mL/min, ELSD): t_R = 13.7 min.

(1*R*,2*R*,3*S*,4*S*,5*R*,6*R*)-3,5-Diamino-6-(hydroxymethyl)cyclohexane-1,2,4-triol (45)

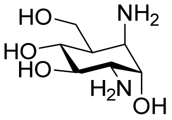

To a stirred solution of **43** (20.0 mg, 24.3 µmol) in dry MeOH (3 mL) was added Pd/C (10 % waterwet, 20.0 mg), $Pd(OH)_2$-C (20 % waterwet, 20.0 mg), and TFA (20.0 µL). The reaction was purged with hydrogen and stirred for 24h under hydrogen atmosphere. The reaction mixture was centrifuged and the supernatant purified by HILIC-HPLC to give compound **45** (3.1 mg, 16.1 µmol, 66 %) as a white powder after lyophilization. ^1H NMR (D_2O, 600 MHz): δ 4.00 (pt, 1H, *J* = 3.5 Hz, H-1), 3.93 (dd, 1H, *J* = 11.6, 4.7 Hz, 1x H-6), 3.76 (dd, 1H, *J* = 11.6, 8.6 Hz, 1x H-6), 3.62 – 3.58 (m, 1H, H-3), 3.58 – 3.54 (m, 1H, H-4), 3.34 (pt, *J* = 3.8 Hz, H-5a), 3.24 (dd, 1H *J* = 10.6, 3.3 Hz, H-2), 2.06 (ddpt, 1H, *J* = 11.5, 8.5, 4.1 Hz, H-5); $^{13}C\{^1H\}$ NMR (D_2O, 151 MHz): δ 72.7 (C-3), 70.6 (C-1), 70.0 (C-4), 59.5 (C-6), 52.7 (C-2), 51.7 (C-5a), 41.3 (C-5); HRMS (ESI) *m*/*z*: $[M+H]^+$ Calcd for $C_7H_{17}N_2O_4$ 193.1183; Found 193.1185; HPLC (Phenomenex® Luna 5µ HILIC 200Å, AXIA Pa, 250 x 21.20 mm, MeCN/12 mM TEAB buffer pH = 7.00 = 40/60, flow rate = 8.0 mL/min, ELSD): t_R = 12.2 min.

(1*S*,2*S*,3*S*,4*R*,5*R*,6*R*)-4,5-Bis(benzyloxy)-6-((benzyloxy)methyl)-3-(dibenzylamino)bicyclo[4.1.0]heptan-2-ol (46)

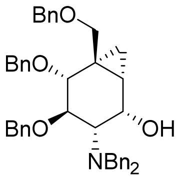

Alcohol **9**[2] (60 mg, 95.9 µmol) was dissolved in DCM (1.5 mL) and the solution was cooled to 0 °C. $ZnEt_2$ solution (958.8 µL, 958.8 µmol, 1 M in hexanes) was added to DCM (1.5 mL),

the solution was cooled to 0 °C and diiodomethane (61.9 µL, 767 µmol) was added to the solution. After 5 min, this solution was added to the solution containing alcohol **9**. The resulting mixture was allowed to warm to room temperature overnight. Water (2 mL) was added, and the aqueous layer was extracted with CH_2Cl_2 (3 x 2 mL). The combined organic layers were dried over $MgSO_4$, concentrated under reduced pressure, and the residue was purified by FC (petroleum ether / EtOAc = 5:1 – 3:1) to give compound **46** (42.0 mg, 65.6 µmol, 68 %) as a colourless oil. ^{1}H NMR ($CDCl_3$, 500 MHz): δ 7.42 – 7.15 (m, 25H, H-arenes), 5.00 (d, 1H, *J* = 11.6 Hz, O-C<u>H</u>HPh), 4.83 – 4.70 (m, 2H, 2x O-C<u>H</u>HPh), 4.67 (d, 1H *J* = 11.2 Hz, O-C<u>H</u>HPh), 4.39 – 4.30 (m, 2H, 2x O-C<u>H</u>HPh), 4.24 (dd, 1H, *J* = 7.8, 5.0 Hz, H-1), 4.16 (d, 1H, *J* = 7.2 Hz, H-4), 4.00 (d, 2H, *J* = 14.0 Hz, 2x N-C<u>H</u>HPh), 3.91 (d, 2H, *J* = 14.0 Hz, 2x N-C<u>H</u>HPh), 3.81 (dd, 1H, *J* = 10.4, 7.2 Hz, H-3), 3.72 (d, 1H, *J* = 10.4 Hz, 1x H-6), 2.81 (d, 1H, *J* = 10.4 Hz, 1x H-6), 2.78 (dd, 1H, *J* = 10.4, 5.0 Hz, H-2), 2.57 (s, 1H, OH), 1.24 (ddd, 1H, *J* = 9.5, 7.8, 5.5 Hz, H-5a), 0.97 (pt, 1H, *J* = 5.5 Hz, 1x $CH_{cyclopropan}$), 0.47 (dd, 1H, *J* = 9.4, 5.5 Hz, 1x $CH_{cyclopropan}$); $^{13}C\{^{1}H\}$ NMR ($CDCl_3$, 126 MHz): δ 140.7, 139.2, 138.6, 138.3 ($C_{quart.\ arenes}$), 128.6, 128.5, 128.4, 127.9, 127.7, 127.7, 127.5, 127.3, 126.9 (C_{arenes}), 81.8 (C-4), 78.5(C-3), 74.6 (C-6), 74.2 (O-<u>C</u>H2Ph), 73.1 (O-<u>C</u>H2Ph), 72.6 (O-<u>C</u>H2Ph), 68.6 (C-1), 62.9 (C-2), 57.1 (2x N-<u>C</u>H2Ph), 27.2(C-5), 24.4 (C-5a), 8.5 (CH_2cyclopropan); HRMS (ESI) *m/z*: $[M+H]^+$ Calcd for $C_{43}H_{46}NO_4$ 640.3421; Found 640.3416.

(1*R*,2*R*,3*R*,4*S*,5*S*,6*S*)-4-Amino-1-(hydroxymethyl)bicyclo[4.1.0]heptane-2,3,5-triol (47)

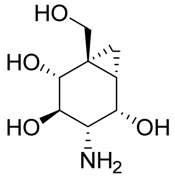

To a stirred solution of **46** (10.0 mg, 15.6 µmol) in dry MeOH (2 mL) were added Pd/C (10 % waterwet, 10.0 mg), $Pd(OH)_2/C$ (20 % waterwet, 10.0 mg), and TFA (10.0 µL). The reaction was purged with hydrogen and stirred for 12 h under hydrogen atmosphere. The reaction mixture was filtered over celite and the crude product purified by HILIC-HPLC to give compound **47** (2.40 mg, 12.7 µmol, 81 %) as a white powder after lyophilization. ^{1}H NMR (CD_3OD, 400 MHz): δ 4.45 (dd, 1H, *J* = 8.0, 4.7 Hz, H-1), 4.06 – 3.91 (m, 2H, H-4, 1x H-6), 3.41 (dd, 1H, *J* = 11.3, 8.5 Hz, H-3), 2.93 – 2.84 (m, 2H, H-2, 1x H-6), 1.47 (m, 1H, H-5a), 0.94 (pt, 1H *J* = 5.6 Hz, $CH_{cyclopropan}$), 0.53 (dd, 1H, *J* = 6.6, 5.6 Hz, $CH_{cyclopropan}$); $^{13}C\{^{1}H\}$ NMR

(CD_3OD, 126 MHz): δ 73.9 (C-4), 68.7 (C-3), 67.0 (C-6), 64.2 (C-1), 56.5 (C-2), 32.3 (C-5), 25.0 (C-5a), 9.6 (CH_2); HRMS (ESI) *m*/*z*: $[M+H]^+$ Calcd for $C_8H_{16}NO_4$ 190.1074; Found 190.1073; HPLC (Phenomenex® Luna 5μ HILIC 200Å, AXIA Pa, 250 x 21.20 mm, MeCN/12 mM TEAB buffer pH = 7.00 = 30/70, flow rate = 8.0 mL/min, ELSD): t_R = 10.2 min.

(2*S*,3*S*,4*R*,5*S*,6*S*)-4,5-bis(benzyloxy)-6-((benzyloxy)methyl)-2-(((1*S*,2*R*,3*R*,4*R*,5*R*,6*R*)-2,4,5-tris(benzyloxy)-6-((benzyloxy)methyl)-3-(dibenzylamino)cyclohexyl)oxy)tetrahydro-2*H*-pyran-3-yl acetate (49)

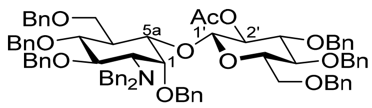

Carba-sugar **6**[2] (50.0 mg, 68.1 μmol) and fluoride **48**[3] (102 mg, 204.4 μmol) were dissolved in DCM (2 mL), and 4 Å molecular sieves were added. The mixture was cooled to 0 °C. Boron trifluoride diethyl etherate (20 μL, 136 μmol) was added. The reaction was allowed to reach room temperature and was stirred overnight. The reaction was stopped by addition of saturated $NaHCO_3$ solution (4 mL) and extracted with DCM (4x 3 mL). The combined organic layers were dried over $MgSO_4$ and the solvent was evaporated under reduced pressure. The crude product was purified by HPLC and product **49** was isolated as a colorless oil (38.0 mg, 31.4 μmol, 46 %). The α-product was also isolated (4 mg, 3.31 μmol, 5 %). ^1H NMR ($CDCl_3$, 500 MHz): δ 7.49 – 6.97 (m, 45H, H-arenes), 5.07 (dd, 1H, *J* = 9.5, 7.9 Hz, H-2'), 5.00 (d, 1H, *J* = 11.6 Hz, O-C<u>H</u>HPh), 4.91 (d, 1H, *J* = 10.2 Hz, O-C<u>H</u>HPh), 4.83 (m, 2H, O-C<u>H</u>HPh), 4.76 (d, 1H, *J* = 10.7 Hz, O-C<u>H</u>HPh), 4.70 (d, 1H, *J* = 10.4 Hz, O-C<u>H</u>HPh), 4.64 (d, 1H, *J* = 11.4 Hz, O-C<u>H</u>HPh), 4.59 – 4.53 (m, 3H, O-C<u>H</u>HPh, H-1'), 4.48 (m, 2H, O-C<u>H</u>HPh), 4.35 (d, 1H, *J* = 11.9 Hz, O-C<u>H</u>HPh), 4.32 – 4.28 (m, 2H, O-C<u>H</u>HPh), 4.20 (dd, 1H, *J* = 10.9, 8.7 Hz, H-3), 4.08 (d, *J* = 1.9 Hz, 1H, H-1), 4.01 (s, 4H, N-C<u>H</u>HPh), 3.83 (m, 2H, 1x H-6, H-4'), 3.77 – 3.71 (m, 2H, 1x H-6, H-5a), 3.66 (m, 4H, 2x H-6', H-4,H-3'), 3.41 (dt, 1H, *J* = 9.8, 3.0 Hz, H-5'), 2.66 (dd, 1H, *J* = 10.9, 1.9 Hz, H-2), 2.36 – 2.26 (m, 1H, H-5), 1.67 (s, 3H, CH_3); $^{13}C\{^1H\}$ NMR ($CDCl_3$, 126 MHz): δ 169.6 (C=O), 141.0, 139.7, 139.0, 138.7, 138.3, 138.1, 138.0 ($C_{quart.\ arenes}$), 128.6, 128.5, 128.4, 128.3, 128.2, 128.0, 128.0, 127.9, 127.8, 127.7, 127.6, 127.5, 127.5, 127.2, 127.1, 126.7 (C_{arenes}), 99.6 (C-1'), 83.6 (C-3'), 80.9 (C-4), 80.8 (C-3), 79.52 (C-1), 78.2 (C-4'), 77.1 (C-5a), 75.3 (O-<u>C</u>H2Ph), 75.3 (O-<u>C</u>H2Ph), 75.2 (O-<u>C</u>H2Ph), 75.1 (C-5'), 74.6 (O-<u>C</u>H2Ph), 73.7 (C-2'), 73.4 (O-<u>C</u>H2Ph), 73.3 (O-<u>C</u>H2Ph), 73.0 (O-<u>C</u>H2Ph), 68.7 (C-6'), 64.5 (C-6), 59.3 (C-2), 56.2 (2x N-<u>C</u>H2Ph), 43.3 (C-5), 21.1

(CH_3); HRMS (ESI) *m*/*z*: [*M*+H]$^+$ Calcd for $C_{78}H_{82}NO_{11}$ 1208.5882; Found 1208.5856; HPLC (Kinetex® 5 µm C8 100Å, 250 x 21.2 mm, MeCN, 10 mL/min, λ = 254 nm): t_R = 10.4 min.

(2*S*,3*S*,4*S*,5*S*,6*S*)-4,5-Bis(benzyloxy)-6-((benzyloxy)methyl)-2-(((1*S*,2*R*,3*R*,4*R*,5*R*,6*R*)-2,4,5-tris(benzyloxy)-6-((benzyloxy)methyl)-3-(dibenzylamino)cyclohexyl)oxy)tetrahydro-2*H*-pyran-3-ol (50)

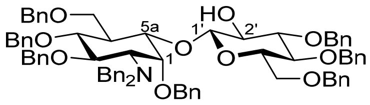

Compound **49** (10 mg, 8.27 µmol) was dissolved in MeOH (3 mL). K_2CO_3 (11.5 mg, 83 µmol) was added. The solution was stirred for 4 h. The solvent was removed under reduced pressure and the residue was distributed between DCM (3 mL) and water (3 mL). The organic phase was separated and dried over $MgSO_4$. The solvent was evaporated under reduced pressure. Crude compound **50** was obtained in quantitative yield and used without further purification. ^{1}H NMR ($CDCl_3$, 400 MHz): δ 7.50 – 6.97 (m, 45H, H-arenes), 5.15 (d, 1H, *J* = 11.1 Hz, O-C<u>H</u>HPh , 4.99 (d, 1H, *J* = 11.5 Hz, O-C<u>H</u>HPh), 4.88 – 4.81 (m, 4H, O-C<u>H</u>HPh), 4.74 – 4.66 (m, 2H, O-C<u>H</u>HPh), 4.61 – 4.52 (m, 2H), 4.50 – 4.43 (m, 3H, O-C<u>H</u>HPh, H-1’), 4.41 – 4.32 (m, 3H, O-C<u>H</u>HPh, H-1), 4.25 (dd, 1H, *J* = 11.0, 8.6 Hz, H-3), 4.07 (d, 2H, *J* = 14.6 Hz, N-C<u>H</u>HPh), 3.95 (d, 2H, *J* = 14.6 Hz, N-C<u>H</u>HPh), 3.86 – 3.54 (m, 9H, H-6, H-6’, H-5a, H-4, H-4’, H-3, H-2’), 3.43 (ddd, 1H, *J* = 9.8, 3.6, 2.1 Hz, H-5’), 2.75 (d,1H, *J* = 2.0 Hz, OH), 2.66 (dd, 1H, *J* = 11.0, 1.8 Hz, H-2), 2.36 (dd, 1H, *J* = 12.1, 10.0 Hz, H-5); ^{13}C{^{1}H} NMR ($CDCl_3$, 126 MHz): δ 141.0, 139.9, 139.6, 139.1, 138.6, 138.4 ($C_{quart. arenes}$), 128.6, 128.5, 128.5, 128.4, 128.4, 128.1, 128.0, 127.9, 127.8, 127.8, 127.7, 127.5, 127.4, 127.2, 126.8 (C_{arenes}), 102.0 (H-1’), 85.3, 80.8, 80.6, 79.4, 78.0, 75.7, 75.4, 75.3, 75.0, 75.0, 74.5, 73.8, 73.7, 73.5, 72.9, 69.4 (C-6’), 65.2 (C-6), 59.4 (C-2), 56.1 (2x N-<u>C</u>H_2Ph), 43.3 (C-5); HRMS (ESI) *m*/*z*: [*M*+H]$^+$ Calcd for $C_{76}H_{80}NO_{10}$ 1166.5777; Found 1166.5743.

(2*S*,3*S*,4*R*,5*R*,6*S*)-2-(((1*S*,2*R*,3*R*,4*R*,5*R*,6*R*)-3-Amino-2,4,5-trihydroxy-6-(hydroxymethyl)cyclohexyl)oxy)-6-(hydroxymethyl)tetrahydro-2H-pyran-3,4,5-triol (51)

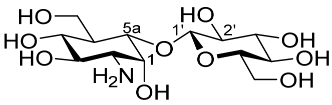

To a stirred solution of **50** (8.0 mg, 6.9 µmol) in dry MeOH (3 mL) was added Pd/C (10 % waterwet, 8.0 mg), $Pd(OH)_2/C$ (20 % waterwet,8.0 mg), and FA (10.0 µL). The reaction was purged with hydrogen and stirred for 24 h under hydrogen atmosphere. The reaction mixture was filtered over celite. The crude product was taken up in D_2O and the product **51** (1.0 mg, 4.8 µmol, 75 %) was obtained as a white powder as the formic acid salt after lyophilization. ^{1}H NMR (D_2O, 500 MHz): δ 8.47 (s, 1H, FA), 4.61 (d, 1H, *J* = 7.8 Hz, H-1'), 4.41 (pt, 1H, *J* = 2.7 Hz, H-1), 3.96-3.91 (m, 4H, 3x H-6, H-5a), 3.78 (dd, 1H, *J* = 10.8, 9.2 Hz, H-3), 3.73 (dd, 1H, *J* = 12.1, 6.6 Hz, 1x H-6'), 3.55 – 3.46 (m, 3H), 3.45 – 3.37 (m, 2H), 3.19 (dd, 1H, *J* = 10.8, 2.8 Hz, H-2), 1.94 (ptpt, 1H, *J* = 11.3, 2.4 Hz, H-5); ^{13}C{^{1}H} NMR (D_2O, 126 MHz): δ 171.1 (C=O, FA), 100.8 (C-1'), 75.9 (C-4), 75.8, 74.3 (C-5a), 72.9 (C-2'), 71.0, 69.8, 69.3, 66.7 (C-1), 60.9 (C-6), 56.3 (C-6), 53.4 (C-2), 42.7 (C-5); HRMS (ESI) *m*/*z*: $[M+H]^+$ Calcd for $C_{13}H_{26}NO_{10}$ 356.1551; Found 356.1547.

(1*R*,2*R*,3*R*,4*R*,5*S*,6*R*)-*N*,*N*-Dibenzyl-2,3,6-tris(benzyloxy)-4-((benzyloxy)methyl)-5-(((2*S*,3*R*,4*S*,5*R*,6*R*)-3,4,5-tris(benzyloxy)-6-((benzyloxy)methyl)tetrahydro-2H-pyran-2-yl)oxy)cyclohexan-1-amine (53)

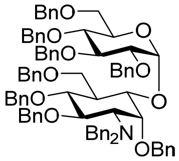

Carba-sugar **6**[2] (220.0 mg, 299.8 µmol) and fluoride **52**[4] (593 mg, 1.2 mmol) were dissolved in DCM (4 mL). 4 Å molecular sieves were added. The mixture was cooled to 0 °C. Boron trifluoride diethyl etherate (76 µL, 600 µmol) was added. The reaction was allowed to reach room temperature and was stirred overnight. The reaction was stopped by addition of sat. $NaHCO_3$ solution (6 mL) and extracted with DCM (4x 5 mL). The combined organic phases were dried over $MgSO_4$, and the solvent was evaporated under reduced pressure. The crude product was purified by HPLC. The product **53** was isolated as a colorless oil (148 mg, 123 µmol, 41 %). ^{1}H NMR ($CDCl_3$, 500 MHz): δ 7.54 – 6.99 (m, 50H, H-arenes), 5.22 (d, 1H, *J* = 10.5 Hz, O-C<u>H</u>HPh), 5.14 (d, 1H, *J* = 3.5 Hz, H-1'), 5.05 (d, 1H, *J* = 11.6 Hz, O-C<u>H</u>HPh), 4.93 (d, 2H *J* = 11.4 Hz, O-C<u>H</u>HPh), 4.88 (d, 1H, *J* = 10.9 Hz, O-C<u>H</u>HPh), 4.83 (d, 1H, *J* = 12.0 Hz, O-C<u>H</u>HPh), 4.79 (m, 3H, O-C<u>H</u>HPh), 4.72 (d, 1H, *J* = 12.4 Hz, O-C<u>H</u>HPh), 4.69 (d, 1H, *J* = 12.0 Hz, O-C<u>H</u>HPh), 4.61 – 4.56 (m, 2H, O-C<u>H</u>HPh), 4.49 (d, 1H, *J* = 10.7 Hz, O-C<u>H</u>HPh), 4.39 (d, 1H, *J* = 11.8 Hz, O-C<u>H</u>HPh), 4.32 – 4.26 (m, 2H, O-C<u>H</u>HPh,

H-3), 4.23 (pt, 1H, , *J* = 1.8 Hz, H-1), 4.13 – 4.01 (m, 6H, 4x N-C<u>H</u>HPh, H-3', H-5'), 3.97 (dd, 1H, *J* = 9.3, 2.1 Hz, 1xH-6), 3.89 (dd, 1H, *J* = 10.6, 3.0 Hz, 1x H-6'), 3.85 – 3.78 (m, 2H, 1x H-6, H-4'), 3.75 (dd, 1H, *J* = 11.4, 1.8 Hz, H-5a), 3.71 – 3.59 (m, 3H, 1x H-6', H-4, H-2'), 2.80 (dd, 1H, *J* = 10.9, 1.9 Hz, H-2), 2.53 (m, 1H, H-5); $^{13}C\{^1H\}$ NMR ($CDCl_3$, 126 MHz): δ 141.0, 139., 139.1, 138.9, 138.6, 138.6, 138.5, 138.1, 138.1, 128.6, 128.5, 128.5, 128.5, 128.4, 128.4, 128.3, 128.2, 128.1, 128.0, 128.0, 127.9, 127.8, 127.7, 127.6, 127.5, 127.5, 127.2, 127.0, 126.7, 98.5 (C-1'), 82.3 (C-5'), 82.1 (C-1), 80.8 (C-3), 80.6 (C-4), 80.1 (C-2'), 77.9 (C-4'), 76.4 (C-5a), 75.6, 75.1, 74.6, 73.8, 73.0, 72.0 (C-3'), 68.4 (C-6'), 64.4 (C-6), 59.3 (C-2), 56.1 (2x N-<u>C</u>H_2Ph), 43.6 (C-5); HRMS (ESI) *m/z*: $[M+H]^+$ Calcd for $C_{83}H_{86}NO_{10}$ 1256.6246; Found 1256.6222; HPLC (Kinetex® 5 µm C8 100Å, 250 x 21.2 mm, MeCN, 10 mL/min, λ = 254 nm): t_R = 13.2 min.

(2*S*,3*R*,4*S*,5*S*,6*R*)-2-(((1*S*,2*R*,3*R*,4*R*,5*R*,6*R*)-3-Amino-2,4,5-trihydroxy-6-(hydroxymethyl)cyclohexyl)oxy)-6-(hydroxymethyl)tetrahydro-2H-pyran-3,4,5-triol (54)

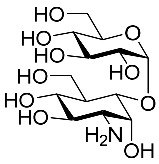

To a stirred solution of **53** (30.0 mg, 23.9 µmol) in dry MeOH (5 mL) was added Pd/C (10 % waterwet, 30.0 mg), $Pd(OH)_2$/C (20 % waterwet, 30.0 mg), and FA (30.0 µL). The reaction was purged with hydrogen and stirred for 24 h under hydrogen atmosphere. The reaction mixture was filtered over celite. The crude product was taken up in D_2O and the product **54** (7.4 mg, 20.8 µmol, 87 %) was obtained as a white powder as the formic acid salt after lyophilization. ^1H NMR (D_2O, 500 MHz): δ 5.14 (d, *J* = 4.0 Hz, 1H), 4.40 (pt, *J* = 2.8 Hz, 1H), 3.97 (m, 2H), 3.90 (dd, *J* = 12.0, 2.1 Hz, 1H), 3.86 – 3.69 (m, 5H), 3.61 (dd, *J* = 10.0, 4.0 Hz, 1H), 3.50 – 3.36 (m, 2H), 3.23 (dd, *J* = 10.9, 2.6 Hz, 1H), 2.03 (m, 1H); $^{13}C\{^1H\}$ NMR (D_2O, 126 MHz): δ 100.8, 76.7, 72.8, 72.6, 71.6, 70.7, 69.8, 69.5, 68.7, 60.8, 56.8, 53.9, 43.5; HRMS (ESI) *m/z*: $[M+H]^+$ Calcd for $C_{13}H_{26}NO_{10}$ 356.1551; Found 356.1553.

(1*R*,2*R*,3*R*,4*R*,5*R*,6*R*)-*N*,*N*-Dibenzyl-2,3,6-tris(benzyloxy)-4-((benzyloxy)methyl)-5-(((2*S*,3*R*,4*S*,5*R*,6*R*)-3,4,5-tris(benzyloxy)-6-((benzyloxy)methyl)tetrahydro-2H-pyran-2-yl)oxy)cyclohexan-1-amine (55)

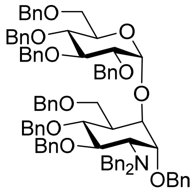

Carba-sugar **31** (200 mg, 273 µmol) and fluoride **52**[4] (539 mg, 1.09 mmol) were dissolved in DCM (4 mL). 4 Å molecular sieves were added. The mixture was cooled to 0 °C. Boron trifluoride diethyl etherate (69 µL, 545 µmol) was added. The reaction was allowed to reach room temperature and was stirred overnight. The reaction was stopped by addition of saturated $NaHCO_3$ solution (5 mL) and extracted with DCM (4x 5 mL). The combined organic phases were dried over $MgSO_4$, and the solvent was evaporated under reduced pressure. The crude product was purified by HPLC. Product **55** was isolated as a colorless oil (145 mg, 120 µmol, 44 %). ^{1}H NMR ($CDCl_3$, 500 MHz): δ 7.72 – 6.87 (m, 50H, H-arenes), 5.11 (d, 1H, *J* = 11.4 Hz, O-C<u>H</u>HPh), 5.01 (d, 1H, *J* = 11.4 Hz, O-C<u>H</u>HPh), 4.95 (d, 1H, *J* = 3.6 Hz, H-1'), 4.85 (d, 1H, *J* = 11.1 Hz, O-C<u>H</u>HPh), 4.79 (m, 2H, O-C<u>H</u>HPh), 4.68 (d, 1H, *J* = 10.9 Hz, O-C<u>H</u>HPh), 4.57 – 4.52 (m, 3H, O-C<u>H</u>HPh), 4.50 (d, *J* = 9.2 Hz, 1H), 4.47 (s, 1H), 4.45 (t, *J* = 5.4 Hz, 2H), 4.40 (d, *J* = 5.4 Hz, 1H), 4.38 (d, *J* = 5.7 Hz, 1H), 4.36 (t, *J* = 3.1 Hz, 1H), 4.32 – 4.25 (m, 2H, H-3,), 4.21 (d, *J* = 11.2 Hz, 1H), 4.15 (d, 2H, *J* = 14.8 Hz, 2x N-C<u>H</u>HPh), 4.09 (d, 2H, *J* = 14.8 Hz, 4x N-C<u>H</u>HPh), 3.94 (d, 1H, *J* = 11.1 Hz,), 3.84-3.77 (m, 2H, 1xH-6,), 3.80 – 3.77 (m, 1H), 3.76 – 3.66 (m, 2H), 3.64 – 3.55 (m, 2H. 1xH-6), 3.54 – 3.48 (m, 1H, 1x H-6',), 3.46 (m, 2H, 1x H-6', H-2'), 3.28 (dd, 1H, *J* = 10.8, 2.8 Hz, H-2), 2.63 (m, 1H, H-5); ^{13}C{^{1}H} NMR ($CDCl_3$, 126 MHz): δ 141.4, 139.7, 139.0, 138.8, 138.7, 138.6, 138.4, 138.3, 138.0, 128.5, 128.4, 128.4, 128.3, 128.3, 128.1, 128.0, 127.9, 127.8, 127.7, 127.7, 127.6, 127.6, 127.3, 127.3, 126.5, 94.1 (C-1'), 82.1, 81.6, 80.3, 78.6 (C-5a), 77.6, 75.5, 75.1, 75.0, 73.6, 73.5, 72.9, 72.8, 72.1, 70.7, 69.4, 68.7(C-6), 67.2 (C-6'), 59.2 (C-2), 56.9 (2x N-<u>C</u>H_2Ph), 41.7 (C-5); HRMS (ESI) *m*/*z*: [*M*+H]$^+$ Calcd for $C_{83}H_{86}NO_{10}$ 1256.6246; Found 1256.6227; HPLC (Kinetex® 5 µm C8 100Å, 250 x 21.2 mm, MeCN, 10 mL/min, λ = 254 nm): t_R = 12.7 min.

(2*S*,3*R*,4*S*,5*S*,6*R*)-2-(((1*R*,2*R*,3*R*,4*R*,5*R*,6*R*)-3-Amino-2,4,5-trihydroxy-6-(hydroxymethyl)cyclohexyl)oxy)-6-(hydroxymethyl)tetrahydro-2H-pyran-3,4,5-triol (56)

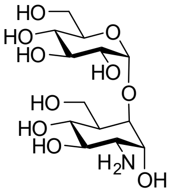

To a stirred solution of **55** (60.0 mg, 47.8 µmol) in dry MeOH (10 mL) was added Pd/C (10 % waterwet, 50.0 mg), $Pd(OH)_2/C$ (20 % waterwet, 50.0 mg), and FA (65.0 µL). The reaction was purged with hydrogen and stirred for 24 h under hydrogen atmosphere. The reaction mixture was filtered over celite. The crude product was taken up in D_2O and the product **56** (13.4 mg, 37.7 µmol, 79 %) was obtained as a white powder after lyophilization. ^{1}H NMR (D_2O, 500 MHz): δ 5.04 (d, *J* = 4.0 Hz, 1H), 4.34 (q, *J* = 5.0 Hz, 1H), 4.15 (t, *J* = 3.3 Hz, 1H), 4.00 (dd, *J* = 10.7, 4.9 Hz, 1H), 3.90 – 3.65 (m, 6H), 3.64 – 3.53 (m, 3H), 3.47 (d, *J* = 8.9 Hz, 1H), 2.19 (m, 1H); ^{13}C{^{1}H} NMR ($CDCl_3$, 126 MHz): δ 97.5, 74.2, 72.7, 72.6, 71.3, 71.0, 70.7, 69.5, 65.3, 60.4, 59.1, 53.3, 42.3; HRMS (ESI) *m*/*z*: $[M+H]^+$ Calcd for $C_{13}H_{26}NO_{10}$ 356.1551; Found: 356.1543.

3 Filter Disk Assay

B. subtilis wt168 cells were plated out from an overnight culture on Luria-Bertani (LB) Agar plates. Filters were saturated with 10 µL of different compound solutions. As a positive control, chloramphenicol (1 mg mL^{-1} = 9.3 mM) was used. The respective compounds were tested in concentrations of 100 mM. Filters were left to dry for 10 min at room temperature before placing them on the plates. Incubation was carried out at 30 °C overnight. Subsequently, the diameters of the inhibition zones were compared.

4 NMR Spectra

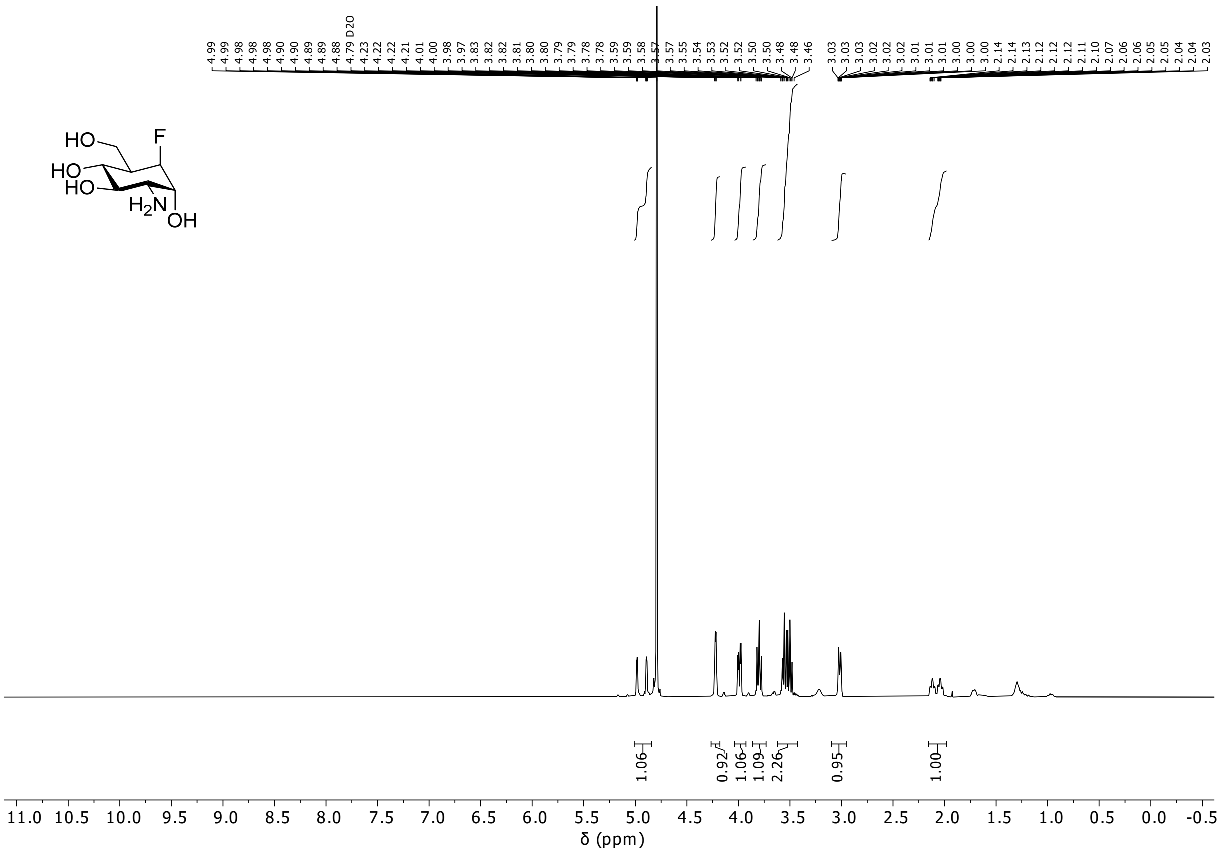

¹H NMR spectrum (500 MHz, D_2O) of **2**

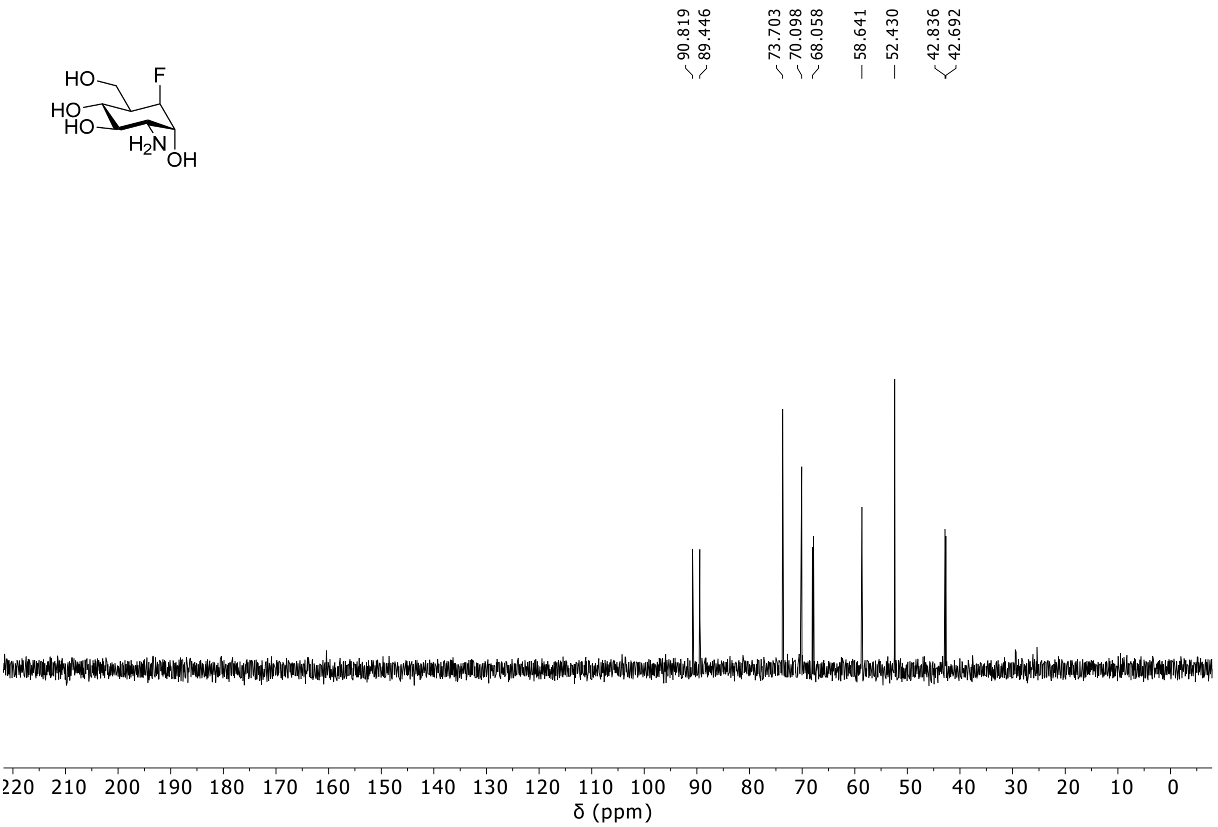

$^{13}C\{^1H\}$ NMR spectrum (126MHz, D_2O) of **2**

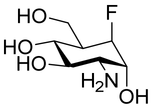

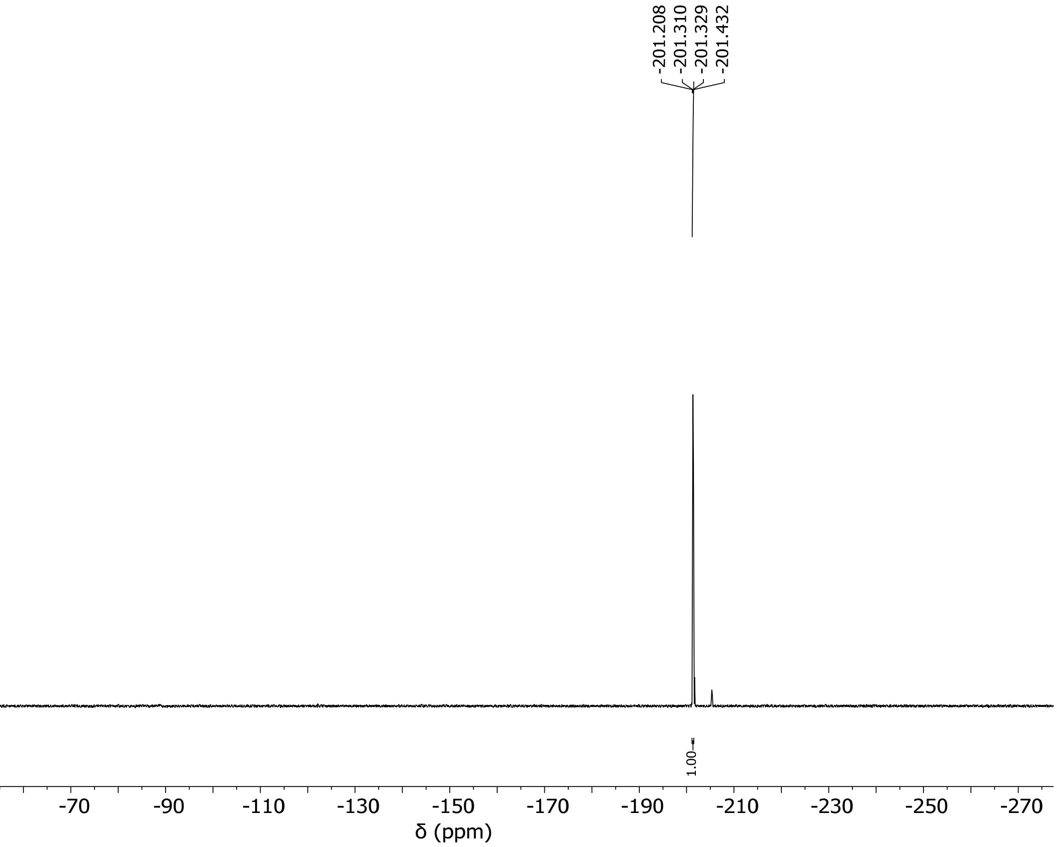

^{19}F NMR spectrum (376MHz, D_2O) of **2**

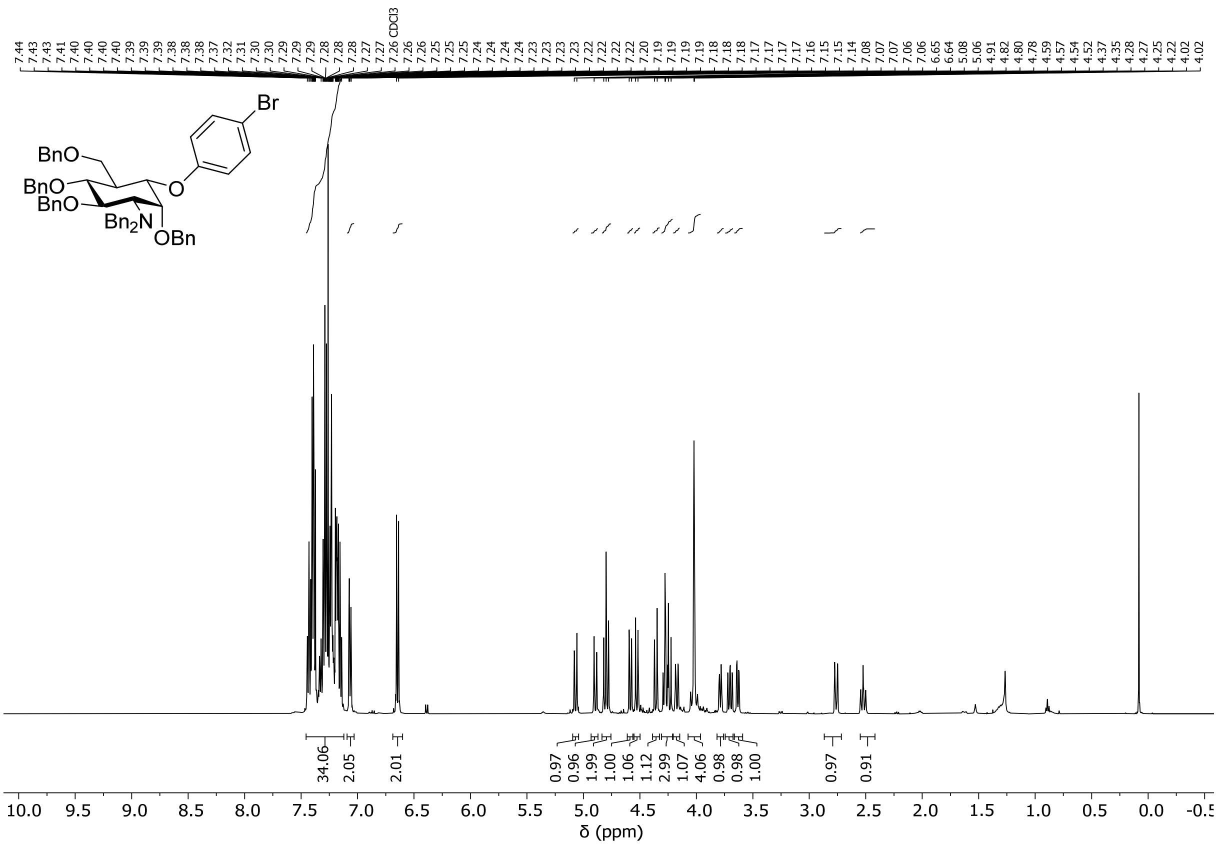

^{1}H NMR spectrum (500 MHz, $CDCl_3$) of **10**

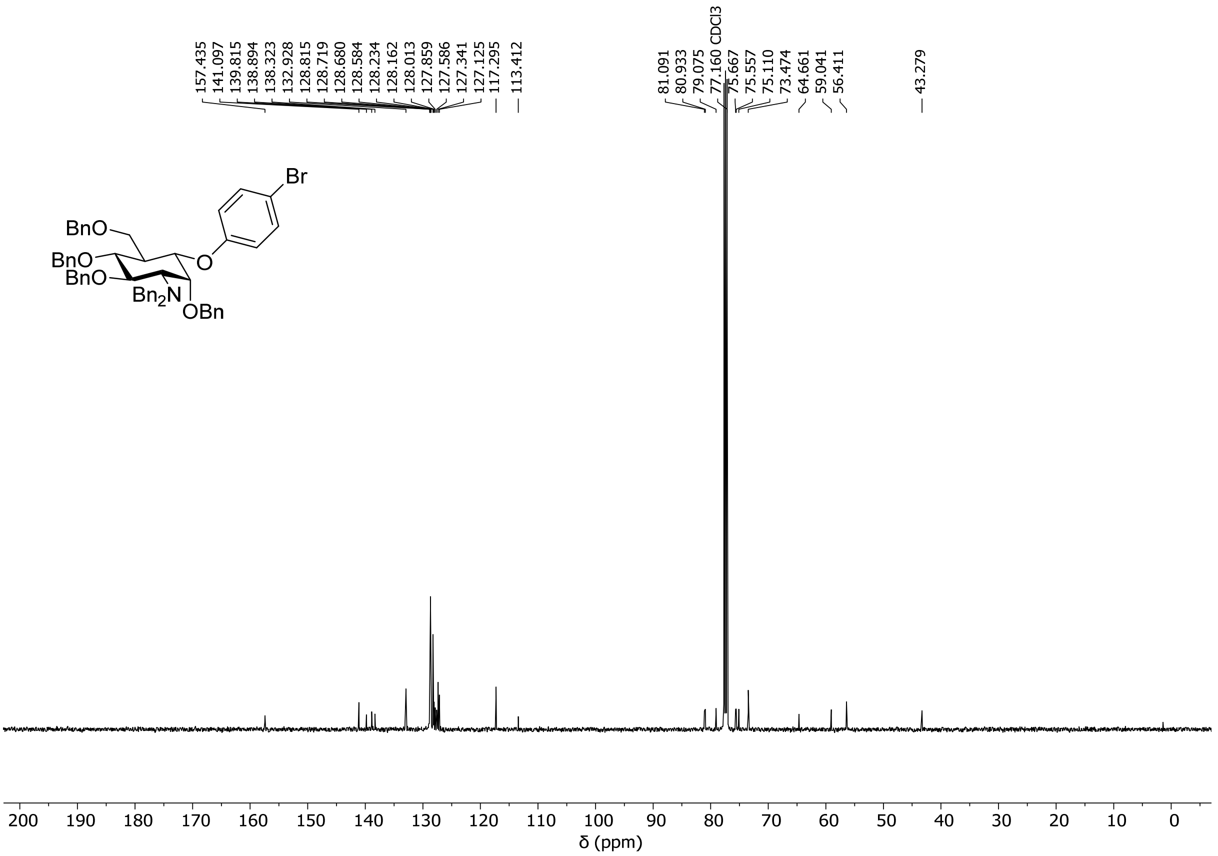

^{13}C{^{1}H} NMR spectrum (126 MHz, $CDCl_3$) of **10**

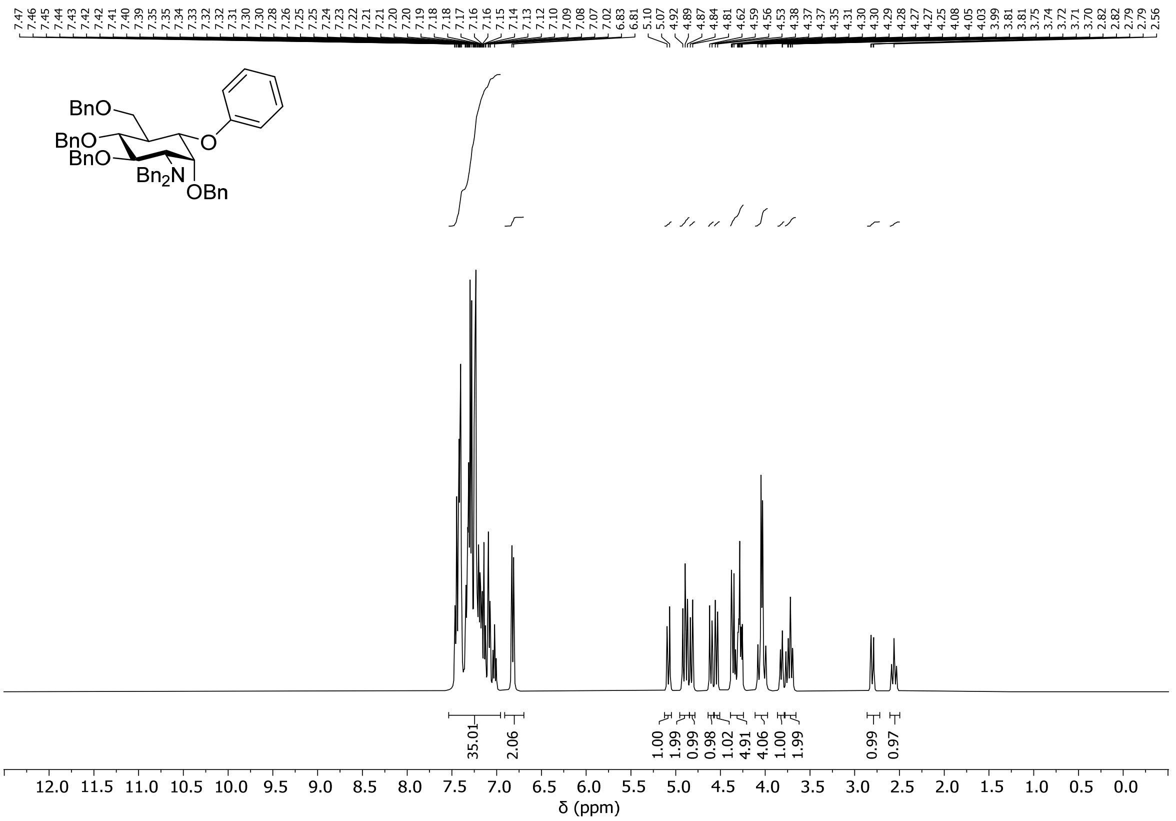

^{1}H NMR spectrum (400 MHz, $CDCl_3$) of **11**

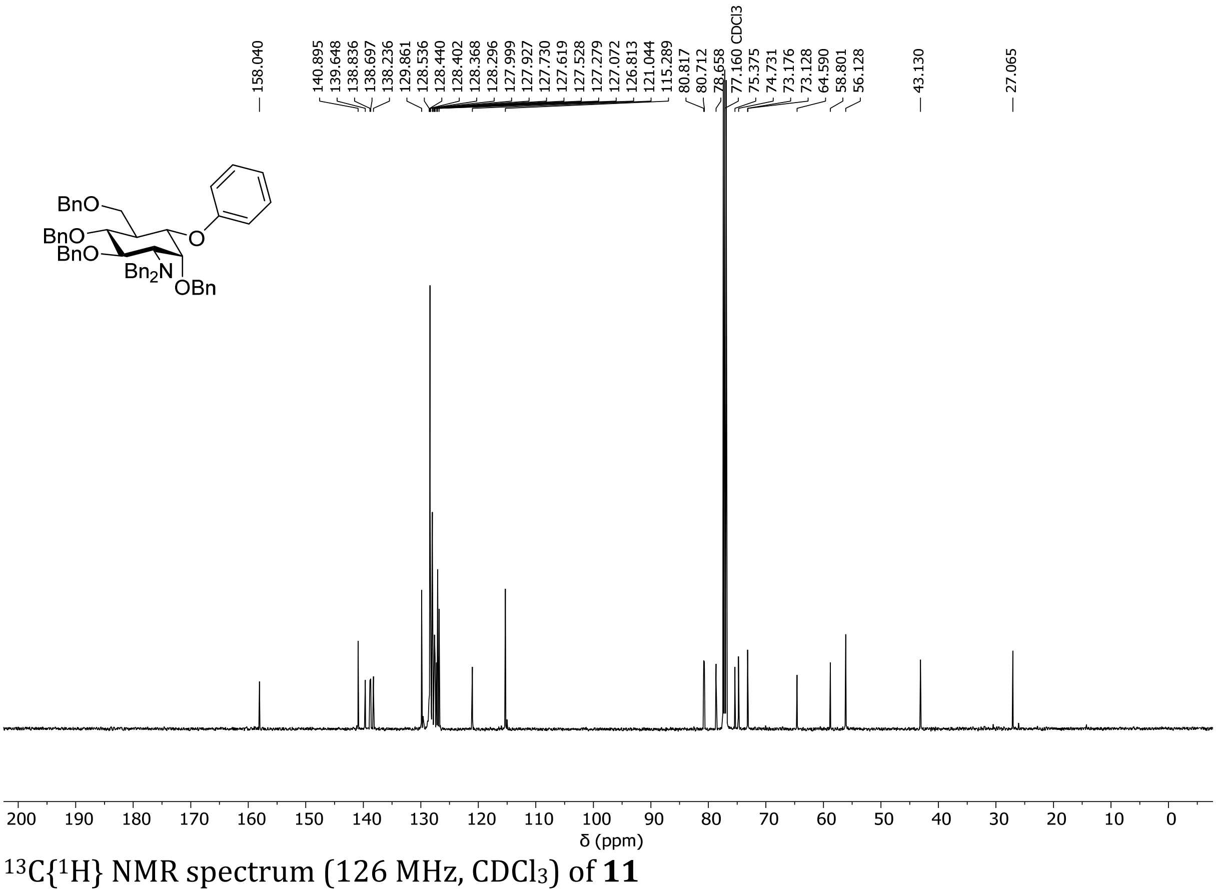

^{13}C{^{1}H} NMR spectrum (126 MHz, $CDCl_3$) of **11**

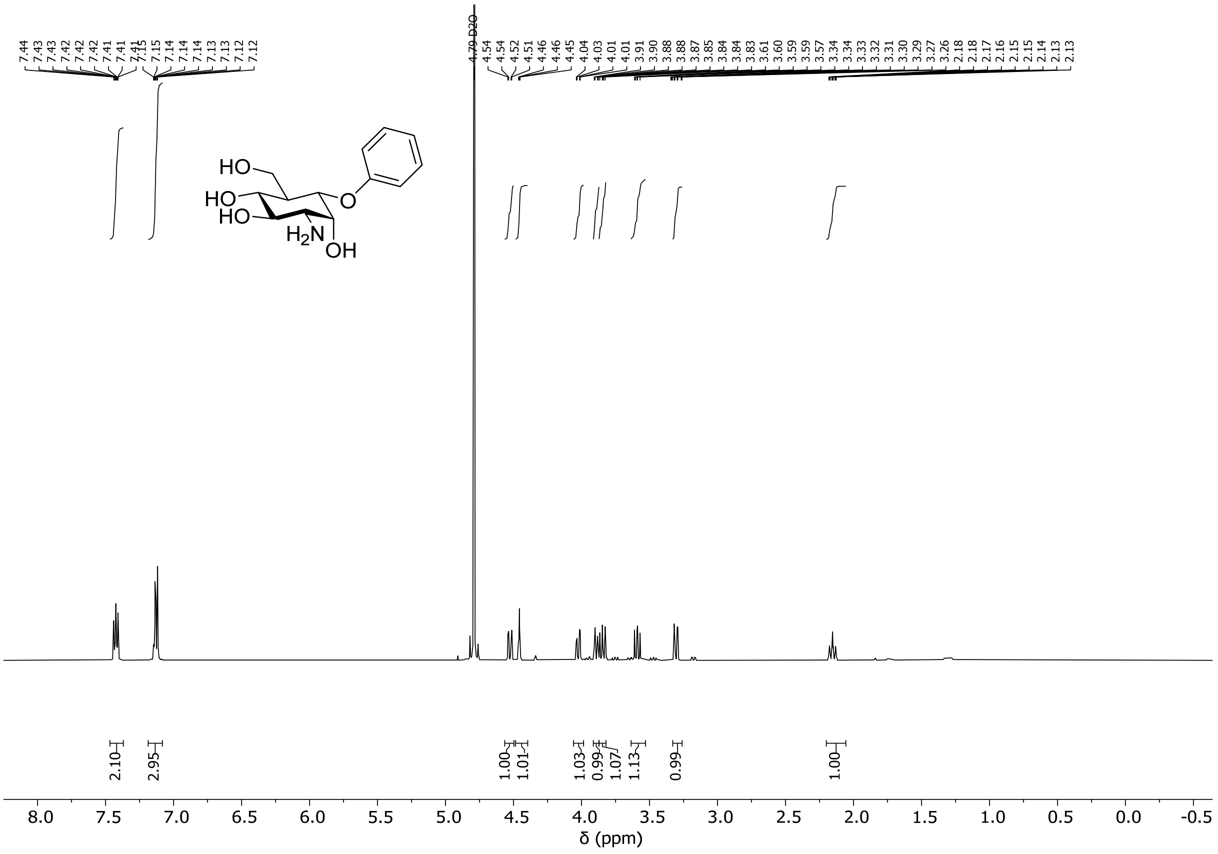

^{1}H NMR spectrum (500 MHz, D_2O) of **12**

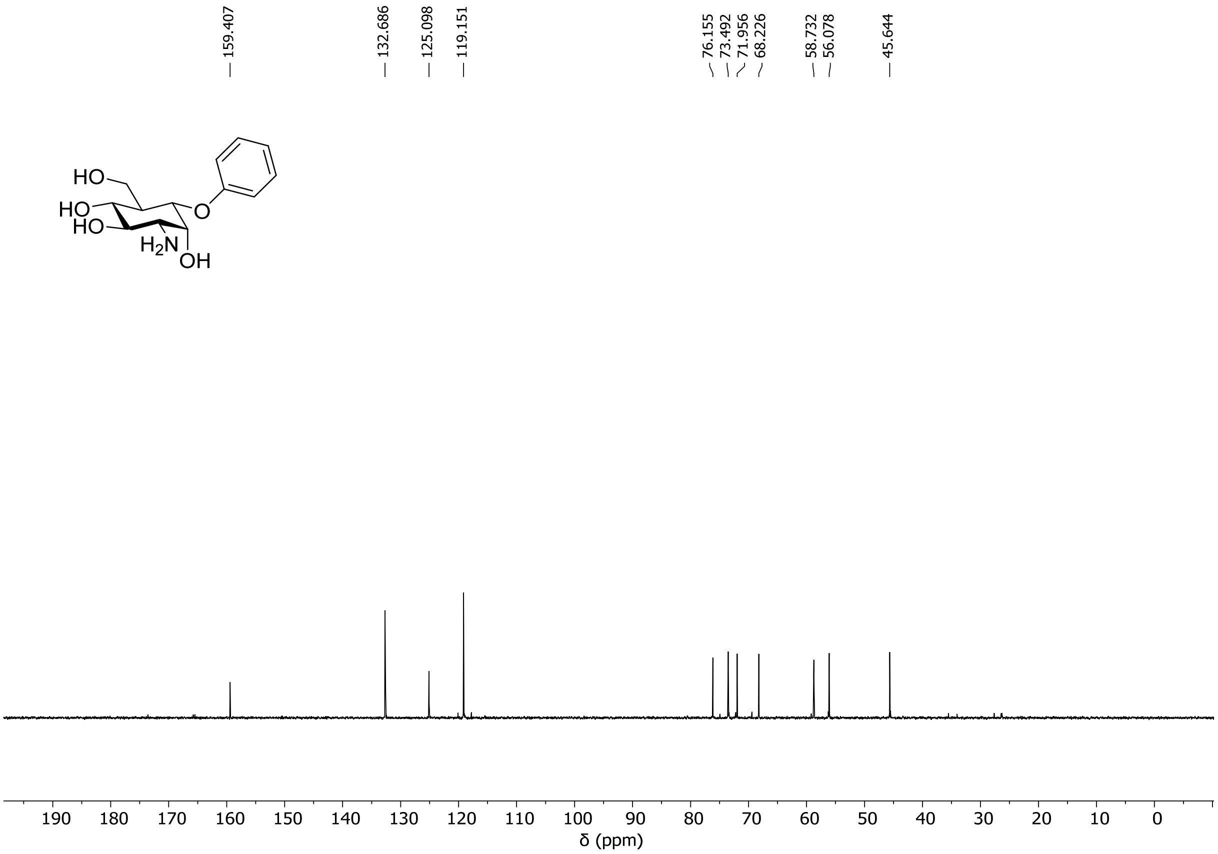

^{13}C{^{1}H} NMR spectrum (126 MHz, D_2O) of **12**

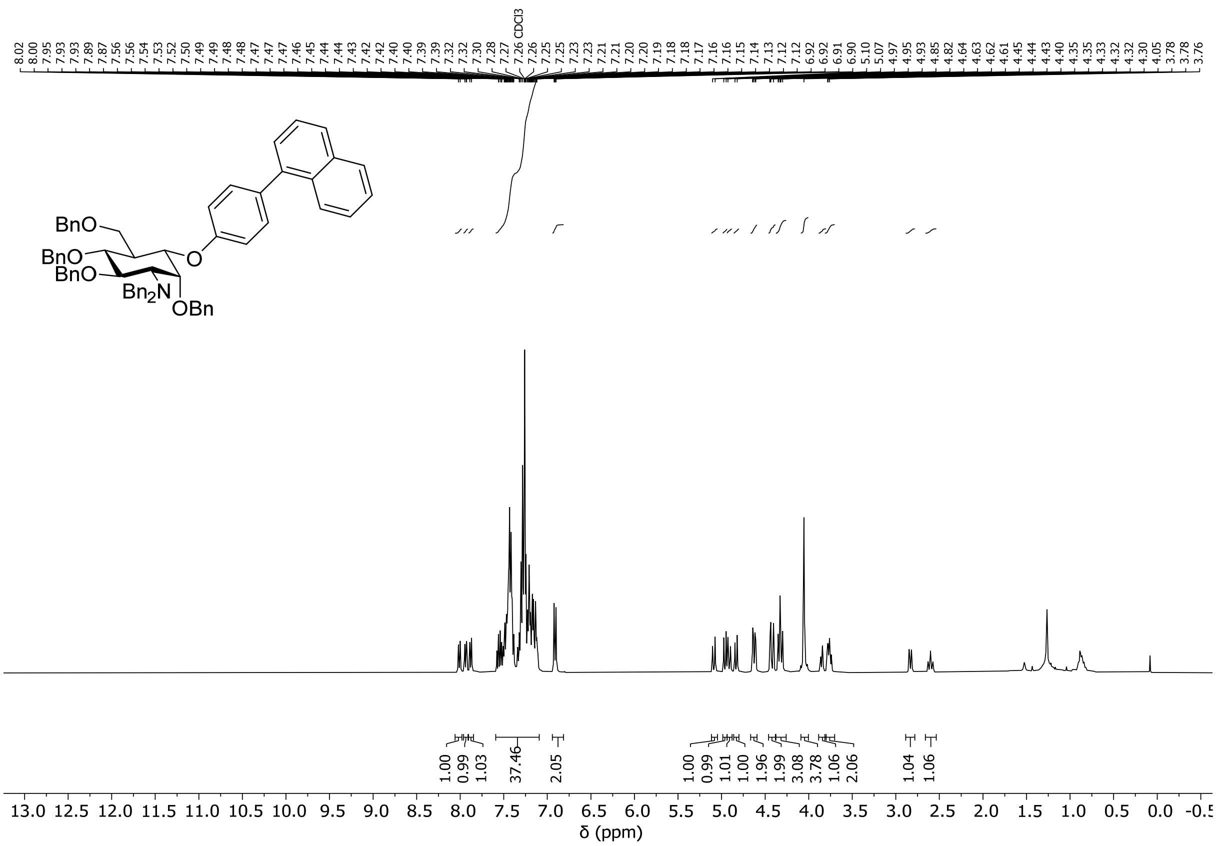

^{1}H NMR spectrum (400 MHz, $CDCl_3$) of **13**

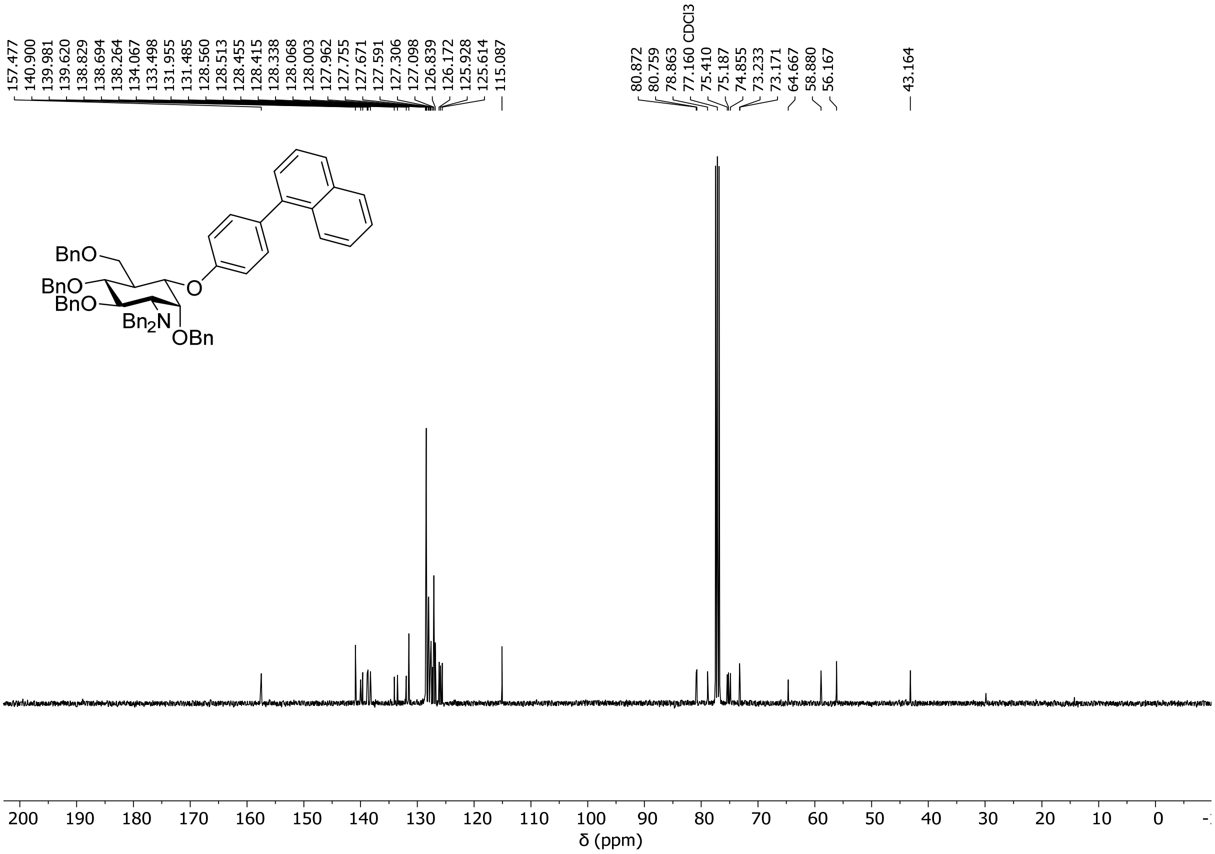

^{13}C{^{1}H} NMR spectrum (101 MHz, $CDCl_3$) of **13**

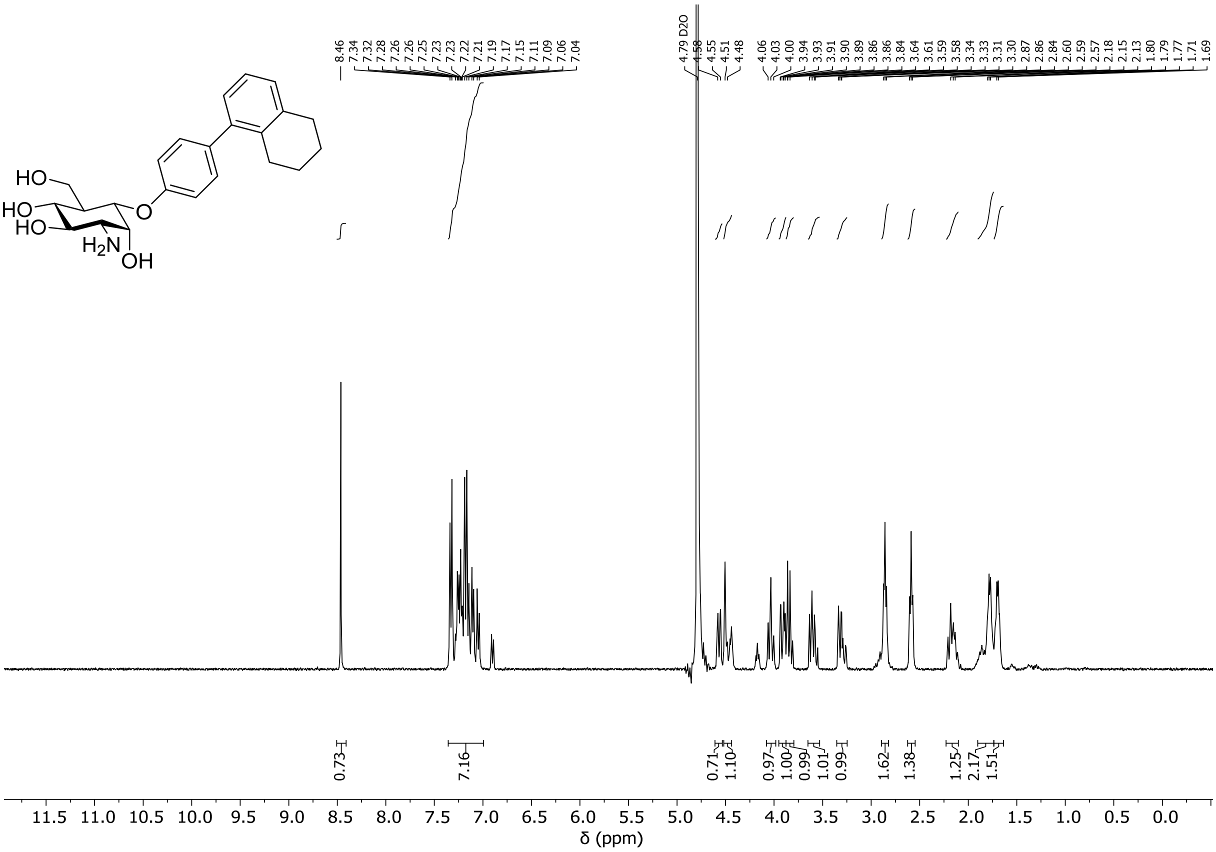

^{1}H NMR spectrum (400 MHz, D_2O) of **14**

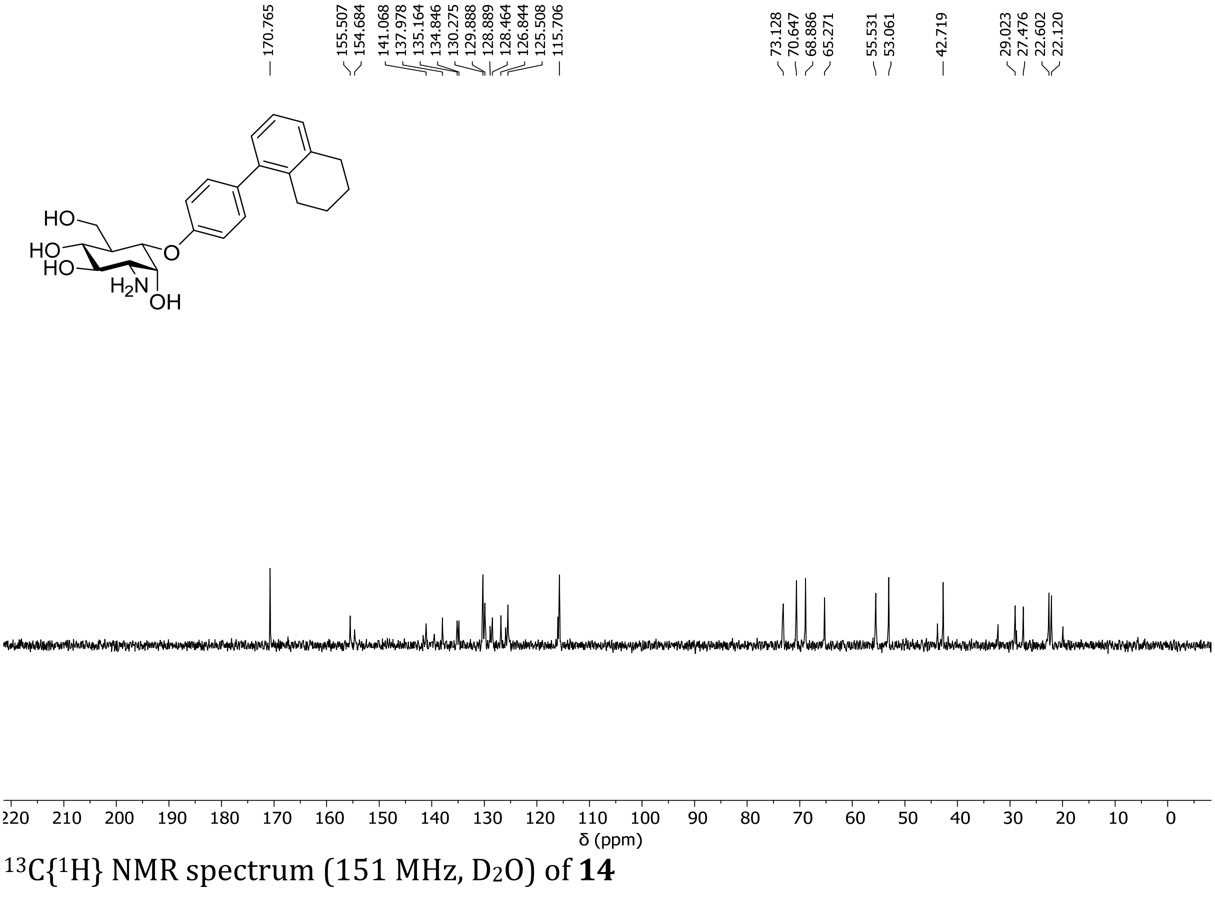

$^{13}C\{^{1}H\}$ NMR spectrum (151 MHz, D_2O) of **14**

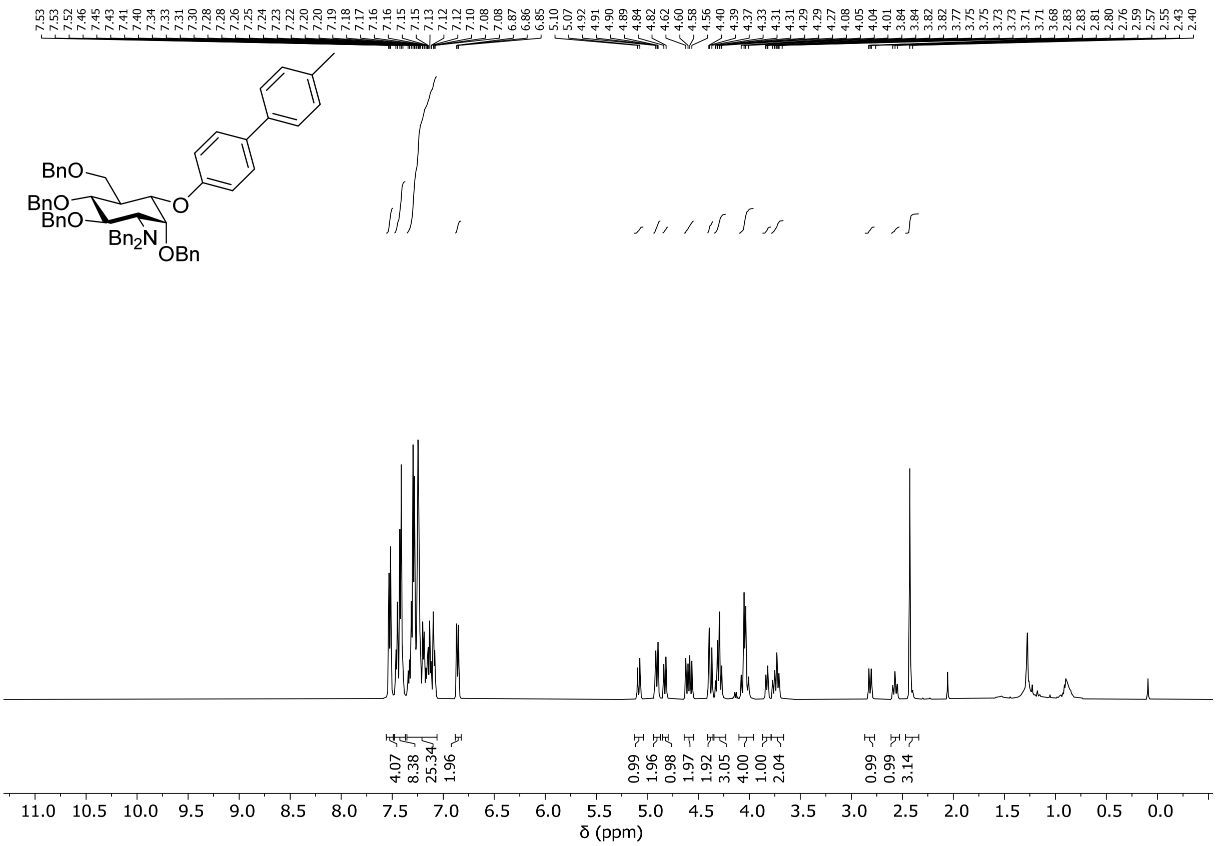

^{1}H NMR spectrum (500 MHz, $CDCl_3$) of **15**

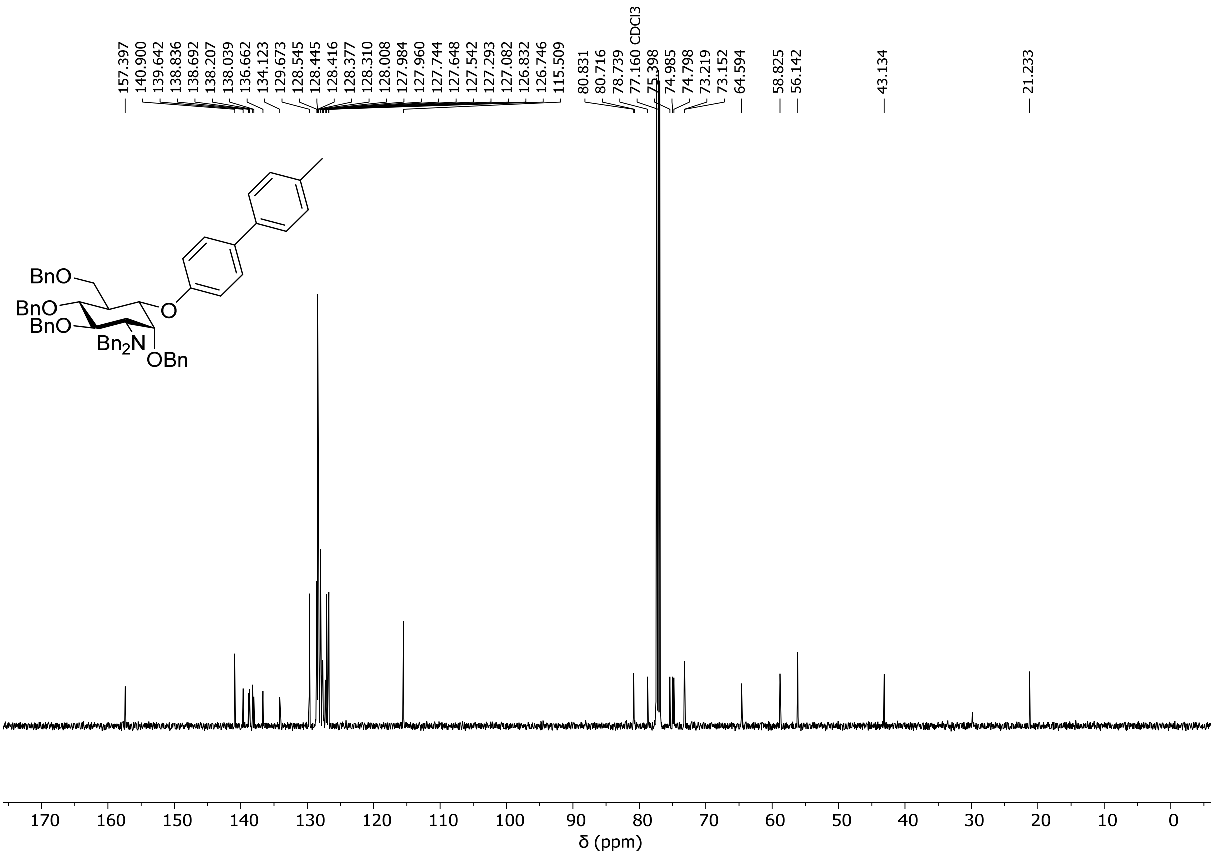

$^{13}C\{^{1}H\}$ NMR spectrum (126 MHz, $CDCl_3$) of **15**

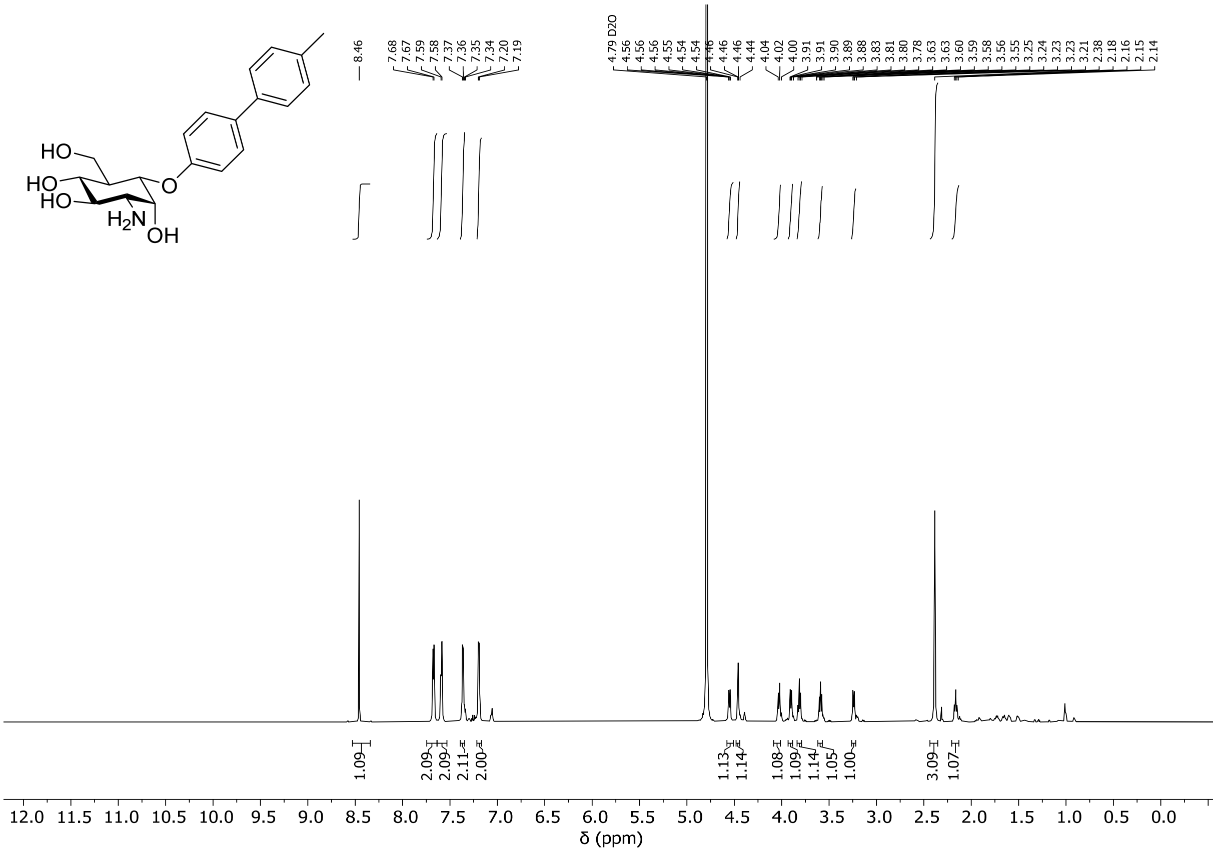

^{1}H NMR spectrum (800 MHz, D_2O) of **16**

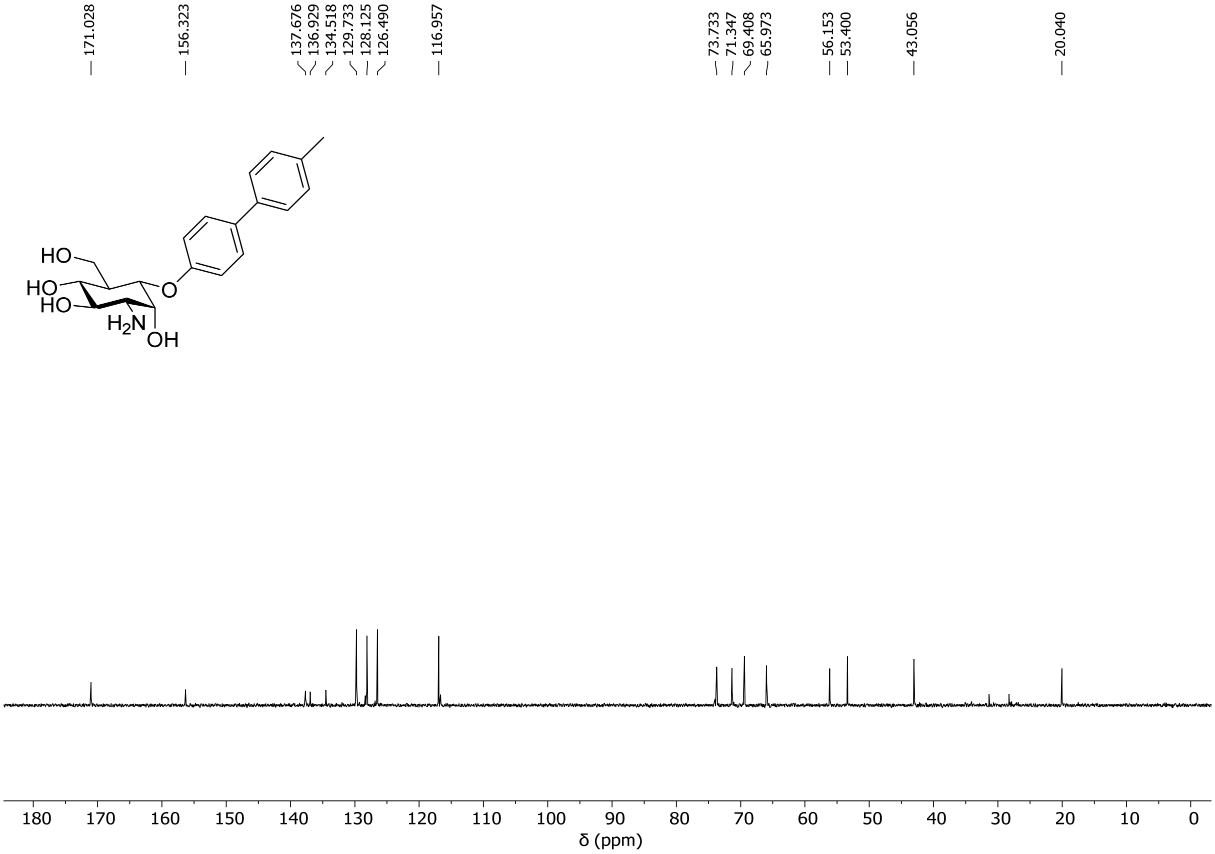

$^{13}C\{^{1}H\}$ NMR spectrum (201 MHz, D_2O) of **16**

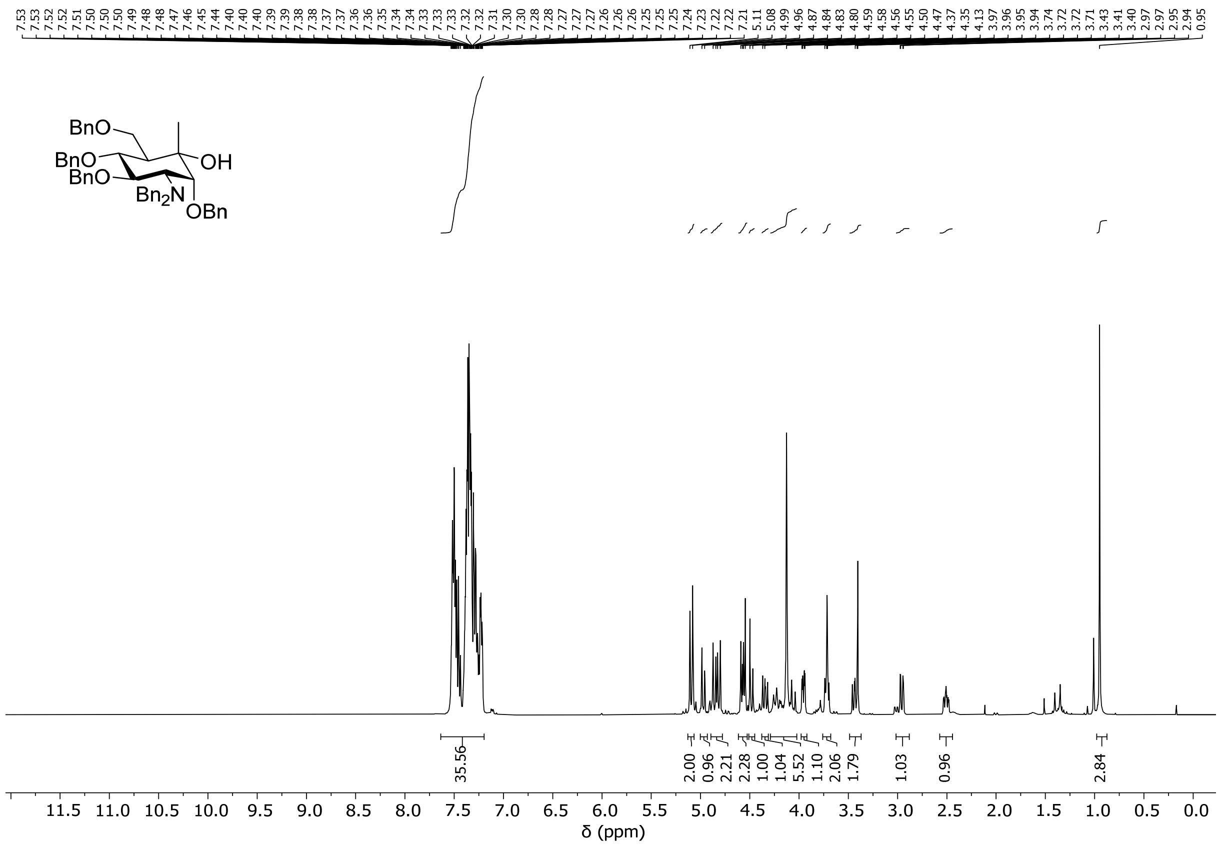

¹H NMR spectrum (400 MHz, CDCl₃) of **17**

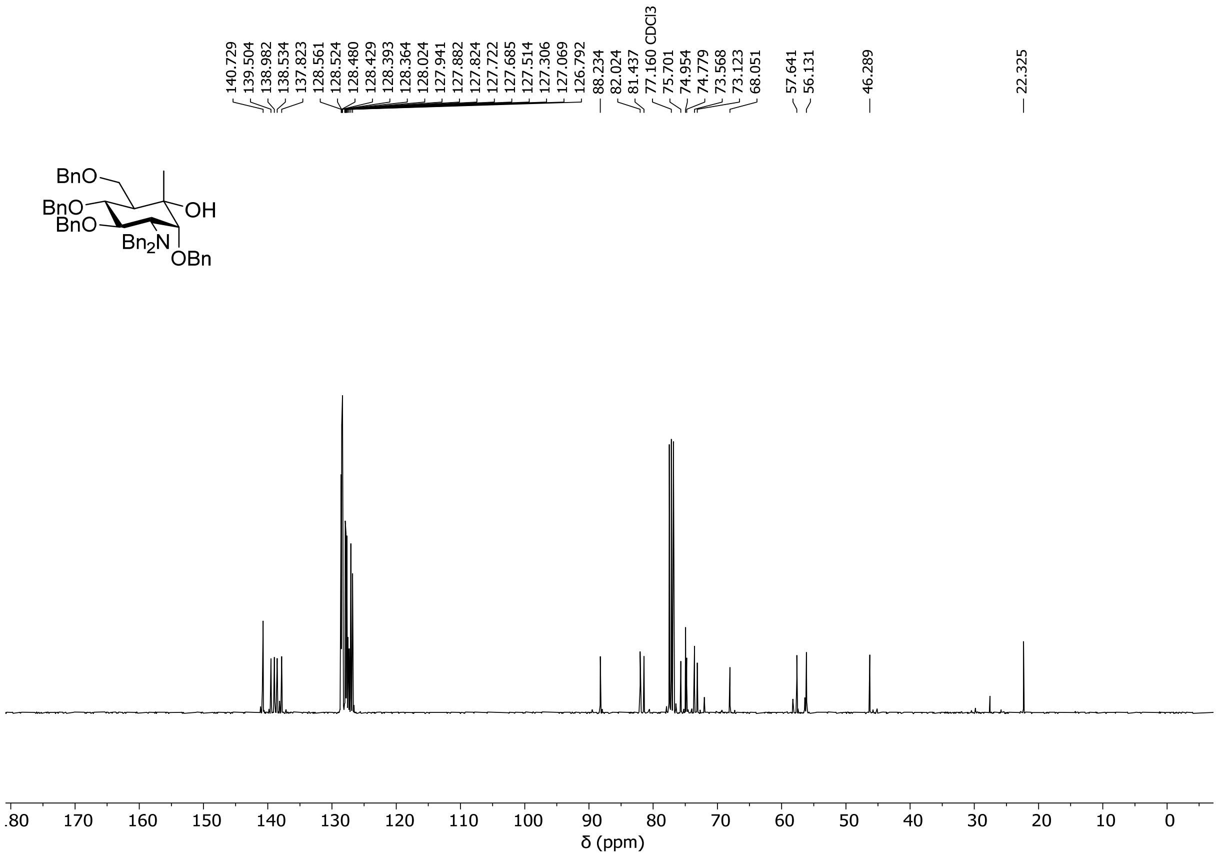

¹³C{¹H} NMR spectrum (101 MHz, CDCl₃) of **17**

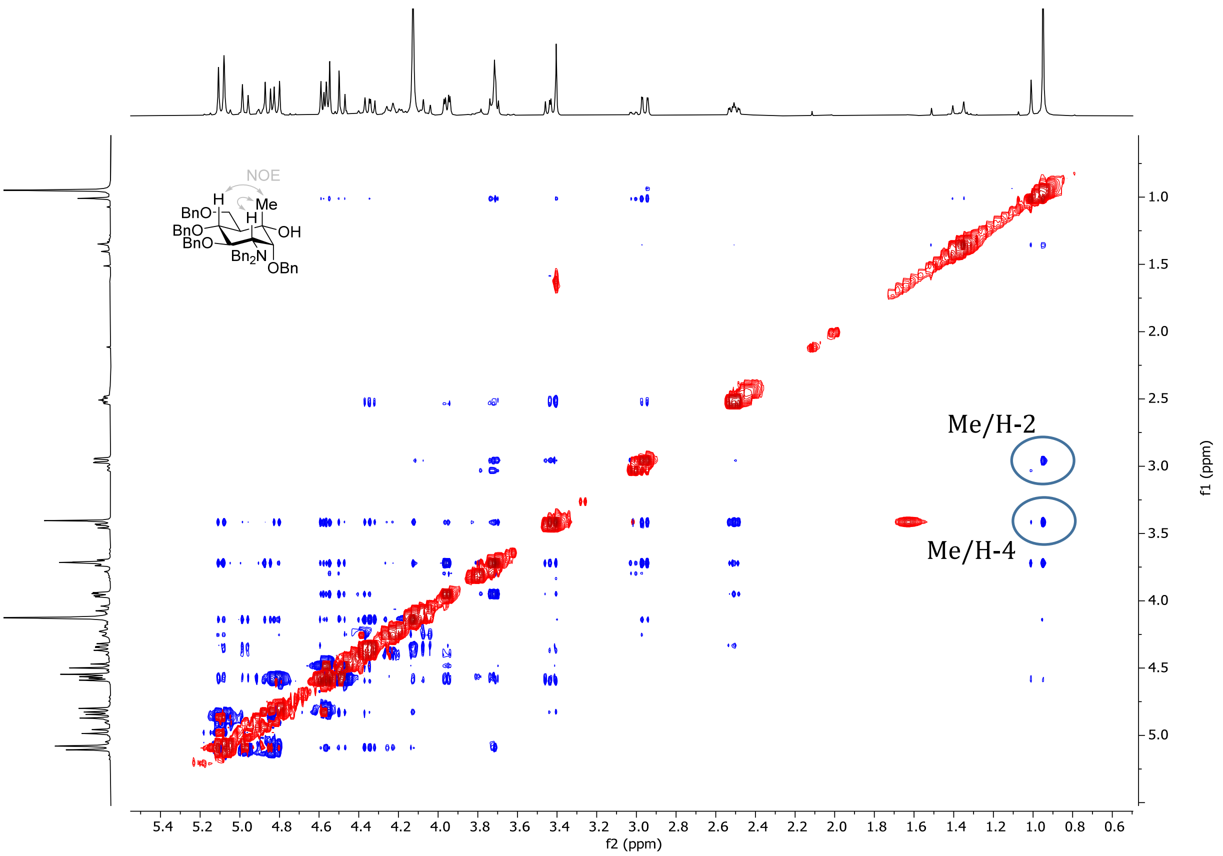

NOESY spectrum (400 MHz, $CDCl_3$) of **17**

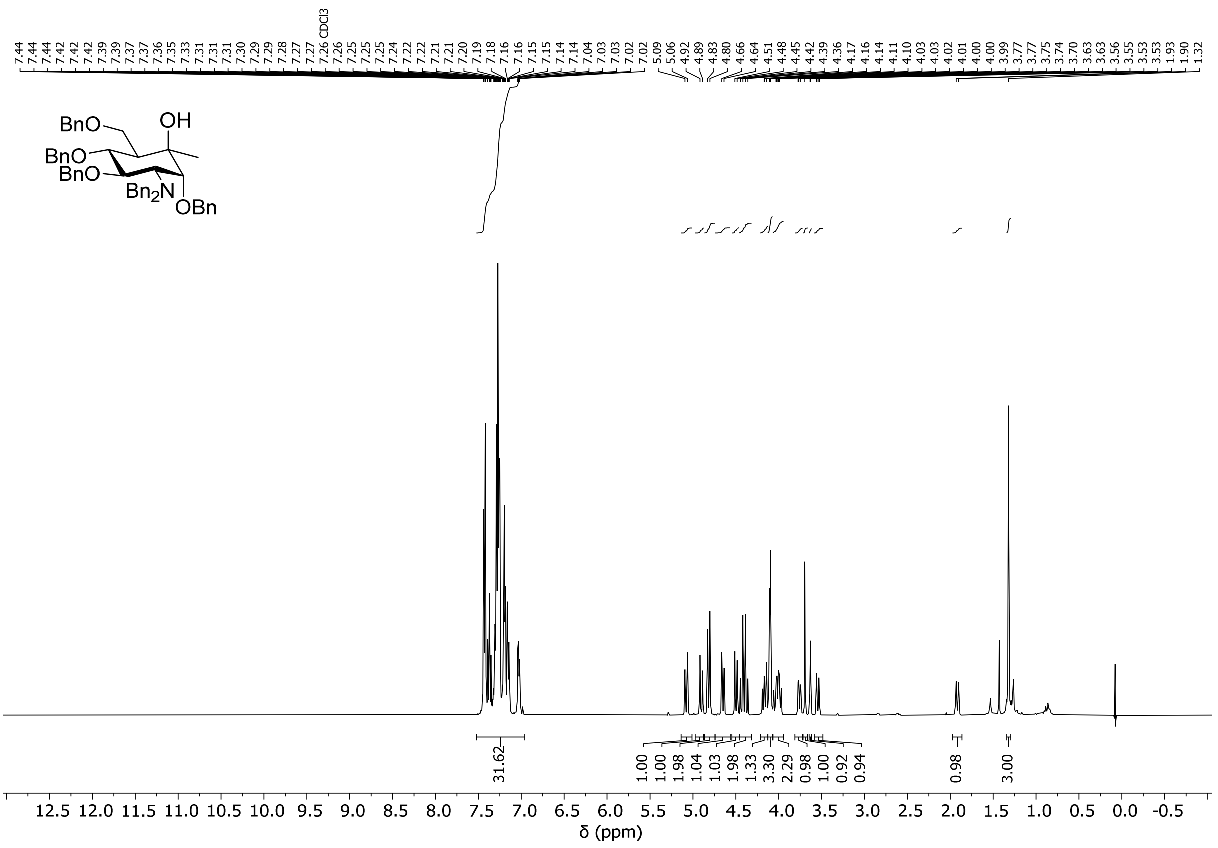

^{1}H NMR spectrum (400 MHz, $CDCl_3$) of **18**

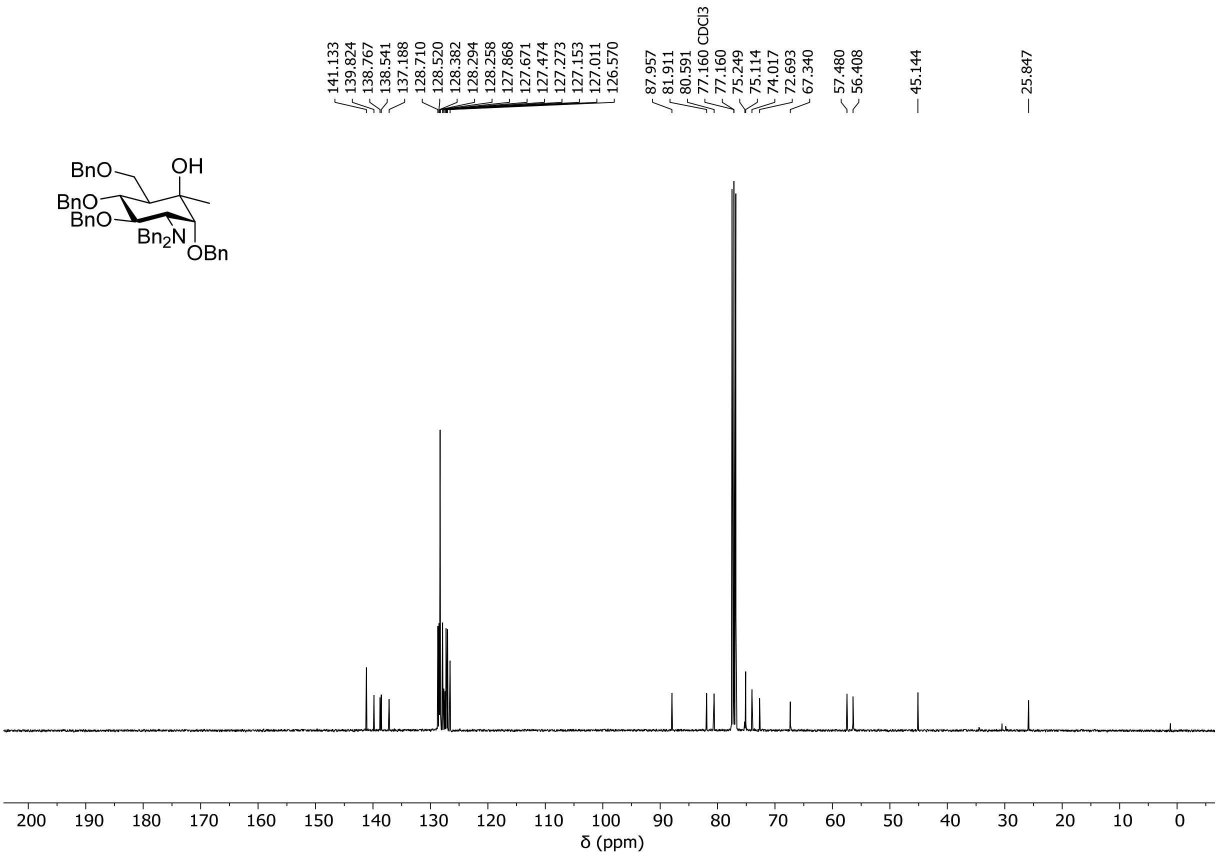

^{13}C{^{1}H} NMR spectrum (151 MHz, CDCl3) of **18**

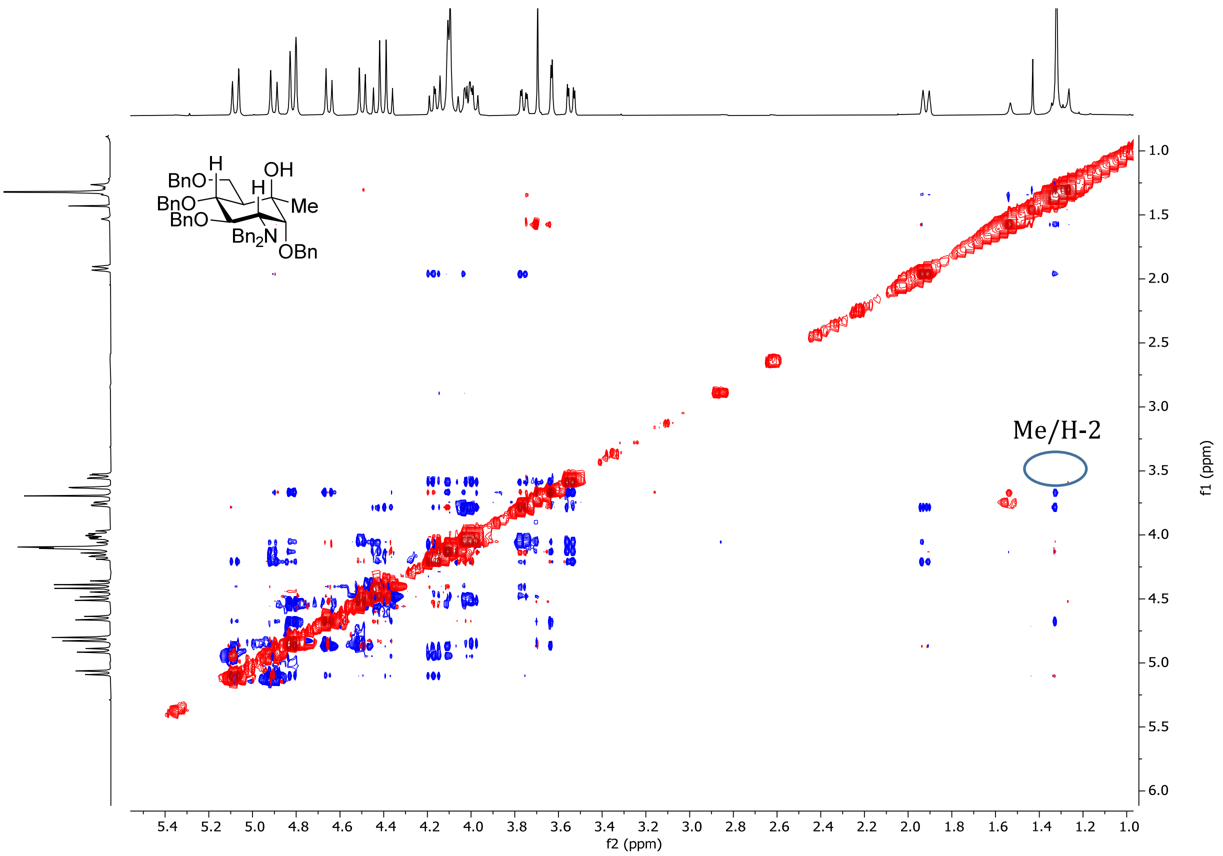

NOESY spectrum (400 MHz, CDCl3) of **18**. No cross peak is visible between the methyl group and H-2.

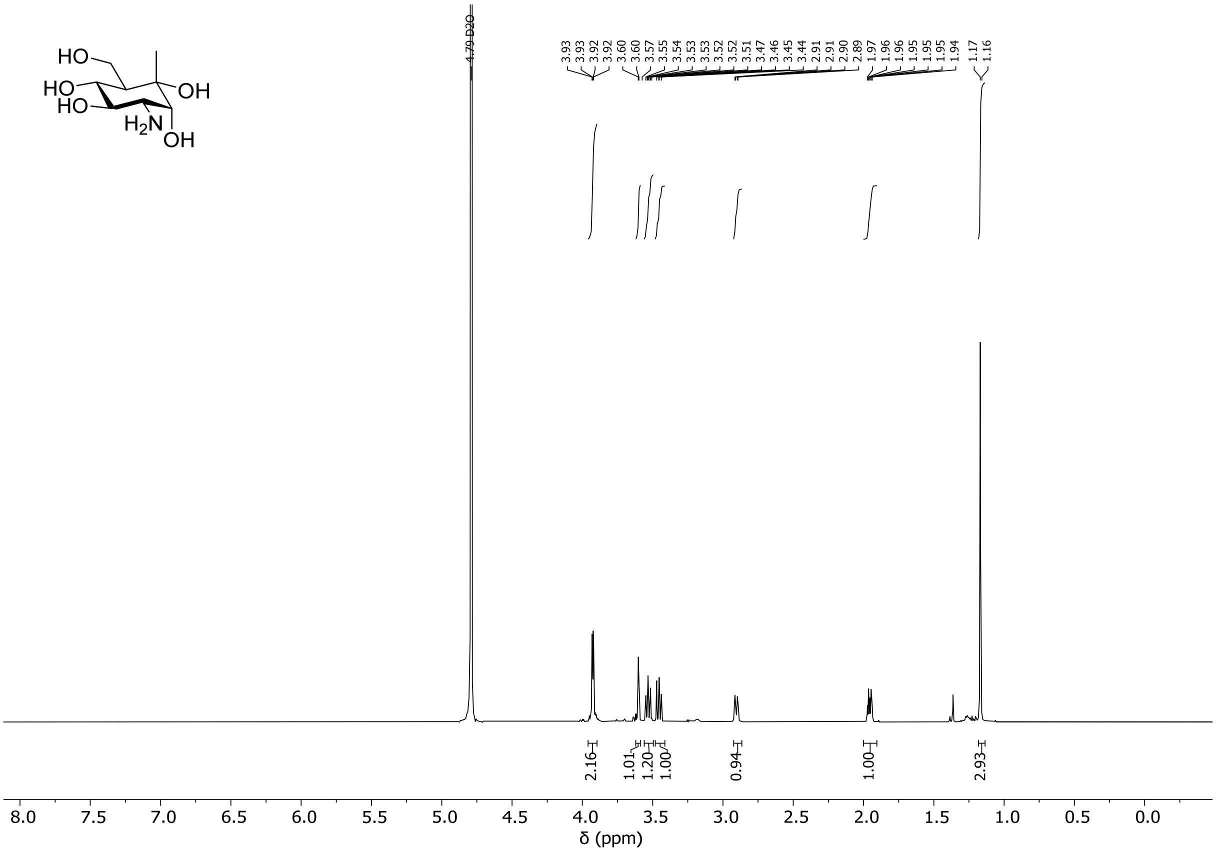

^{1}H NMR spectrum (600 MHz, D_2O) of **19**

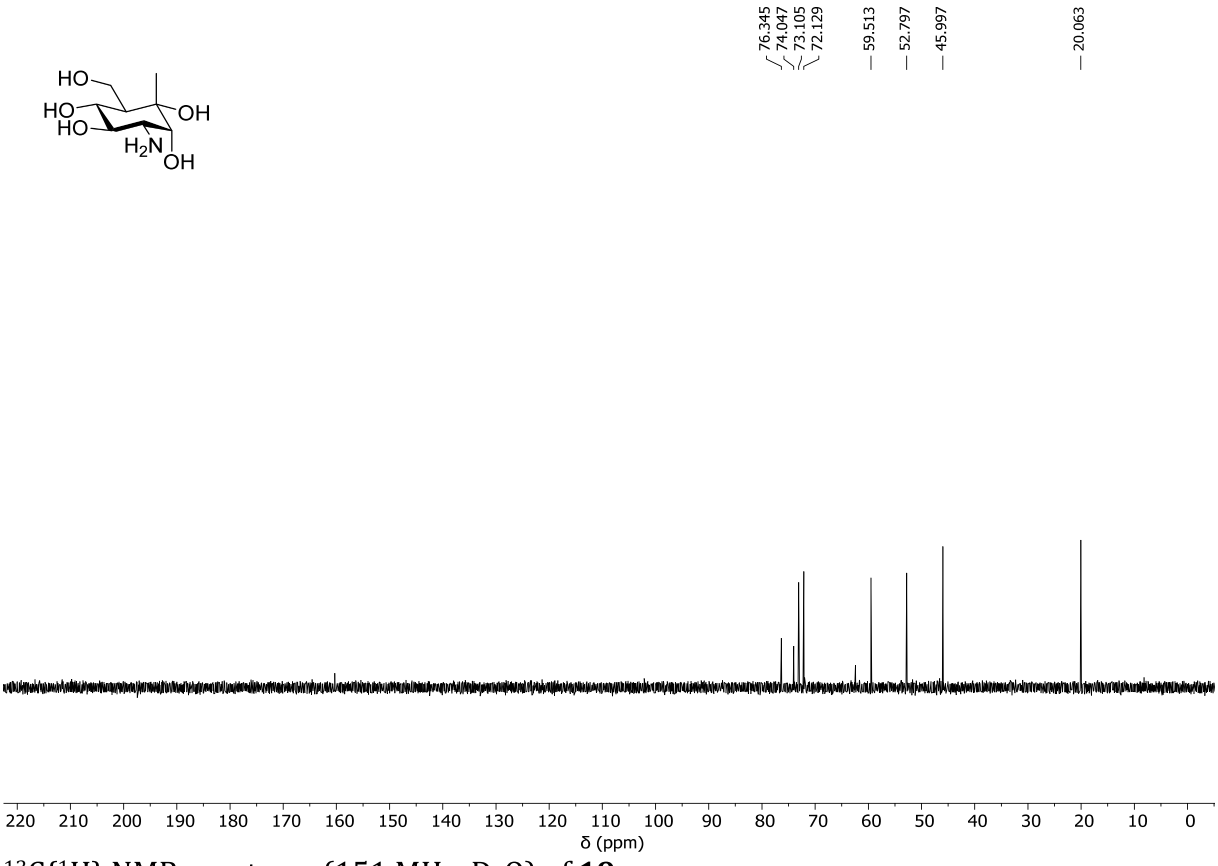

$^{13}C\{^{1}H\}$ NMR spectrum (151 MHz, D_2O) of **19**

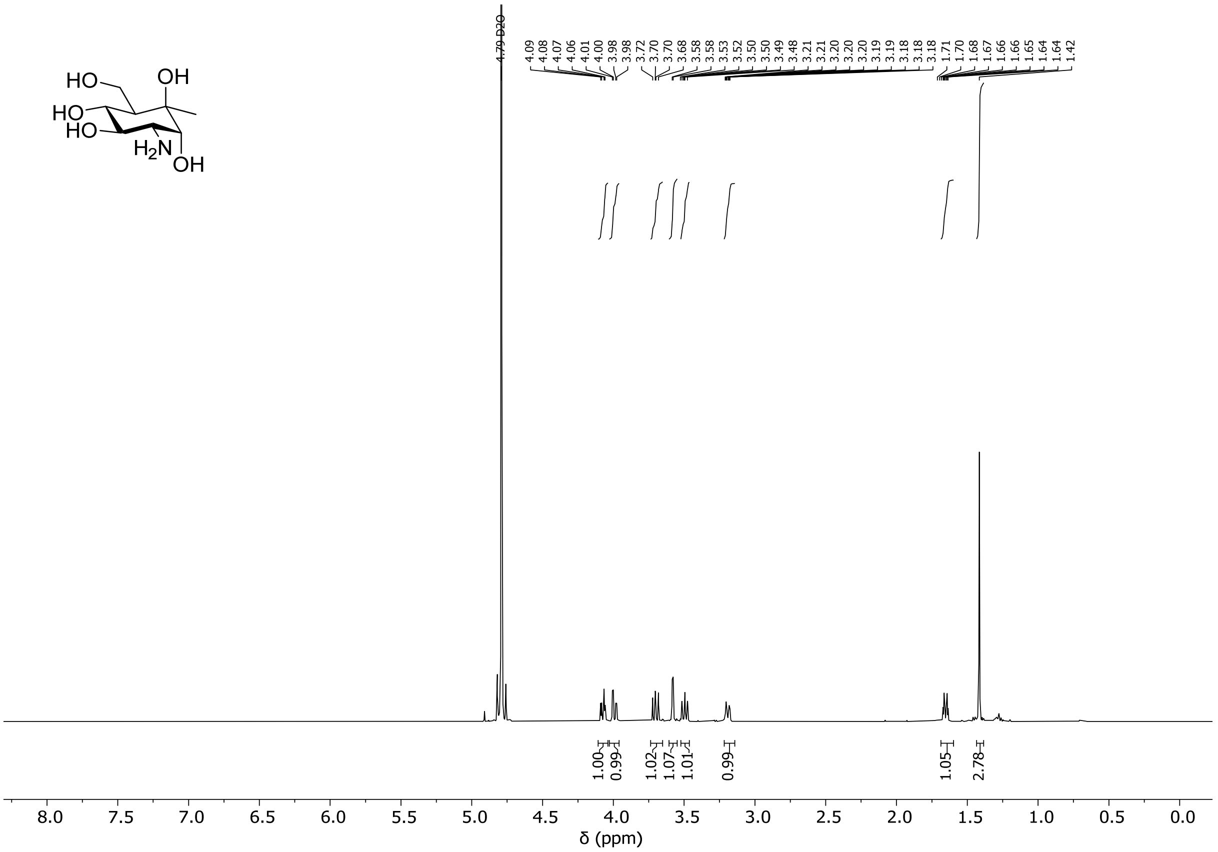

^{1}H NMR spectrum (500 MHz, D_2O) of **20**

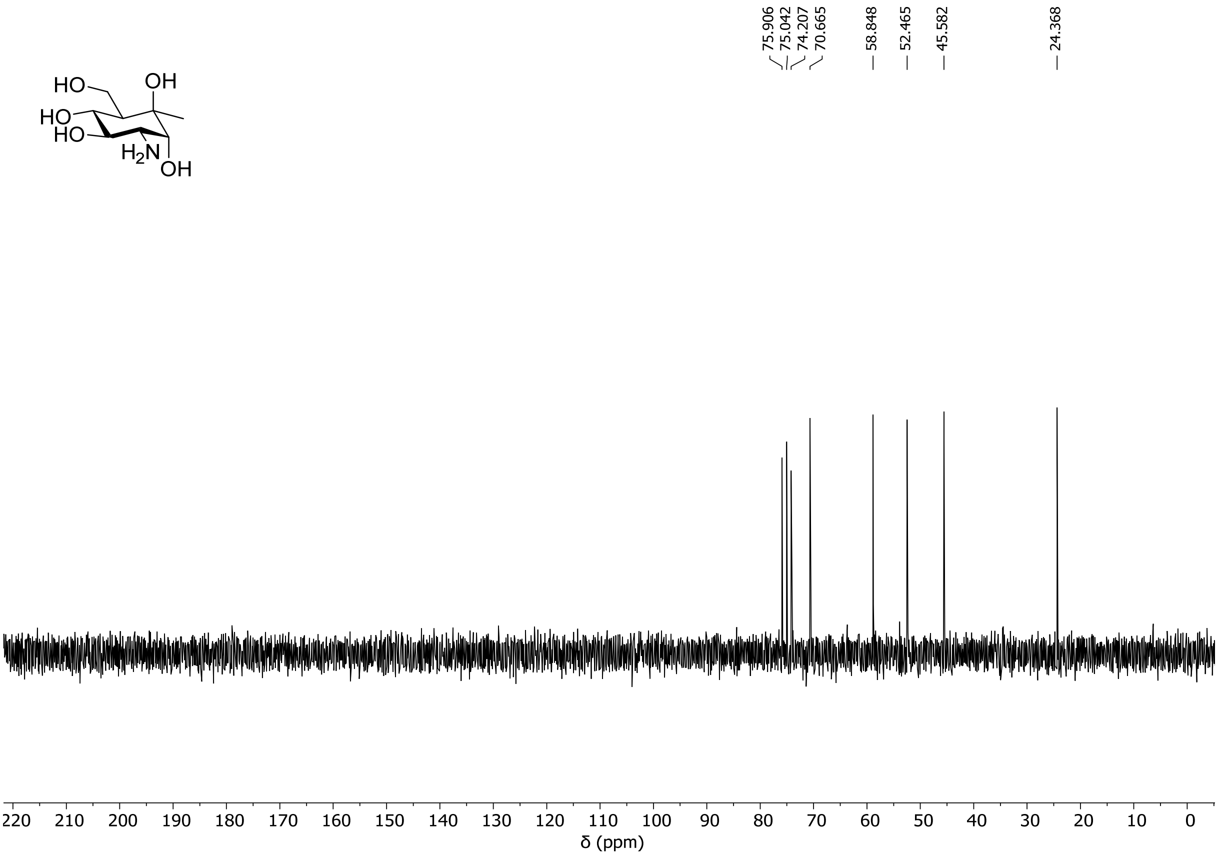

$^{13}C\{^{1}H\}$ NMR spectrum (126 MHz, D_2O) of **20**

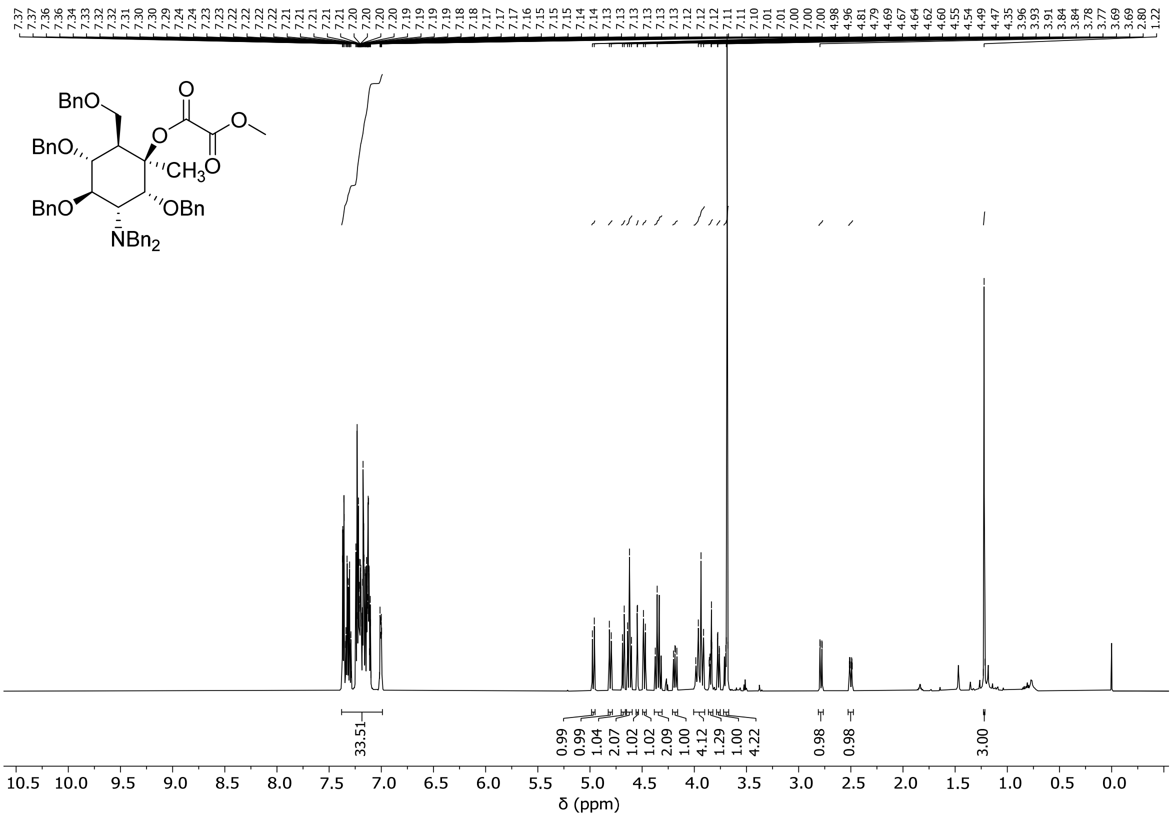

^{1}H NMR spectrum (600 MHz, $CDCl_3$) of **21**

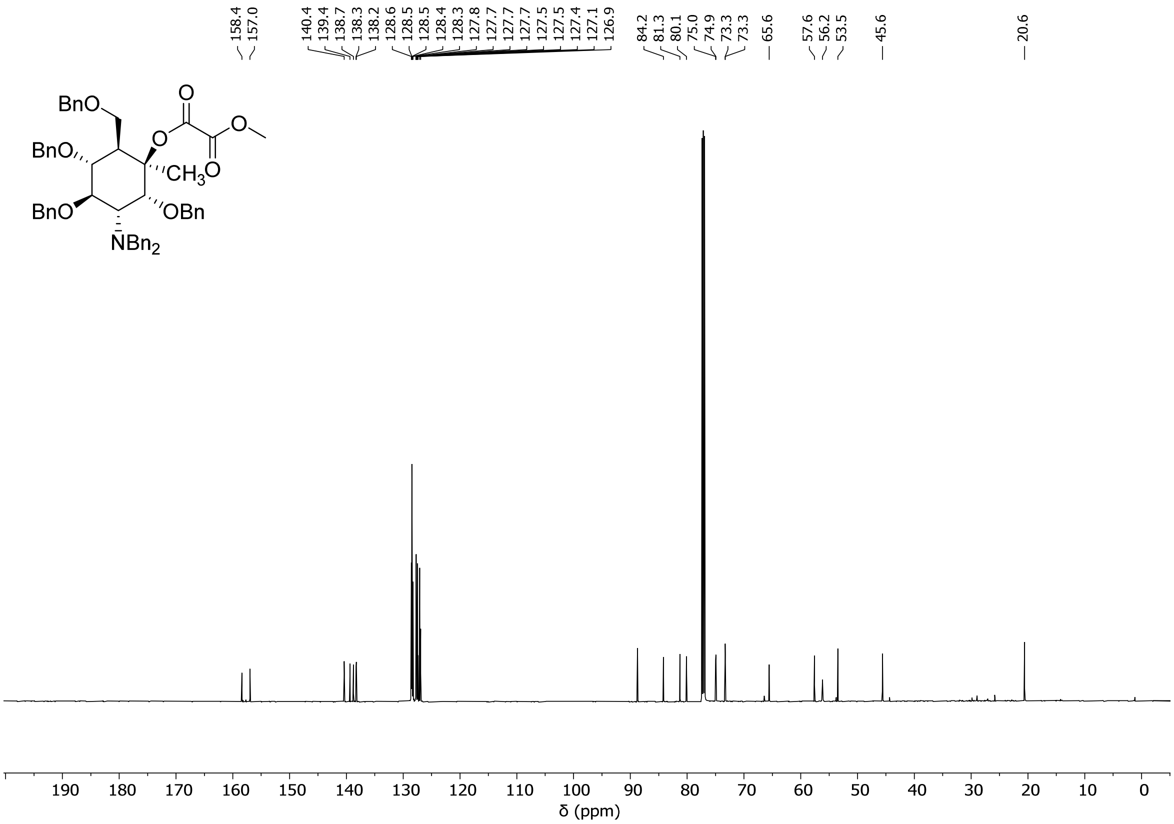

$^{13}C\{^{1}H\}$ NMR spectrum (150 MHz, $CDCl_3$) of **21**

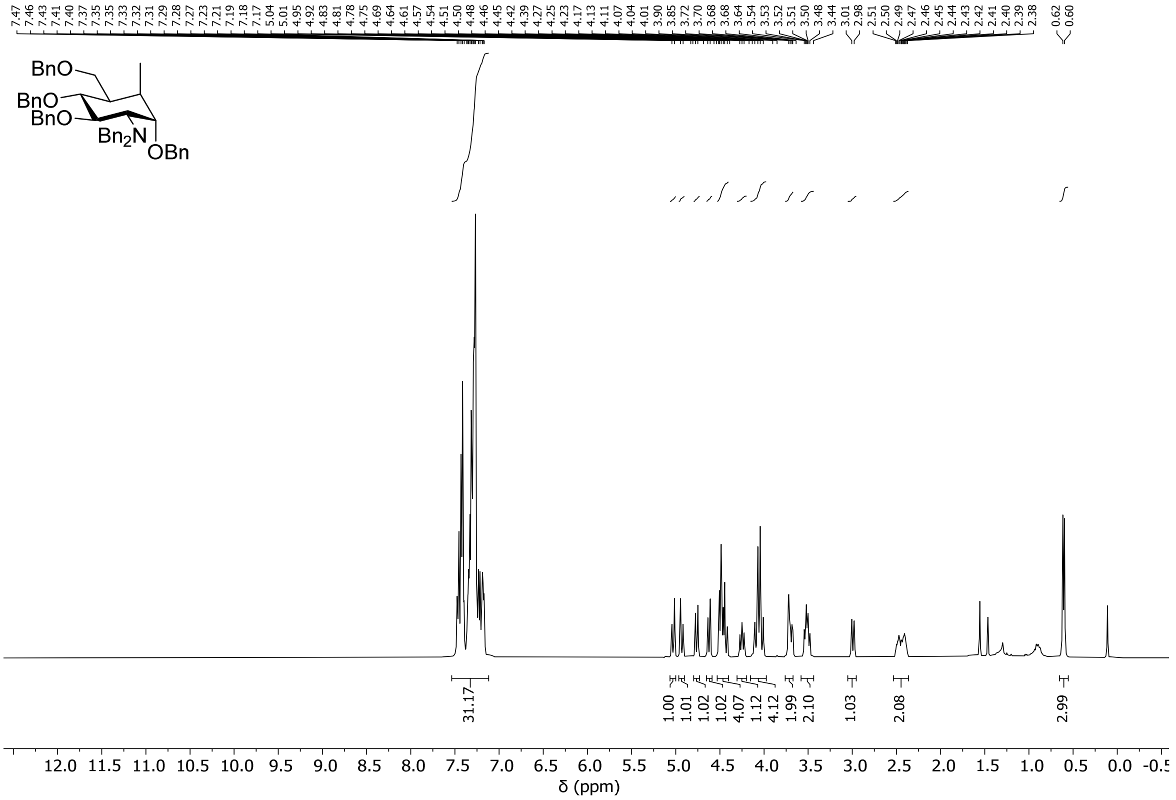

^{1}H NMR spectrum (400 MHz, $CDCl_3$) of **22**

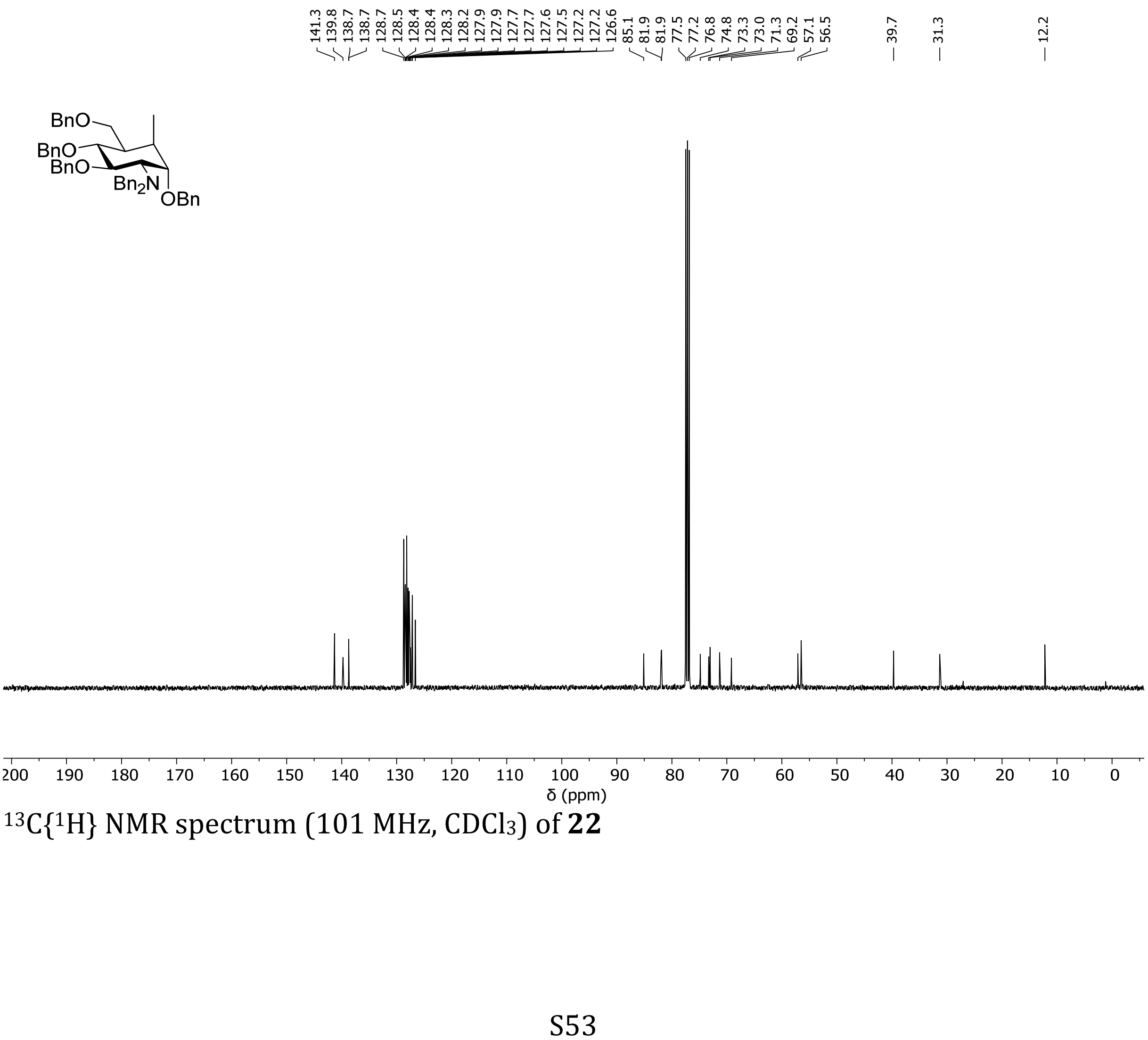

$^{13}C\{^{1}H\}$ NMR spectrum (101 MHz, $CDCl_3$) of **22**

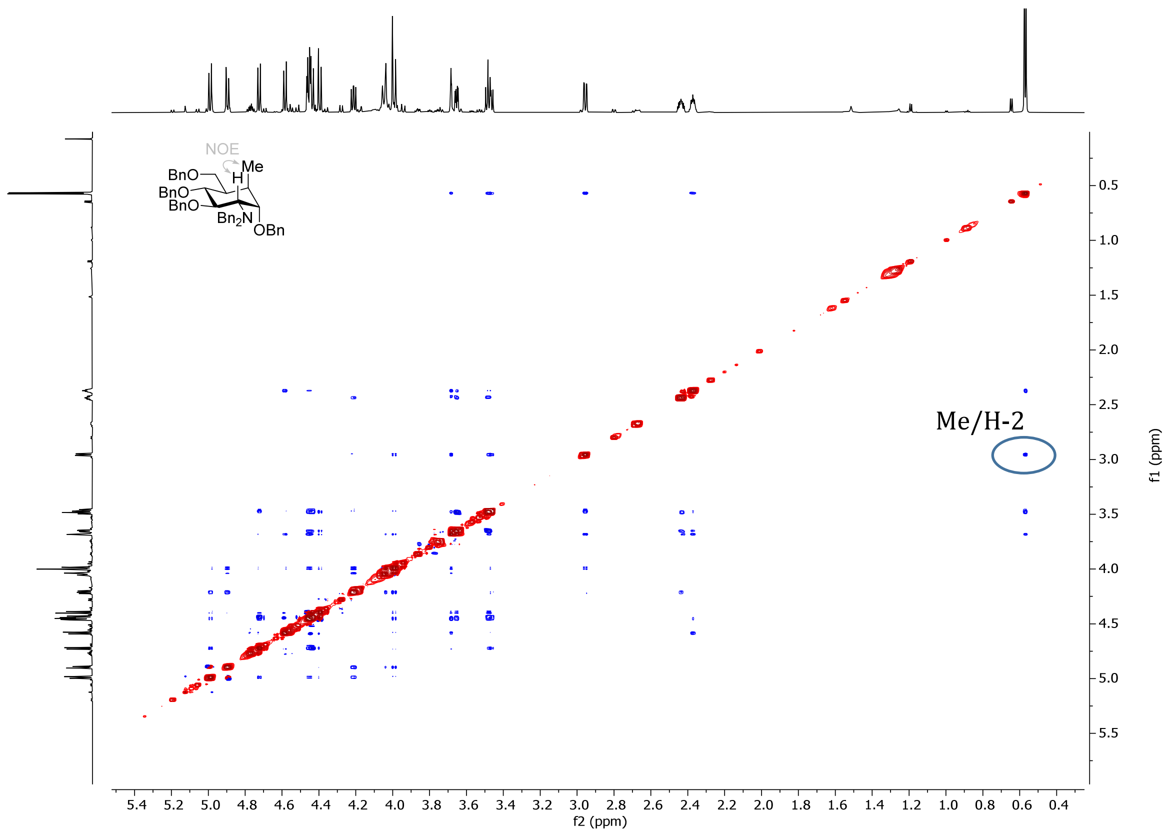

ROESY (800 MHz, $CDCl_3$) spectrum of **22**

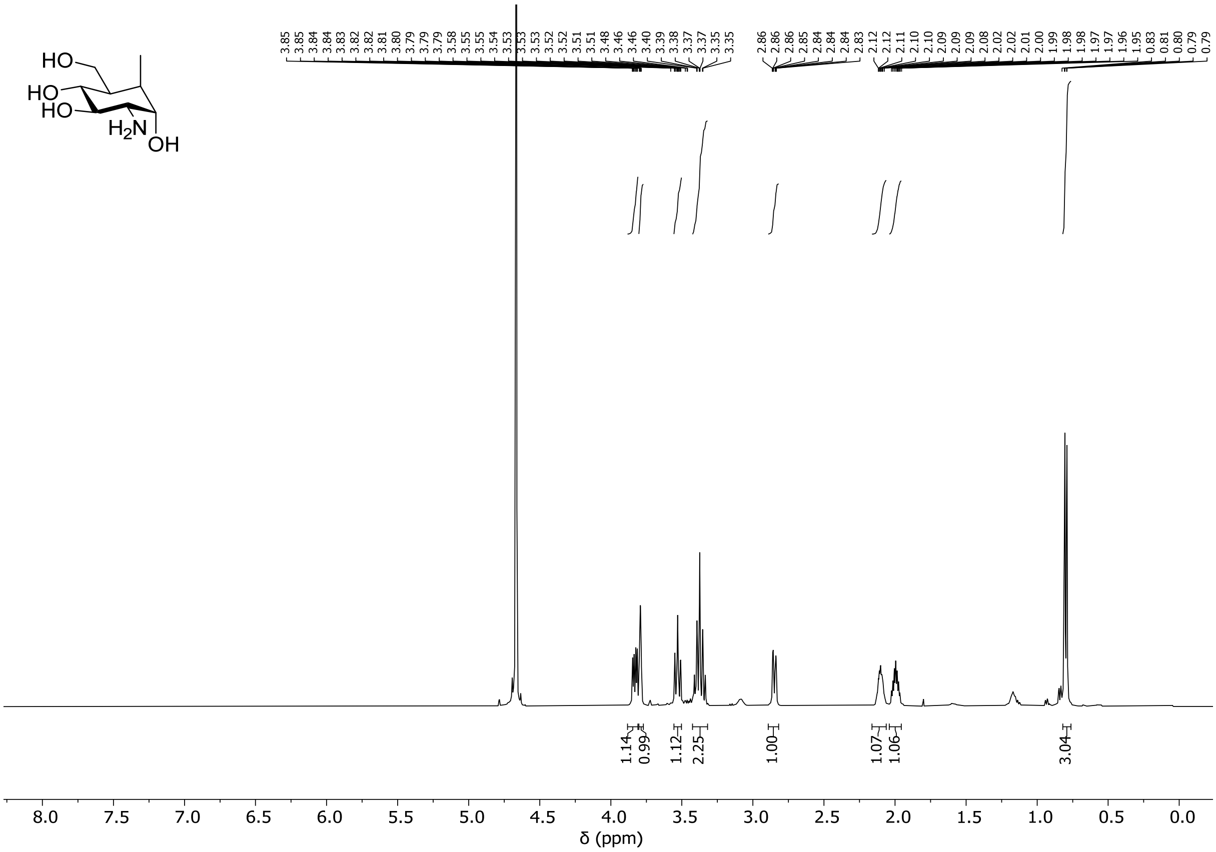

^{1}H NMR spectrum (500 MHz, D_2O) of **23**

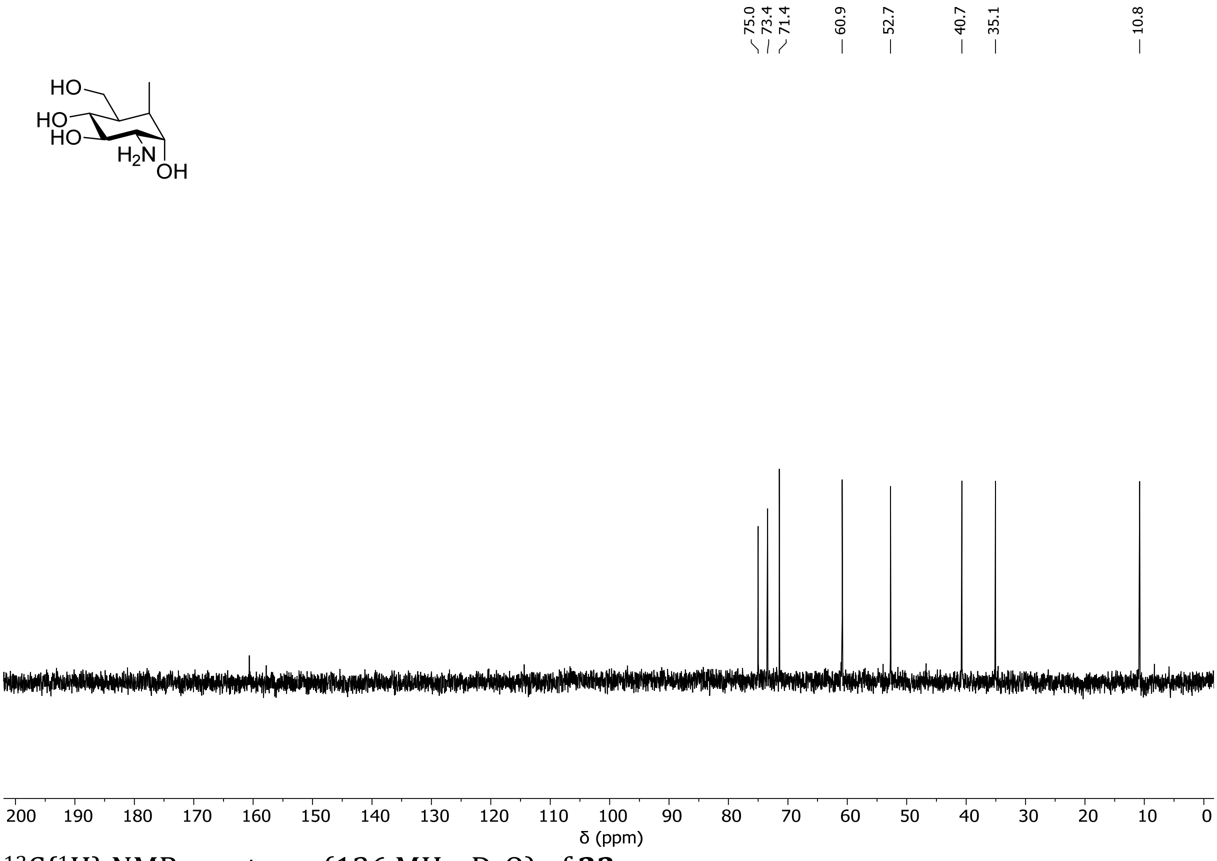

^{13}C{^{1}H} NMR spectrum (126 MHz, D_2O) of **23**

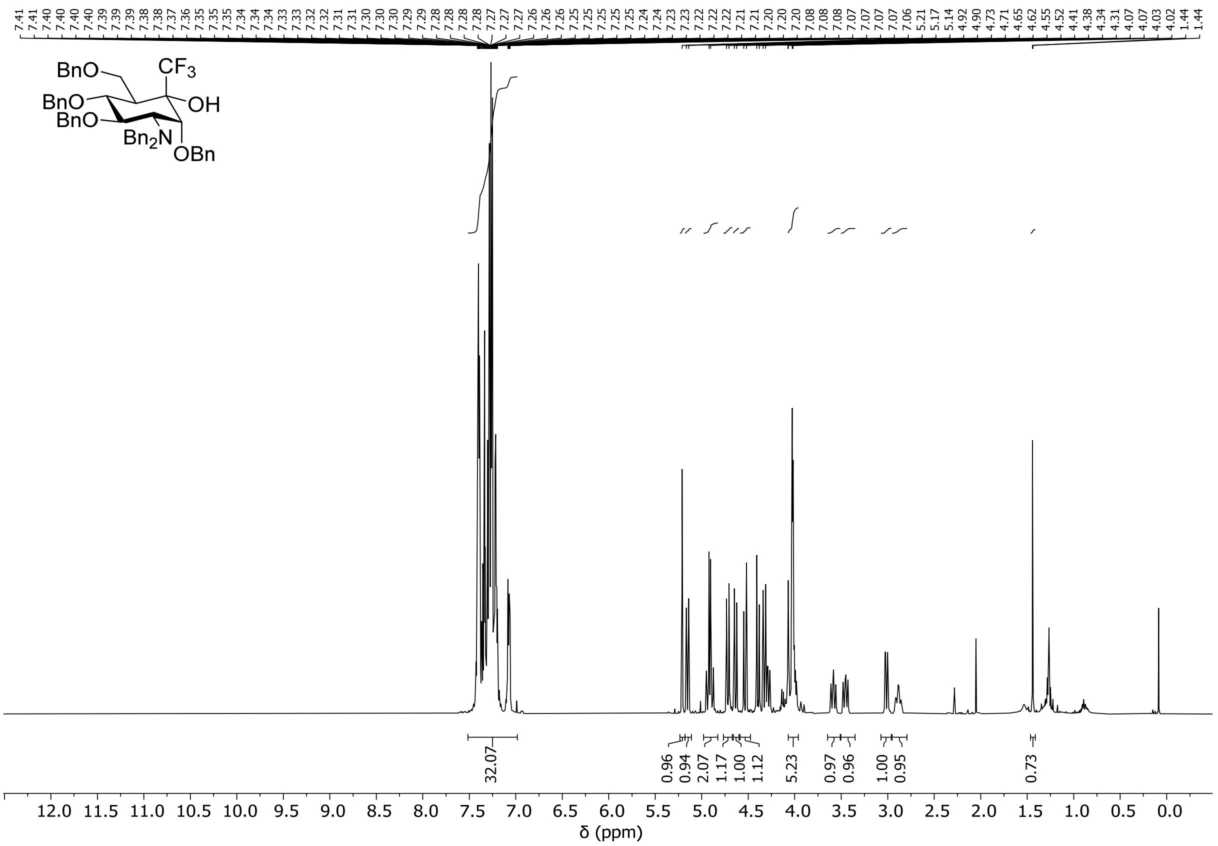

^{1}H NMR spectrum (400 MHz, $CDCl_3$) of **24**

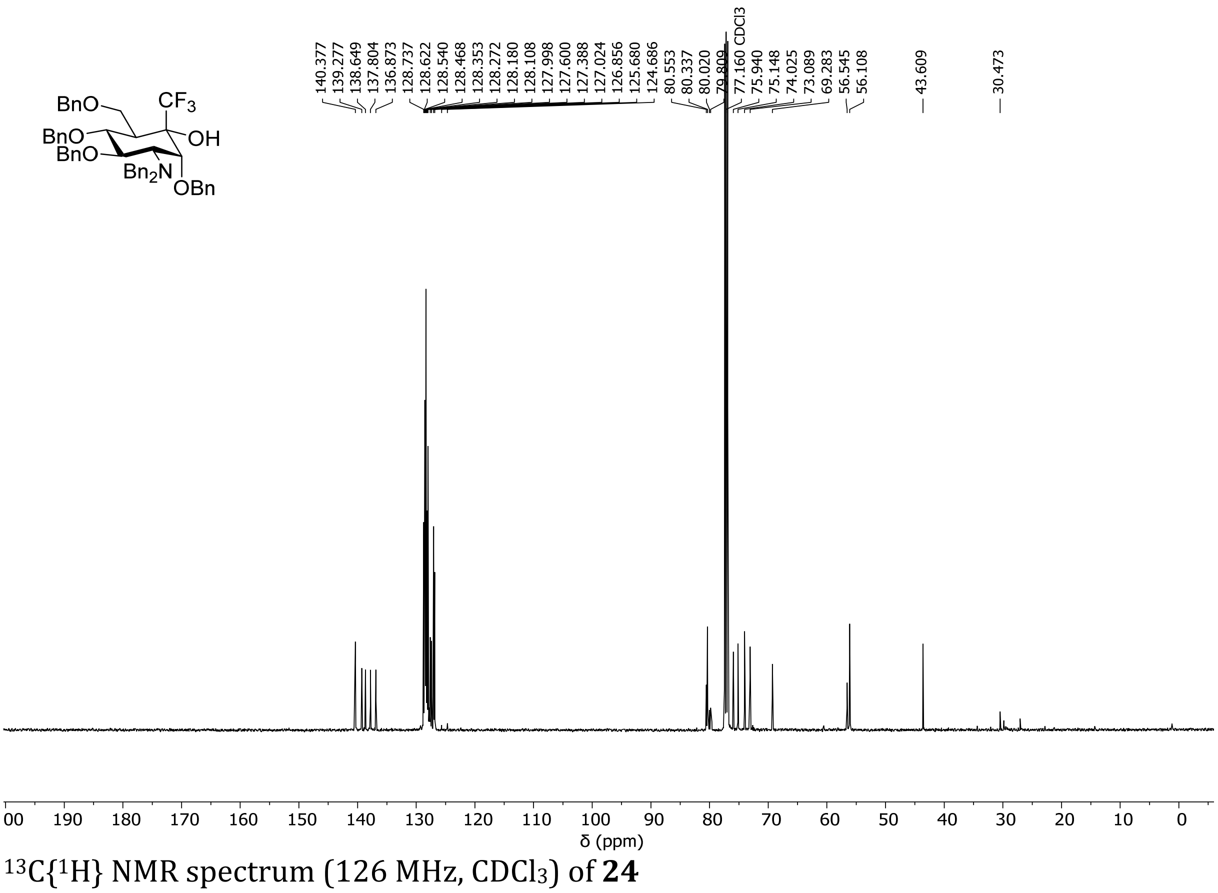

$^{13}C\{^{1}H\}$ NMR spectrum (126 MHz, $CDCl_3$) of **24**

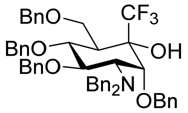

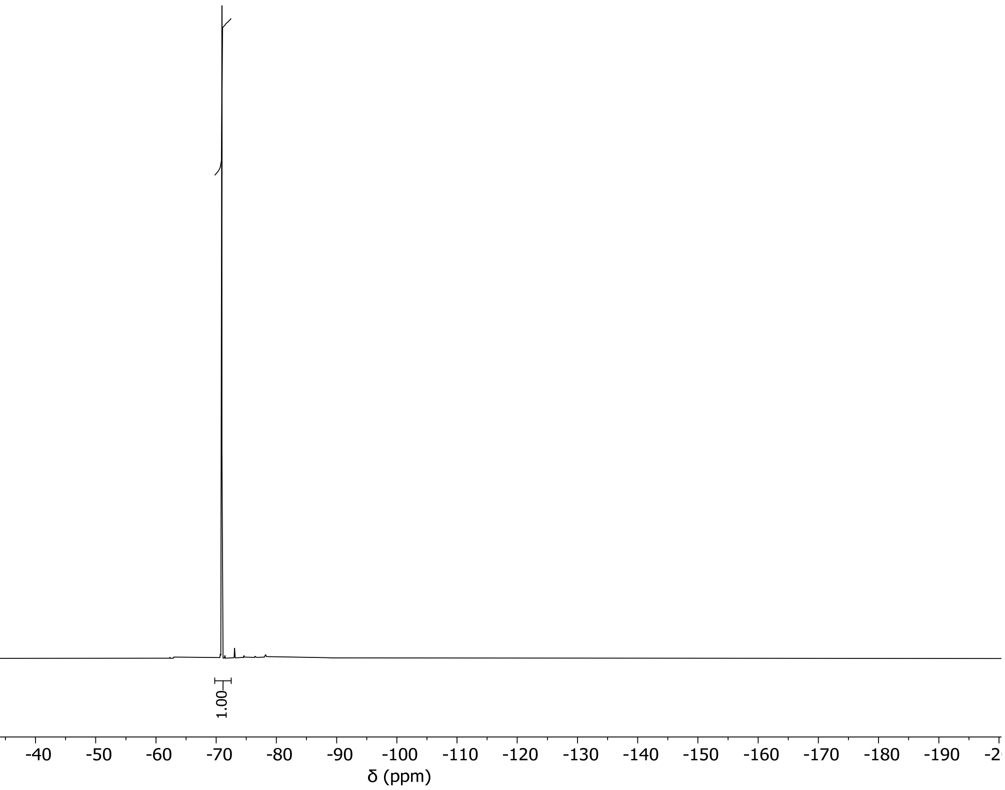

^{19}F NMR spectrum (377 MHz, $CDCl_3$) of **24**

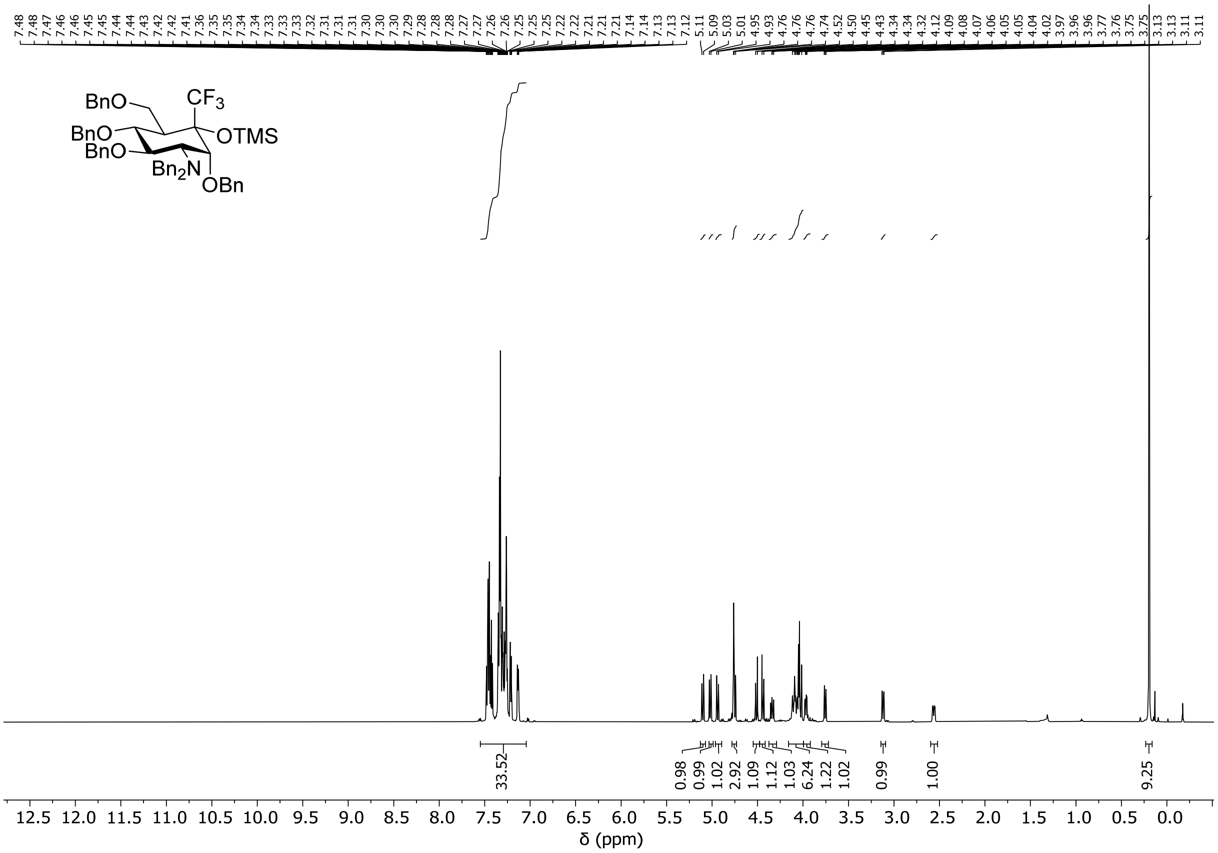

^{1}H NMR spectrum (600 MHz, CDCl$_3$) of **TMS-24**

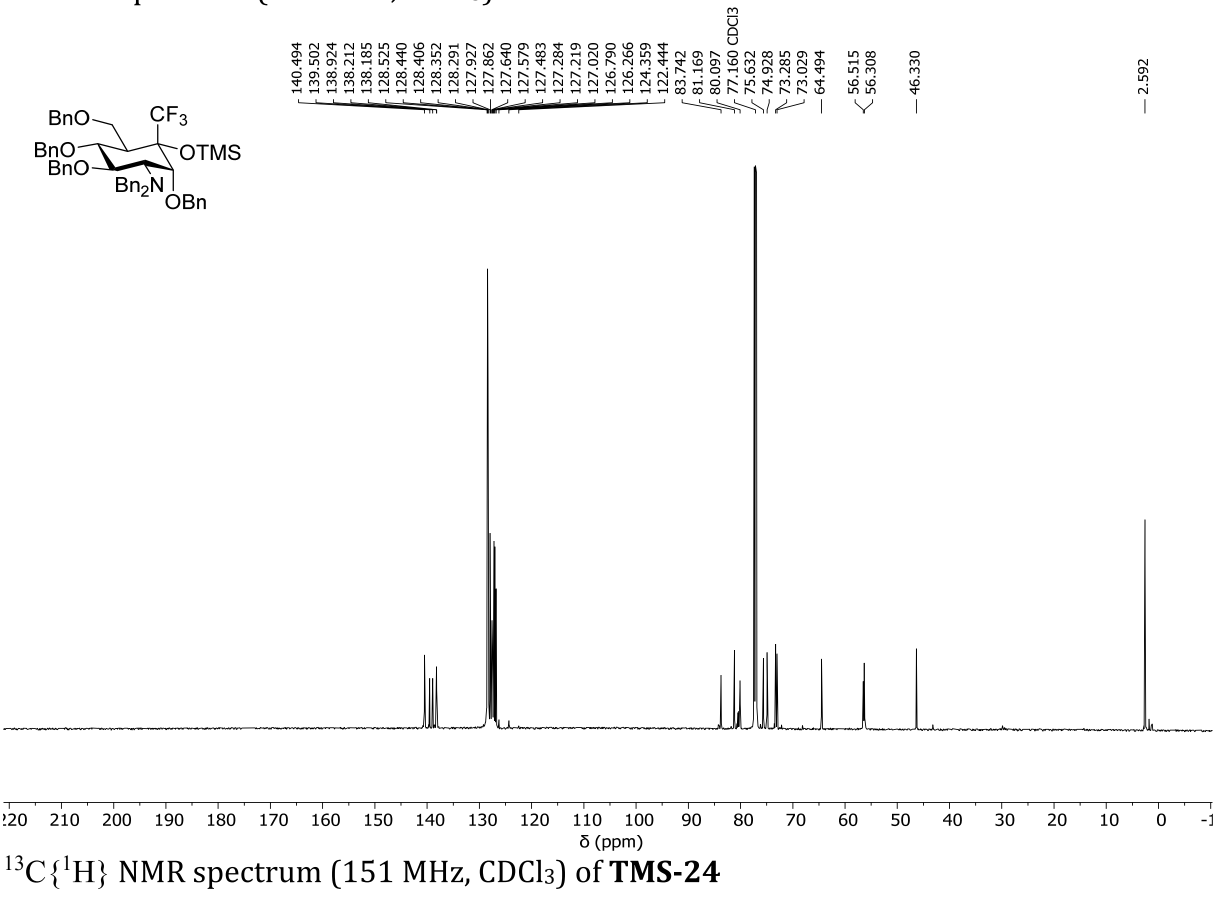

$^{13}C\{^{1}H\}$ NMR spectrum (151 MHz, CDCl$_3$) of **TMS-24**

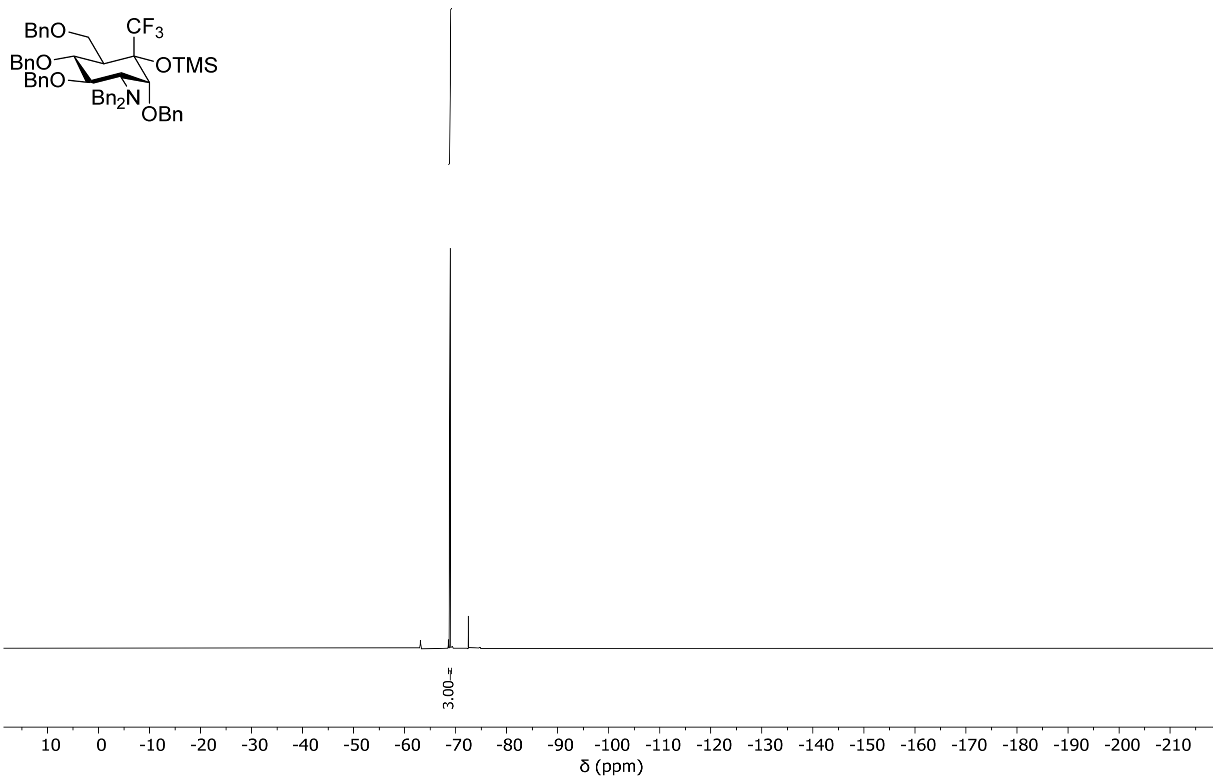

^{19}F NMR spectrum (377 MHz, $CDCl_3$) of **TMS-24**

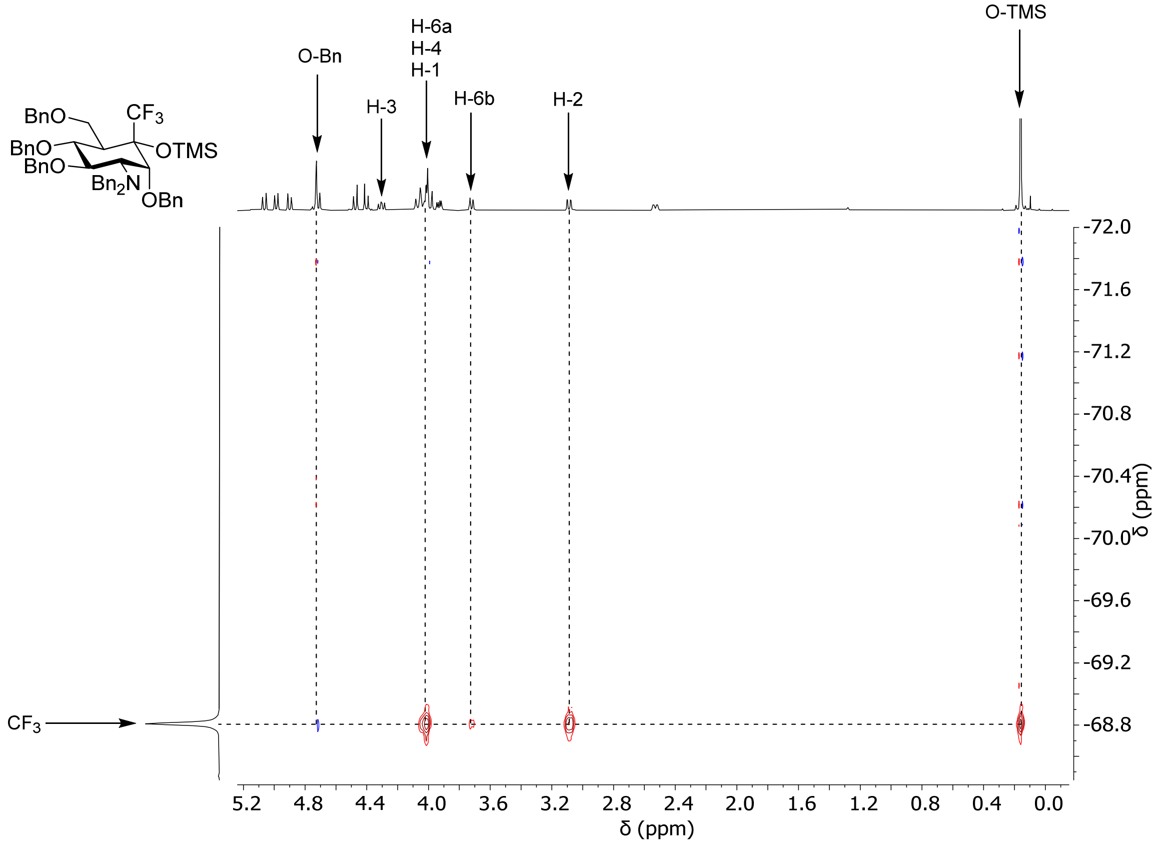

^{19}F-^{1}H HOESY spectrum of **TMS-24**

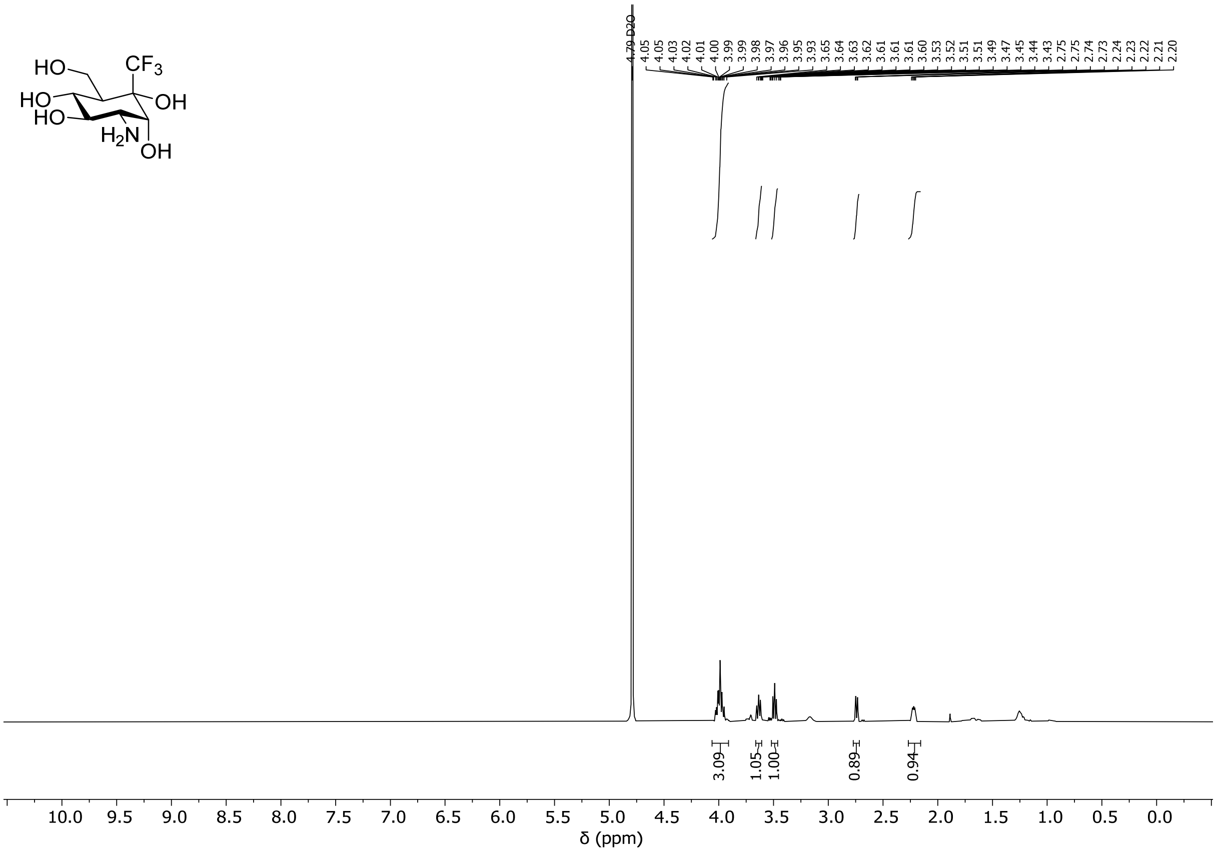

^{1}H NMR spectrum (600 MHz, D_2O) of **25**

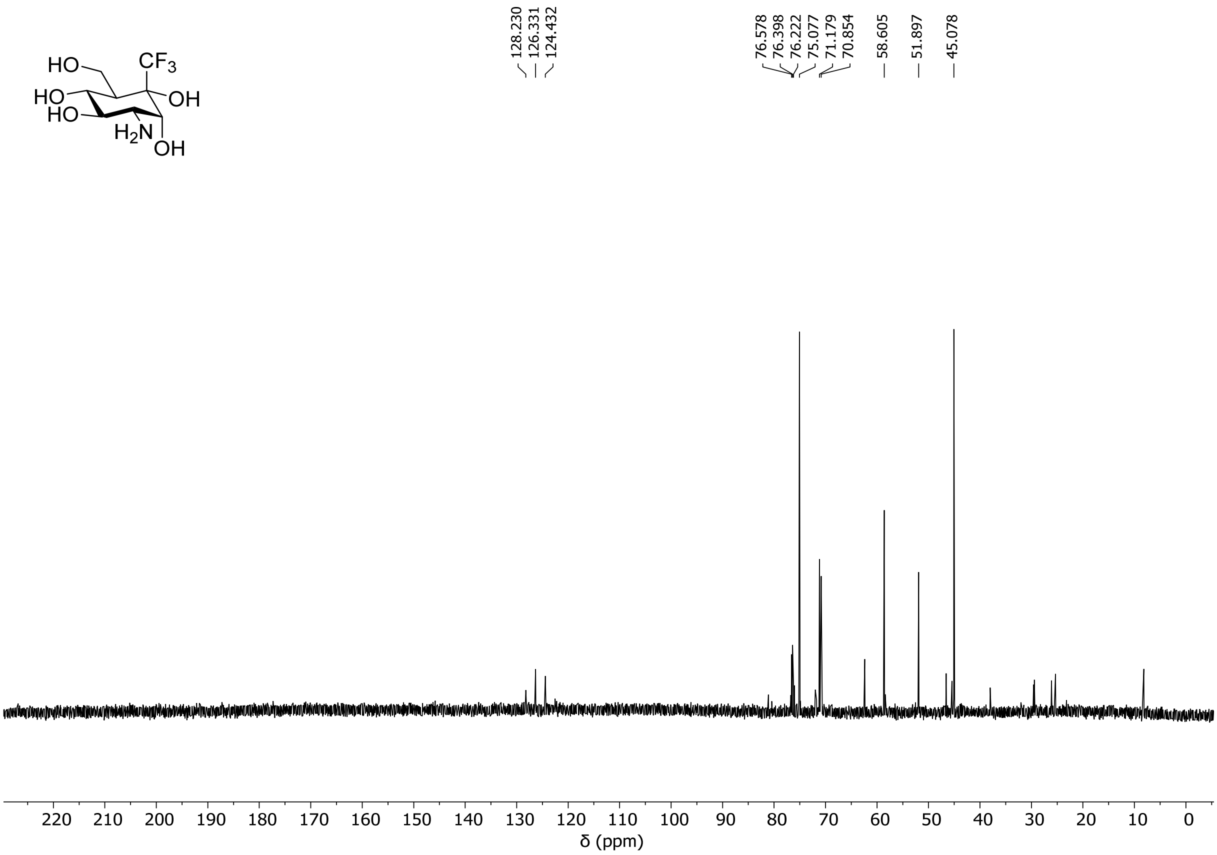

$^{13}C\{^{1}H\}$ NMR spectrum (151 MHz, D_2O) of **25**

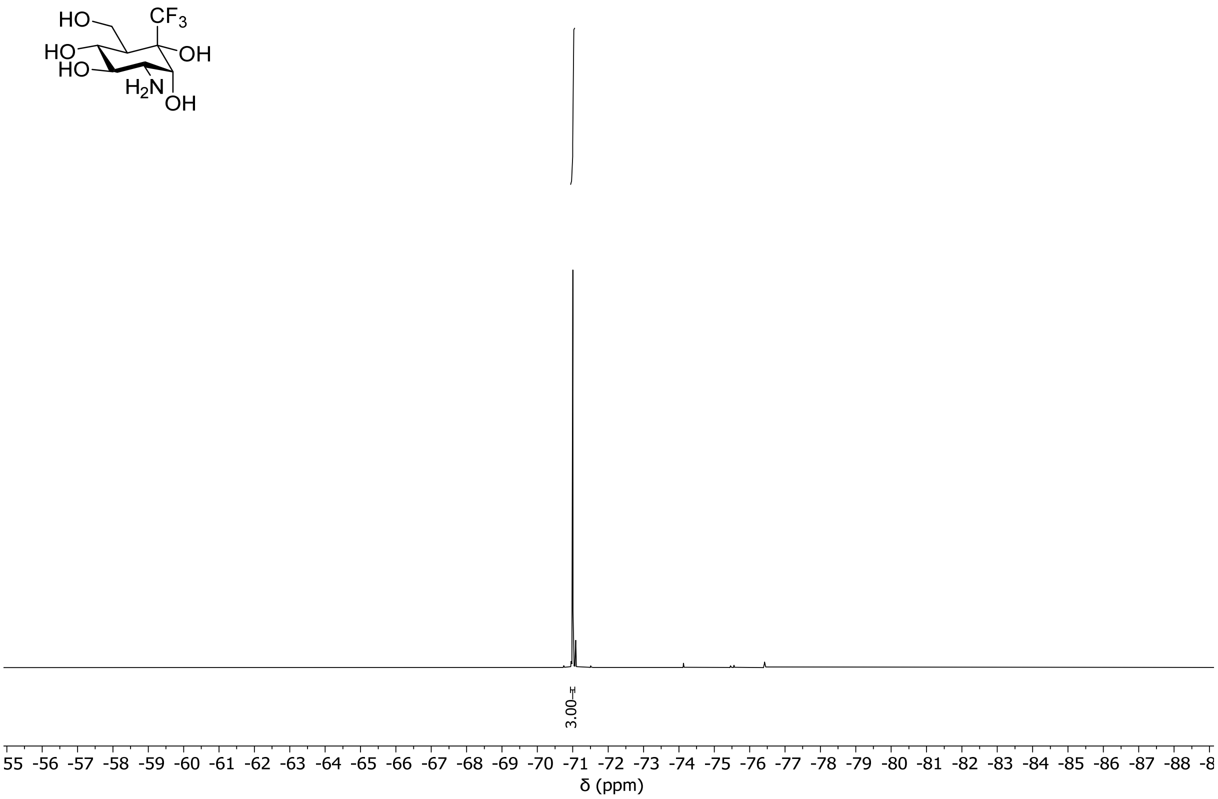

^{19}F NMR spectrum (377 MHz, D_2O) of **25**

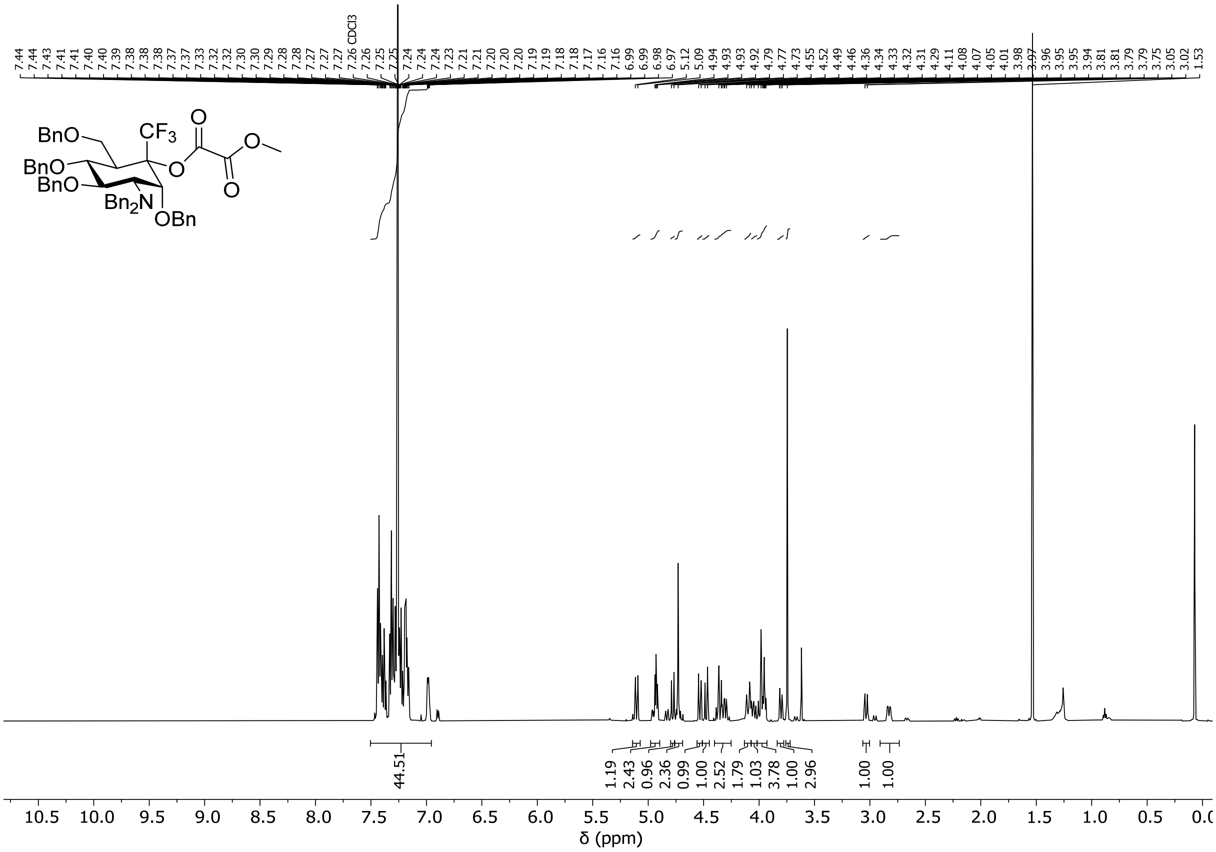

^{1}H NMR spectrum (500 MHz, $CDCl_3$) of **26**

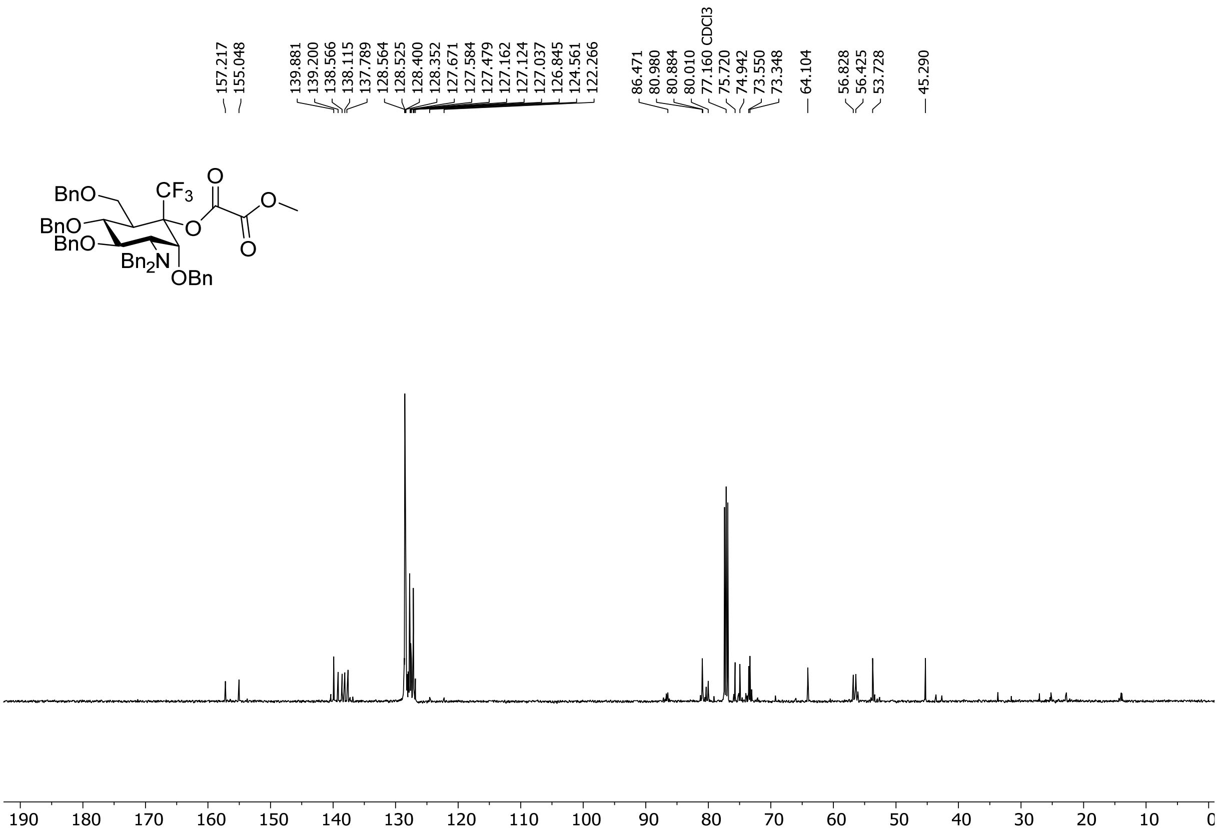

$^{13}C\{^{1}H\}$ NMR spectrum (126 MHz, $CDCl_3$) of **26**

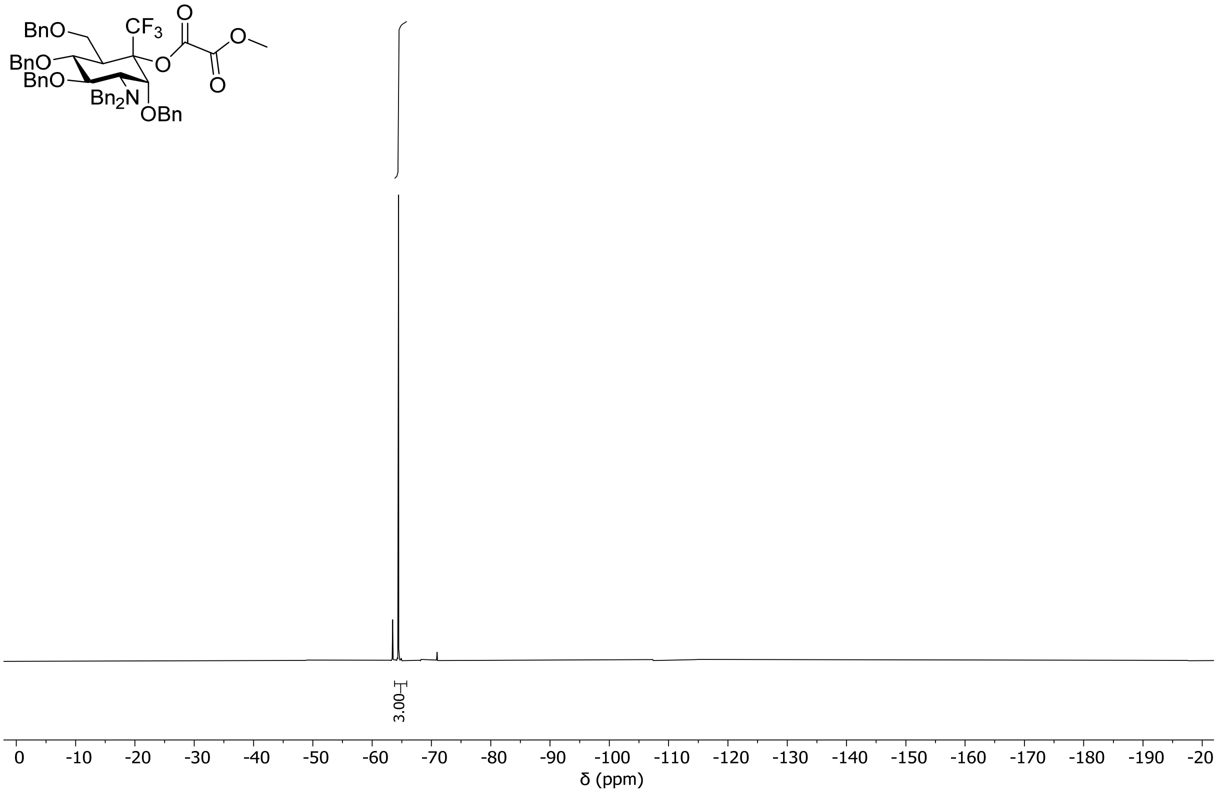

^{19}F NMR spectrum (471 MHz, $CDCl_3$) of **26**

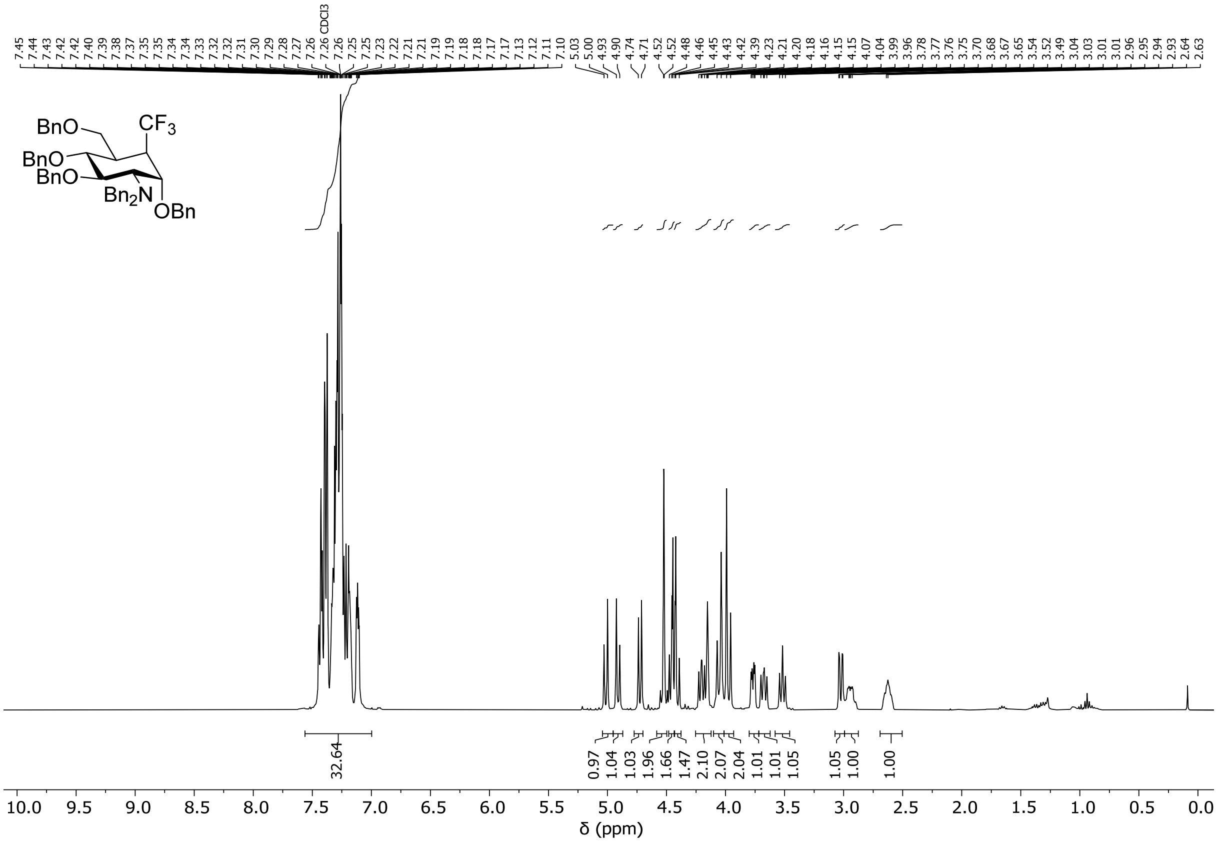

^{1}H NMR spectrum (500 MHz, $CDCl_3$) of **27**

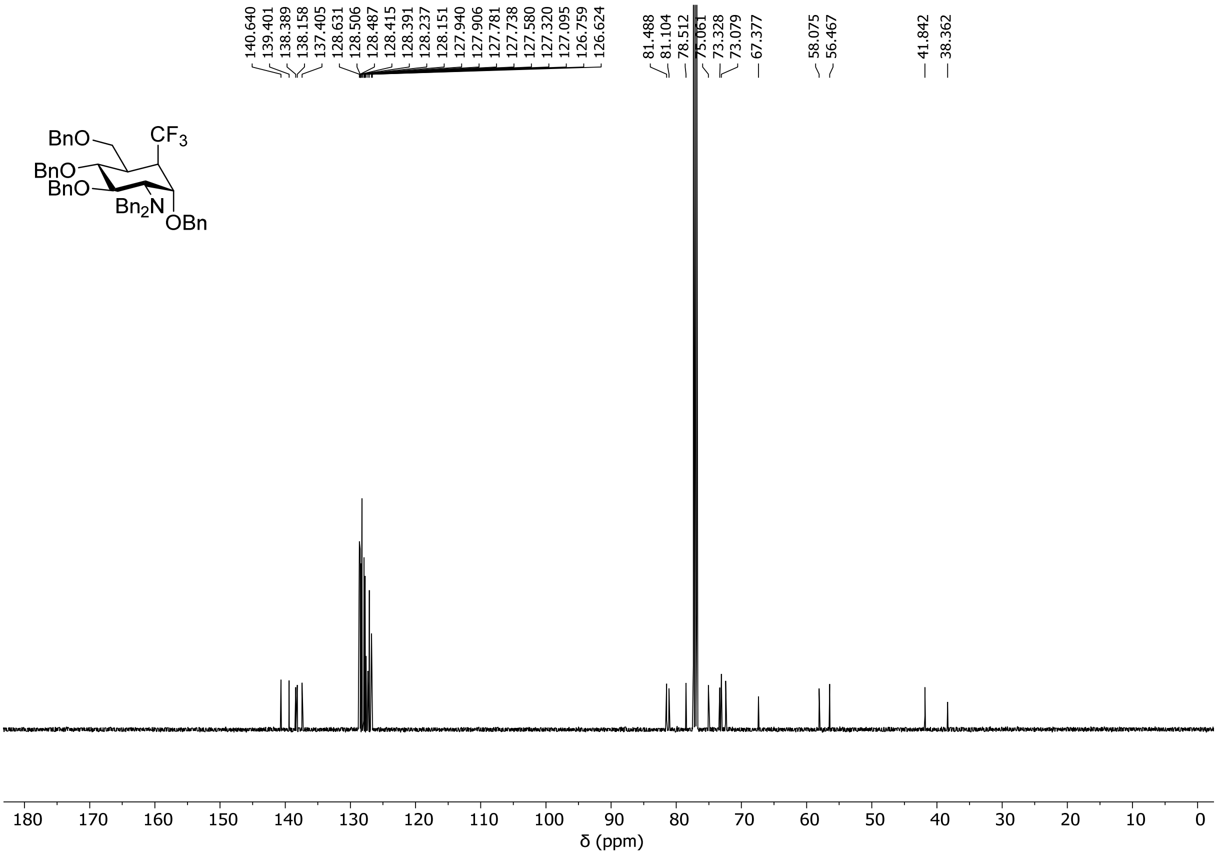

$^{13}C\{^{1}H\}$ NMR spectrum (121 MHz, $CDCl_3$) of **27**

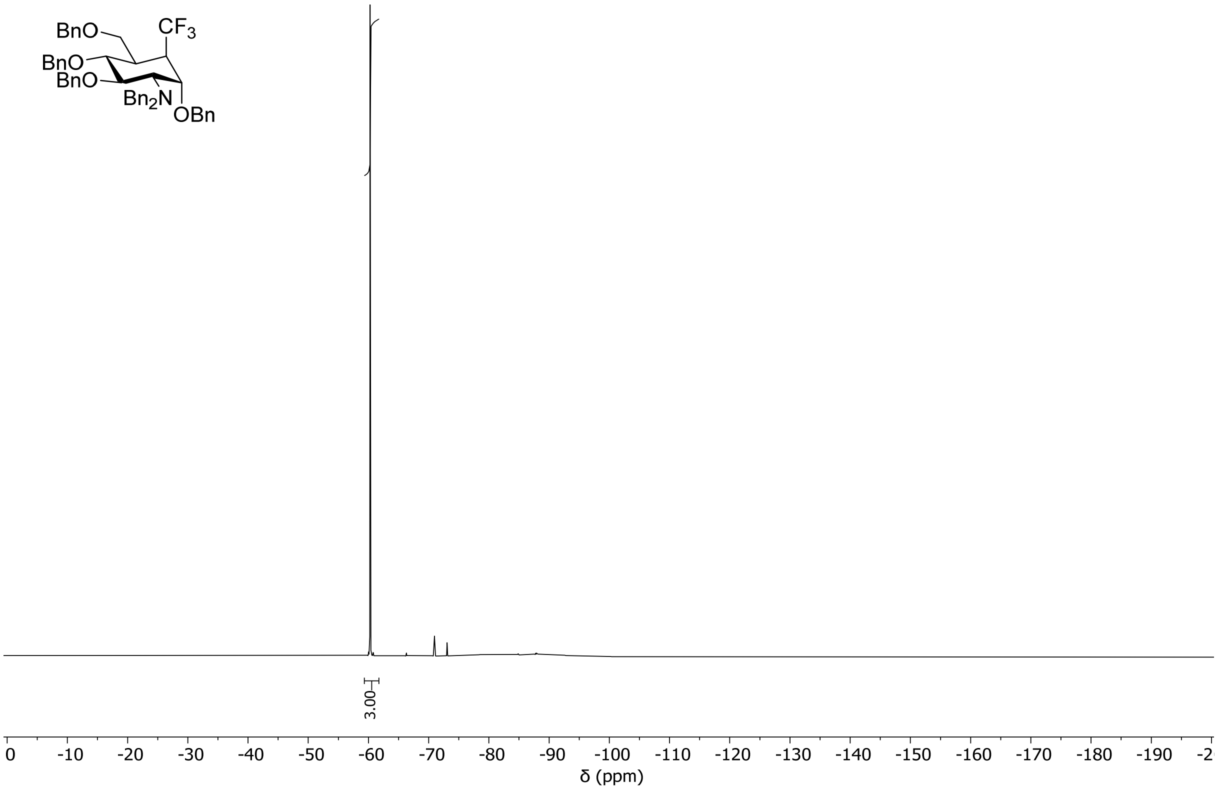

^{19}F NMR spectrum (377 MHz, $CDCl_3$) of **27**

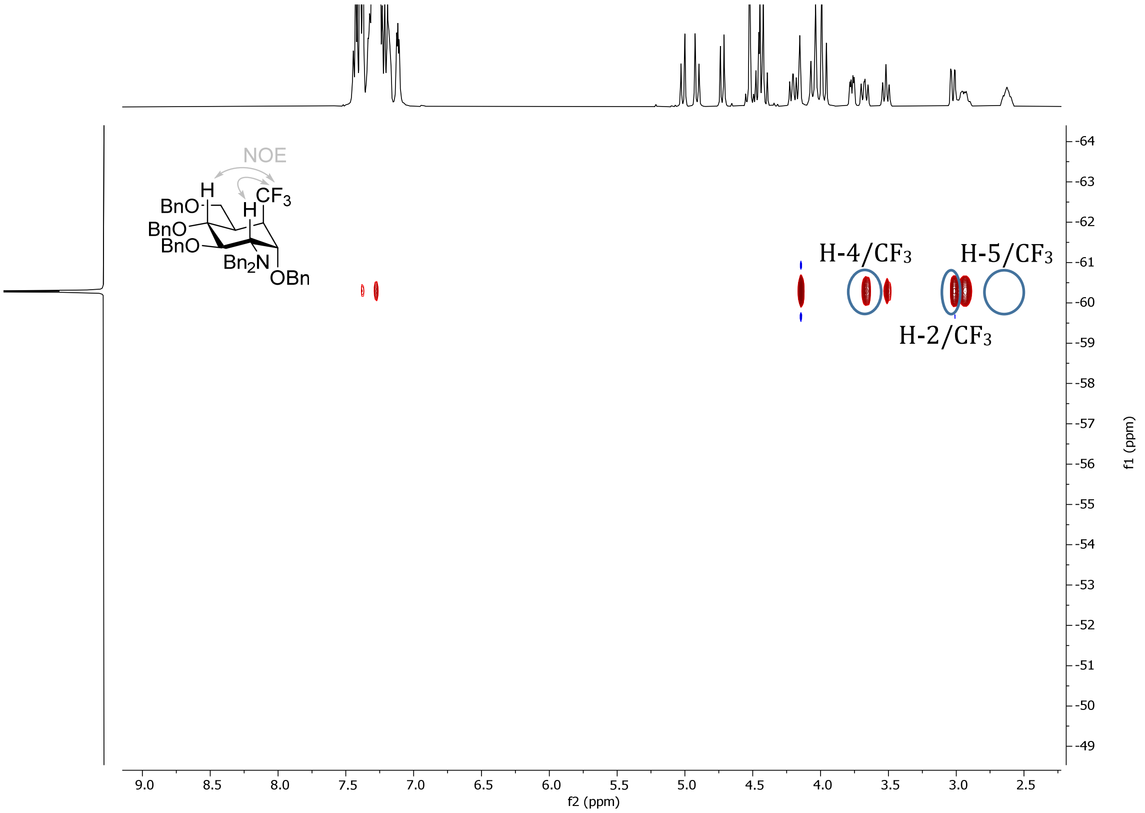

^{1}H-^{19}F HOESY (500 MHz/470 MHz, $CDCl_3$) spectrum of **27**. No cross peak between H-5 and the CF_3 group is visible.

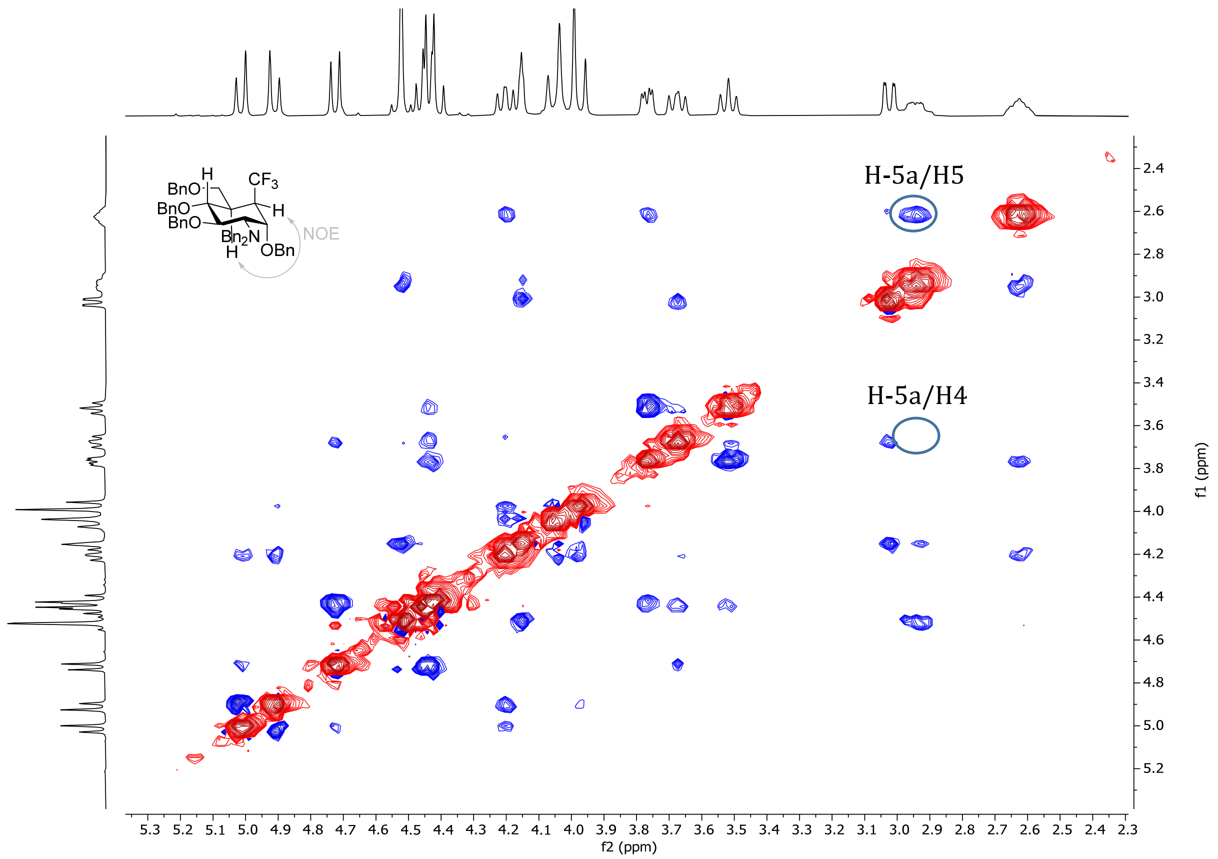

NOESY (500 MHz, CDCl3) spectrum of **27**. No cross peak between H-5a and H4 is visible.

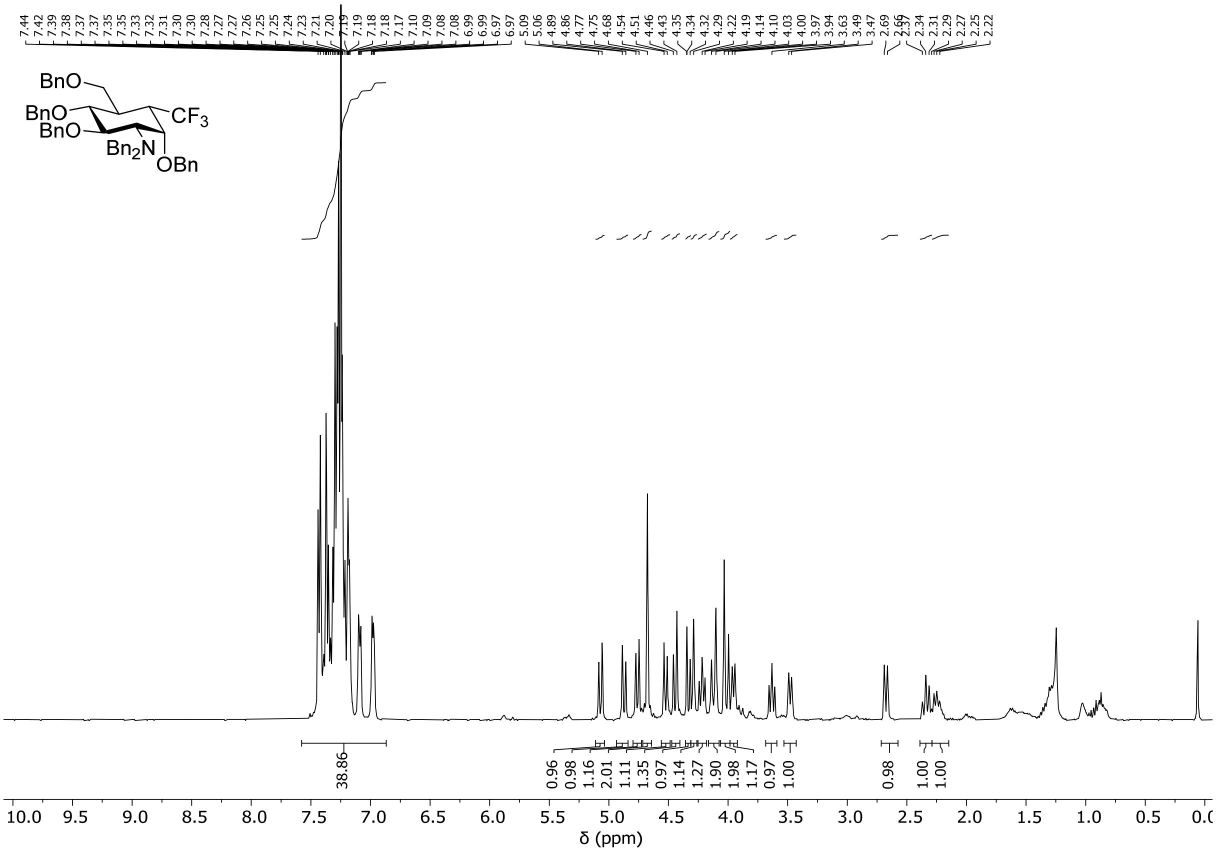

^{1}H NMR spectrum (400 MHz, $CDCl_3$) of **28**

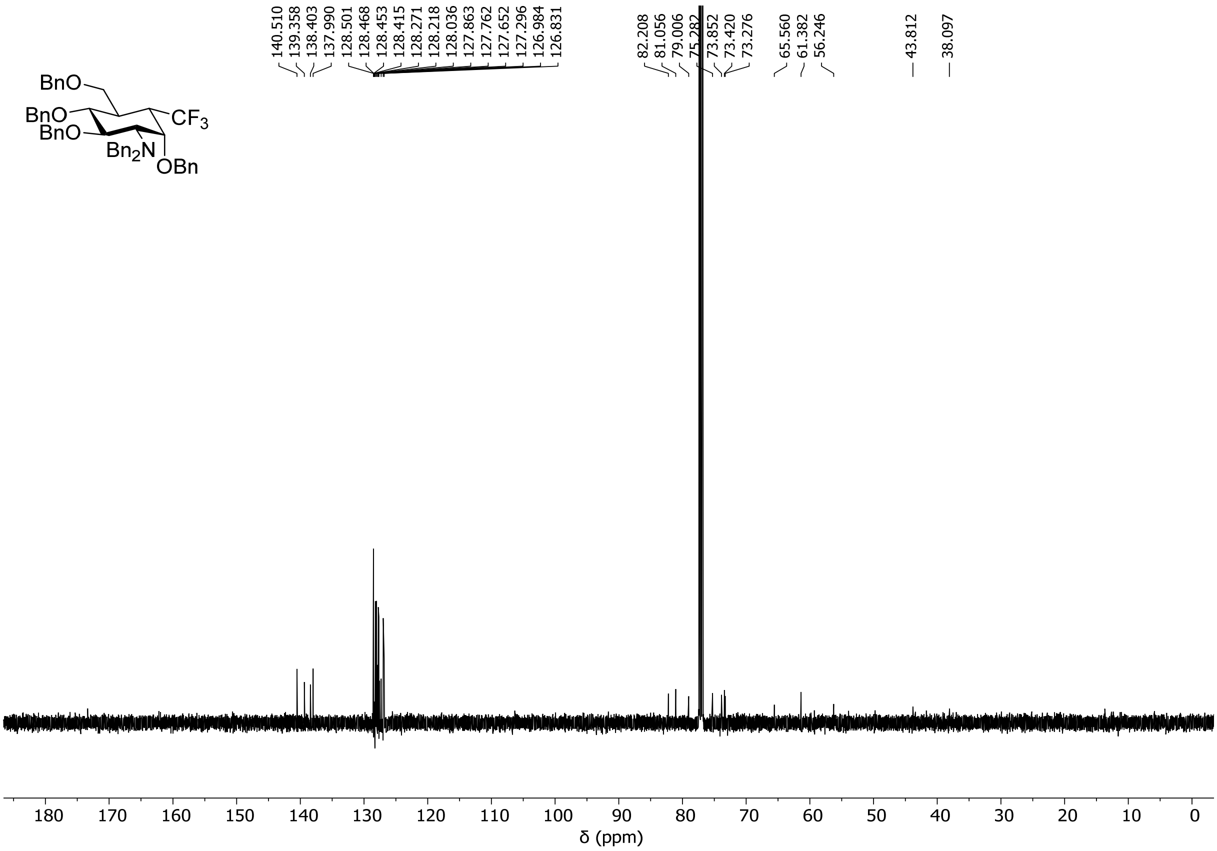

^{13}C{^{1}H} NMR spectrum (121 MHz, $CDCl_3$) of **28**

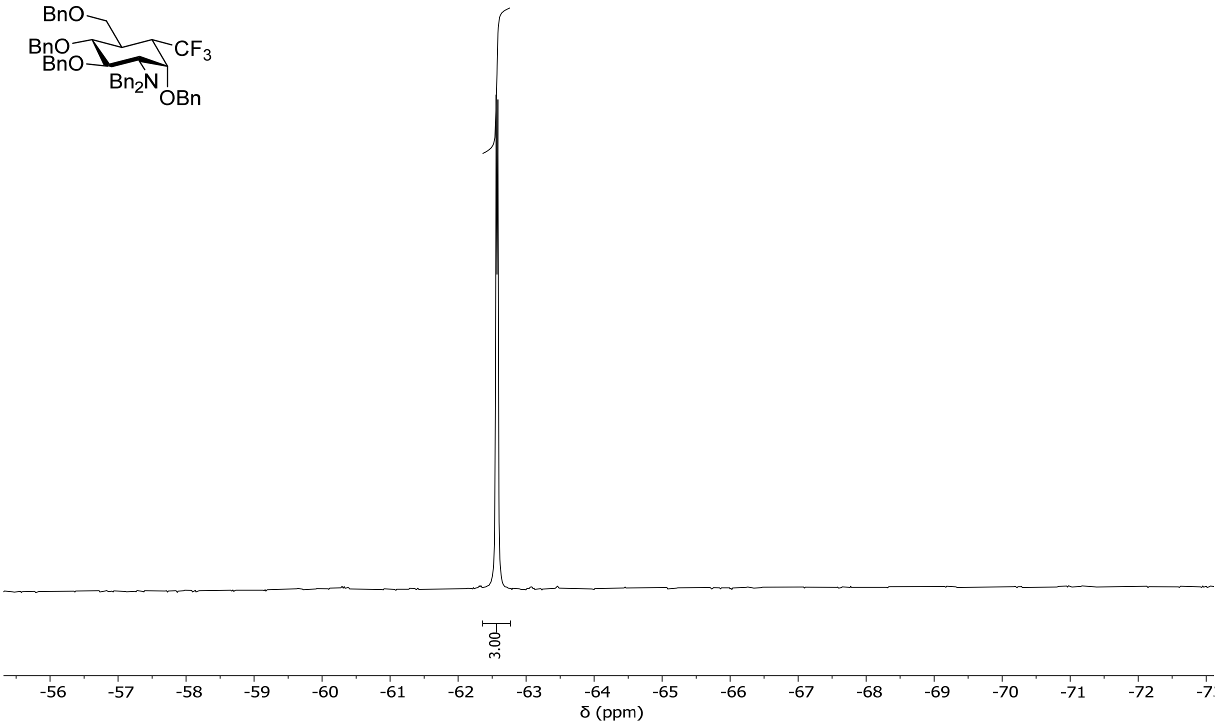

^{19}F NMR spectrum (377 MHz, $CDCl_3$) of **28**

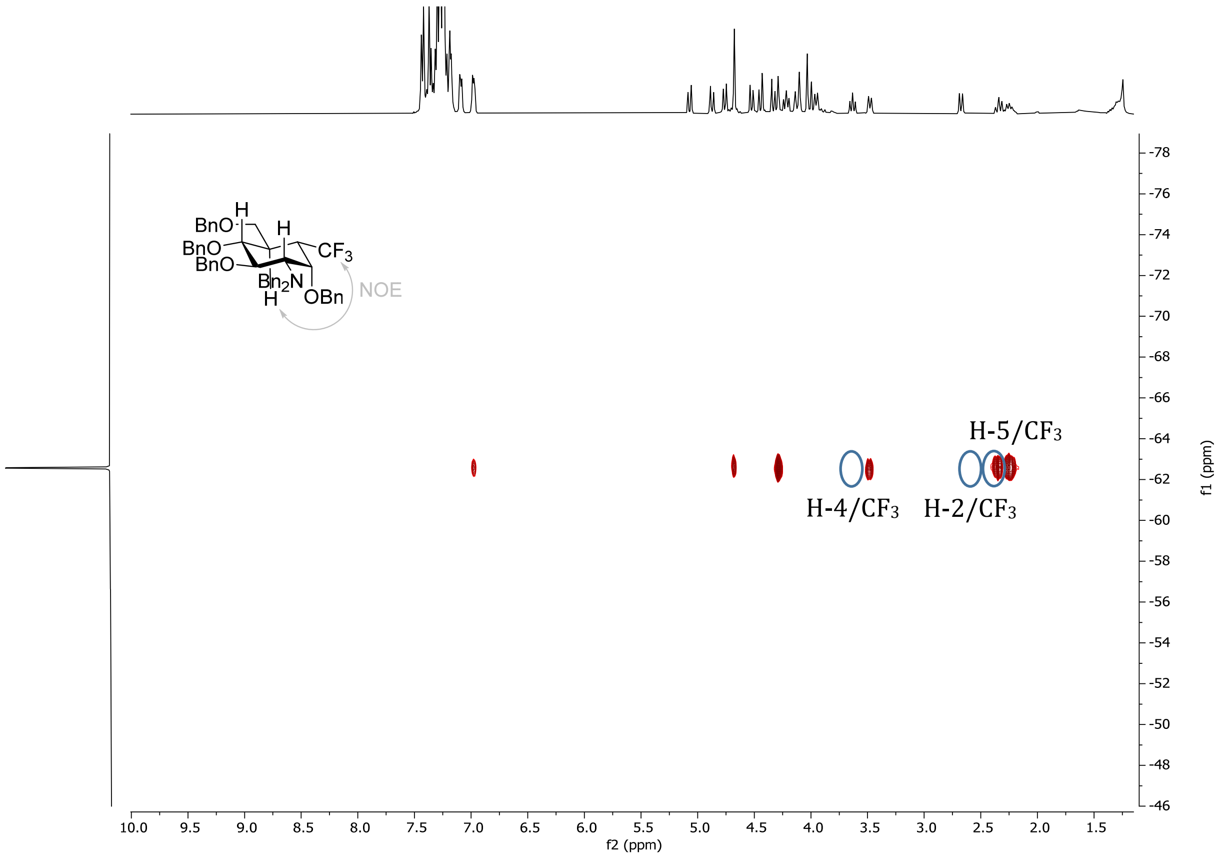

^{1}H-^{19}F HOESY (500 MHz/470 MHz, $CDCl_3$) spectrum of **28.** No crosspeaks between the CF_3 group and H-2 and H-4 are visible.

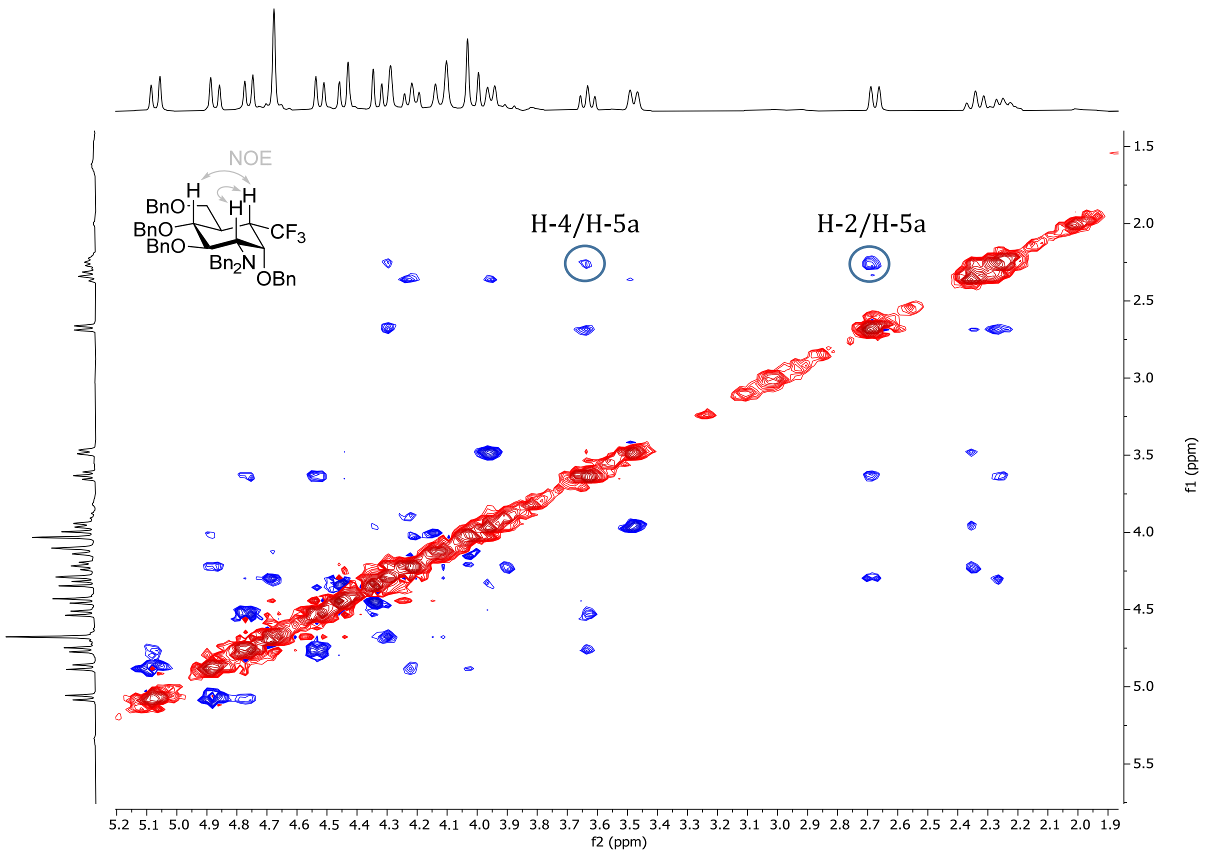

NOESY (500 MHz, $CDCl_3$) spectrum of **28**

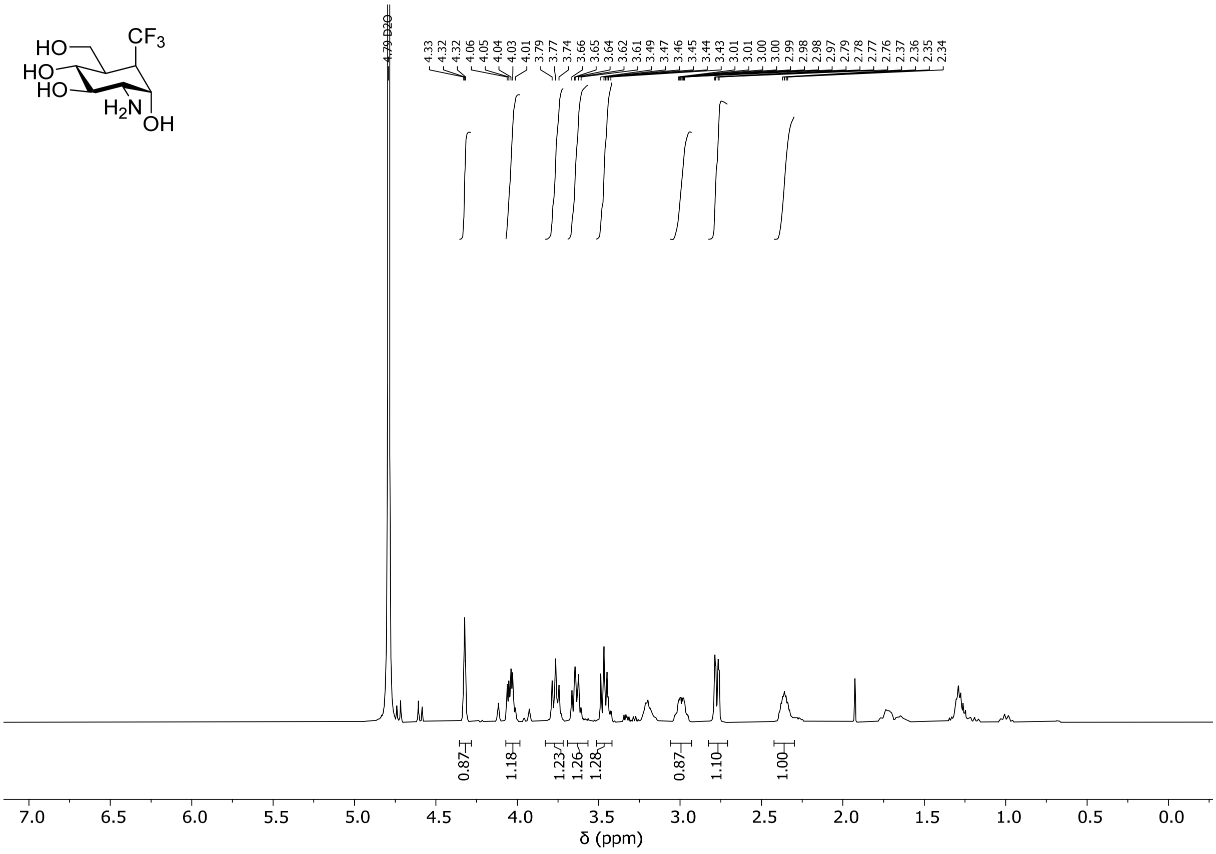

^{1}H NMR spectrum (500 MHz, D_2O) of **29**

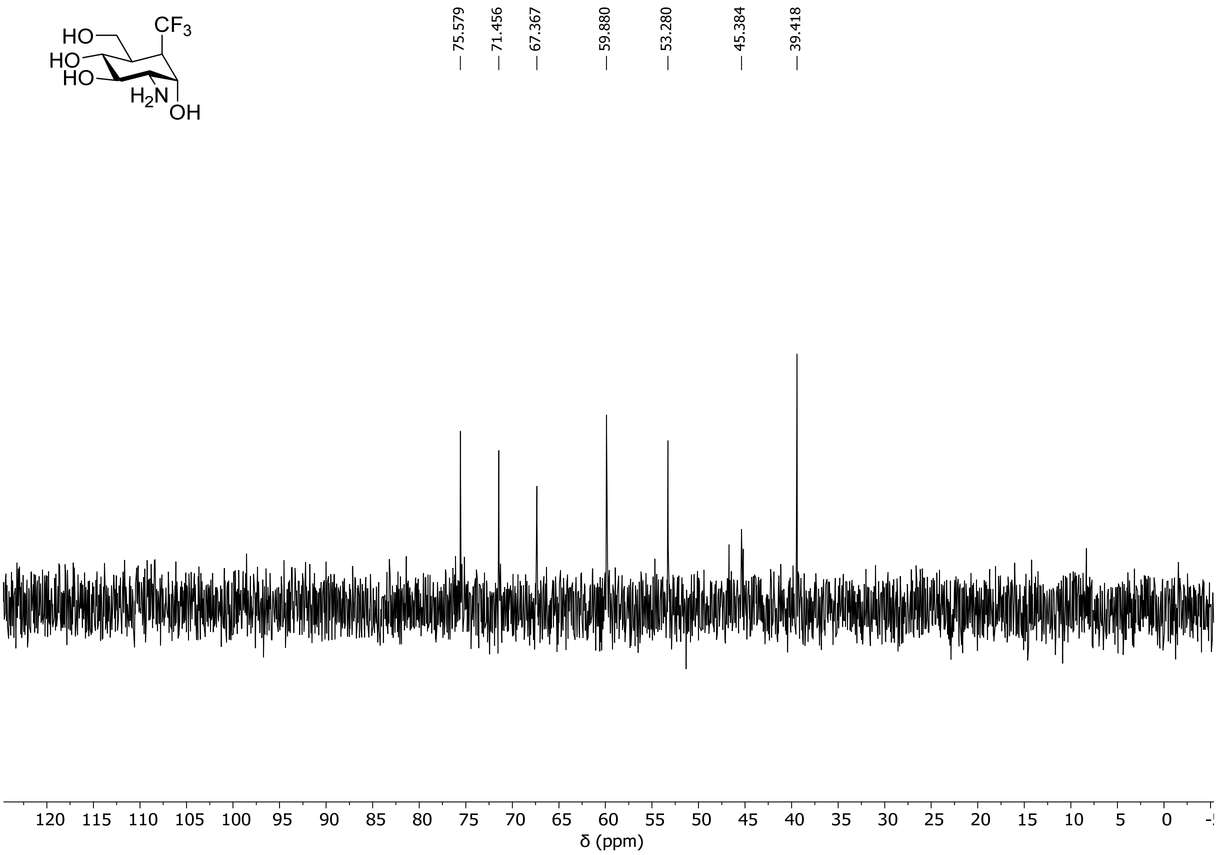

$^{13}C\{^{1}H\}$ NMR spectrum (126 MHz, D_2O) of **29**

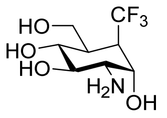

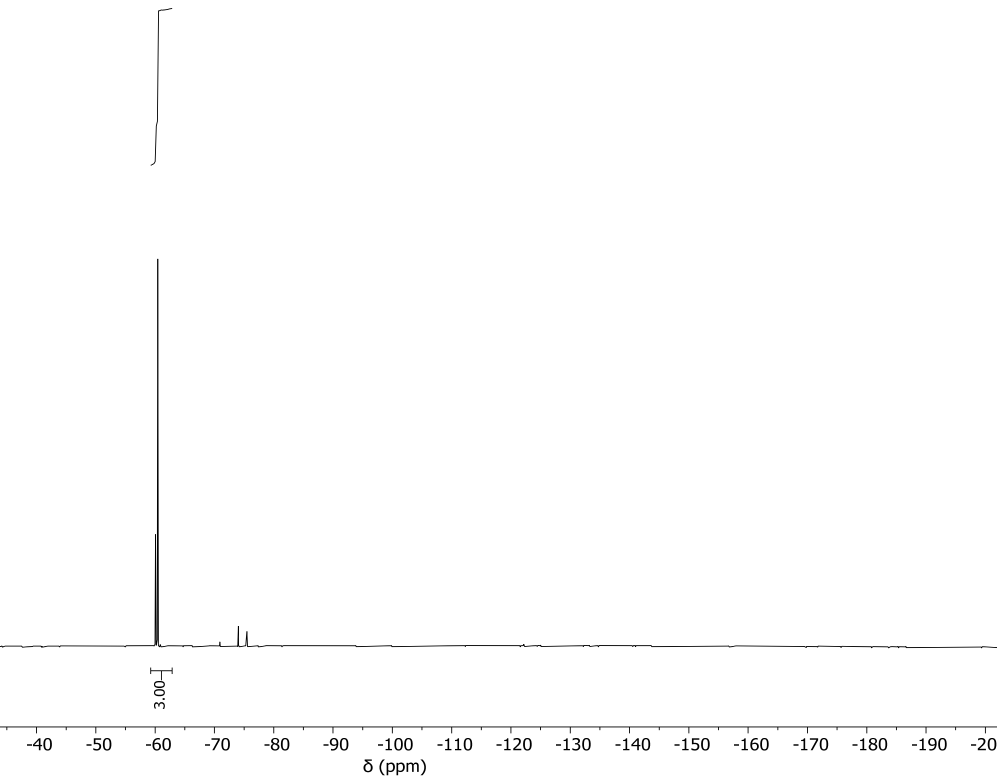

^{19}F NMR spectrum (471 MHz, D_2O) of **29**

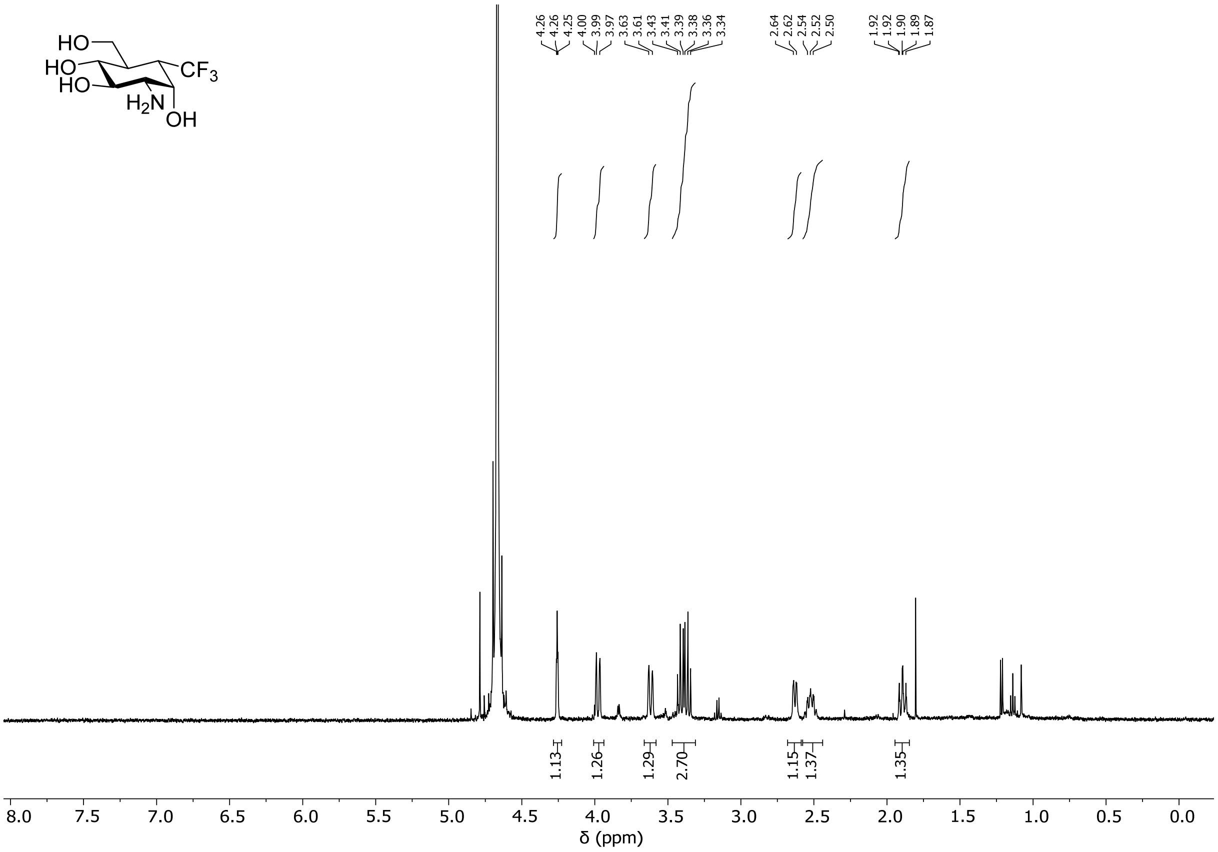

^{1}H NMR spectrum (500 MHz, D_2O) of **30**

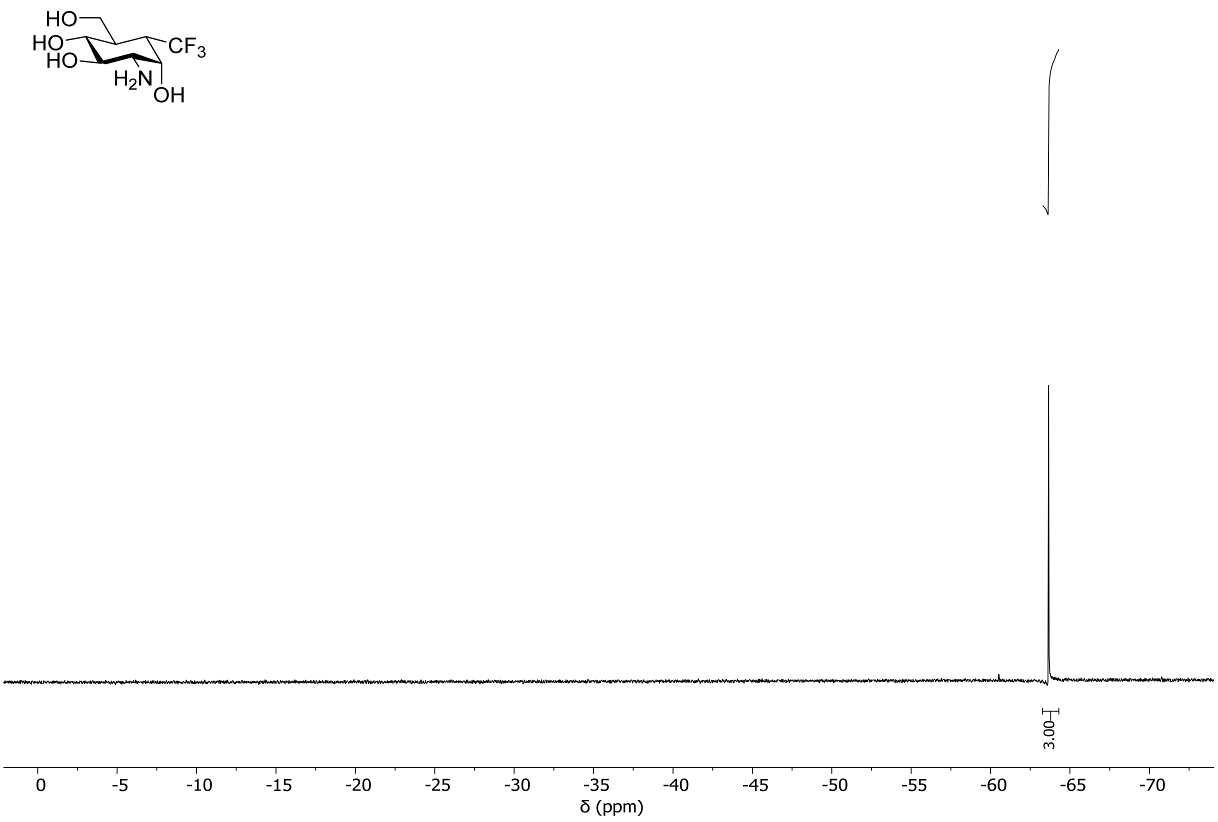

^{19}F NMR spectrum (471 MHz, D_2O) of **30**

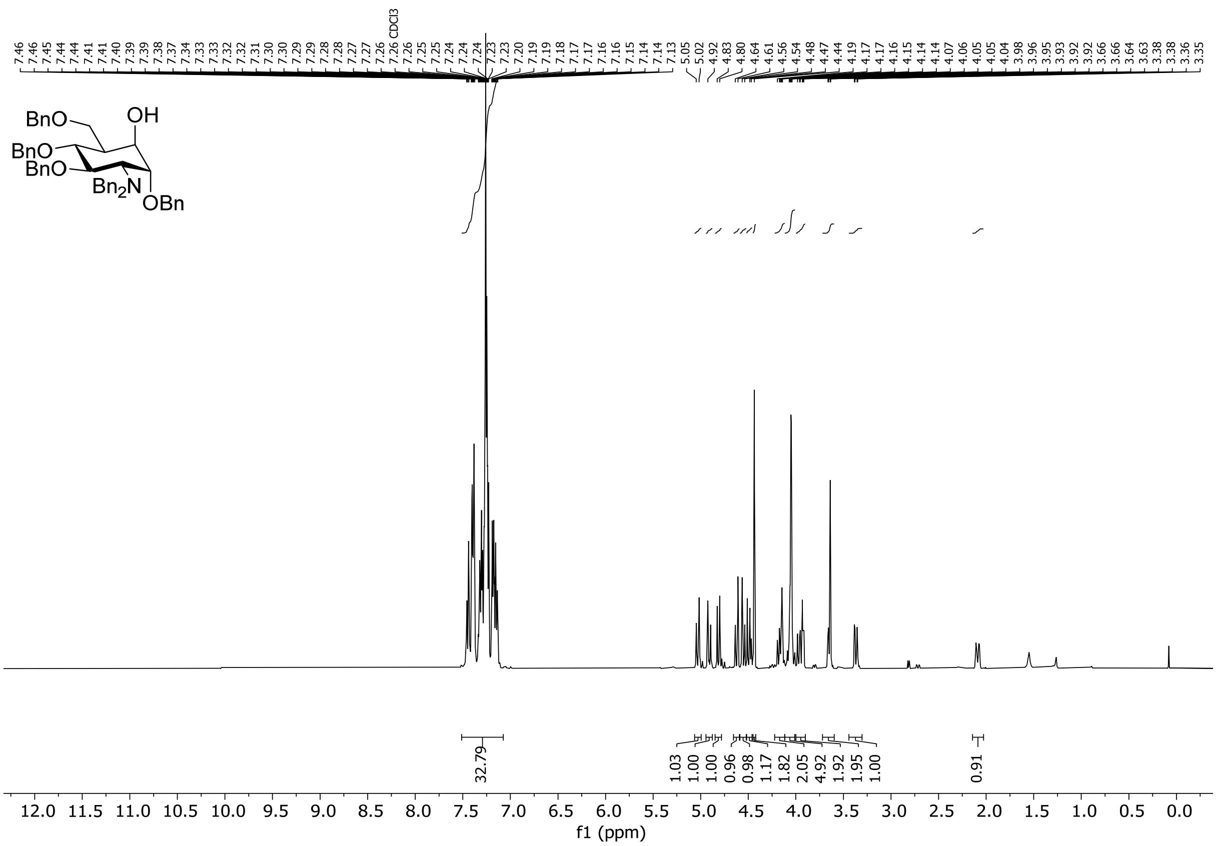

^{1}H NMR spectrum (400 MHz, $CDCl_3$) of **31**

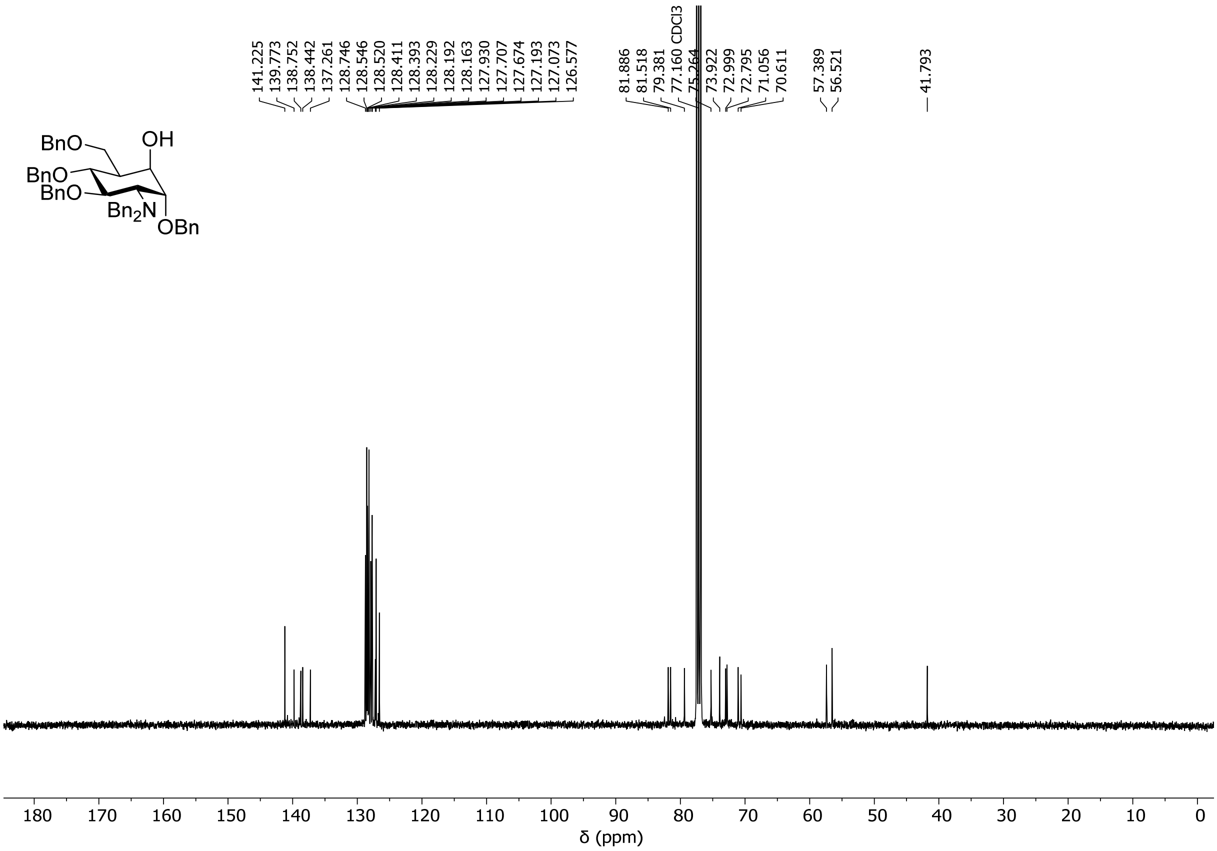

$^{13}C\{^{1}H\}$ NMR spectrum (101 MHz, $CDCl_3$) of **31**

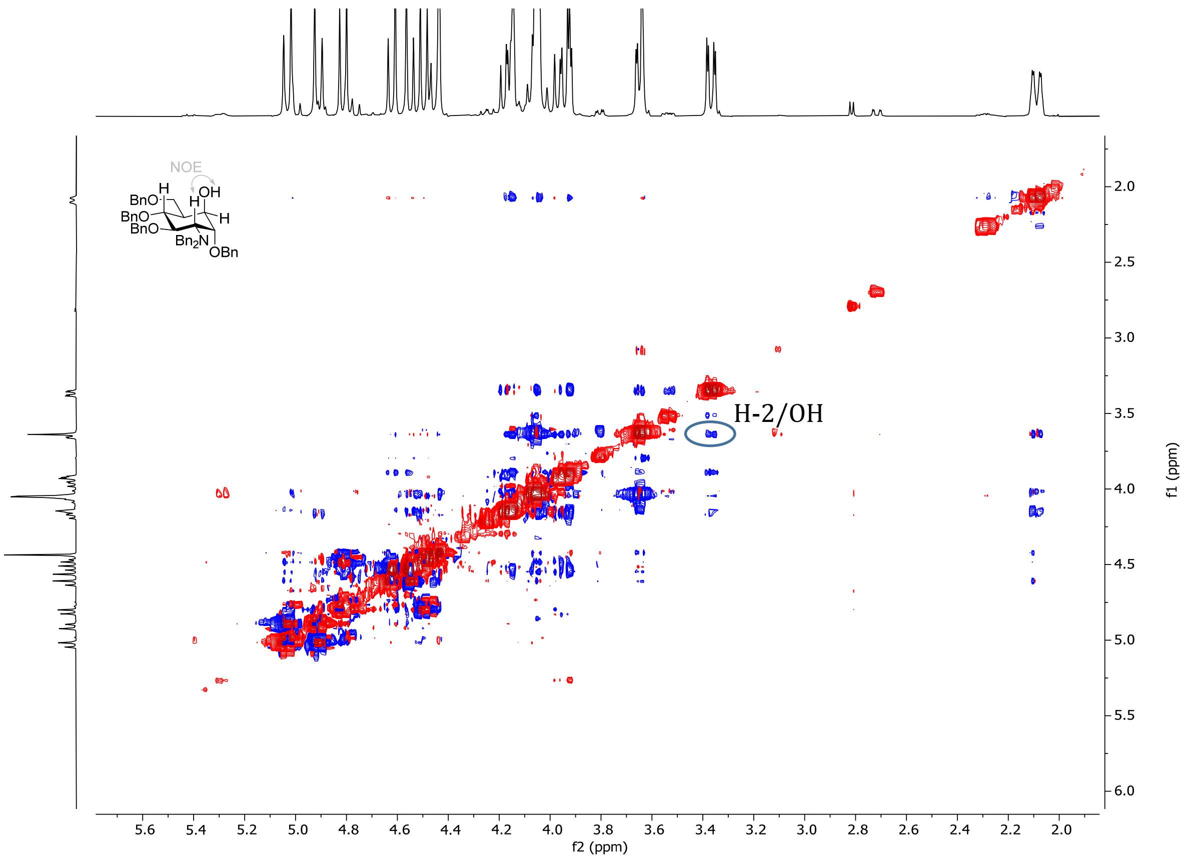

NOESY (400 MHz, $CDCl_3$) spectrum of **31**. Stereochemical assignment at position 5a was further consolidated by comparison to the published spectra of previously published compound **6**[2] featuring an equatorial hydroxy group.

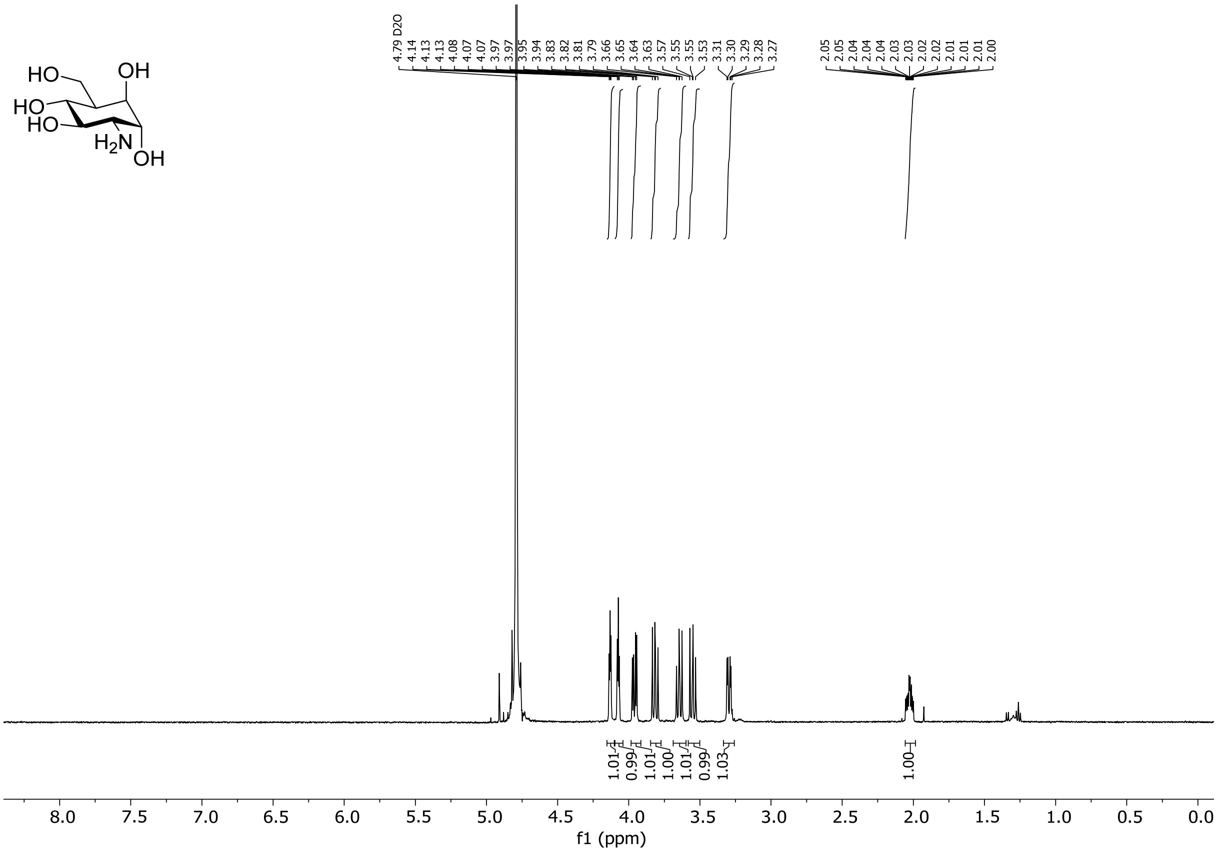

^{1}H NMR spectrum (500 MHz, D_2O) of **32**

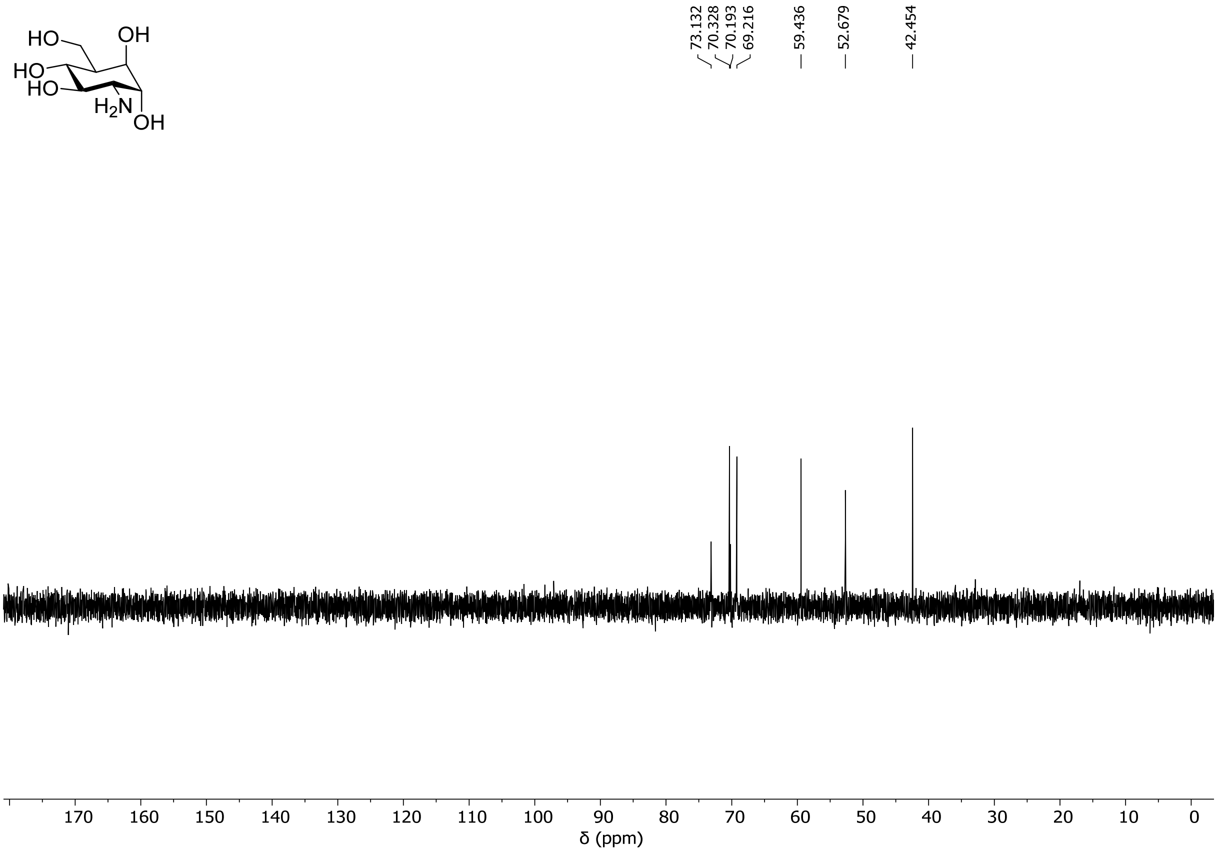

$^{13}C\{^{1}H\}$ NMR spectrum (101 MHz, D_2O) of **32**

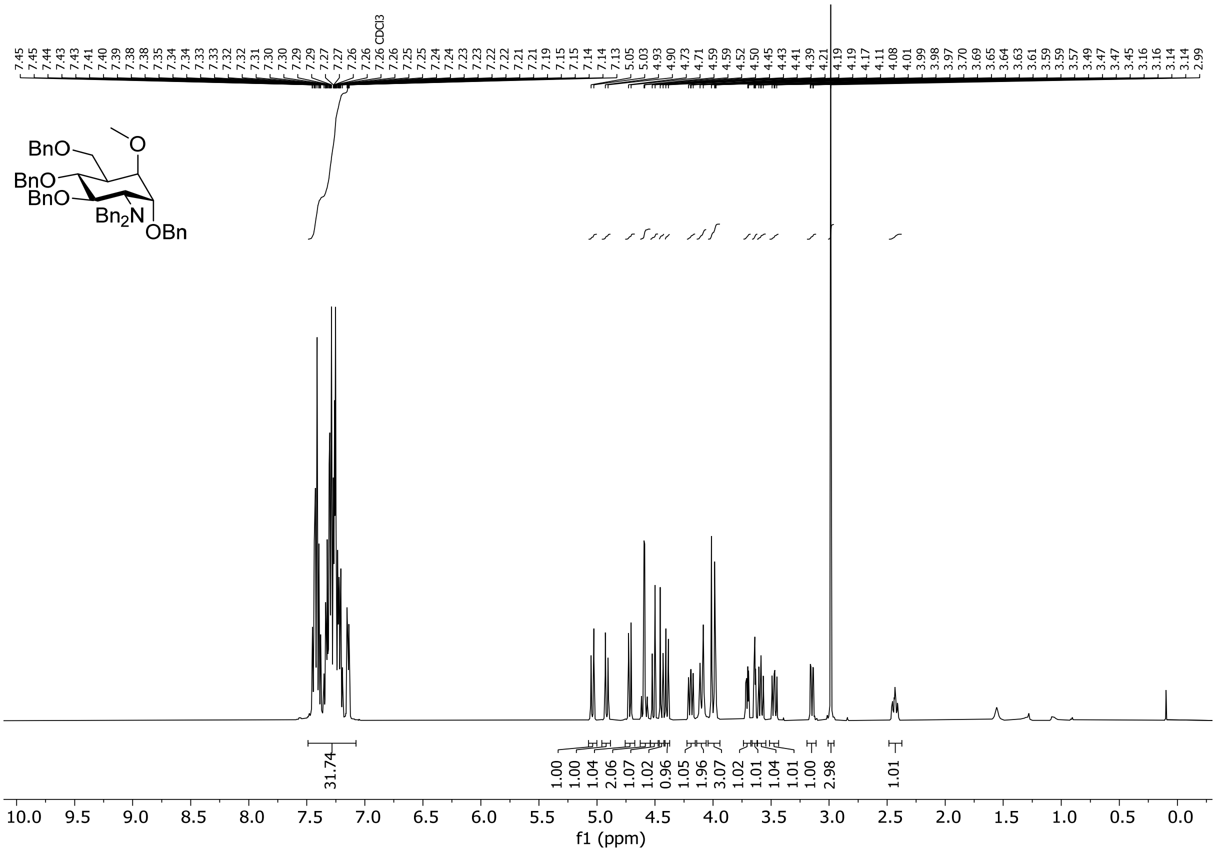

^{1}H NMR spectrum (500 MHz, $CDCl_3$) of **33**

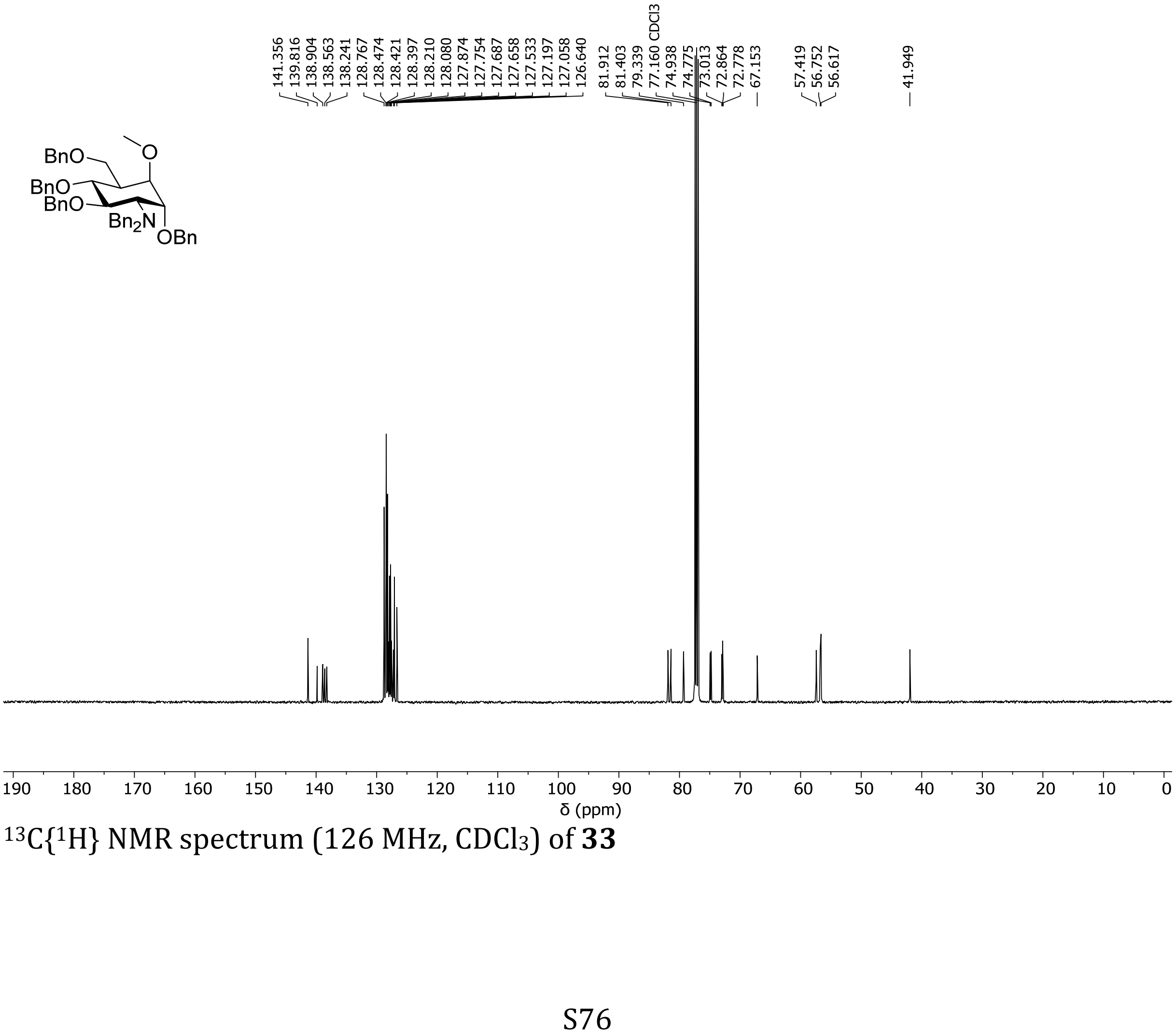

$^{13}C\{^{1}H\}$ NMR spectrum (126 MHz, $CDCl_3$) of **33**

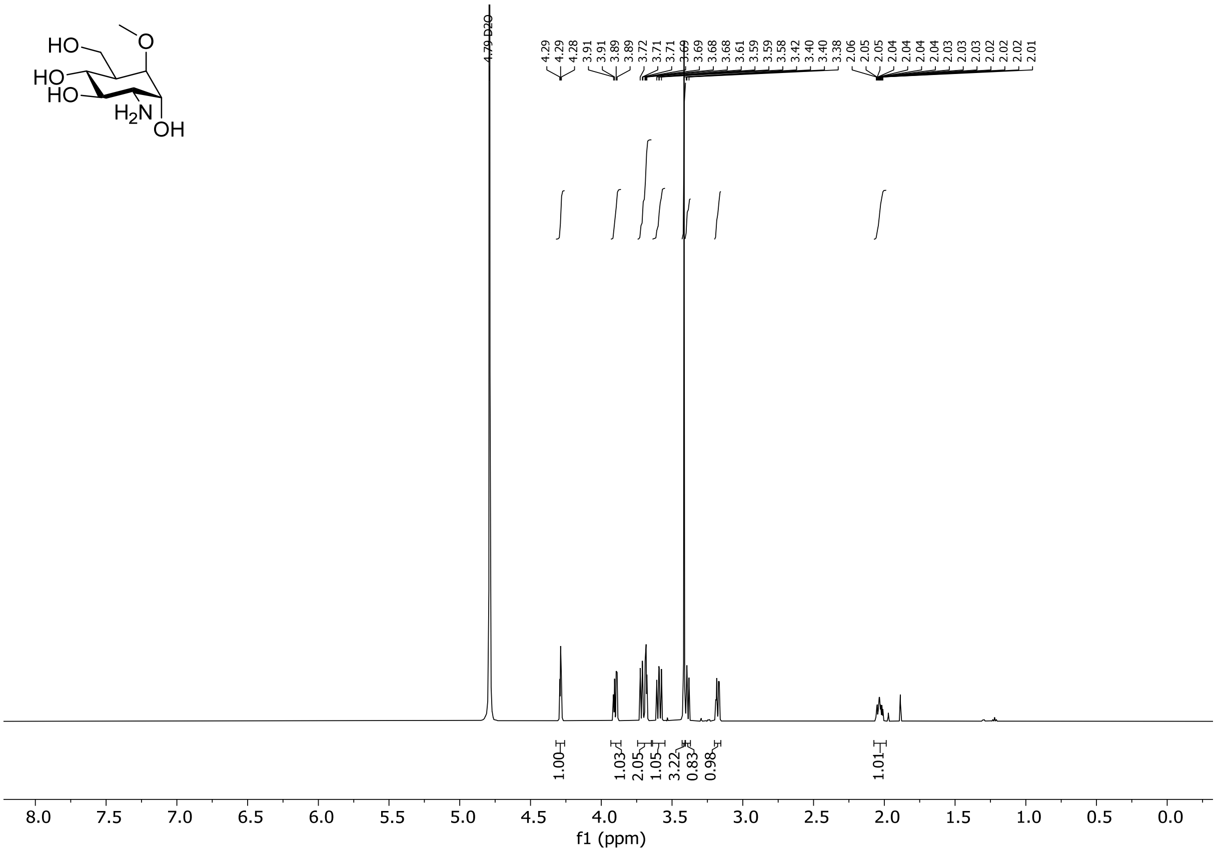

^{1}H NMR spectrum (600 MHz, D_2O) of **34**

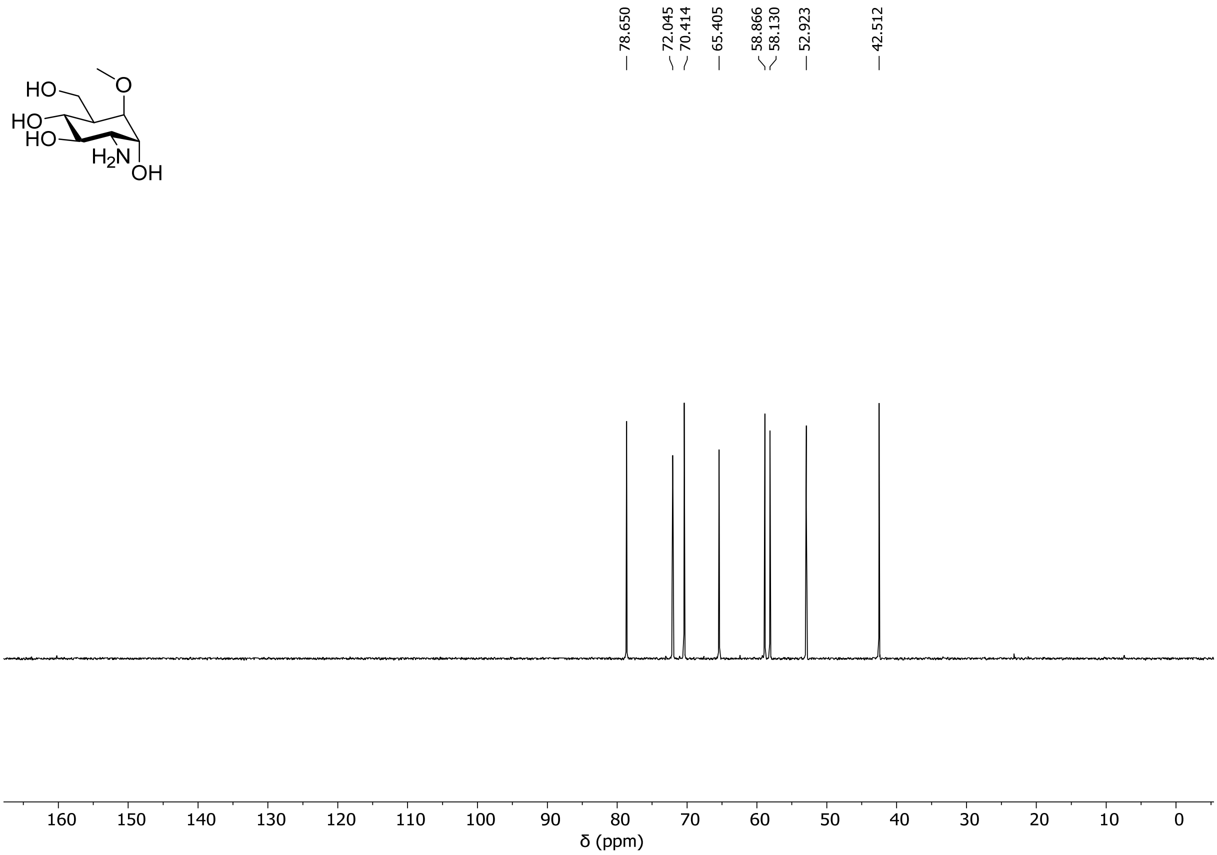

^{13}C{^{1}H} NMR spectrum (150 MHz, D_2O) of **34**

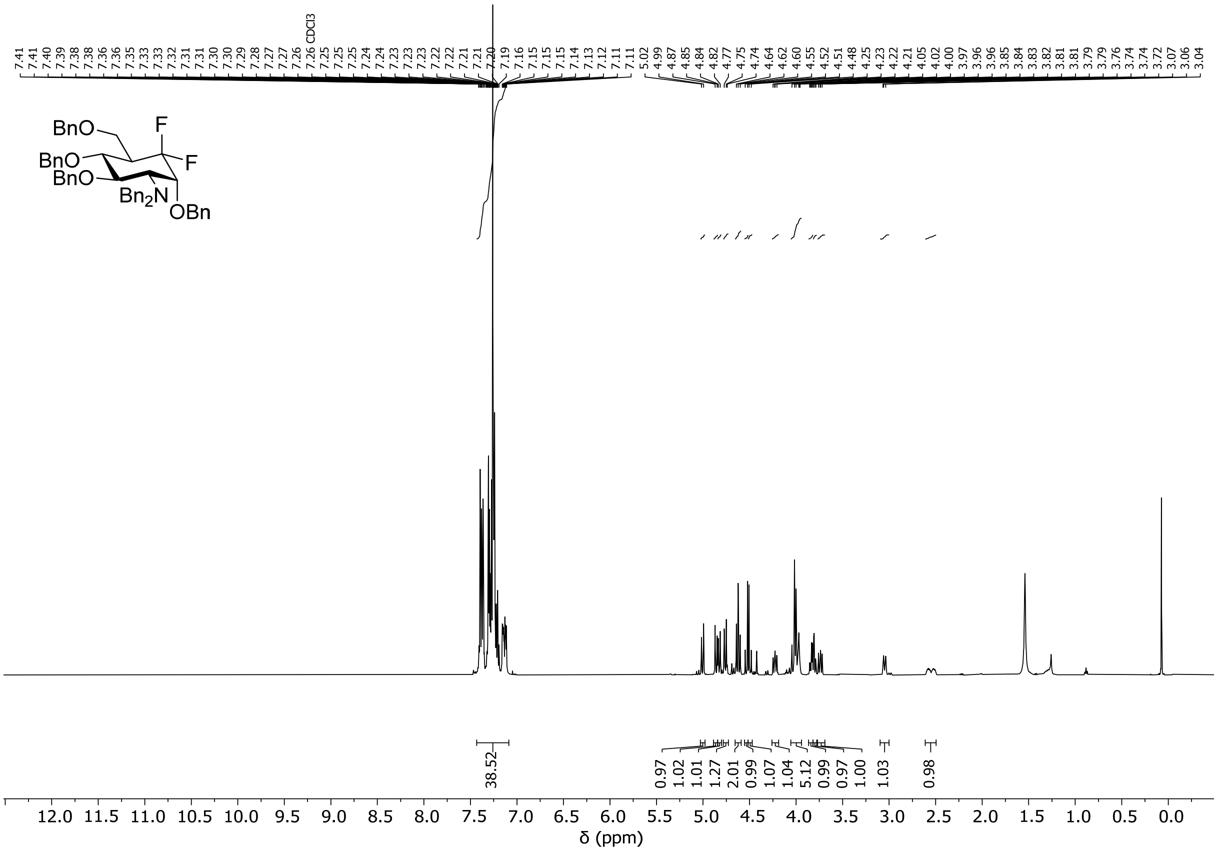

^{1}H NMR spectrum (500 MHz, $CDCl_3$) of **35**

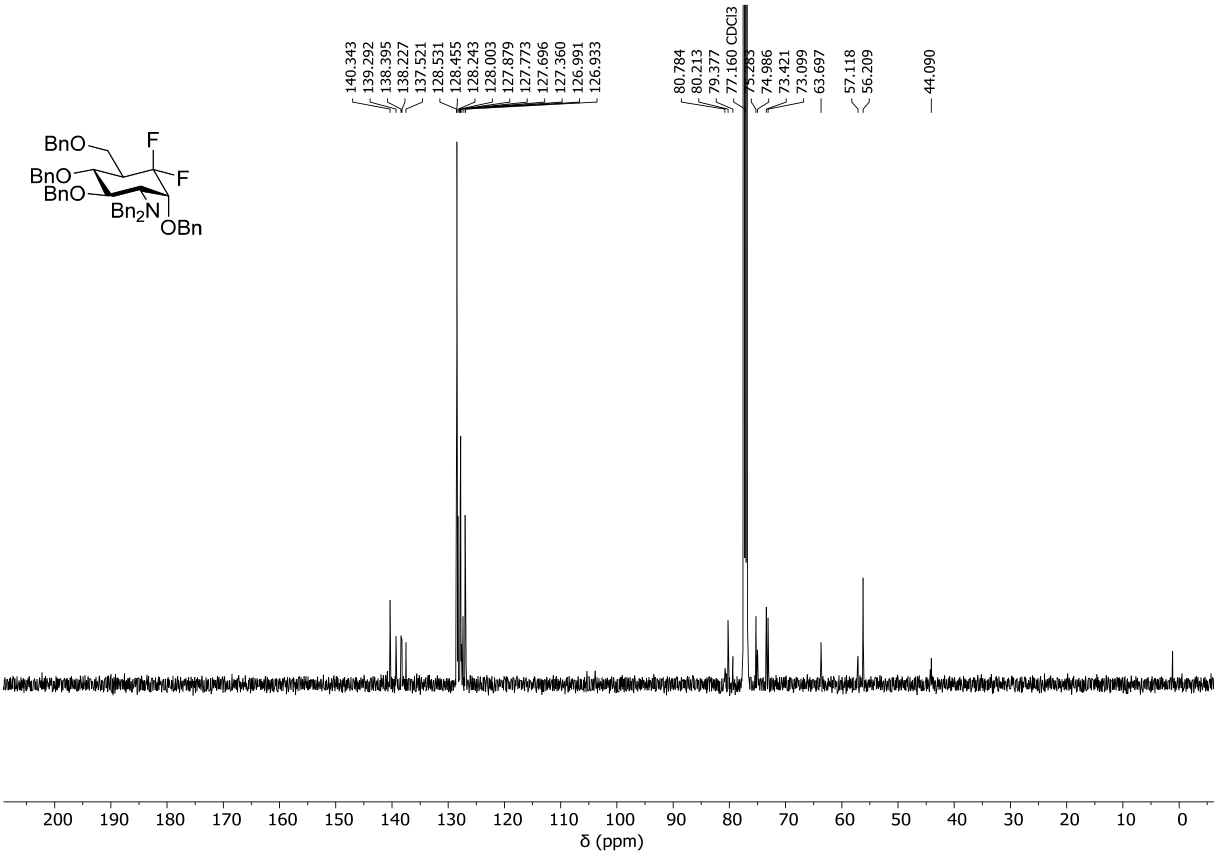

$^{13}C\{^1H\}$ NMR spectrum (126 MHz, $CDCl_3$) of **35**

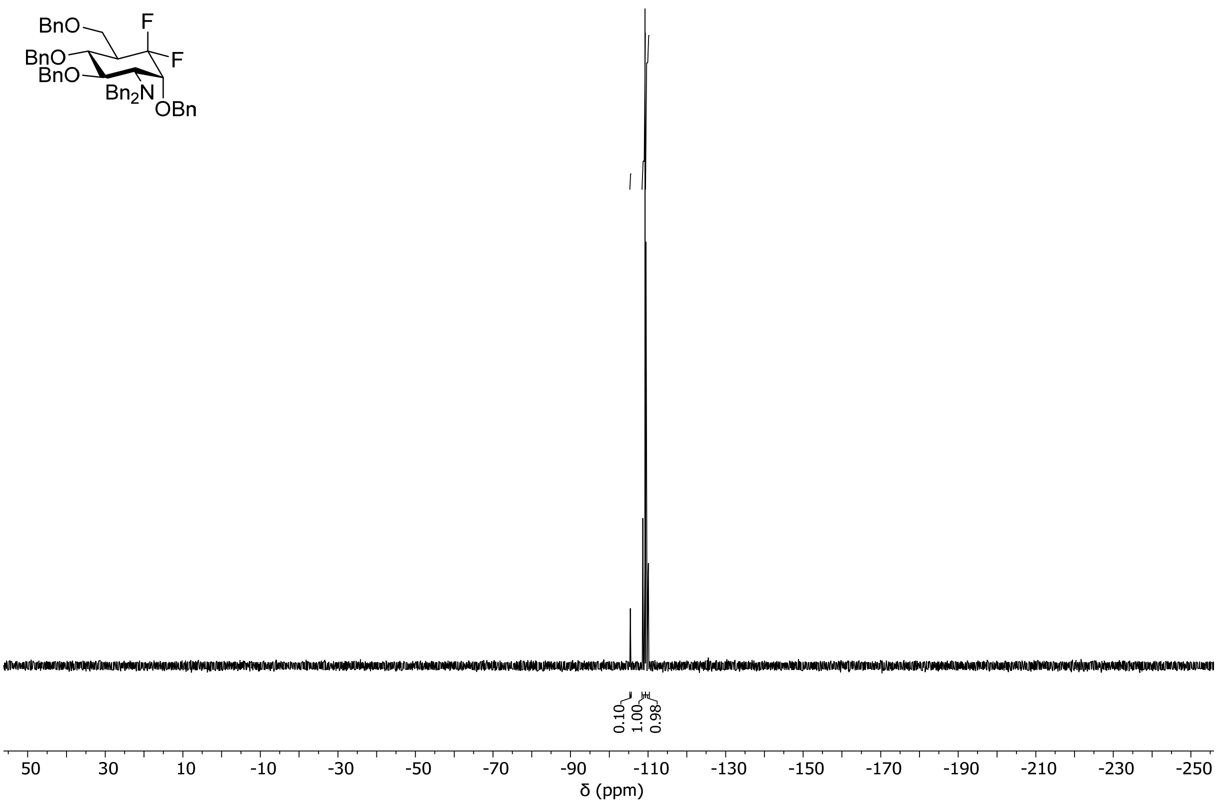

^{19}F NMR spectrum (471 MHz, $CDCl_3$) of **35**

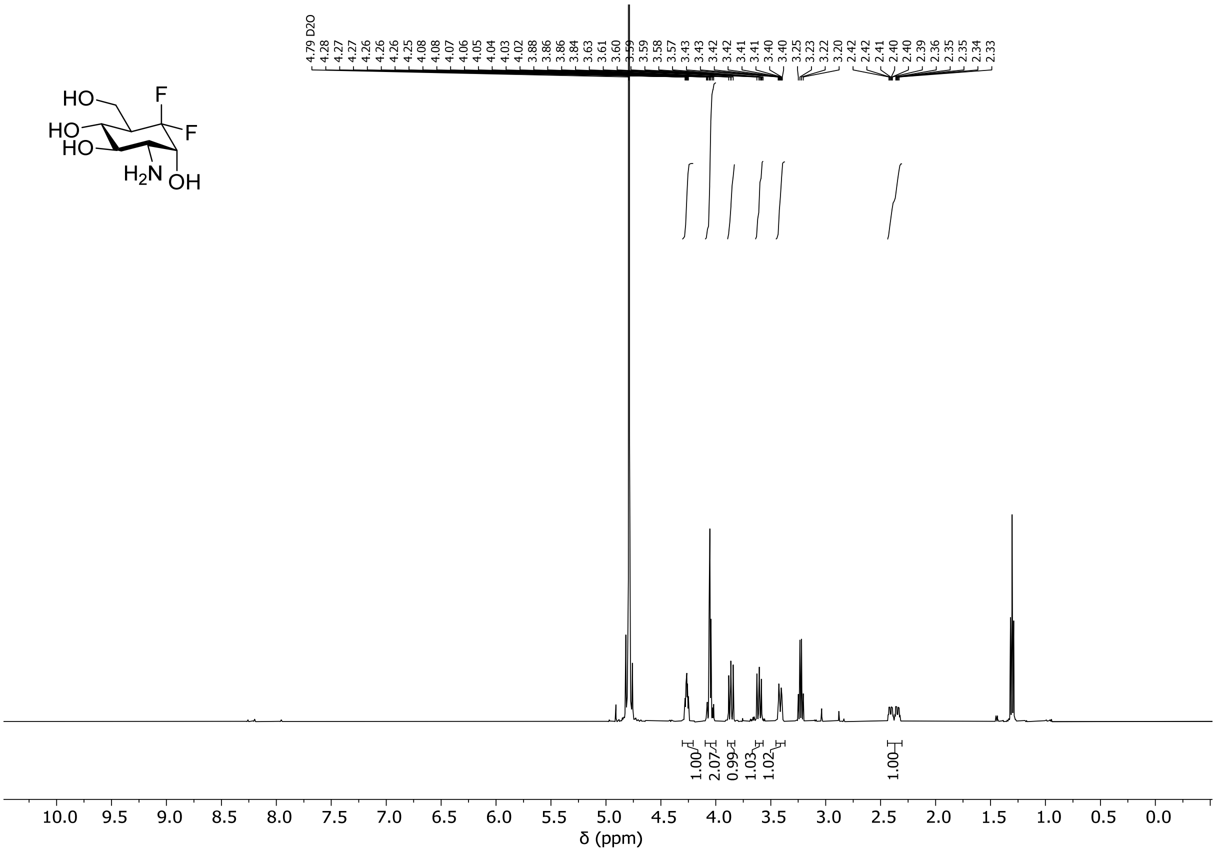

^{1}H NMR spectrum (500 MHz, D_2O) of **37**

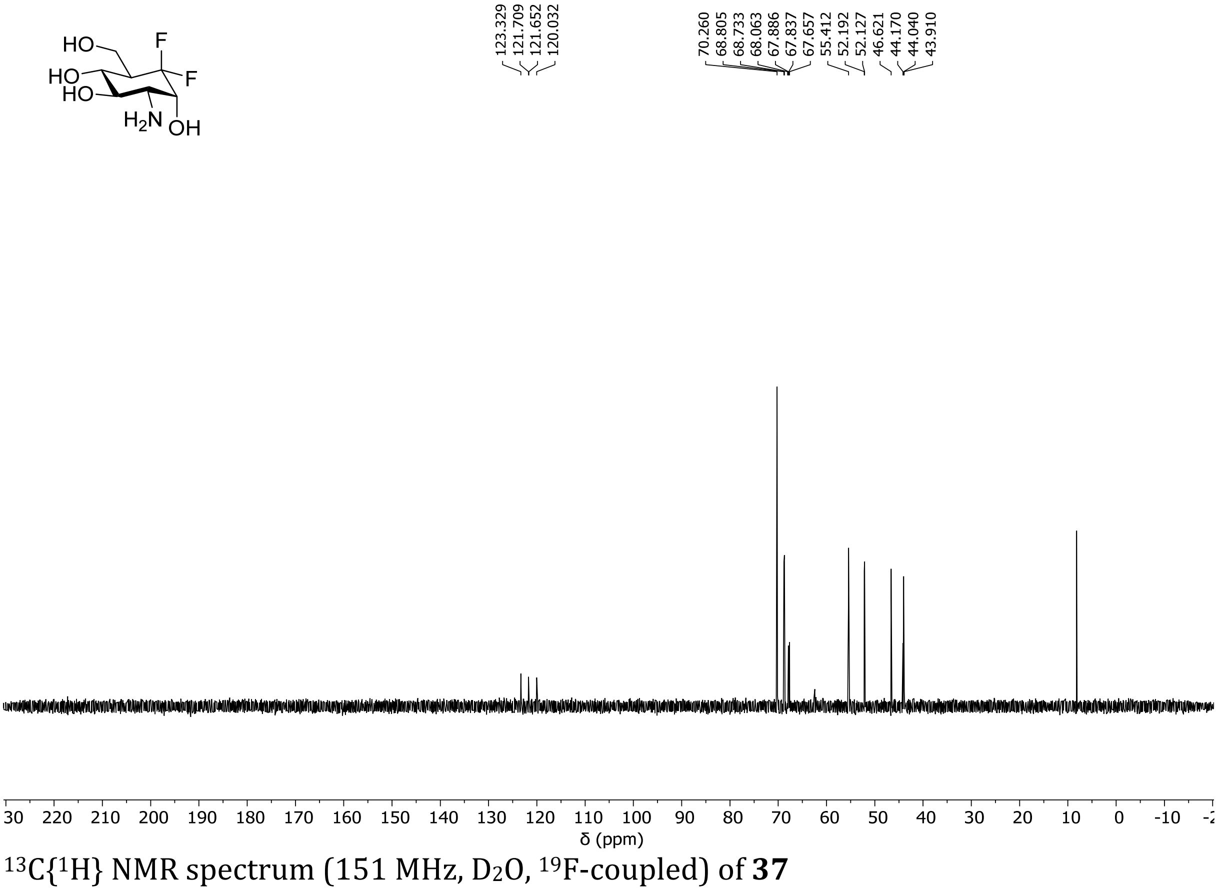

$^{13}C\{^{1}H\}$ NMR spectrum (151 MHz, D_2O, ^{19}F-coupled) of **37**

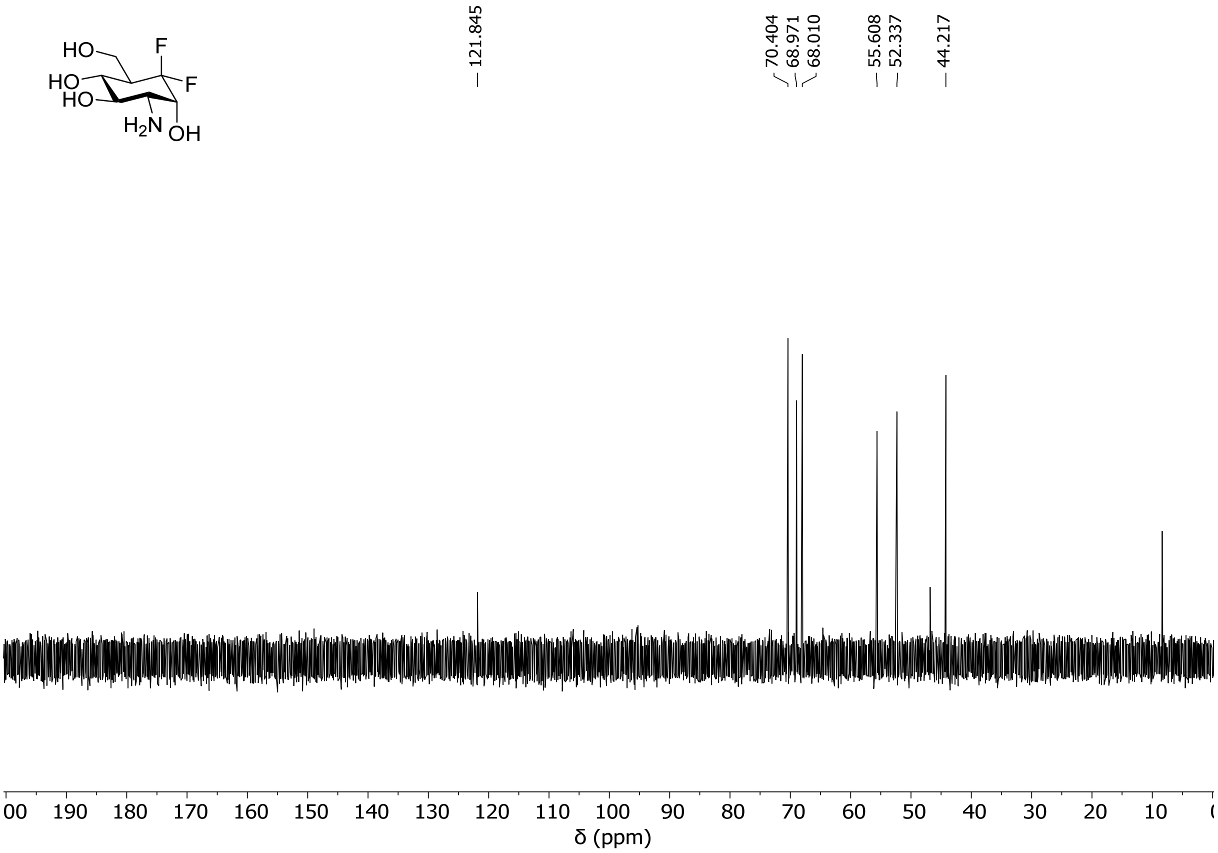

$^{13}C\{^1H\}$ NMR spectrum (126 MHz, D_2O, ^{19}F-decoupled) of **37**

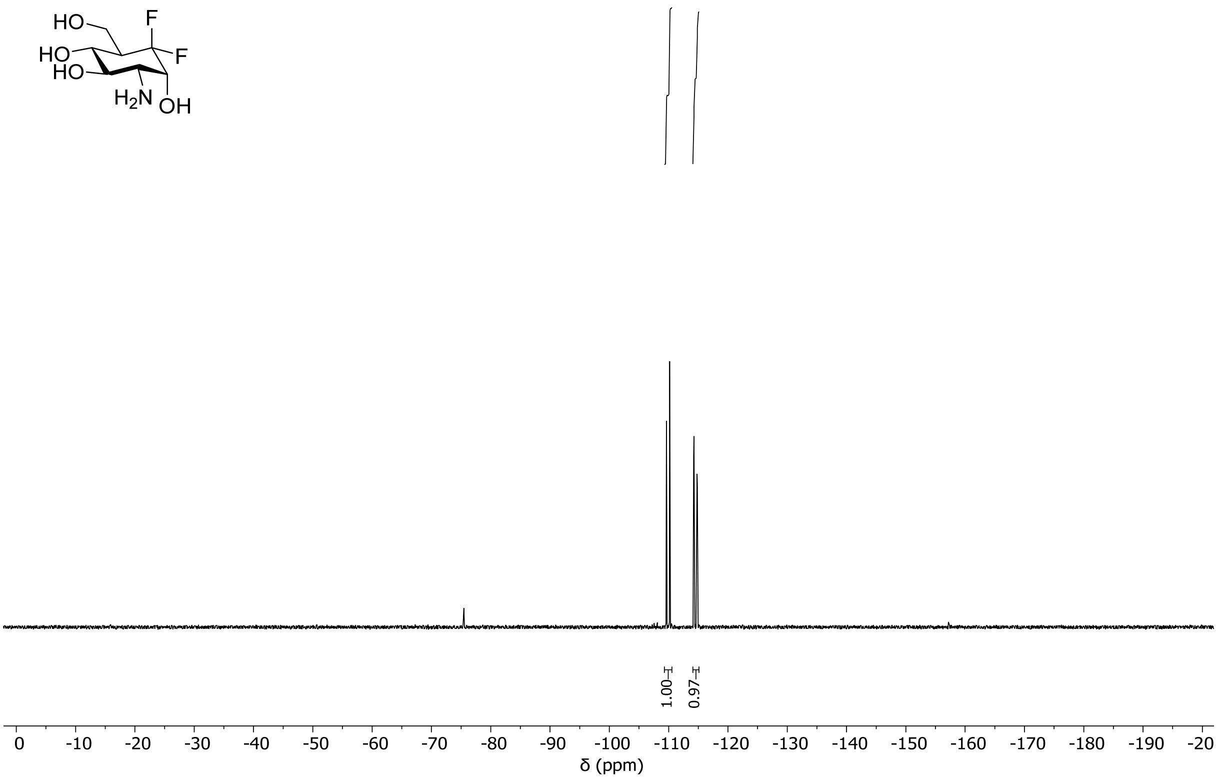

^{19}F NMR spectrum (471 MHz, D_2O) of **37**

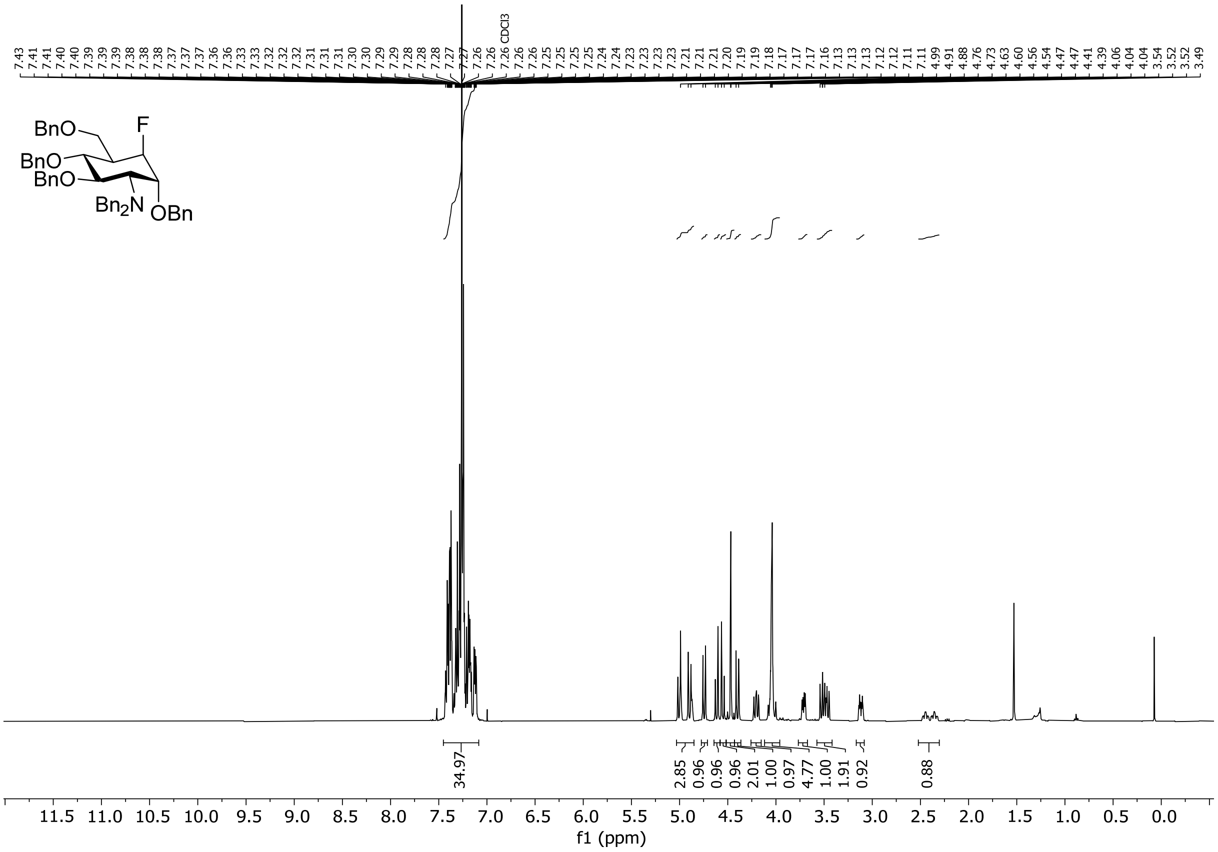

^{1}H NMR spectrum (400 MHz, $CDCl_3$) of **39**

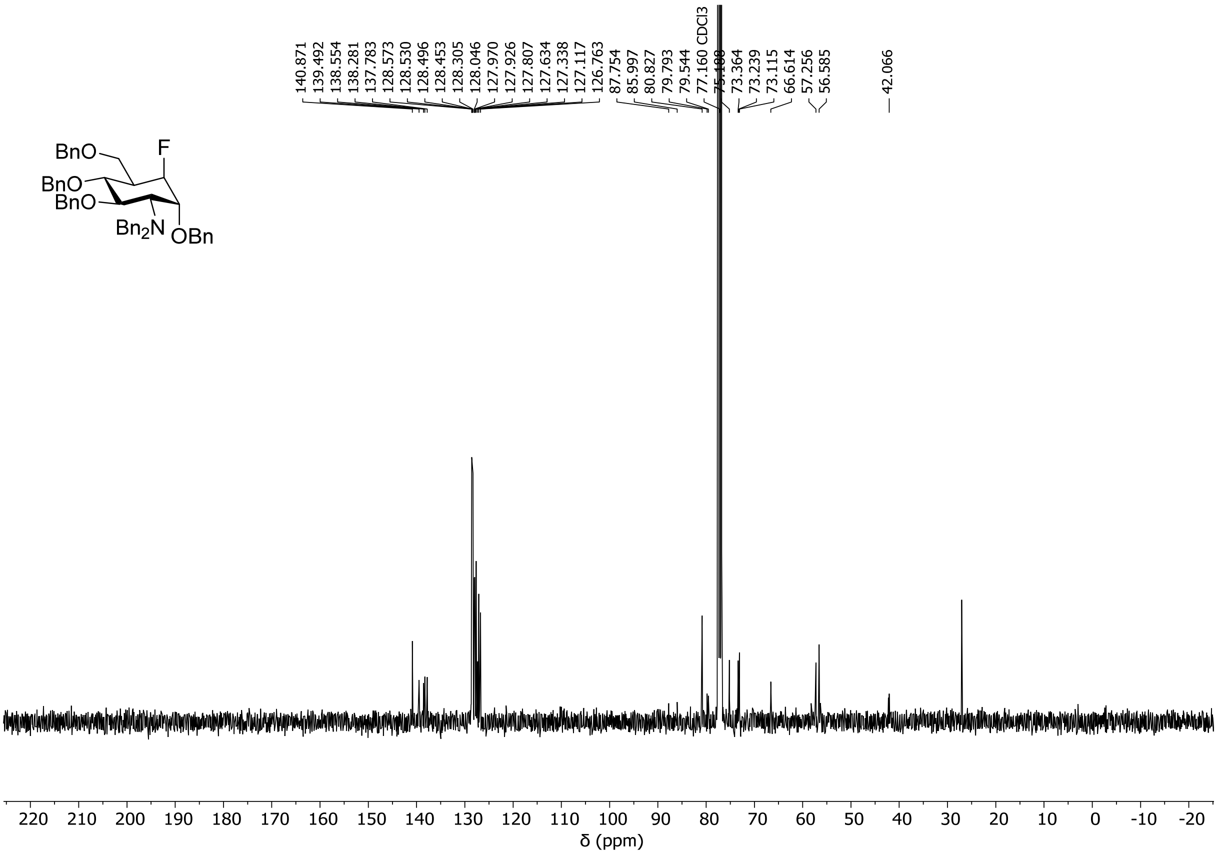

$^{13}C\{^{1}H\}$ NMR spectrum (101 MHz, $CDCl_3$) of **39**

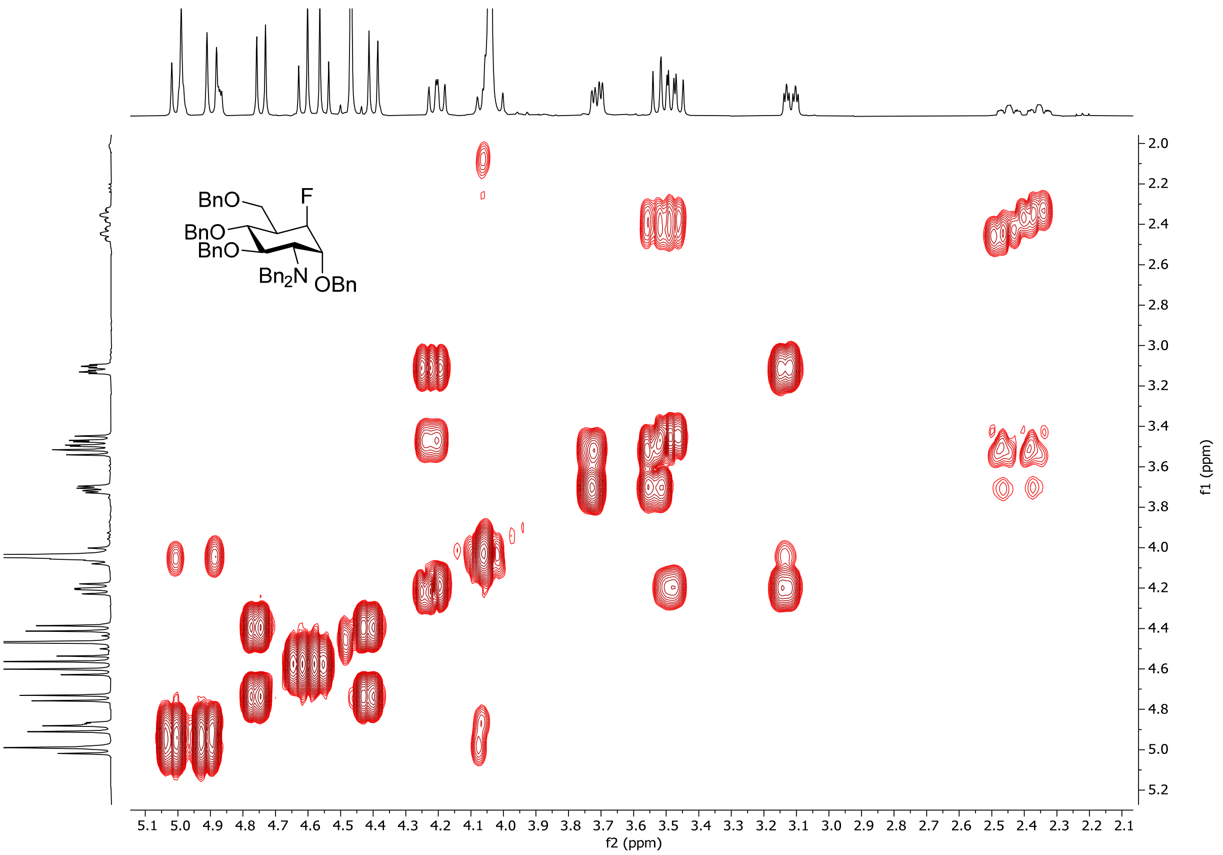

COSY spectrum (400 MHz, CDCl3) of **39**

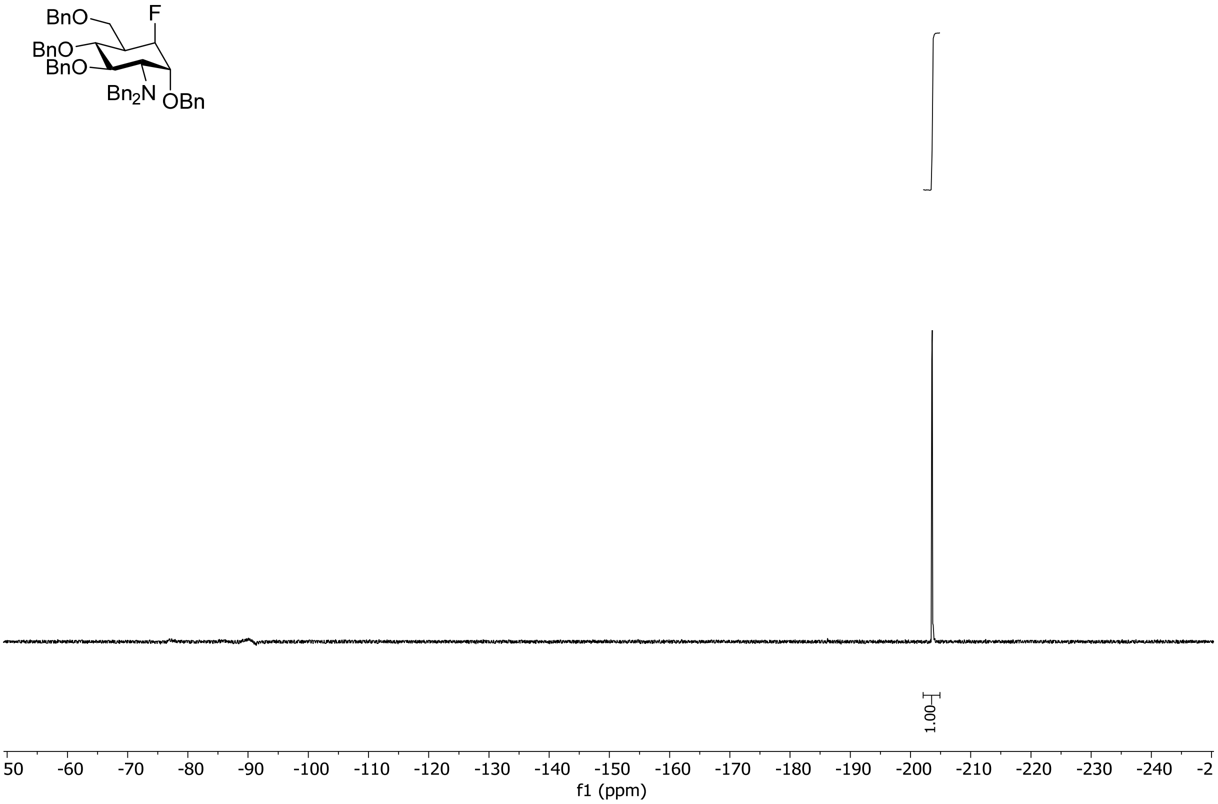

^{19}F NMR spectrum (377 MHz, CDCl3) of **39**

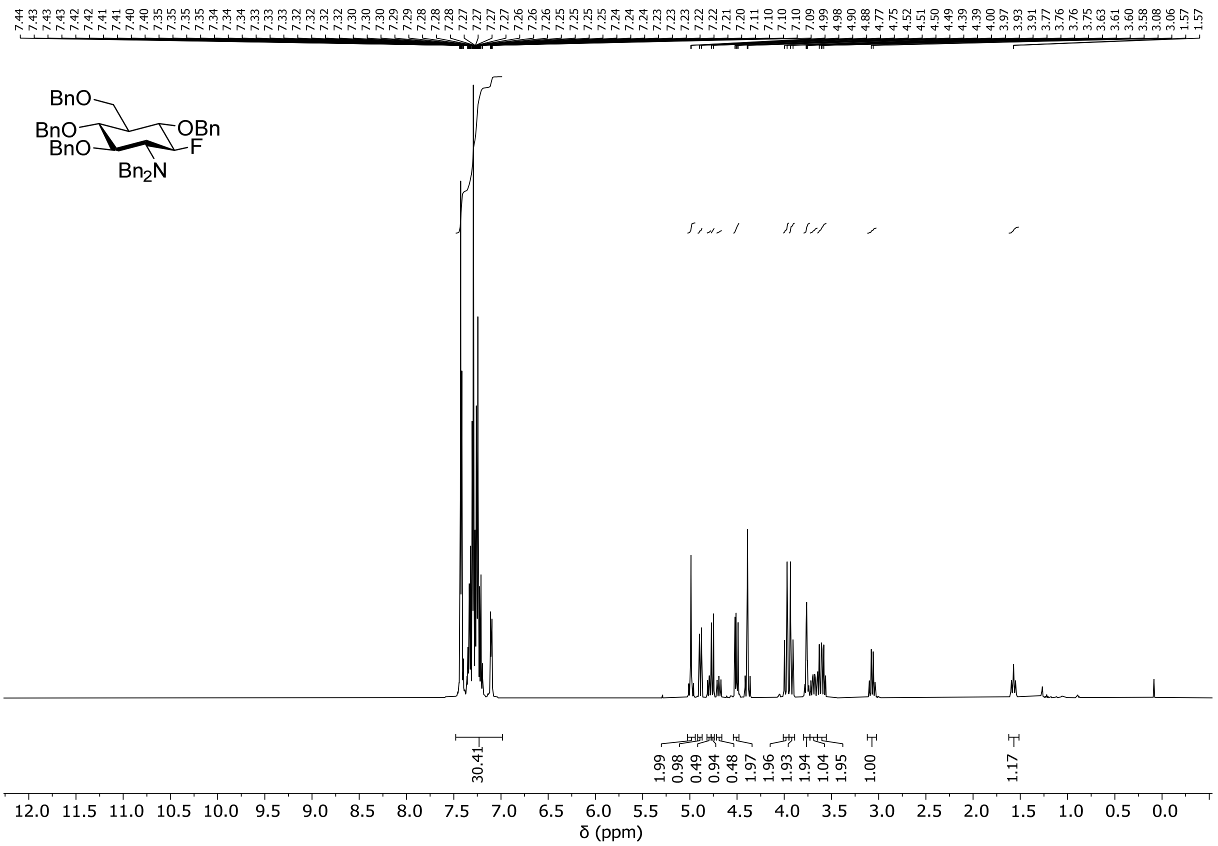

^{1}H NMR spectrum (500 MHz, $CDCl_3$) of **40**

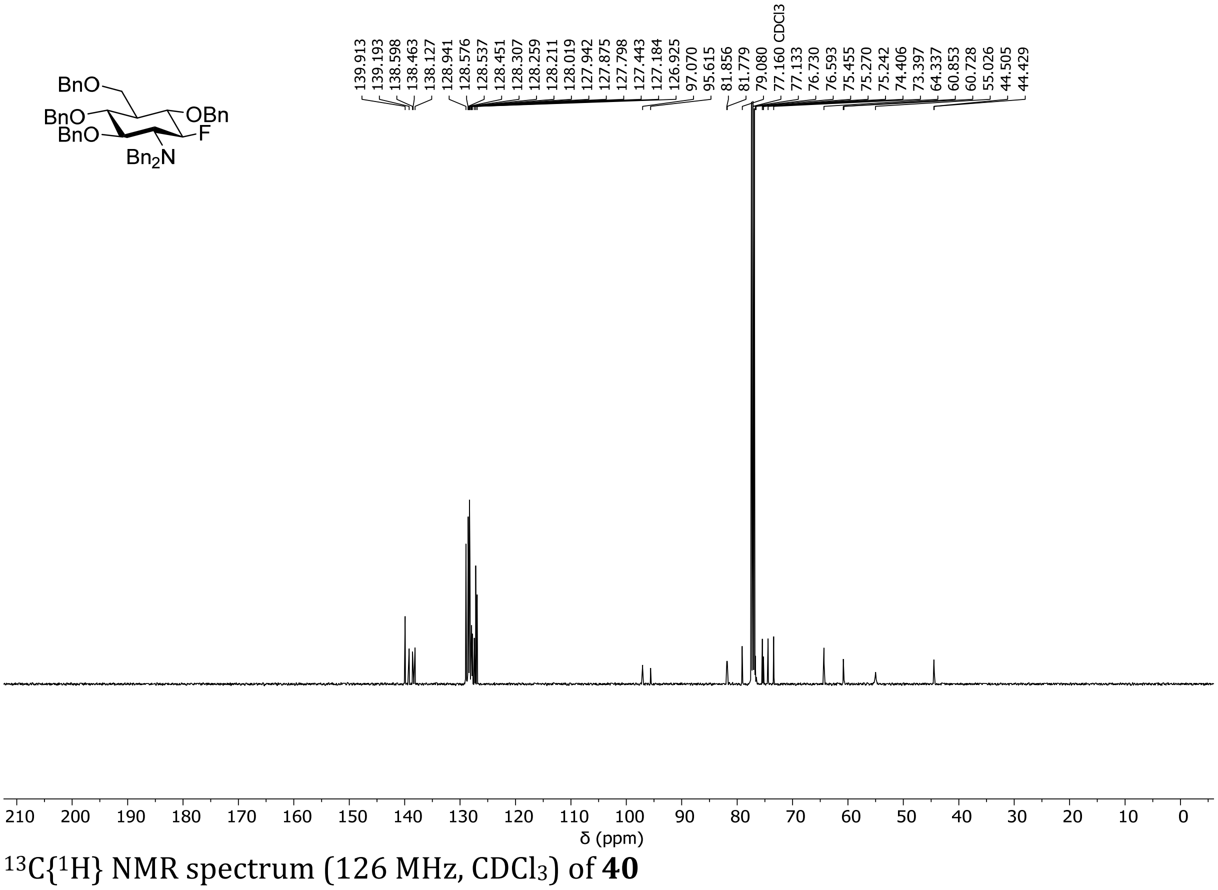

^{13}C{^{1}H} NMR spectrum (126 MHz, $CDCl_3$) of **40**

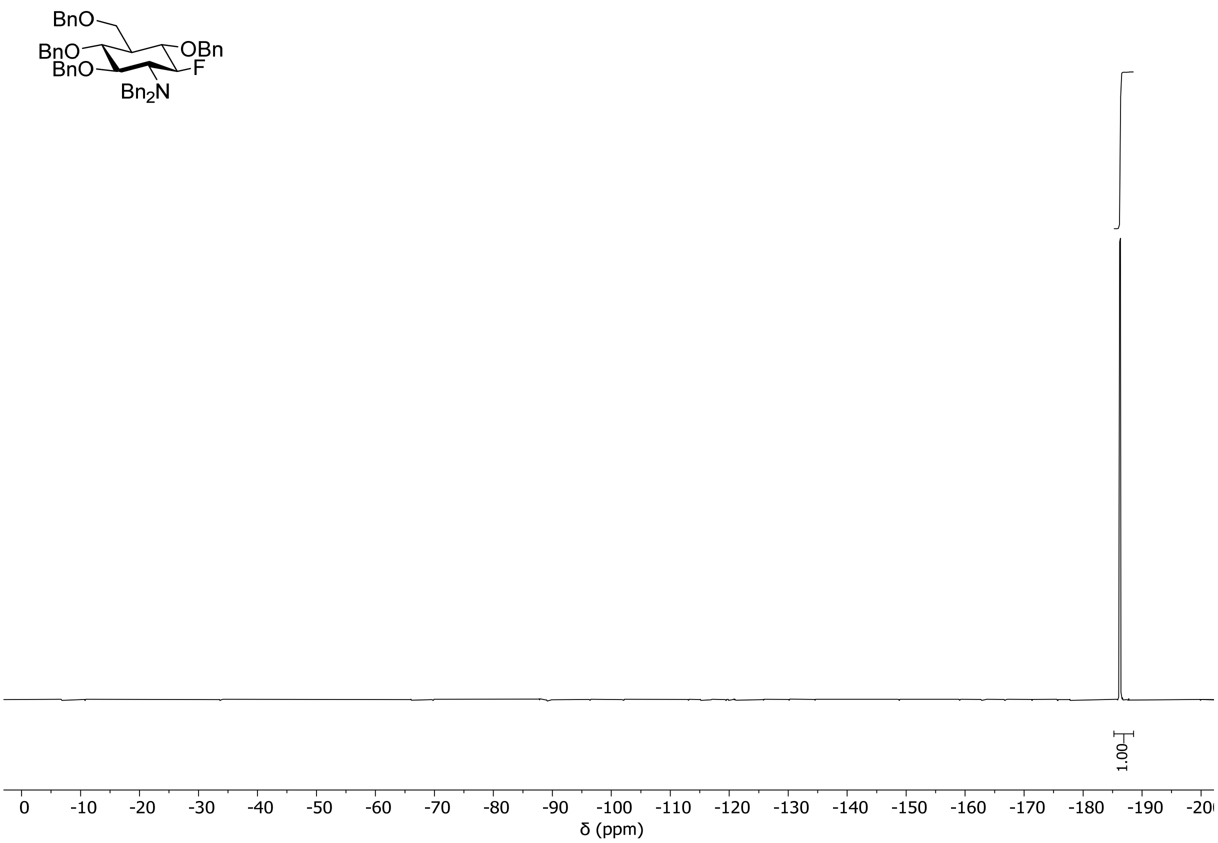

^{19}F NMR spectrum (377 MHz, $CDCl_3$) of **40**

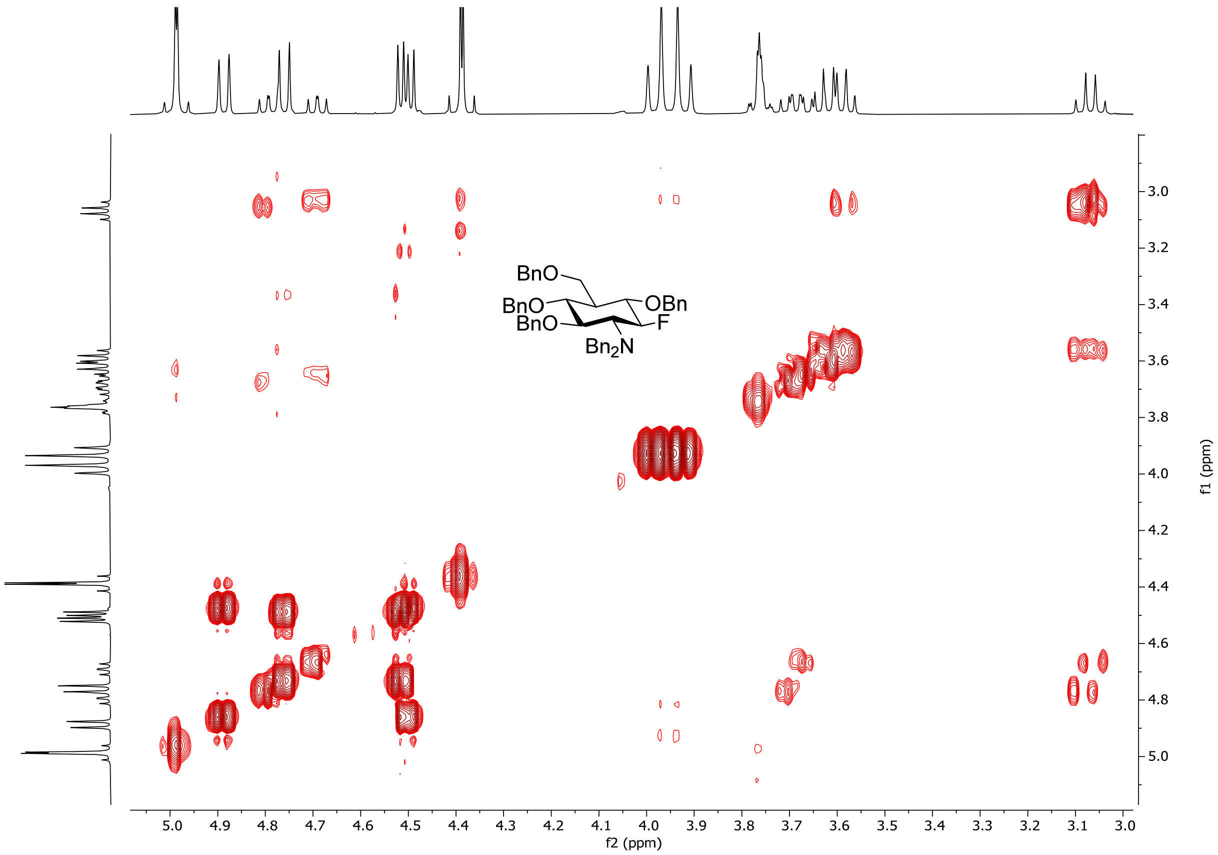

COSY spectrum (500 MHz, $CDCl_3$) of **40**

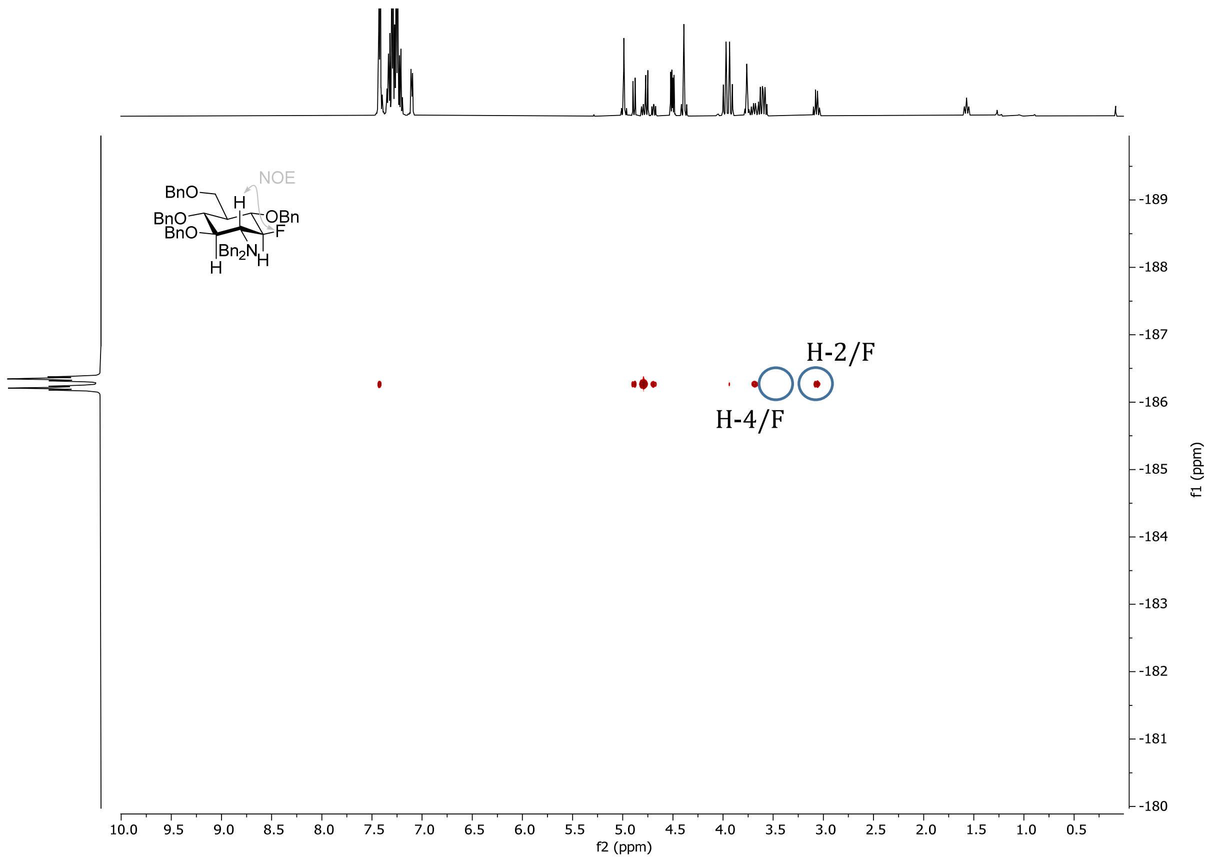

^{1}H-^{19}F HOESY spectrum (500 MHz/470 MHz, $CDCl_3$) of **40**. No cross peak is visible between H-4 and F.

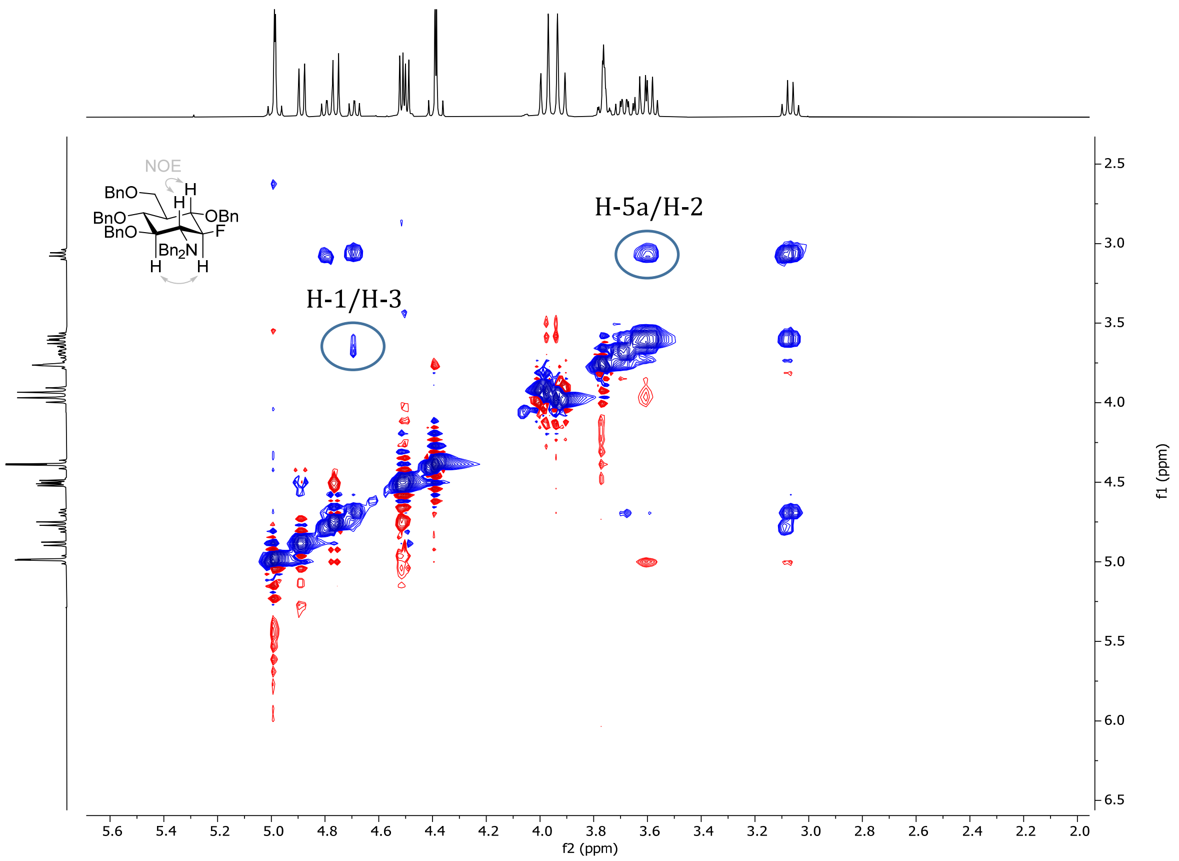

ROESY spectrum (500 MHz, $CDCl_3$) of **40**

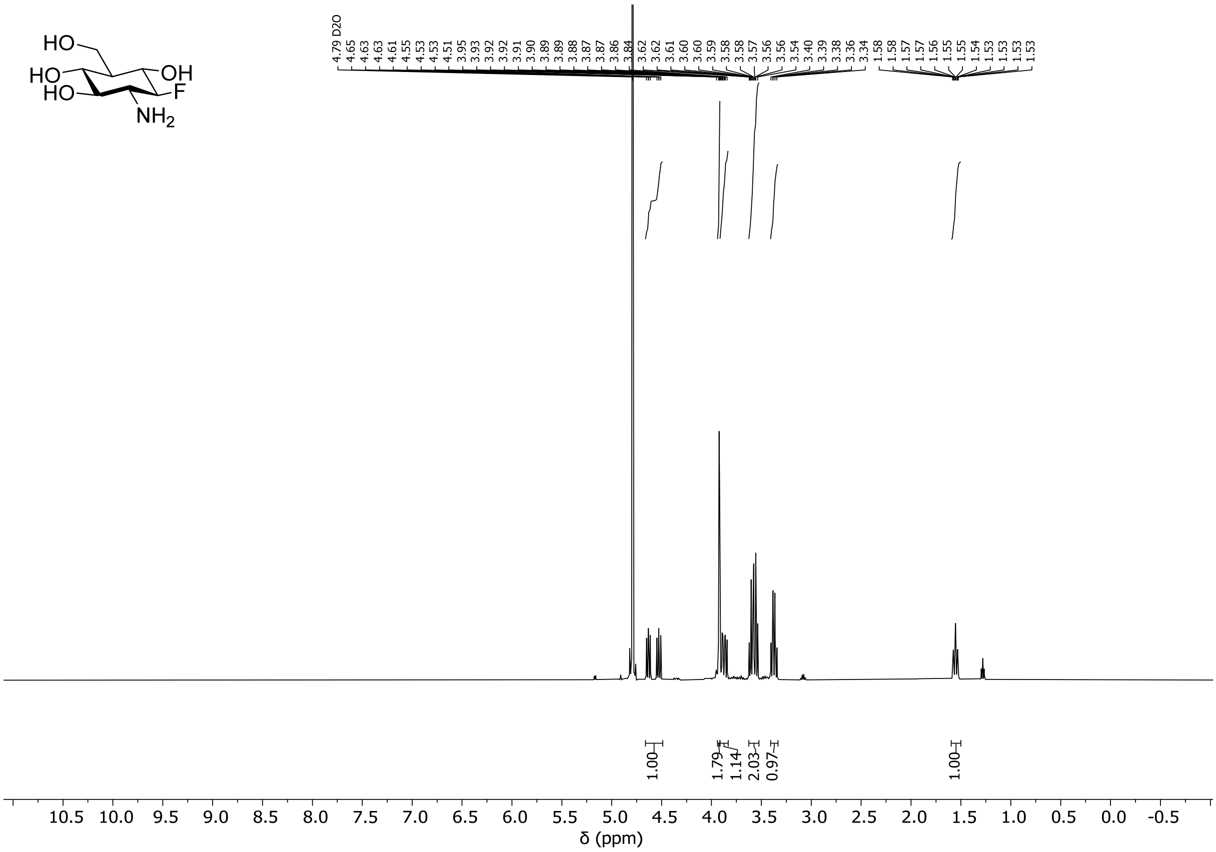

^{1}H NMR spectrum (500 MHz, D_2O) of **41**

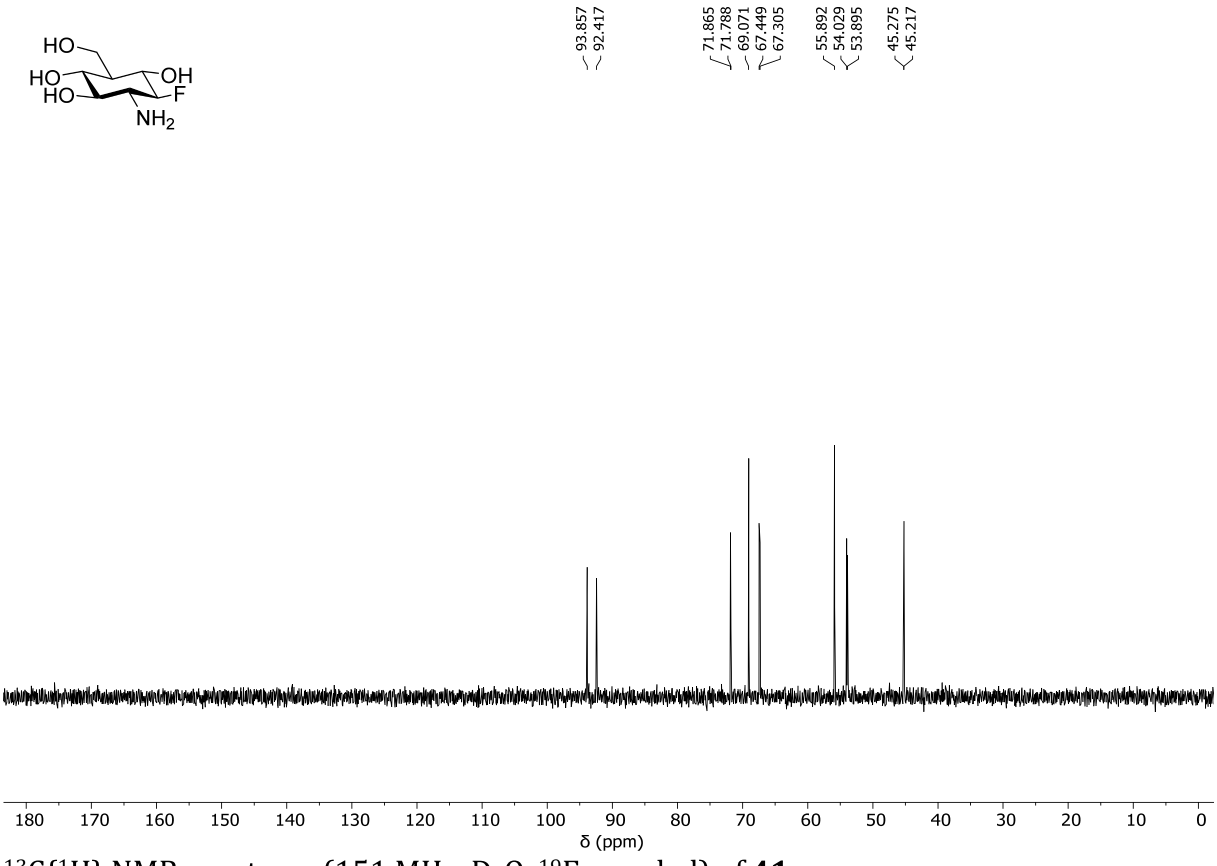

$^{13}C\{^{1}H\}$ NMR spectrum (151 MHz, D_2O, ^{19}F-coupled) of **41**

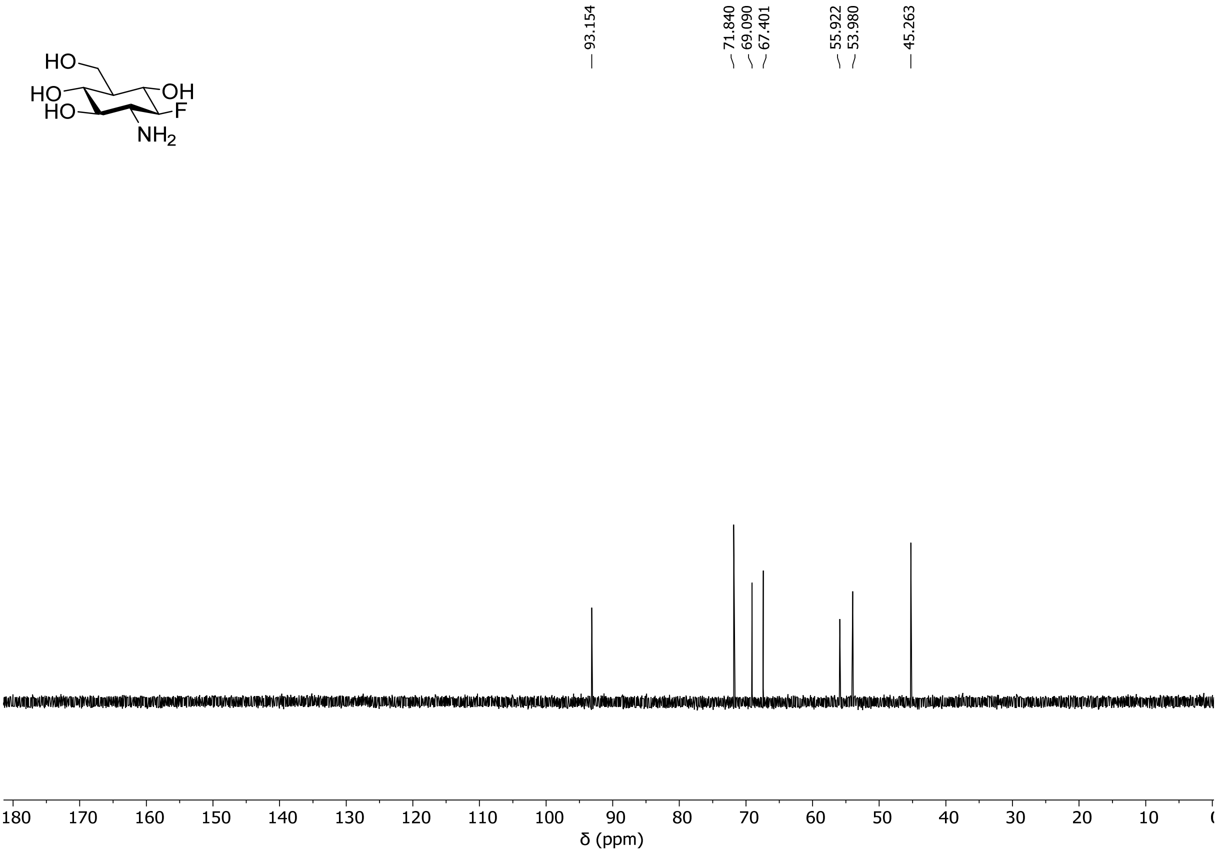

$^{13}C\{^{1}H\}$ NMR spectrum (126 MHz, D_2O, ^{19}F-decoupled) of **41**

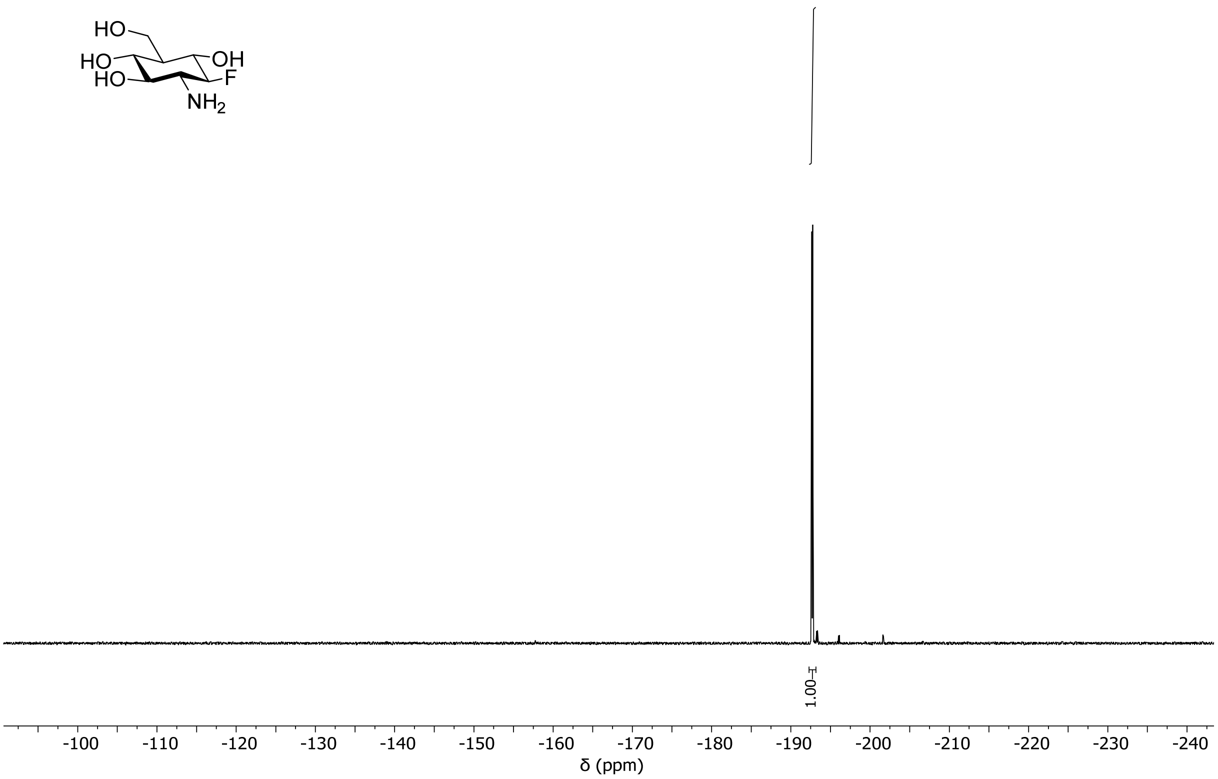

^{19}F NMR spectrum (471 MHz, D_2O) of **41**

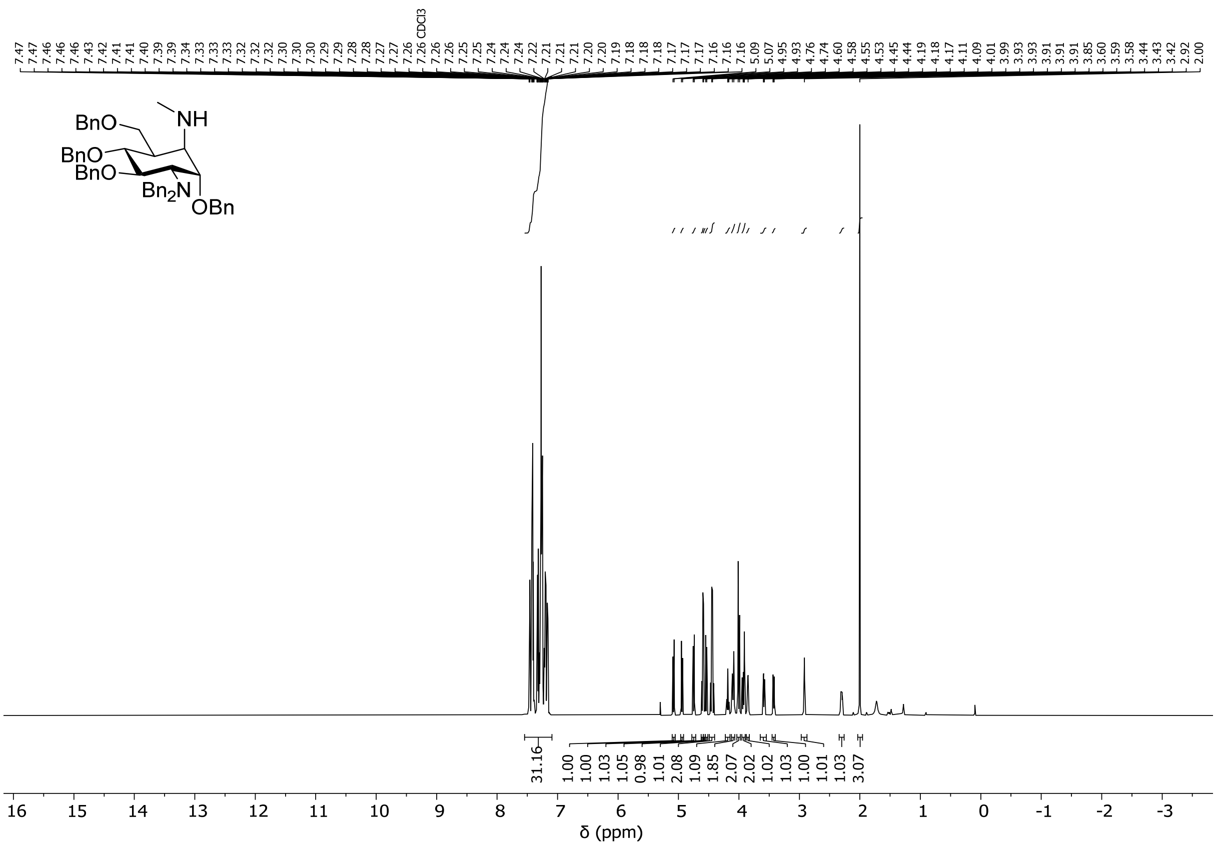

^{1}H NMR spectrum (600 MHz, $CDCl_3$) of **42**

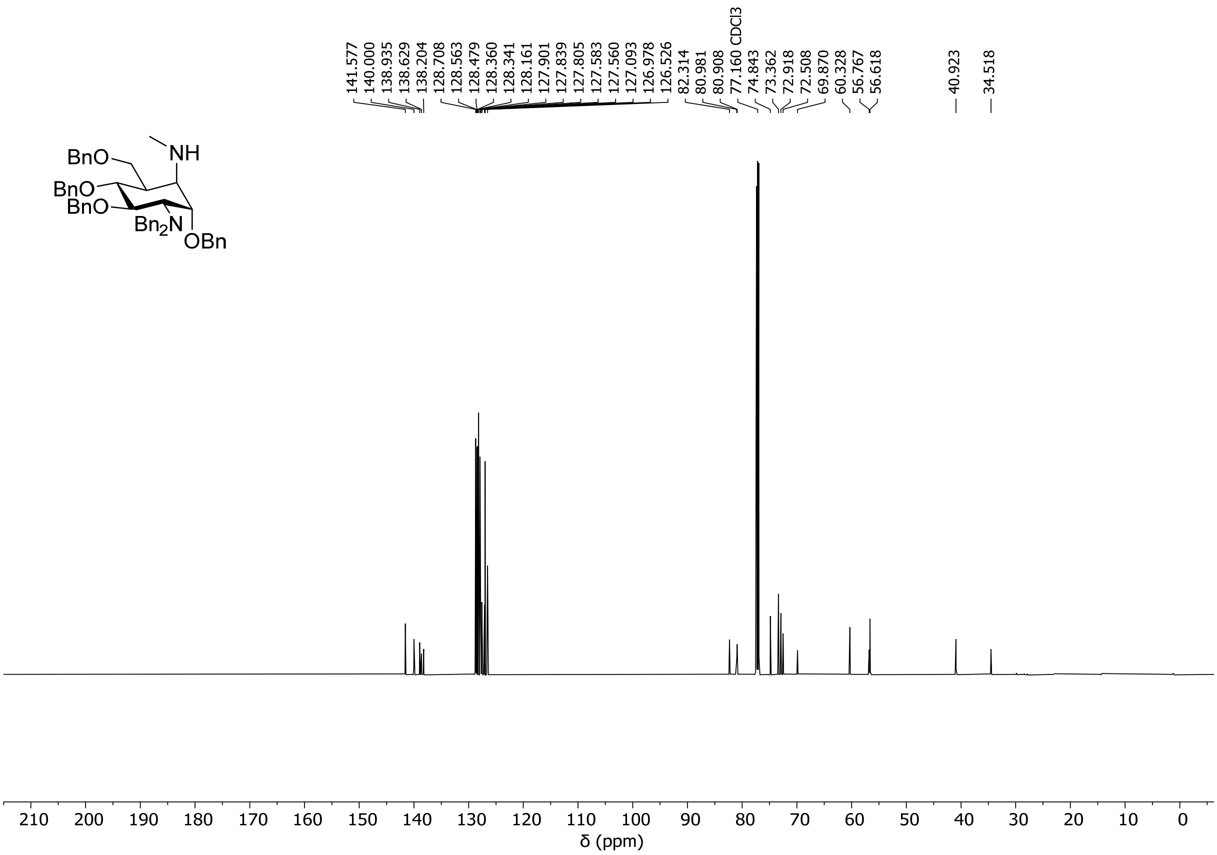

$^{13}C\{^1H\}$ NMR spectrum (151 MHz, $CDCl_3$) of **42**

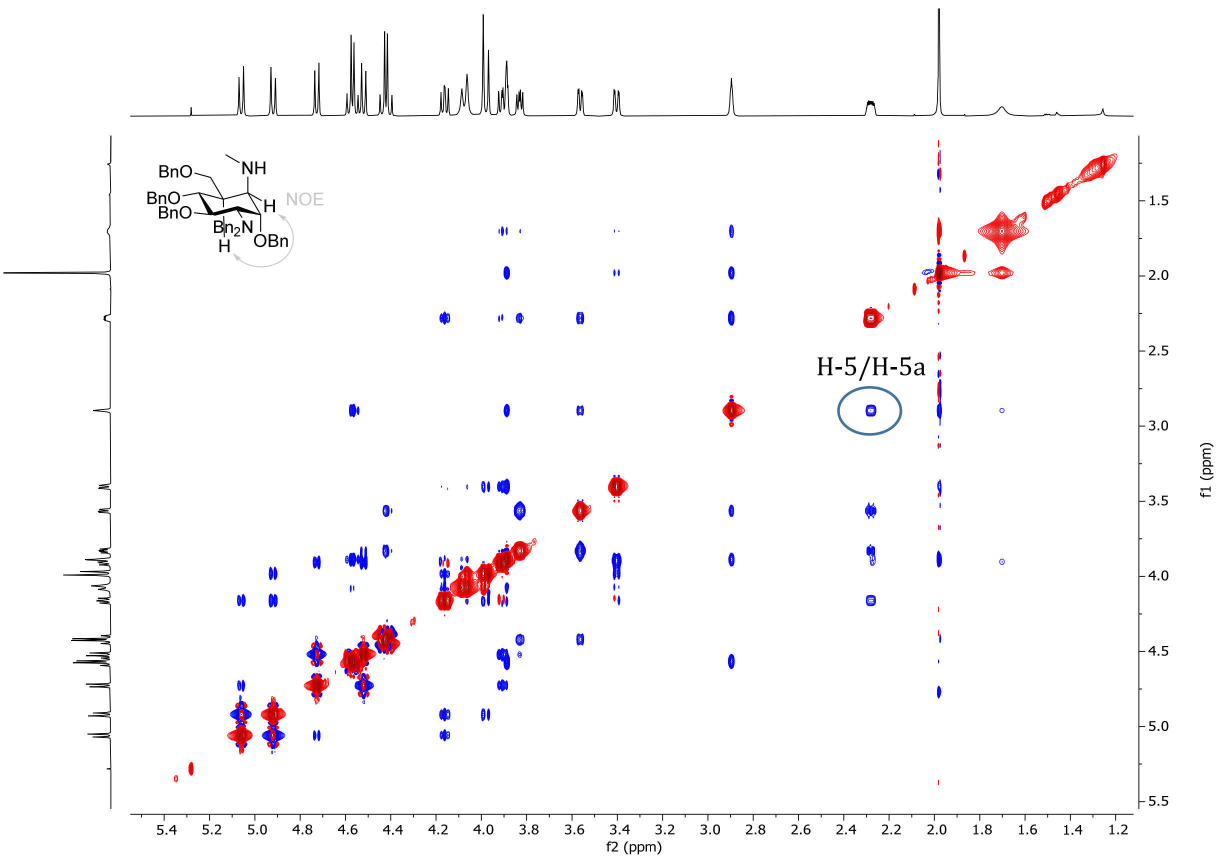

NOESY (600 MHz, $CDCl_3$) spectrum of **42**. In addition to the observed cross peak between H-5 and H-5a, the coupling constants of H-5a provide further evidence for the stereochemistry. H-5a is observed as a pseudo triplet with *J* = 3.2 Hz, which indicates the absence of an axial-axial coupling and, therefore, implies its equatorial orientation.

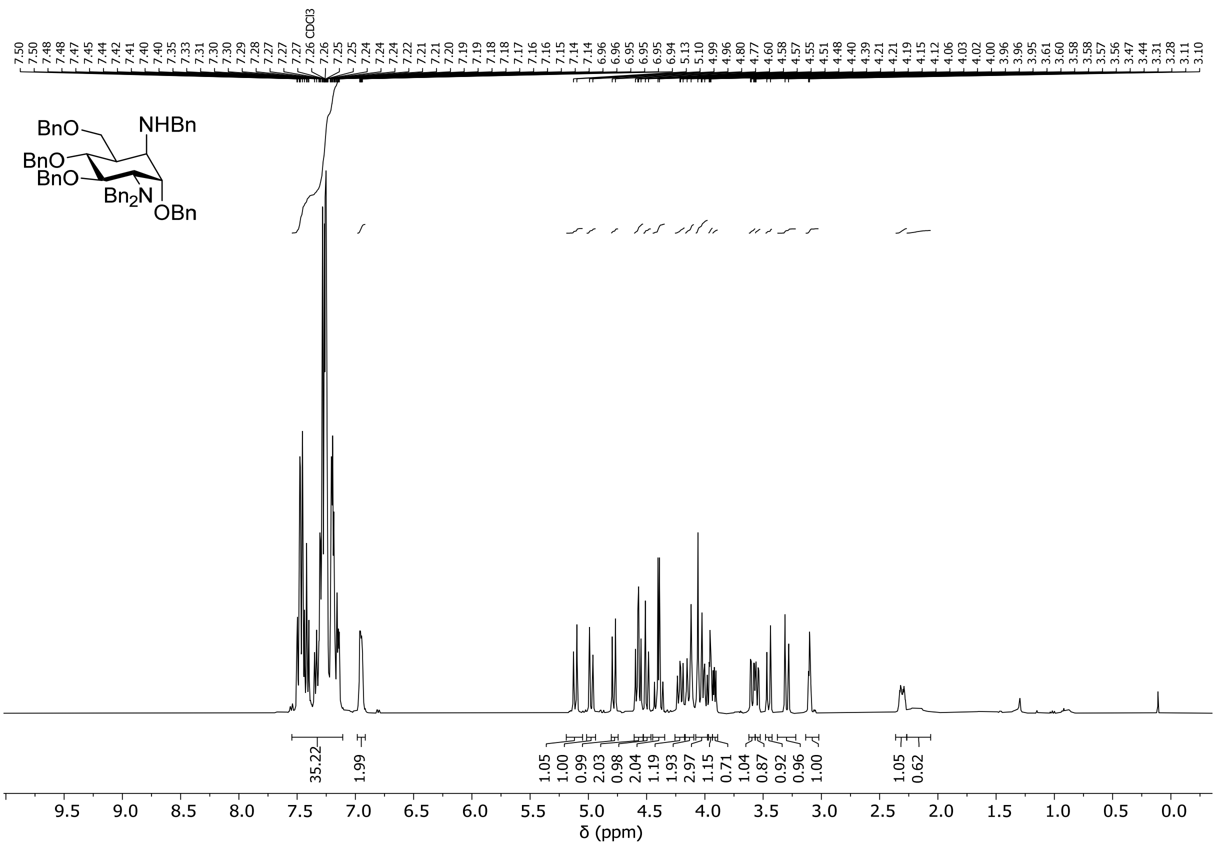

^1H NMR spectrum (400 MHz, $CDCl_3$) of **43**

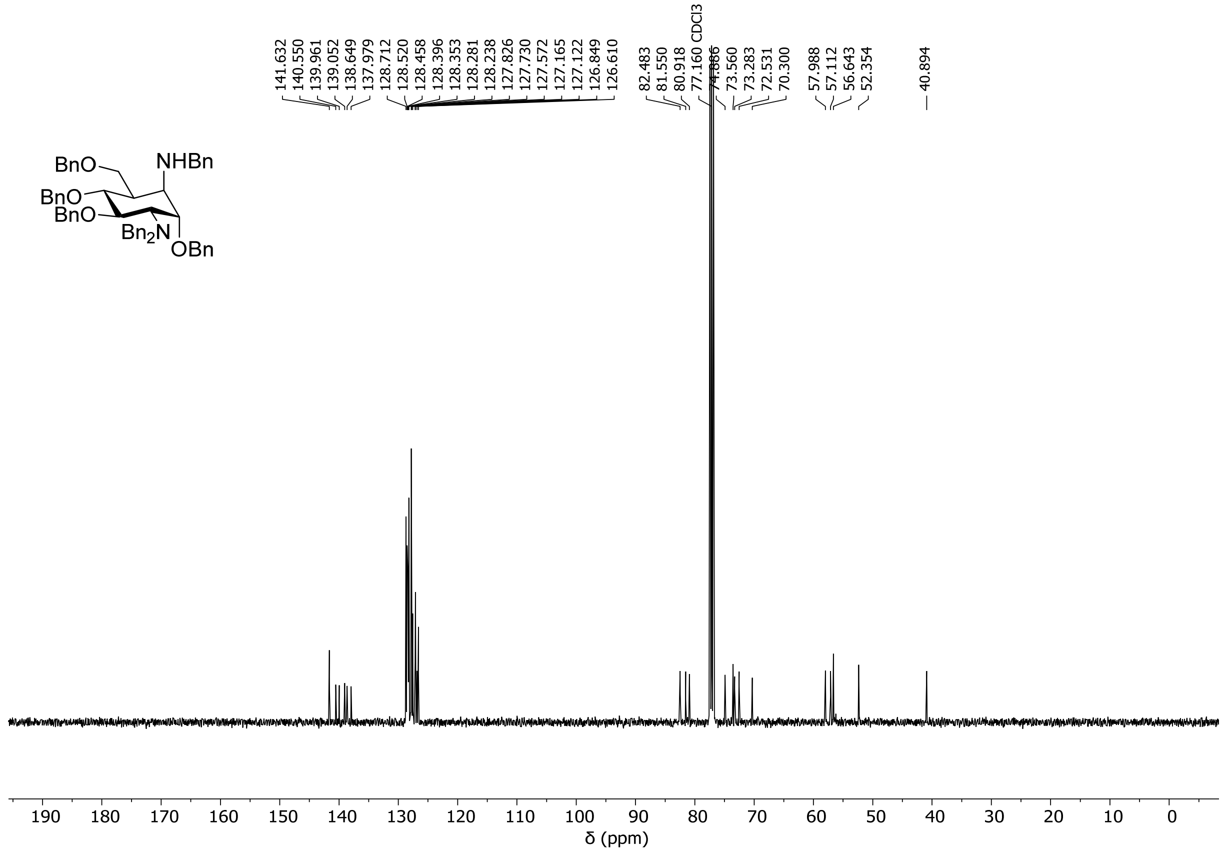

$^{13}C\{^1H\}$ NMR spectrum (101 MHz, $CDCl_3$) of **43**

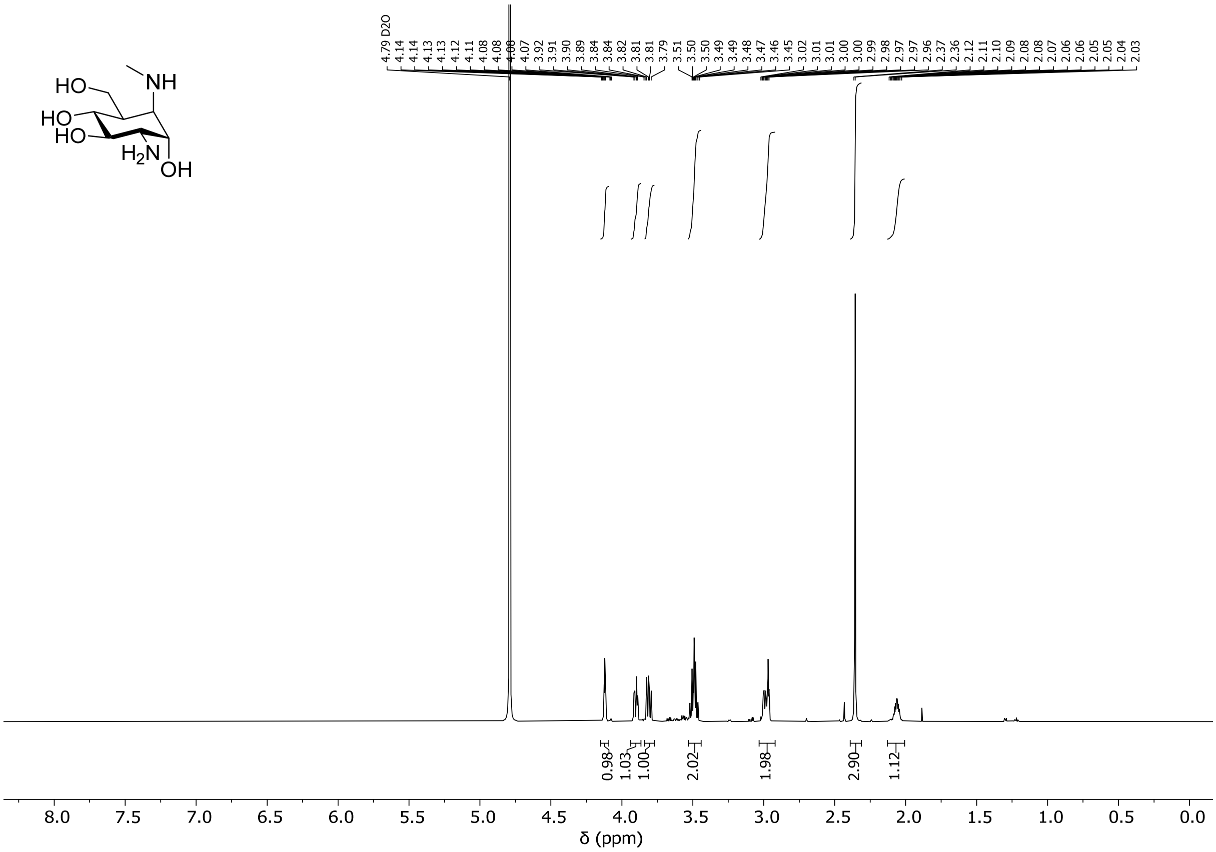

^{1}H NMR spectrum (600 MHz, D_2O) of **44**

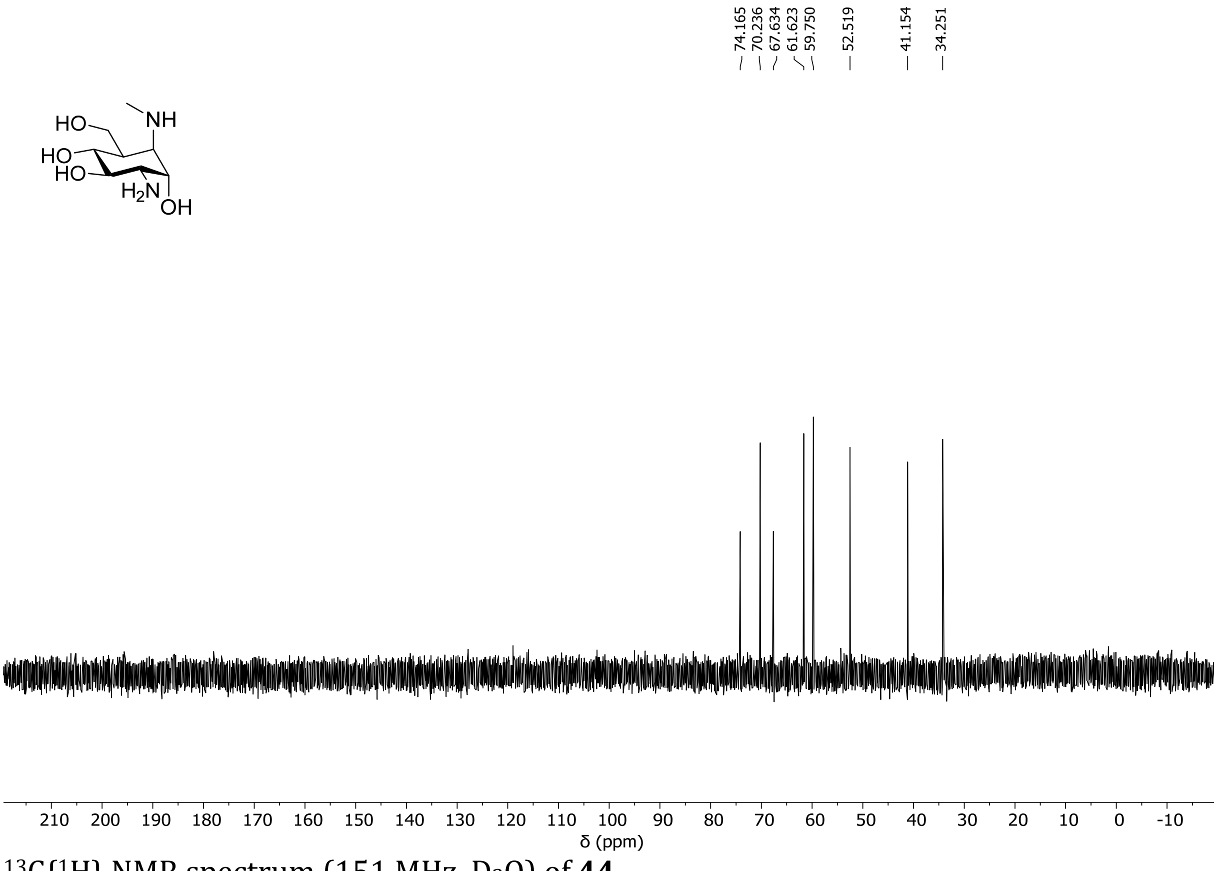

$^{13}C\{^{1}H\}$ NMR spectrum (151 MHz, D_2O) of **44**

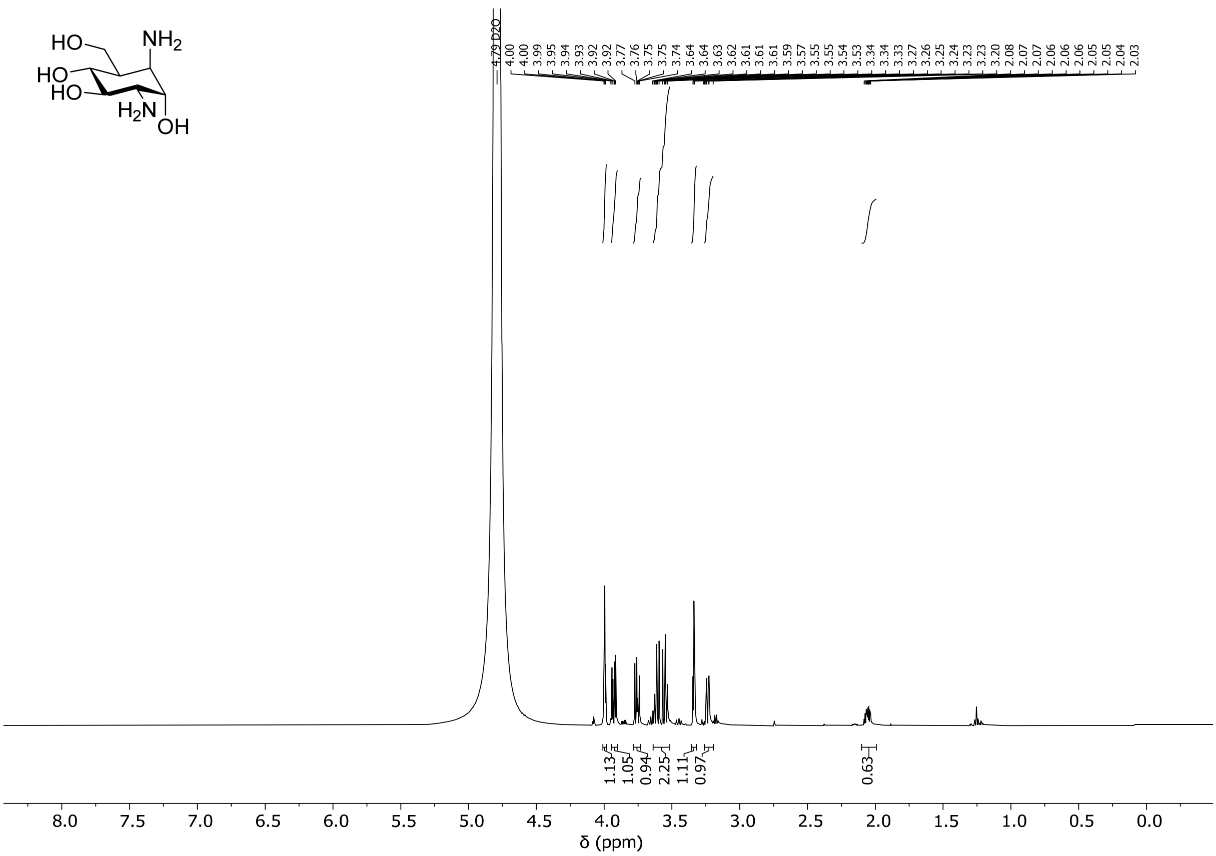

^{1}H NMR spectrum (600 MHz, D_2O) of **45**

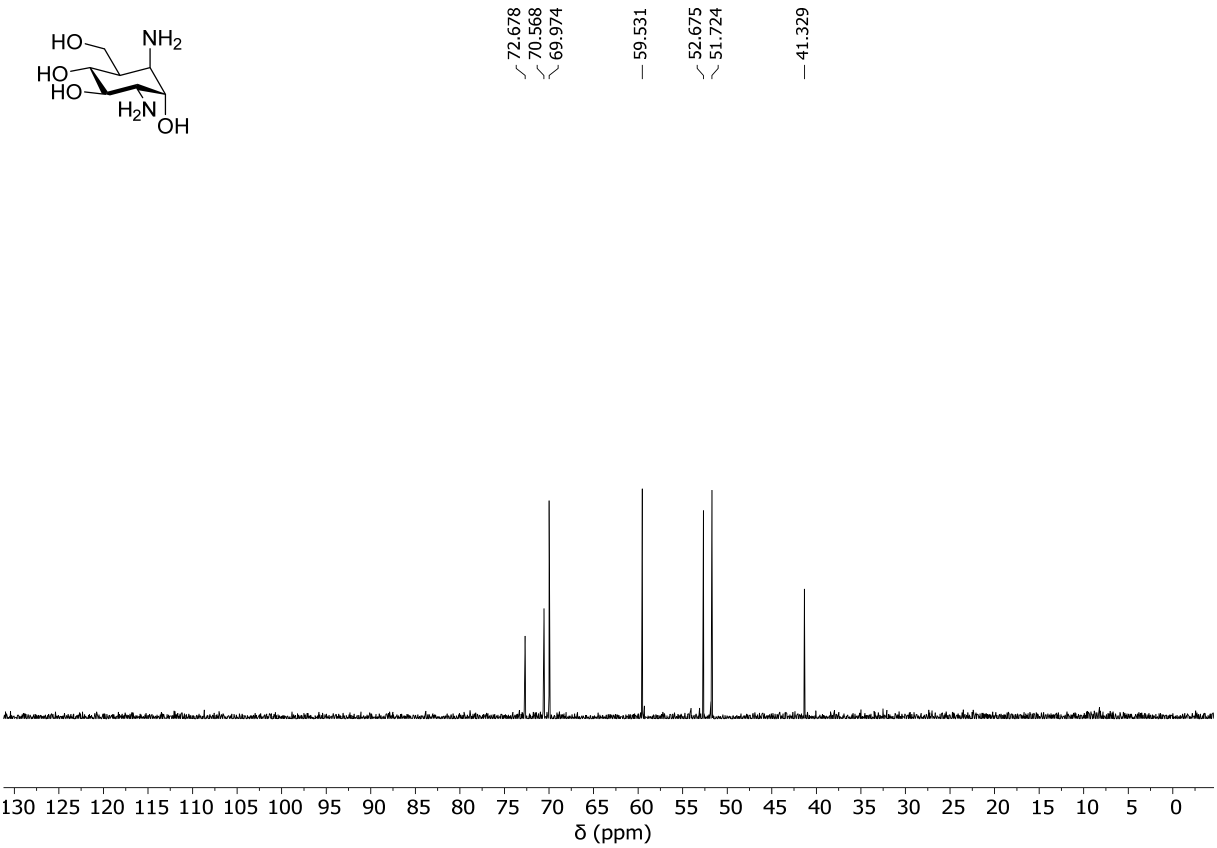

^{13}C{^{1}H} NMR spectrum (151 MHz, D_2O) of **45**

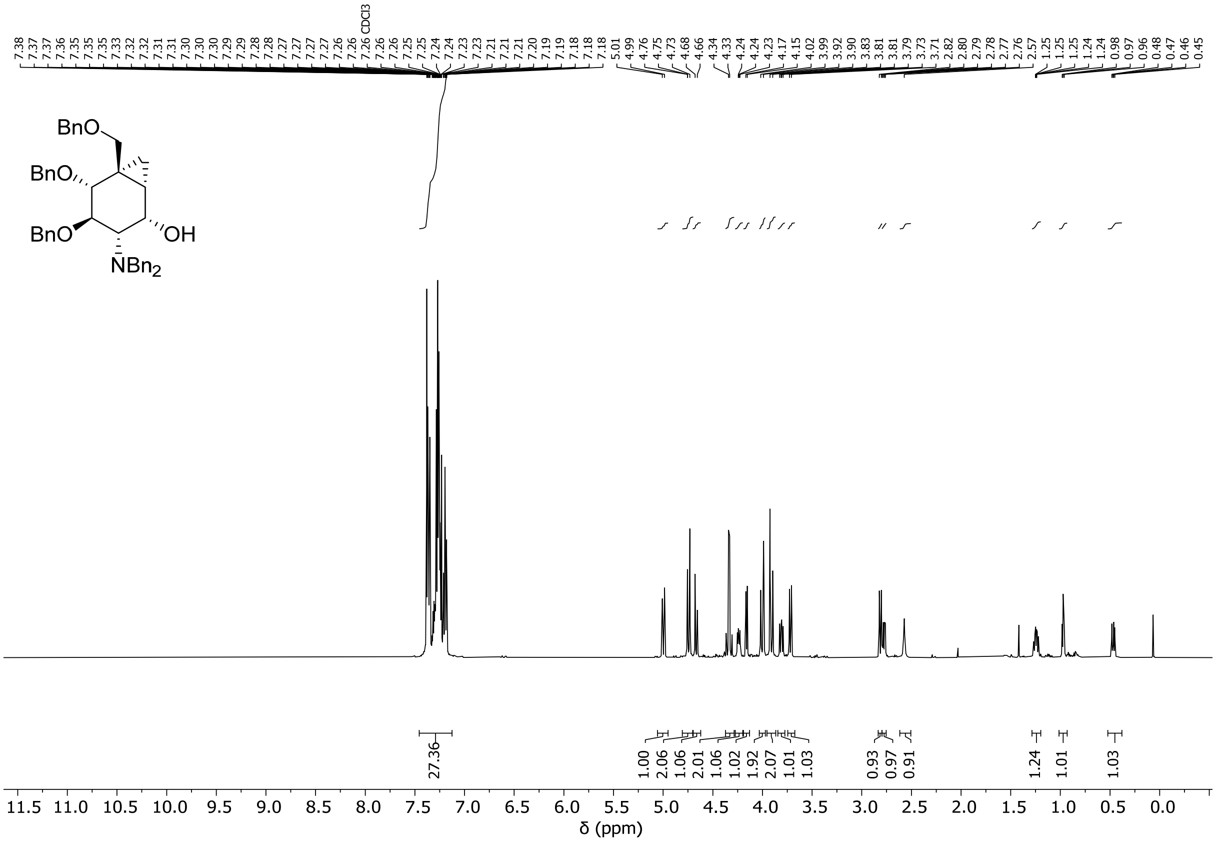

^{1}H NMR spectrum (500 MHz, $CDCl_3$) of **46**

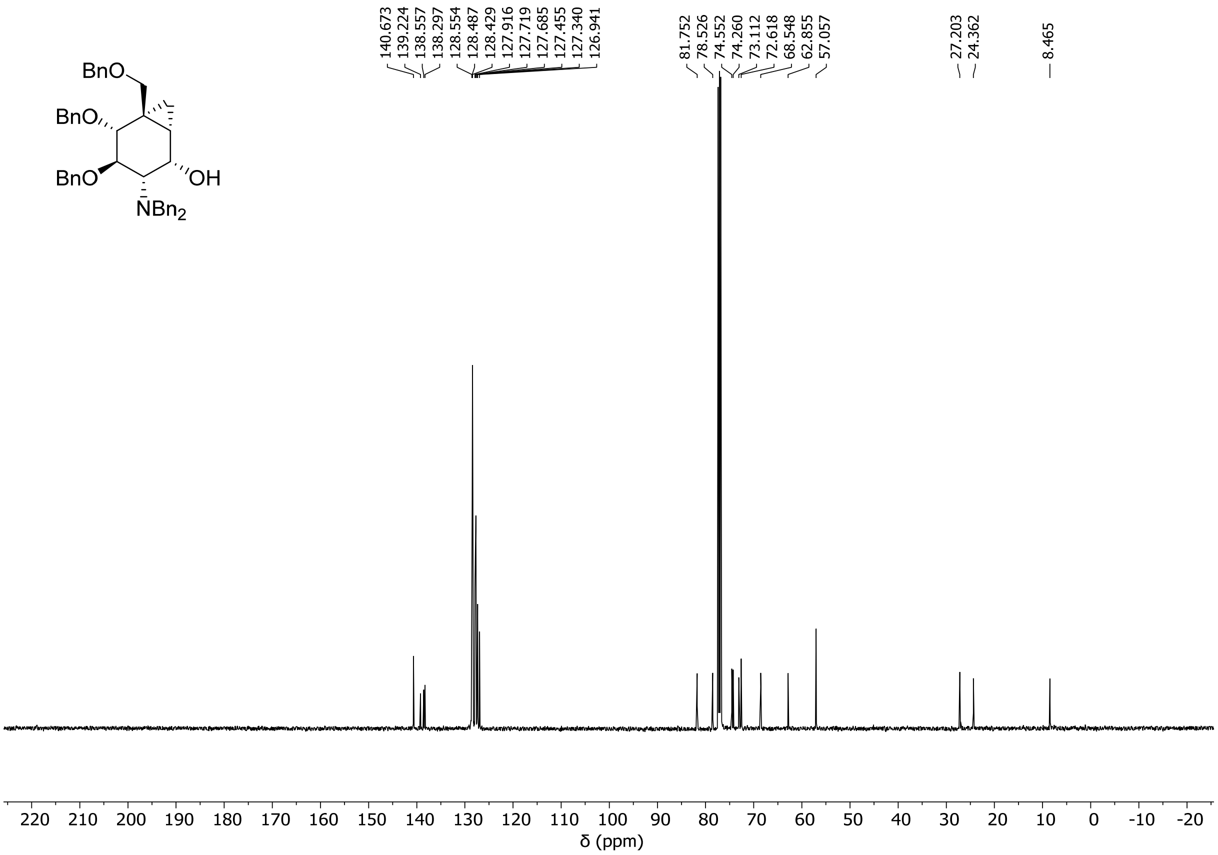

$^{13}C\{^{1}H\}$ NMR spectrum (126 MHz, $CDCl_3$) of **46**

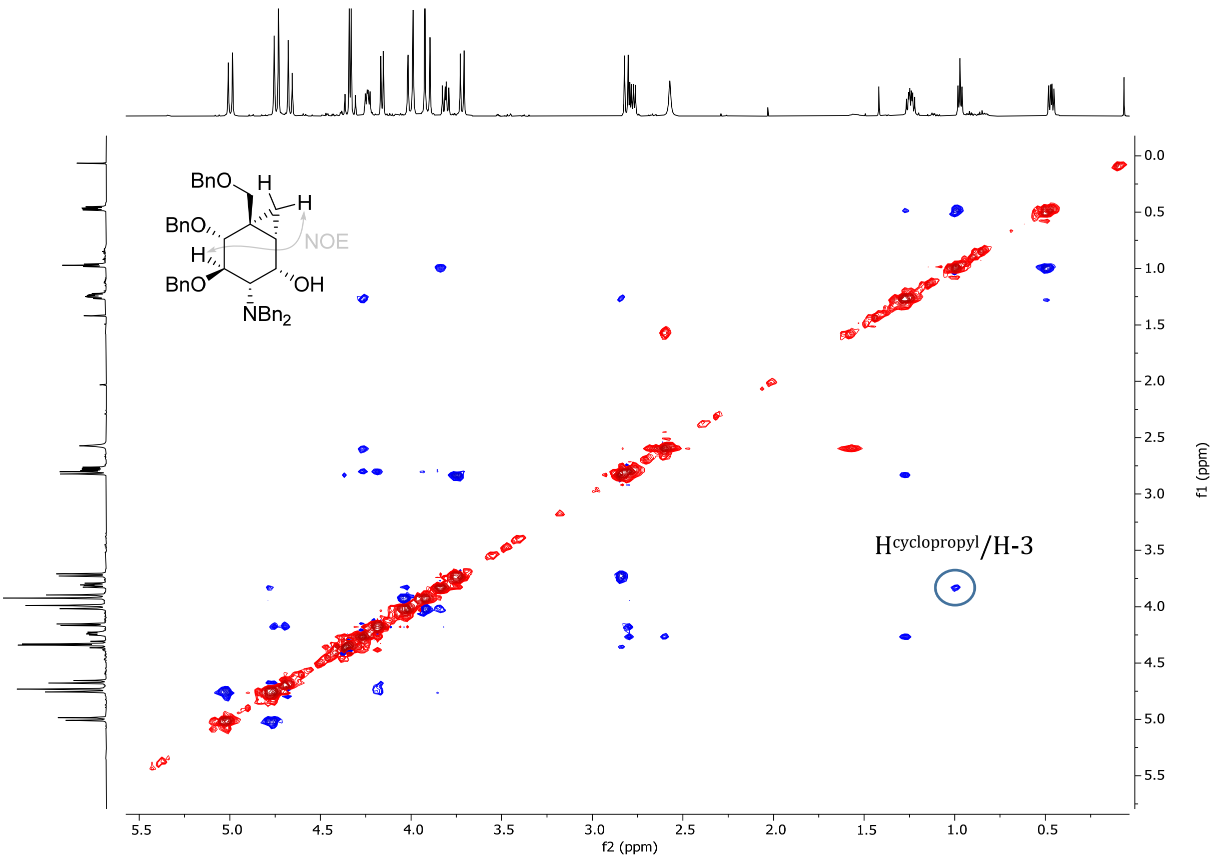

NOESY (500 MHz, $CDCl_3$) spectrum of **46**

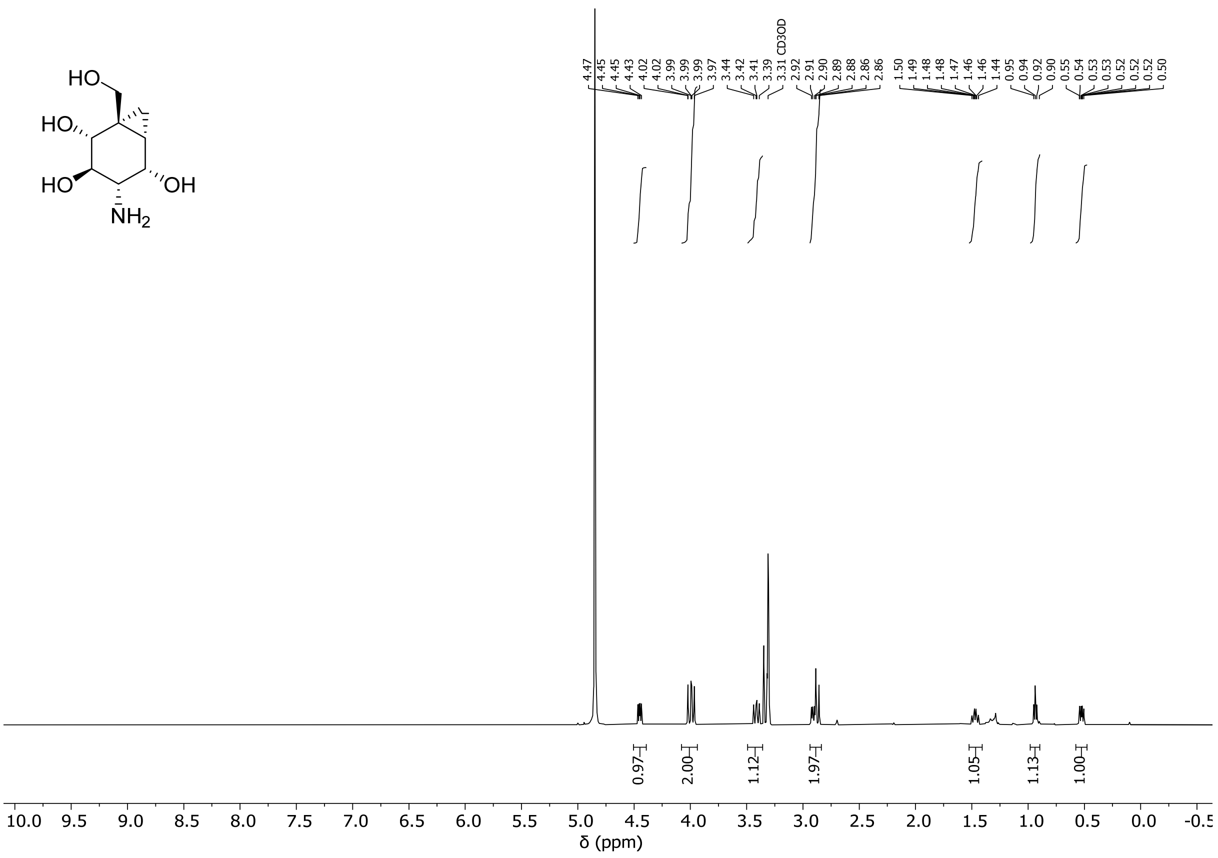

^{1}H NMR spectrum (500 MHz, D_2O) of **47**

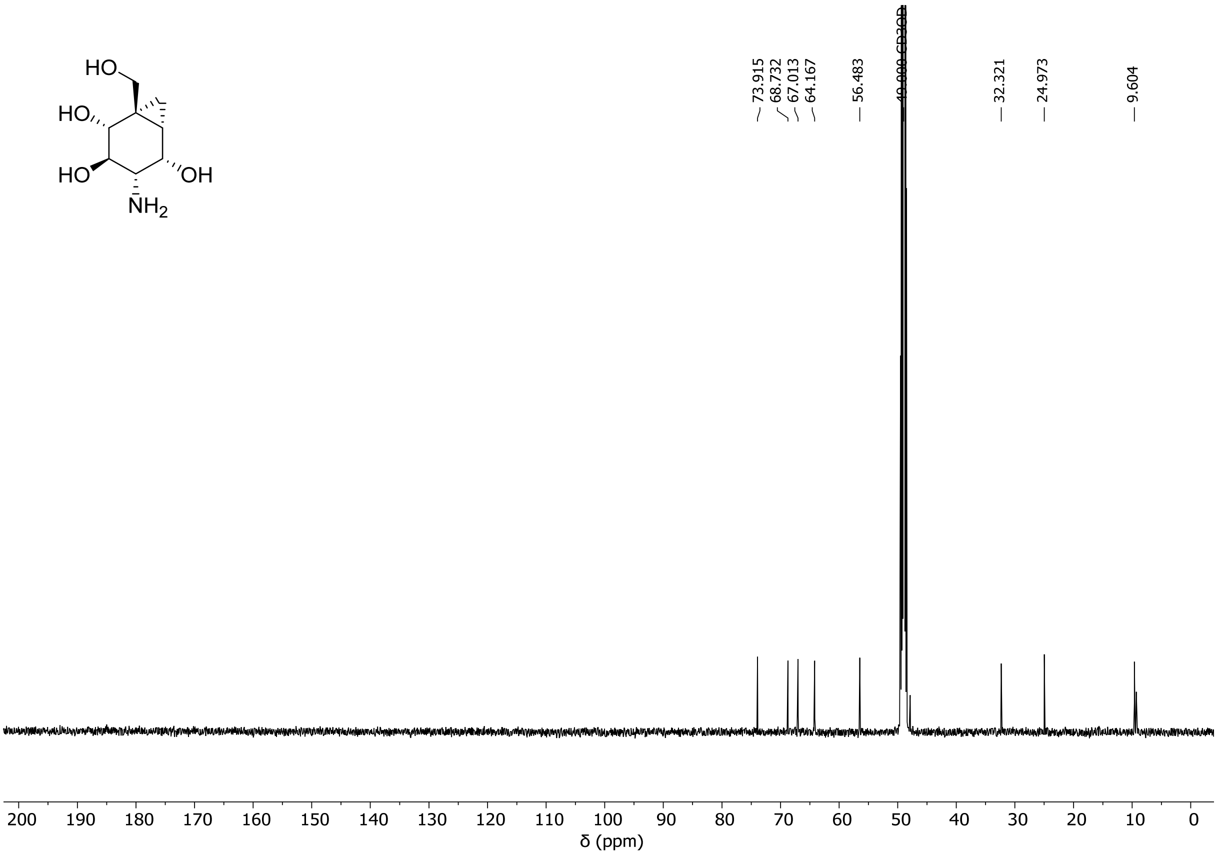

^{13}C{^{1}H}NMR spectrum (126 MHz, CD_3OD) of **47**

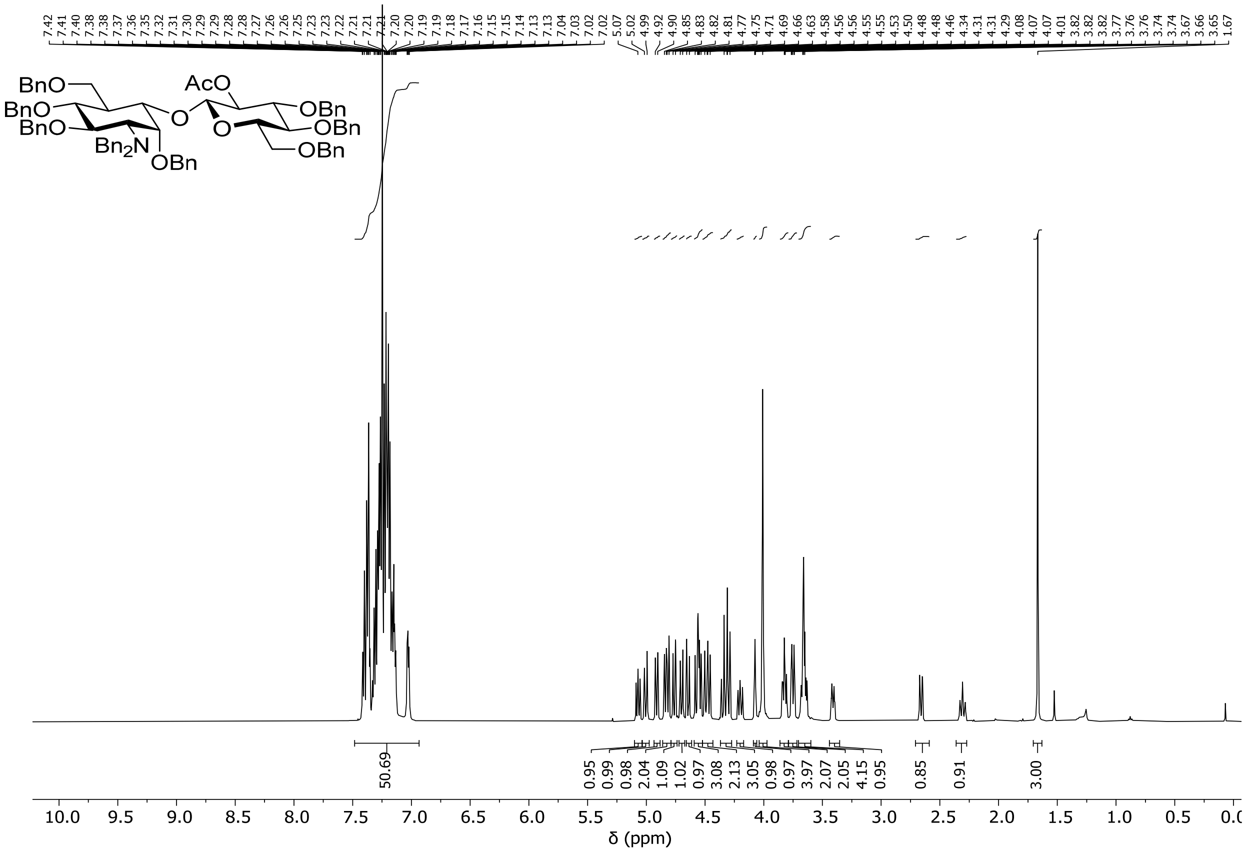

^{1}H-NMR (500 MHz, CDCl$_3$) spectrum of **49**

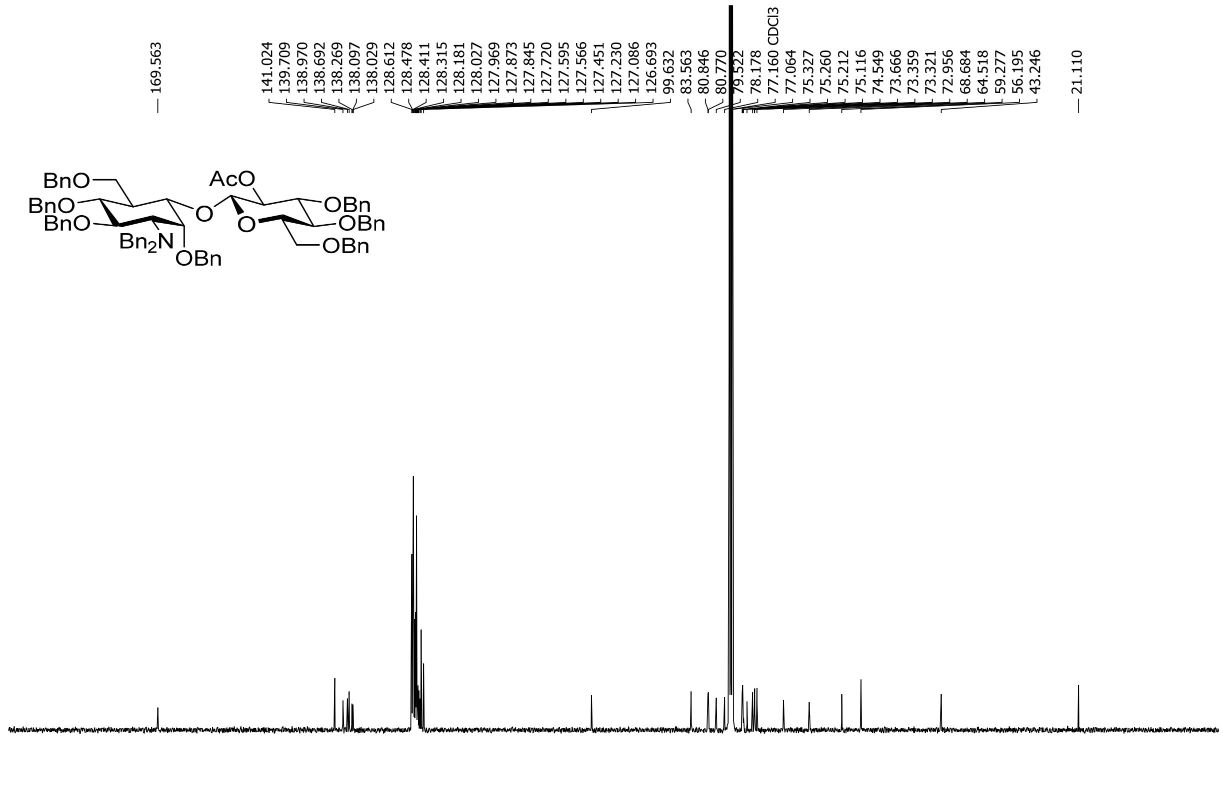

^{13}C{^{1}H}-NMR (121 MHz, CDCl$_3$) spectrum of **49**

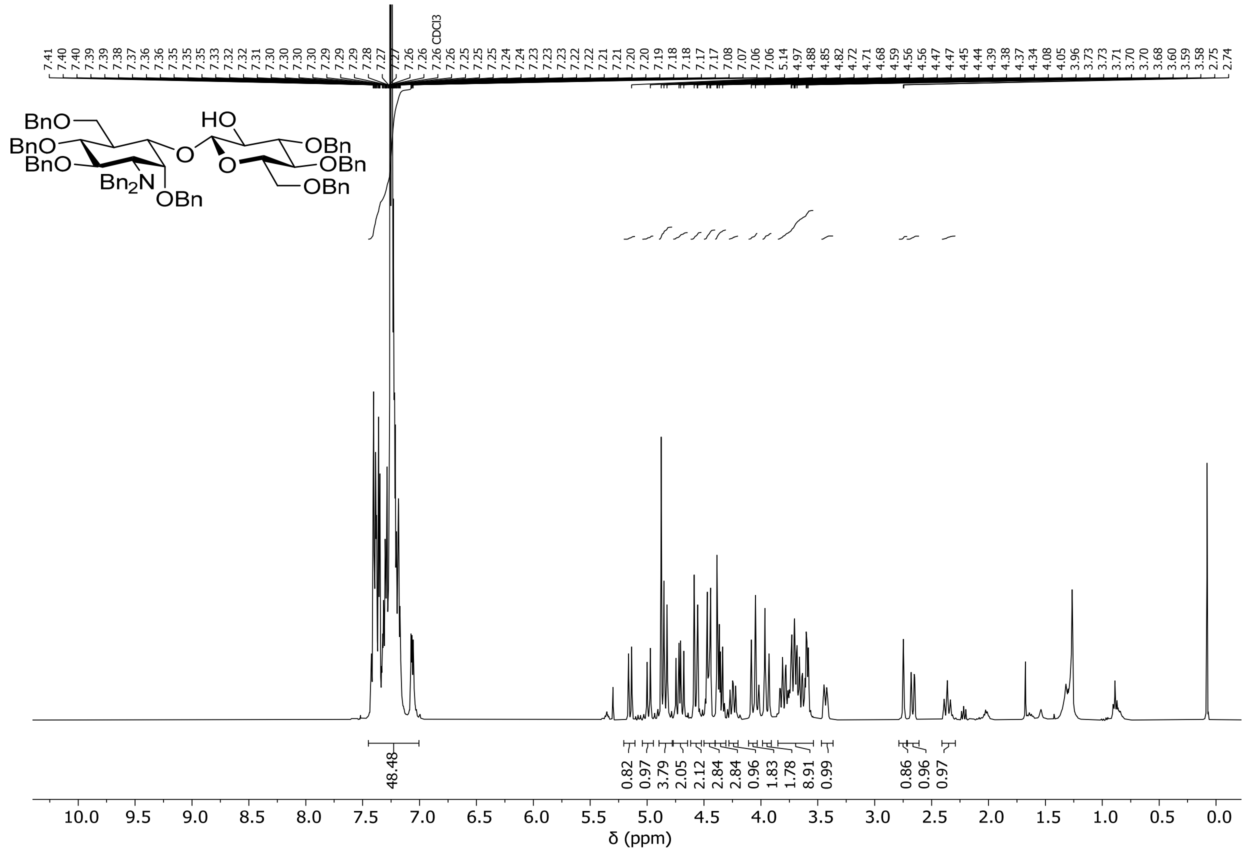

^{1}H-NMR (400 MHz, $CDCl_3$) spectrum of **50**

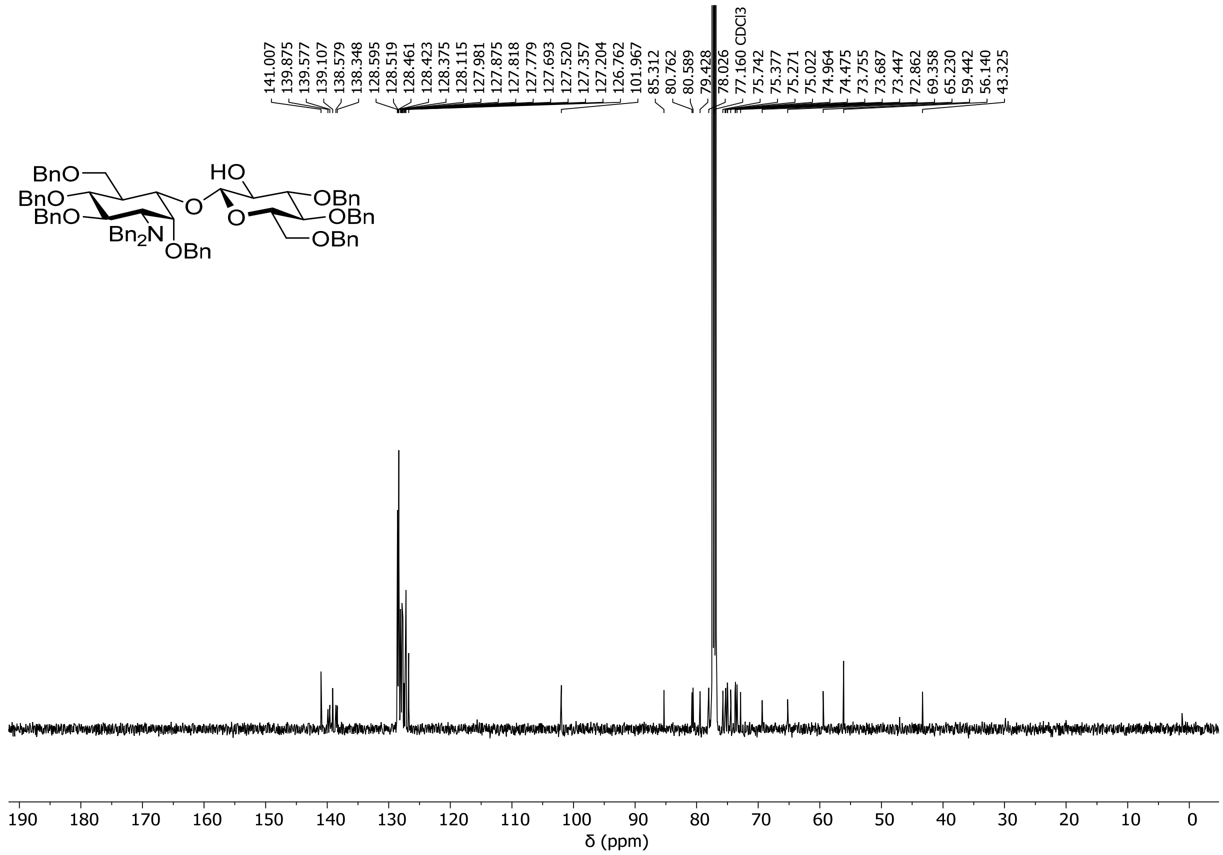

$^{13}C\{^{1}H\}$-NMR (121 MHz, $CDCl_3$) spectrum of **50**

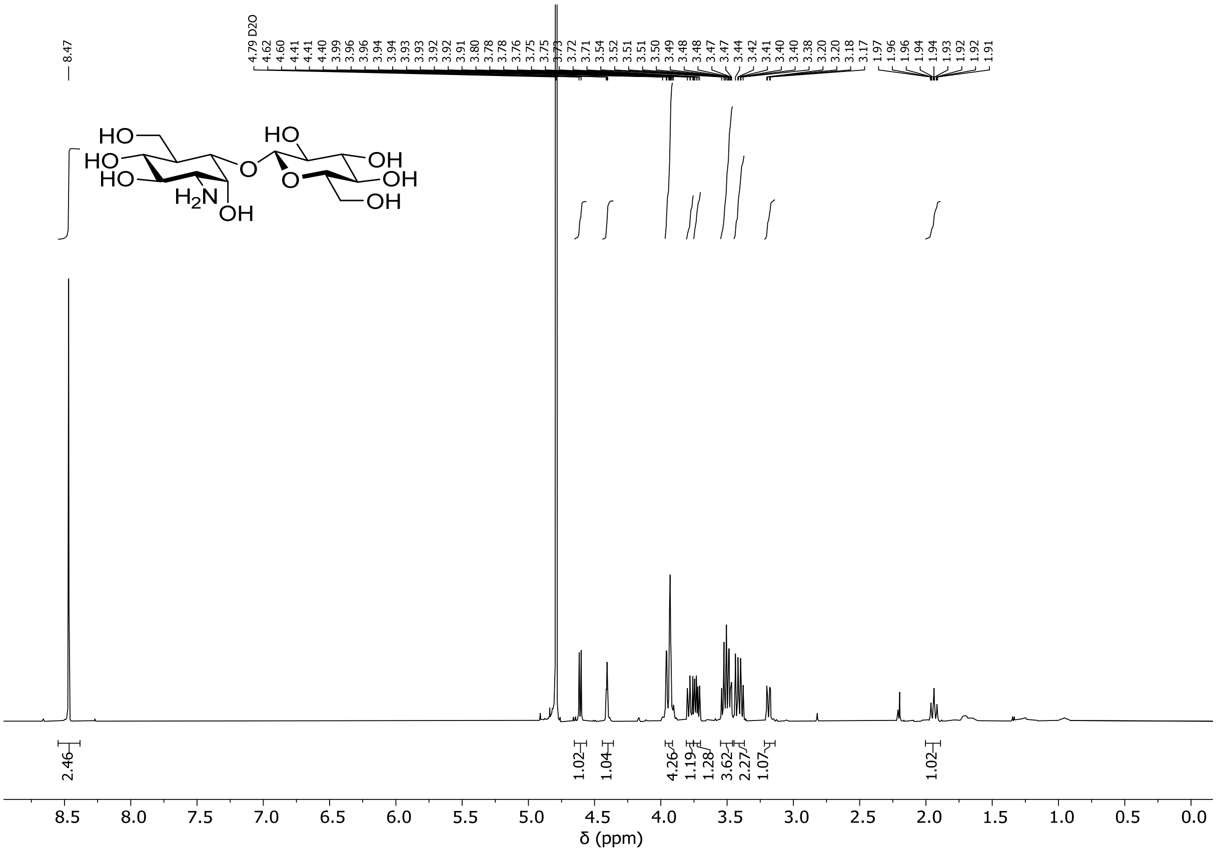

^{1}H-NMR (500 MHz, D_2O) spectrum of **51**

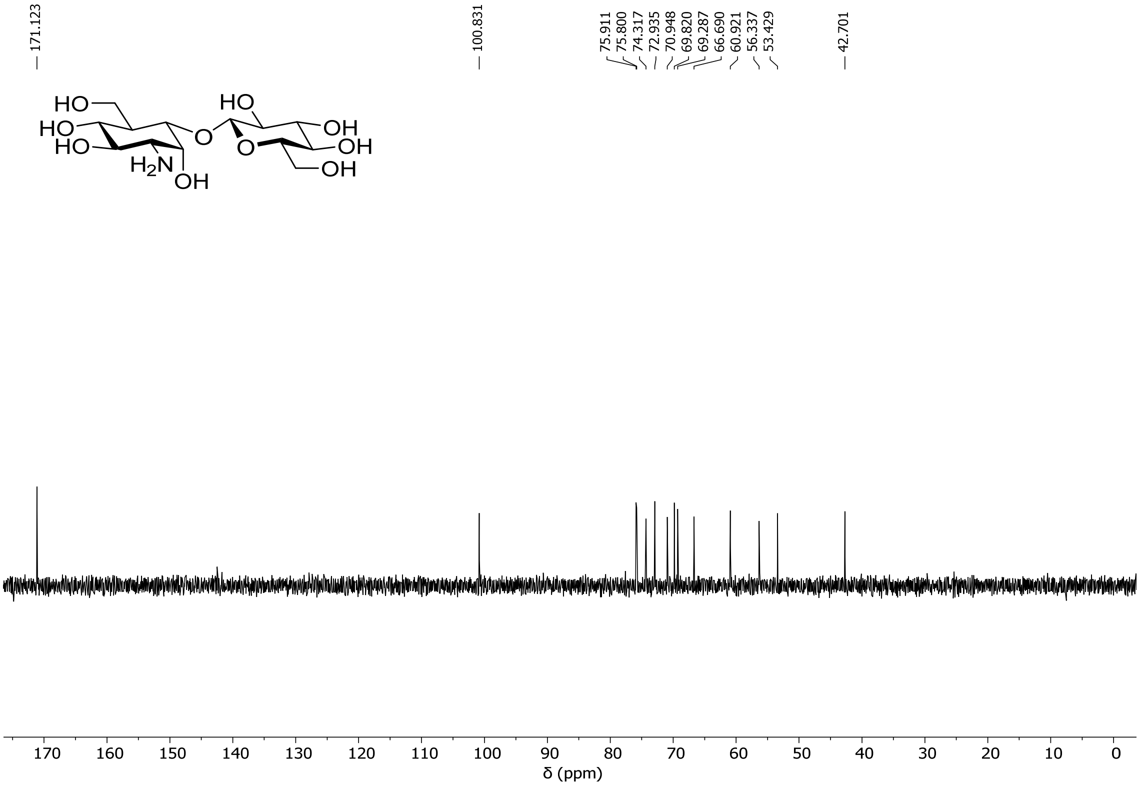

^{13}C{^{1}H}-NMR (121 MHz, D_2O) spectrum of **51**

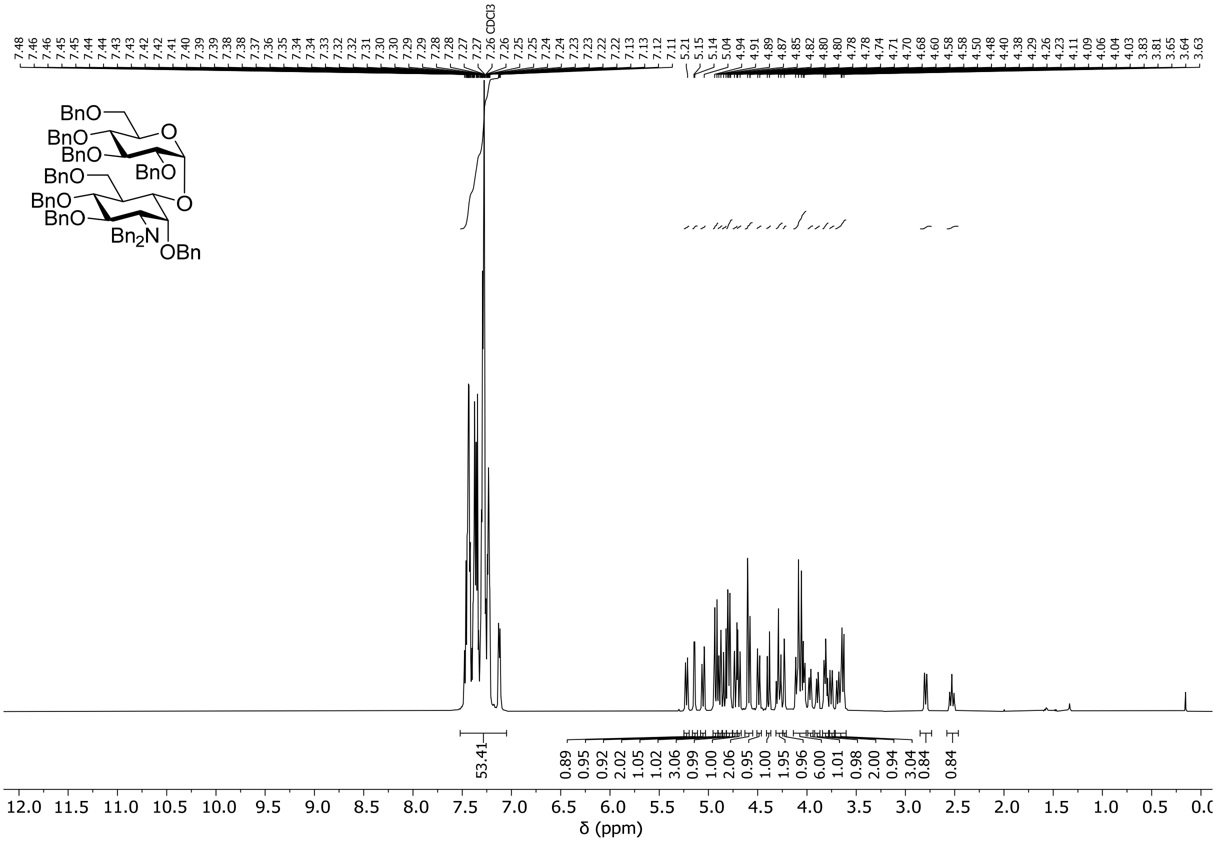

^{1}H-NMR (500 MHz, $CDCl_3$) spectrum of **53**

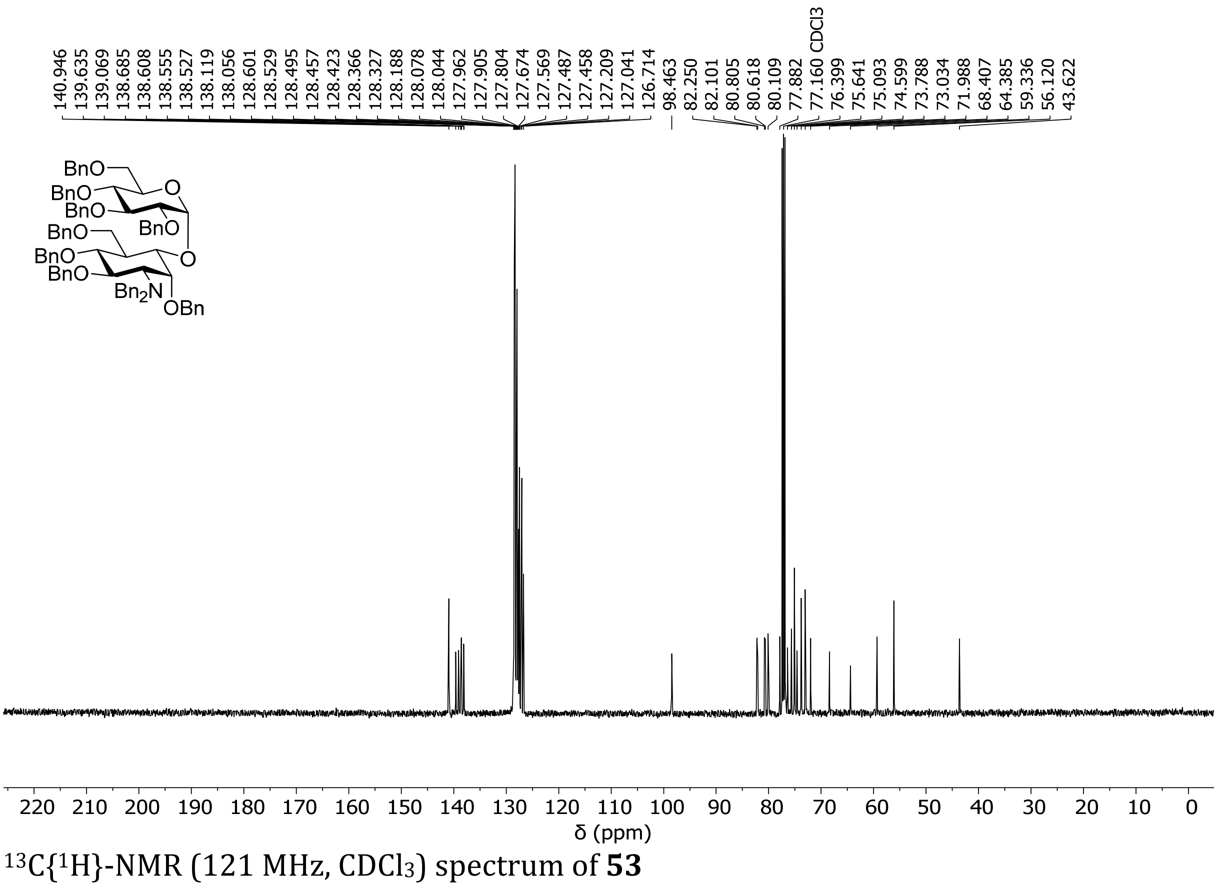

$^{13}C\{^1H\}$-NMR (121 MHz, $CDCl_3$) spectrum of **53**

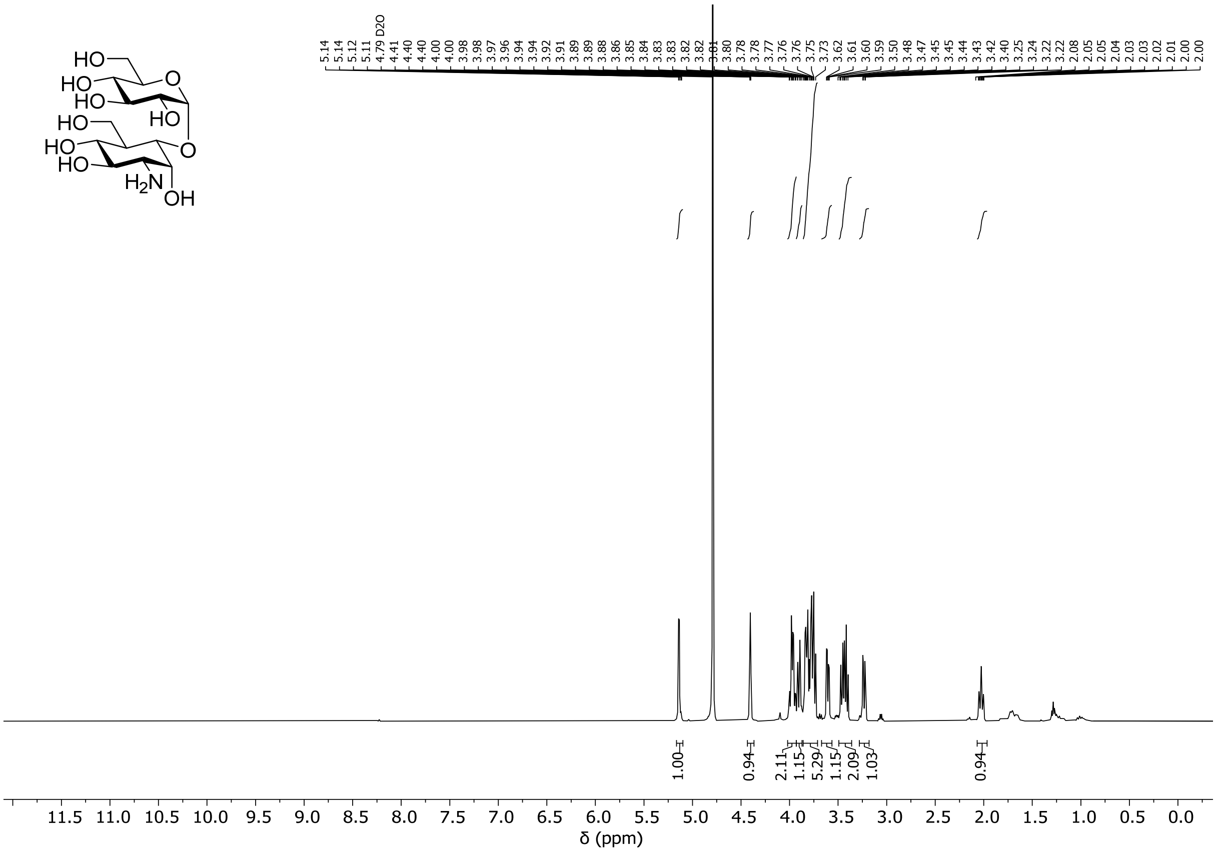

^{1}H-NMR (500 MHz, D_2O) spectrum of **54**

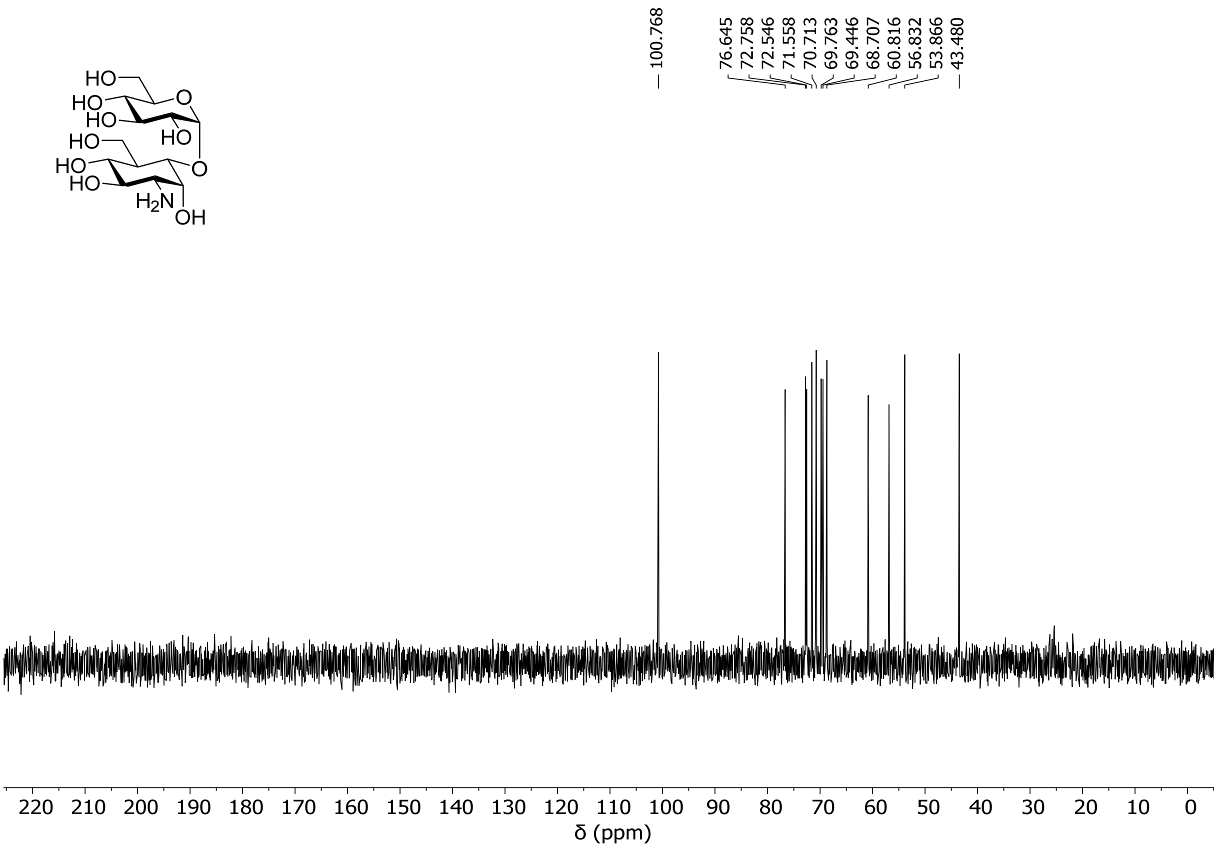

^{13}C{^{1}H}-NMR (121 MHz, D_2O) spectrum of **54**

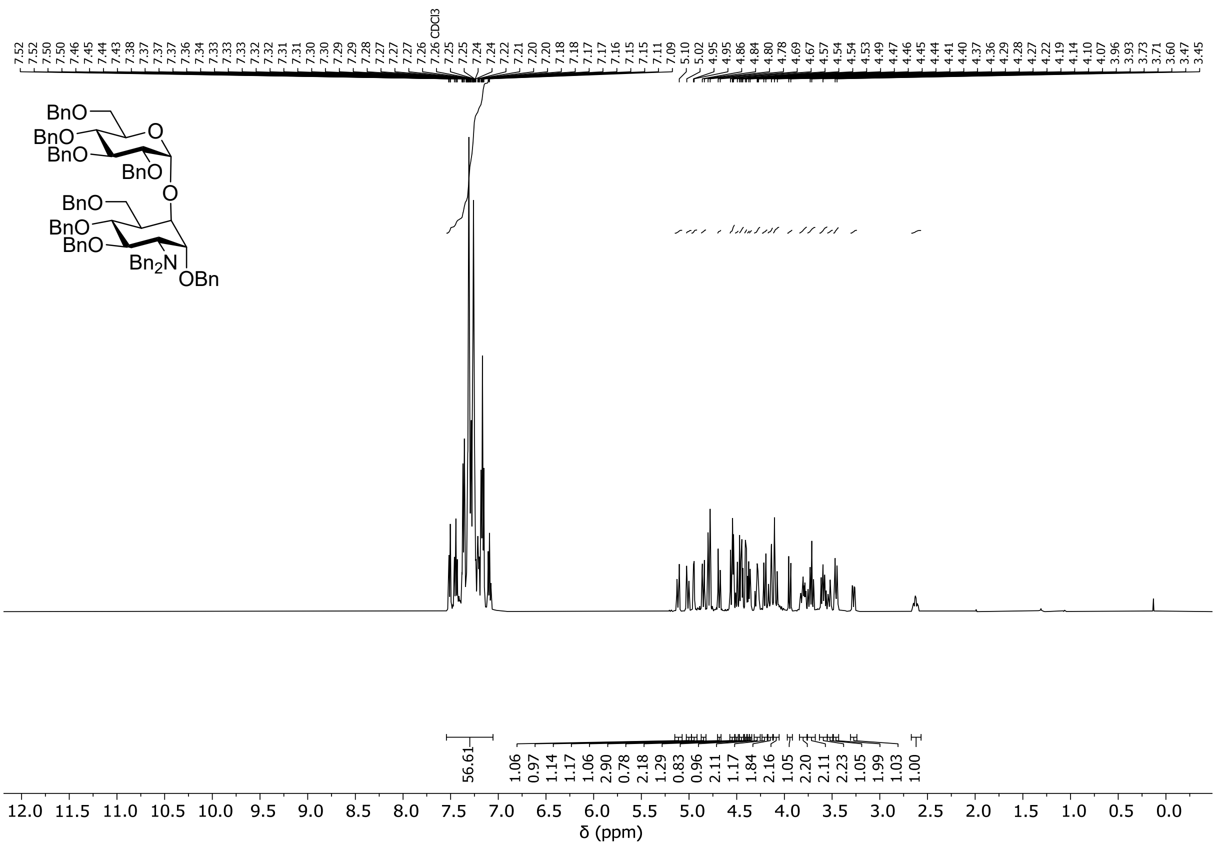

^{1}H-NMR (500 MHz, $CDCl_3$) spectrum of **55**

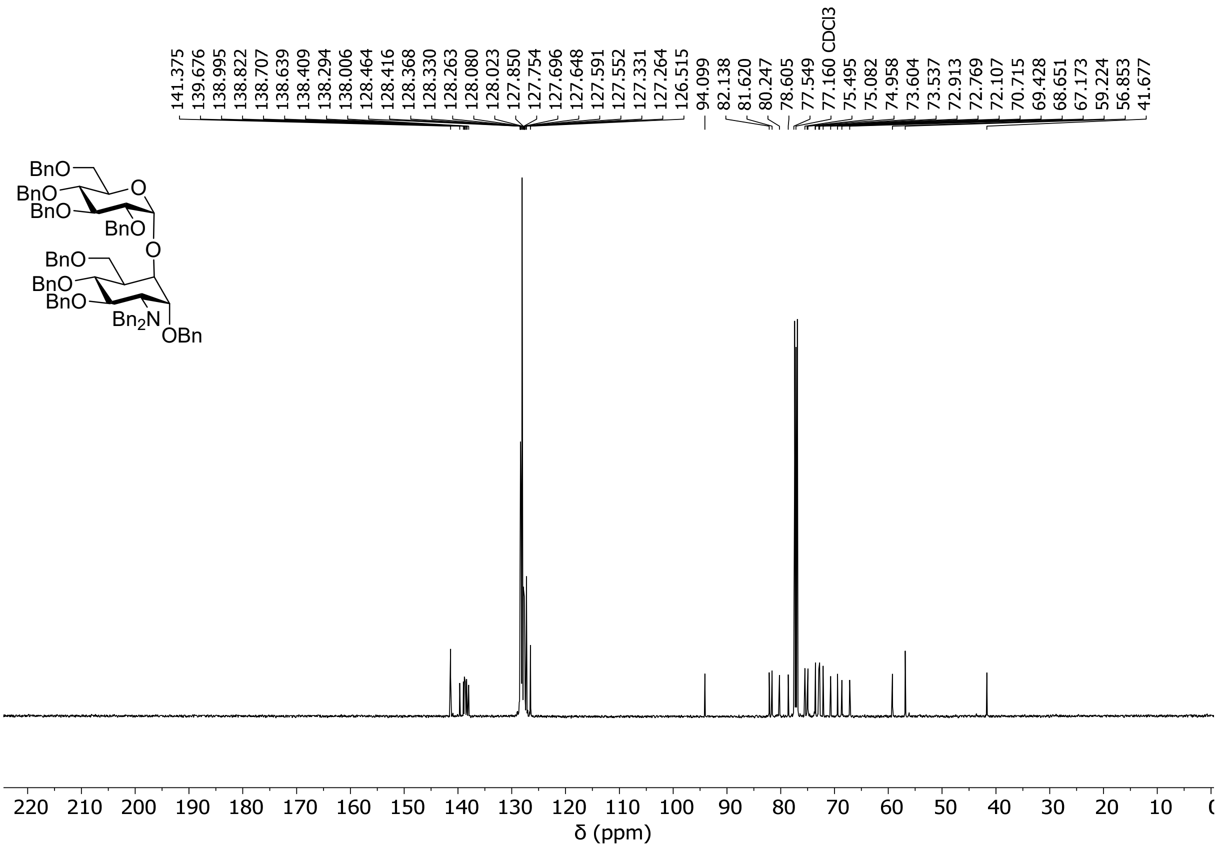

^{13}C{^{1}H}-NMR (121 MHz, $CDCl_3$) spectrum of **55**

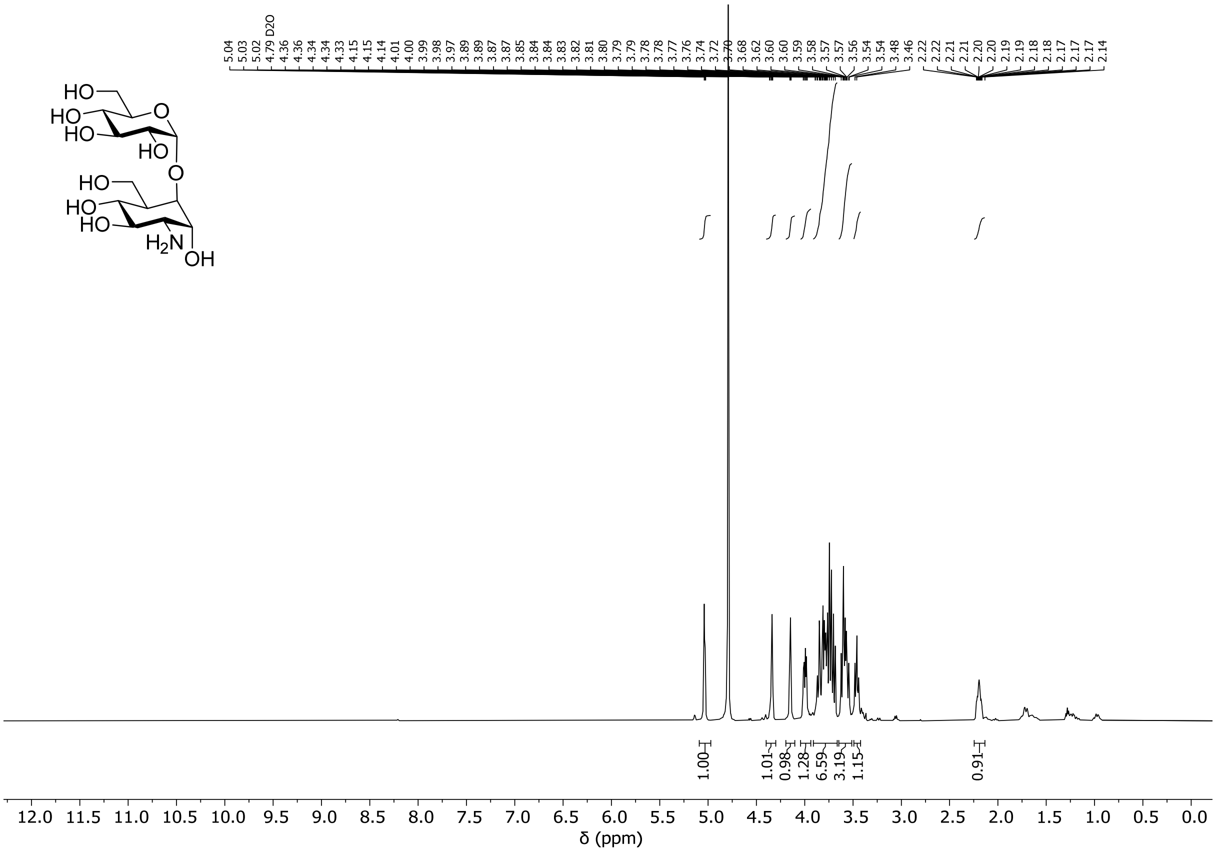

^{1}H-NMR (500 MHz, D_2O) spectrum of **56**

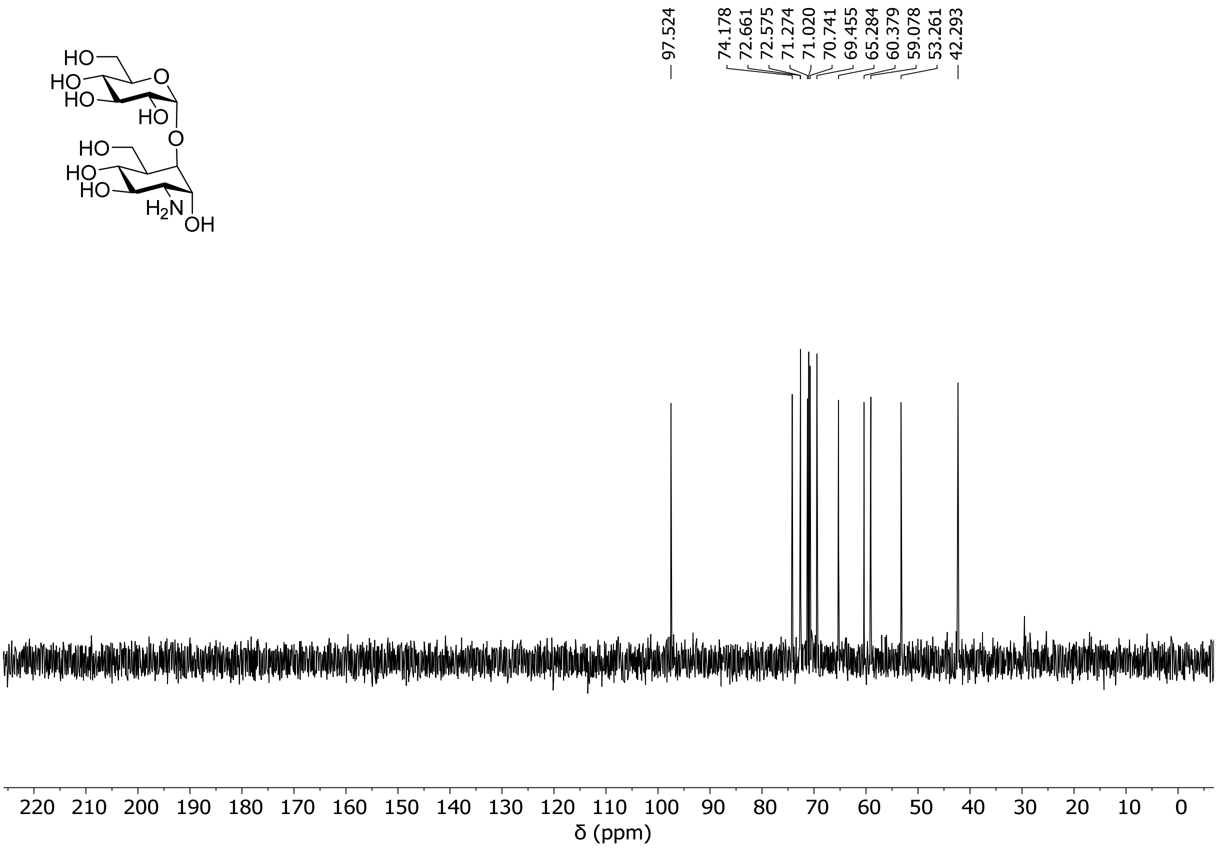

^{13}C{^{1}H}-NMR (121 MHz, D_2O) spectrum of **56**

5 References

(1) Matzner, D.; Schüller, A.; Seitz, T.; Wittmann, V.; Mayer, G. *Chem. Eur. J.* **2017**, *23*, 12604-12612.
(2) Stängle, D.; Silkenath, B.; Gehle, P.; Esser, A.; Mayer, G.; Wittmann, V. *Chem. Eur. J.* **2023**, e202202378.
(3) Nicolaou, K. C.; van Delft, F. L.; Conley, S. R.; Mitchell, H. J.; Jin, Z.; Rodríguez, R. M. *J. Am. Chem. Soc.* **1997**, *119*, 9057-9058.
(4) Zeng, J.; Vedachalam, S.; Xiang, S.; Liu, X.-W. *Org. Lett.* **2011**, *13*, 42-45.
